# Torix *Rickettsia* are widespread in arthropods and reflect a neglected symbiosis

**DOI:** 10.1093/gigascience/giab021

**Published:** 2021-03-25

**Authors:** Jack Pilgrim, Panupong Thongprem, Helen R Davison, Stefanos Siozios, Matthew Baylis, Evgeny V Zakharov, Sujeevan Ratnasingham, Jeremy R deWaard, Craig R Macadam, M Alex Smith, Gregory D D Hurst

**Affiliations:** Institute of Infection, Veterinary and Ecological Sciences, Faculty of Health and Life Sciences, University of Liverpool, Leahurst Campus, Chester High Road, Neston, Wirral CH64 7TE, UK; Institute of Infection, Veterinary and Ecological Sciences, Faculty of Health and Life Sciences, University of Liverpool, Leahurst Campus, Chester High Road, Neston, Wirral CH64 7TE, UK; Institute of Infection, Veterinary and Ecological Sciences, Faculty of Health and Life Sciences, University of Liverpool, Leahurst Campus, Chester High Road, Neston, Wirral CH64 7TE, UK; Institute of Infection, Veterinary and Ecological Sciences, Faculty of Health and Life Sciences, University of Liverpool, Leahurst Campus, Chester High Road, Neston, Wirral CH64 7TE, UK; Institute of Infection, Veterinary and Ecological Sciences, Faculty of Health and Life Sciences, University of Liverpool, Leahurst Campus, Chester High Road, Neston, Wirral CH64 7TE, UK; Health Protection Research Unit in Emerging and Zoonotic Infections, University of Liverpool, 8 West Derby Street, Liverpool L69 7BE, UK; Centre for Biodiversity Genomics, University of Guelph, 50 Stone Road East, Guelph, Ontario N1G2W1, Canada; Centre for Biodiversity Genomics, University of Guelph, 50 Stone Road East, Guelph, Ontario N1G2W1, Canada; Centre for Biodiversity Genomics, University of Guelph, 50 Stone Road East, Guelph, Ontario N1G2W1, Canada; Buglife – The Invertebrate Conservation Trust, Balallan House, 24 Allan Park, Stirling FK8 2QG, UK; Department of Integrative Biology, University of Guelph, Summerlee Science Complex, Guelph, Ontario N1G 2W1, Canada; Institute of Infection, Veterinary and Ecological Sciences, Faculty of Health and Life Sciences, University of Liverpool, Leahurst Campus, Chester High Road, Neston, Wirral CH64 7TE, UK

**Keywords:** Rickettsia, symbiosis: arthropods, endosymbiont, DNA barcoding

## Abstract

**Background:**

*Rickettsia* are intracellular bacteria best known as the causative agents of human and animal diseases. Although these medically important *Rickettsia* are often transmitted via haematophagous arthropods, other *Rickettsia*, such as those in the Torix group, appear to reside exclusively in invertebrates and protists with no secondary vertebrate host. Importantly, little is known about the diversity or host range of Torix group *Rickettsia*.

**Results:**

This study describes the serendipitous discovery of *Rickettsia* amplicons in the Barcode of Life Data System (BOLD), a sequence database specifically designed for the curation of mitochondrial DNA barcodes. Of 184,585 barcode sequences analysed, *Rickettsia* is observed in ∼0.41% of barcode submissions and is more likely to be found than *Wolbachia* (0.17%). The Torix group of *Rickettsia* are shown to account for 95% of all unintended amplifications from the genus. A further targeted PCR screen of 1,612 individuals from 169 terrestrial and aquatic invertebrate species identified mostly Torix strains and supports the “aquatic hot spot” hypothesis for Torix infection. Furthermore, the analysis of 1,341 SRA deposits indicates that Torix infections represent a significant proportion of all *Rickettsia* symbioses found in arthropod genome projects.

**Conclusions:**

This study supports a previous hypothesis that suggests that Torix *Rickettsia* are overrepresented in aquatic insects. In addition, multiple methods reveal further putative hot spots of Torix *Rickettsia* infection, including in phloem-feeding bugs, parasitoid wasps, spiders, and vectors of disease. The unknown host effects and transmission strategies of these endosymbionts make these newly discovered associations important to inform future directions of investigation involving the understudied Torix *Rickettsia*.

## Background

It is now widely recognized that animals live in a microbial world and that many aspects of animal biology, ecology, and evolution are a product of their symbioses with microorganisms [1]. In invertebrates, these symbioses may be particularly intimate and involve transmission of the microbe from parent to offspring [2]. The alignment of host reproduction with symbiont transmission produces a correlation between the fitness interests of the parties, reflected in symbionts evolving to play a number of physiological roles within the host, from defence [3, 4] through to core anabolic and digestive functions [5, 6]. However, the maternal inheritance of these microbes has led to the retention of parasitic phenotypes associated with distortion of reproduction, with symbiont phenotypes including biases towards daughter production and cytoplasmic incompatibility [7]. These diverse individual impacts alter the ecology and evolution of the host, in terms of diet, dynamics of interaction with natural enemies, sexual selection, and speciation.

Heritable symbioses have evolved on multiple occasions amongst microbial taxa. In some cases, the microbial lineage is limited to a single clade of related animal hosts, such as *Buchnera* in aphids [8]. In other cases, particular heritable microbes are found across a wide range of arthropod species. *Wolbachia* represents the most common associate, considered to infect nearly half of all species [9], and this commonness is a function in part of the ability of *Wolbachia* to transfer to a broad range of new host species and spread within them (host shift events) [10]. Aside *Wolbachia*, other microbes are found commonly as heritable symbionts of arthropod hosts [11]. *Cardinium* and *Rickettsia*, for instance, have been estimated at being present in 13–55% and 20–42% of terrestrial arthropod species, respectively [12].

In this article, we address the diversity and commonness of symbioses between *Rickettsia* and arthropods. The *Rickettsia* have increasingly been recognized as a genus of bacteria with diverse interactions with arthropods [13, 14]. First discovered as the agents underlying several diseases of humans vectored by haematophagous arthropods [15, 16], our understanding of the group changed in the 1990s with the recognition that *Rickettsia* were commonly arthropod symbionts [17, 18]. *Rickettsia* were recognized first as male-killing reproductive parasites [17, 19] and then later as beneficial partners [3, 20, 21].

Following this extension of our understanding of *Rickettsia*-arthropod interactions, a new clade of *Rickettsia* was discovered from work in *Torix* leeches [22, 23]. This clade was sister to all other *Rickettsia* genera and contained 2 subgroups (Leech and Limoniae [24]), with no evidence to date of any strain having a vertebrate pathogen phase. The host range for Torix *Rickettsia* is broader than that for other members of the genus, going beyond arthropods to include amoeba hosts [25, 26]. Targeted PCR-based screening has revealed Torix group *Rickettsia* as particularly common in 3 groups with aquatic association: *Culicoides* biting midges, deronectid beetles, and odonates [24, 27, 28]. However, some previous hypothesis-free PCR screens that aimed to detect *Rickettsia* in arthropods have likely missed these symbioses, owing to divergence of the marker sequence and mismatch with the primers [29].

During our previous work on Torix *Rickettsia* in biting midges [27], we became aware of the presence of *Rickettsia* cytochrome c oxidase I (*COI*) sequences deposited in GenBank that derived from studies where the intended target of amplification/sequencing was mitochondrial *COI*. These deposits derived from studies using mitochondrial DNA (mtDNA) barcoding for phylogeographic inference [30], or in barcoding-based species identification approaches [31, 32]. Non-target amplification of *Rickettsia COI* using mitochondrial *COI* barcoding primers has been reported in spiders [31, 32] and freshwater amphipods [30, 33]. Furthermore, we have noted 2 cases in our laboratory where amplicons obtained for mtDNA barcoding of an arthropod have, on sequence analysis, revealed *Rickettsia COI* amplification (Belli group *Rickettsia* from Collembola, and Torix group *Rickettsia* from *Cimex lectularius* bedbugs). Previous work had established that barcoding approaches may amplify *COI* from *Wolbachia* symbionts [34], and the aforementioned data indicated that non-target *Rickettsia COI* may be likewise amplified during this PCR amplification for mitochondrial *COI*.

In this article, we use 3 approaches to reveal the diversity and commonness of Torix *Rickettsia* in arthropods. First, we probed a bin from the Barcode of Life Data System (BOLD [35]), containing non-target *COI* sequences, for *Rickettsia* amplicons and then used the DNA extracts from these projects to define the diversity of *Rickettsia* observed using a multilocus approach. Second, we screened DNA extracts from multiple individuals from 169 invertebrate species for *Rickettsia* presence to determine the distribution of the symbiont in both terrestrial and aquatic biomes. Finally, we used bioinformatic approaches to examine the SRA depositions for 1 individual from 1,341 arthropod species for the presence of *Rickettsia* and used this as a means of estimating the relative balance of Torix group to other *Rickettsia* within symbioses.

## Data Description

### Barcode of Life Data System

**Figure 1:**
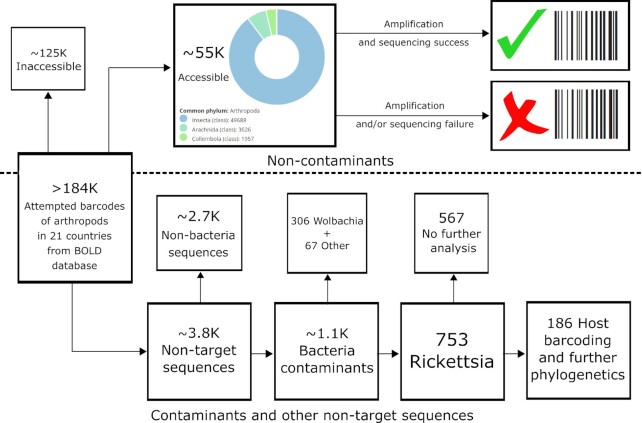
Workflow of the BOLD project demonstrating the acquisition and fates of contaminant and non-contaminant *COI* barcoding sequences.

While searching BOLD, a depository of >8 million *COI* mtDNA sequences, hundreds of hits were observed with high sequence similarity to Torix group *Rickettsia*. To investigate the diversity and host distribution of these non-target amplicons, access was permitted to analyse *COI* barcoding data deriving from a BOLD screening project totalling 184,585 arthropod specimens (including individuals where barcoding had failed) from 21 countries that had been collected between 2010 and 2014. *COI* sequences provided by BOLD were generally derived from DNA extracts created from somatic tissues (legs are often used in order to retain most of the specimen for further analyses if necessary) but also rarely included abdominal tissues. The first dataset made available [36] included 3,817 specimens containing sequences not matching initial morphological assignment (and likely to contain contaminant sequences). The second dataset included 55,366 specimens judged not to contain non-target amplicons [37]. A remaining 125,402 specimens were not made available, and the 55,366 subsample was used as a representative sample from which the contaminants had originated (Fig. 1). The protocols for data collection, data curation, and quality control of submitted BOLD samples are described by Ratnasingham and Hebert [38].

### Sequence read archive

Further insights into the balance of *Rickettsia* groups within arthropod symbioses were obtained through searching for *Rickettsia* presence in Illumina datasets associated with arthropod whole-genome sequencing projects in the SRA (60,409 records as of 20 May 2019). To reduce the bias from overrepresented laboratory model species (e.g., *Drosophila* spp., *Anopheles* spp.) a single dataset per species was examined, and where multiple datasets existed for a species, that with the largest read count was retained. The resultant dataset [39], representing 1,341 arthropod species, was then screened with phyloFlash [40], which finds, extracts, and identifies single-subunit (SSU) ribosomal RNA (rRNA) sequences.

### Targeted screen of aquatic and terrestrial arthropods

Both the BOLD and SRA datasets have inherent biases that make them unsuitable to assess whether Torix *Rickettsia* are more common in aquatic or terrestrial biomes. For example, most SRA submissions are from laboratory-reared terrestrial insects. Likewise, a majority of the BOLD specimens containing *Rickettsia* have limited taxonomic and ecological information, by virtue of not returning an mtDNA *COI* sequence. Therefore, a targeted PCR screen of 1,612 individuals from 169 species was undertaken (Tables 1 and 2) using primers that hybridize with all known clades of *Rickettsia* [27]. Within this, we included a range of both aquatic and terrestrial taxa to investigate whether the previous work highlighting particular aquatic taxa as hot spots for *Rickettsia* symbiosis (water beetles, biting midges, damselflies) reflects a wider higher incidence in species from this habitat.

**Table 1:** Targeted *Rickettsia* screen of aquatic/semiaquatic invertebrates

Aquatic/semiaquatic invertebrate group	Species	Location	Year	No. tested	No. positive
Ephemeroptera	*Baetis muticus*	Stirling, Scotland, UK	2017	3	0
	*Baetis rhodani*	Stirling, Scotland, UK	2017	3	0
	*Cloeon dipterum*	Cheshire, UK	2016	3	0
	*Ecdyonurus* sp. 1	Stirling, Scotland, UK	2017	5	0
	*Ecdyonurus* sp. 2	Cheshire, UK	2016	3	0
	*Ecdyonurus venosus*	Cheshire, UK	2016	6	0
	*Leptophlebia vespertina*	Hampshire, UK	2016	1	0
	*Paraleptophlebia submarginata*	Stirling, Scotland, UK	2017	3	0
	*Rhithrogena semicolorata*	Stirling, Scotland, UK	2017	3	0
Trichoptera	*Hydropsyche* sp.	Stirling, Scotland, UK	2017	3	0
	*Polycentropus flavomaculatus*	Cheshire, UK	2017	3	0
	***Rhyacophila dorsalis***	**Stirling, Scotland, UK**	**2017**	**3**	**2**
Plecoptera	*Amphinemura sulcicollis*	Stirling, Scotland, UK	2017	3	0
	*Dinocras cephalotes*	Stirling, Scotland, UK	2017	3	0
	*Isoperla grammatica*	Stirling, Scotland, UK	2017	3	0
	*Perla bipunctata*	Stirling, Scotland, UK	2017	3	0
Hemiptera	*Corixa punctata*	Cheshire, UK	2016	1	0
	*Gerris* sp.	Montferrier sur Lez, France	2006	12	0
	*Gerris thoracicus*	Cheshire, UK	2016	1	0
	*Hydrometra stagnorum*	Montferrier sur Lez, France	2006	20	0
	*Nepa cinerea*	Montferrier sur Lez, France	2006	3	0
	*Notonecta glauca*	Cheshire, UK	2016	2	0
	*Plea minutissima*	Notre Dame de Londres, France	2006	8	0
	*Sigara lateralis*	Notre Dame de Londres, France	2006	6	0
	***Sigara striata***	**Cheshire, UK**	**2006**	**2**	**1**
Diptera	*Aedes* sp.	Cheshire, UK	2017	8	0
	*Aedes albopictus*	Roma, Italy	2005	20	0
	***Anopheles plumbeus***	**Chester Zoo, UK**	**2018**	**2**	**2**
	**Chironomidae sp**.	**Cheshire, UK**	**2016**	**4**	**1**
	*Chironomus acidophilus*	Cheshire, UK	2017	1	0
	*Chironomus plumosus*	Notre Dame de Londres, France	2006	20	0
	*Chironomus* sp.	Cheshire, UK	2016	4	0
	*Culex pipiens (ssp. quinquefasciatus)*	Puerto Viejo de Talamanca, Costa Rica	2006	20	0
	*Culex pipiens*	St Nazaire de Pézan, France	2006	20	0
	*Eristalinus* sp.	Cheshire, UK	2016	3	0
	*Eristalis tenax*	Montpellier (grotte du zoo), France	2002	7	0
	***Glyptotendipes* sp**.	**Cheshire, UK**	**2016**	**1**	**1**
	***Hilarainterstincta***	**Cheshire, UK**	**2017**	**3**	**1**
	***Simulium aureum***	**Hampshire, UK**	**2017**	**1**	**1**
	*Simulium ornatum*	N/A	2003	12	0
	*Tipula* sp.	UK	2006	10	0
	*Tipula oleracea*	UK	2006	13	0
	***Zavrelimyia* sp**.	**Northumberland, UK**	**2017**	**1**	**1**
Coleoptera	*Agabus bipustulatus*	Cheshire, UK	2017	3	0
	*Guignotus pusillus*	Notre Dame de Londres, France	2006	12	0
	Unknown sp.1	Cheshire, UK	2017	2	0
	Unknown sp.2	Cheshire, UK	2017	3	0
Acarina	Unknown sp.	Cheshire, UK	2017	3	0
Isopoda	*Asellus aquaticus*	Cheshire, UK	2016	3	0
Amphipoda	*Gammarus pulex*	Stirling, Scotland, UK	2017	3	0
	*Crangonyx pseudogracilis*	Cheshire, UK	2016	6	0
Gastropoda	*Radix balthica*	Cheshire, UK	2016	3	0
	*Planorbis* sp.	Cheshire, UK	2016	3	0
	***Galba truncatula***	**Cheshire, UK**	**2017**	**20**	**3**
Hirudinea	*Erpobdella octoculata*	Cheshire, UK	2016	2	0
	*Hemiclepsis marginata*	Cheshire, UK	2017	1	0
Tricladida	Unknown sp.	Cheshire, UK	2016	1	0

A species was deemed positive through PCR and designated to *Rickettsia* group after Sanger sequencing and phylogenetic placement. All strains belong to the Torix group. Bold entries indicate *Rickettsia*-positive hosts identified in this study.

**Table 2:** Targeted *Rickettsia* screen of terrestrial invertebrates

Terrestrial invertebrate group	Species	Location	Year	No. tested	No. positive
Araneae	*Agelenopsis aperta*	Tennessee, USA	N/A	12	0
	*Allopecosa pulverulenta*	Berne, Germany	N/A	16	0
	***Amaurobius fenestralis***	**Montpellier, France**	**2006**	**16**	**1**
	*Araneus diadematus*	Beerse, Belgium	N/A	19	0
	*Araneus diadematus*	Greater London, UK	N/A	8	0
	*Argiope bruennichi*	Hamburg, Germany	N/A	7	0
	*Argiope lobata*	Spain	N/A	7	0
	*Argiope lobata*	Israel	N/A	4	0
	*Cyclosa conica*	Brandenburg, Germany	N/A	11	0
	*Dysdera crocata*	Montpellier, France	2006	2	0
	*Enoplognatha ovata*	Greater London, UK	N/A	20	0
	*Erigone atra*	Cheshire, UK	2017	1	0
	*Evarcha falcata*	Beerse, Belgium	N/A	5	0
	*Holochnemus pluchei*	Montpellier, France	2006	7	0
	***Hylyphantes graminicola***	**Cheshire, UK**	**2017**	**1**	**1**
	*Larinioides cornutus*	Greater London, UK	N/A	6	0
	*Larinoides sclopetarius*	Hamburg, Germany	N/A	17	0
	***Linyphia triangularis***	**Berlin, Germany**	**N/A**	**9**	**9**
	*Linyphia triangularis*	Greater London, UK	N/A	6	0
	*Lycosa* sp.	Cheshire, UK	2017	2	0
	*Metellina mengei*	Greater London, UK	N/A	13	0
	*Metellina segmentata*	Brandenburg, Germany	N/A	9	0
	*Neriene clathrata*	Beerse, Belgium	N/A	13	0
	*Neriene peltata*	Cheshire, UK	2017	1	0
	*Pachygnatha degeeri*	Berne, Germany	N/A	11	0
	*Pachygnatha listeri*	Beerse, Belgium	N/A	17	0
	***Pardosa lugubris***	**Darmstadt, Germany**	**N/A**	**20**	**1**
	*Pardosa pullata*	Brandenburg, Germany	N/A	20	0
	*Pardosa purbeckensis*	Belgium	N/A	19	0
	***Pholcus phalangioides***	**Berlin, Germany**	**N/A**	**20**	**17**
	***Pisaura mirabilis***	**Greater London, UK**	**N/A**	**12**	**1**
	*Tetragnatha montana*	Greater London, UK	N/A	20	0
	*Tetragnatha* sp.	Hampshire, UK	2017	3	0
	Unknown sp.	Cheshire, UK	2017	2	0
	*Xysticus cristatus*	Cambridgeshire, UK	N/A	16	0
Opiliones	*Leiobunum rotundum*	Feurs, France	2006	6	0
Ixodida	*Ixodes uriae*	Hornøya, Norway	2005	19	0
	*Rhipicephalus microplus*	New Caledonia	2003	1	0
Scorpiones	*Euscorpius flavicauda*	St Nazaire de Pézan, France	2006	1	0
Diplopoda	*Ommatoiulu*s sp.	Cheshire, UK	2016	1	0
Neuroptera	Unknown sp.	Cheshire, UK	2017	1	0
Mecoptera	*Panorpa* sp.	Cheshire, UK	2017	2	0
Orthoptera	*Calliptamus italicus*	Notre Dame de Londres, France	2016	18	0
	*Chorthippus brunneus*	Uk	2006	20	0
	*Gryllomorpha dalmatina*	Montpellier, France	2006	2	0
Blattaria	*Loboptera decipiens*	Montpellier, France	2006	17	0
Mantodae	*Iris oratoria*	St Nazaire de Pézan, France	2006	6	0
	*Mantis religiosa*	Feurs, France	2006	3	0
Dermaptera	*Forficula Auricularia*	Feurs, France	2006	9	0
Hemiptera	*Aphis fabae*	Montpellier, France	2006	12	0
	*Aphis nerii*	Montpellier, France	2006	8	0
	*Baizongia pistaciae*	Viols le Fort, France	2006	12	0
	*Cicadella viridis*	L'Olme, France	2006	16	0
	***Cimex lectularius***	**Yorkshire, UK**	**2008**	**12**	**12**
	*Elasmucha grisea*	Greater London, UK	2006	16	0
	*Graphosoma italicum*	Montpellier, France	2006	12	0
	*Lygaeus equestris*	Montpellier, France	2006	12	0
	*Notostira elongata*	L'Olme, France	2006	11	0
	*Pyrrhocoris apterus*	Montpellier, France	2006	11	0
	*Rhyparochromus vulgaris*	Castelnaudary, France	2006	20	0
Coleoptera	*Anaspis frontalis*	Mont Barri, France	2004	12	0
	*Anthaxia nitidula*	Mont Barri, France	2004	20	0
	*Anthaxia* sp.	Mont Barri, France	2004	16	0
	*Calvia 14-guttata*	Greater London, UK	2006	6	0
	*Capnodis tenebrionis*	Montpellier, France	2006	1	0
	*Cetonia aurata*	Feurs, France	2006	3	0
	*Cetonia aurata*	Mont Barri, France	2004	12	0
	*Chrysolina varians*	Mont Barri, France	2004	18	0
	*Clytus arietis*	Mont Barri, France	2004	20	0
	*Dermestes* sp.	Mont Barri, France	2004	20	0
	*Dermestes tessellatocollis*	Cheshire, UK	2016	2	0
	*Gastrophysa* sp.	Greater London, UK	2006	20	0
	*Geotrupes stercorarius*	Mont Barri, France	2004	3	0
	*Larinus scolymi*	Aldira de Irmeros, Spain	2005	12	0
	*Leptinotarsa decemlineata*	Feurs, France	2006	10	0
	*Mordellistena* sp.	Mont Barri, France	2004	10	0
	*Oedemera* sp.	Mont Barri, France	2004	20	0
	*Oncocerna* sp.	Mont Barri, France	2004	20	0
	***Phyllobius argentatus***	**Mont Barri, France**	**2004**	**15**	**4†**
	*Pseudovadonia livida*	Mont Barri, France	2004	19	0
	*Stenopterus* sp.	Mont Barri, France	2004	20	0
Diptera	*Braula coeca*	Ouessant, France	2002	4	0
	*Chorisops tunisiae*	Montpellier, France	2003	8	0
	*Delia antiqua*	N/A	N/A	11	0
	*Delia platura*	N/A	N/A	11	0
	*Delia radiacum*	N/A	N/A	10	0
	*Gasterophilus intestinalis*	France	N/A	10	0
	*Hippobosca equina*	Restinclières, France	2006	15	0
	*Lonchoptera lutea*	Cheshire, UK	2017	3	0
	*Medetera petrophila*	St Bauzille de Putois, France	2003	12	0
	*Musca domestica*	L'Olme, France	2006	20	0
	*Musca vitripennis*	Notre Dame de Londres, France	2003	8	0
	*Neomyia cornicina*	Notre Dame de Londres, France	2003	8	0
	*Protocalliphora* sp.	Corse, France	2003	2	0
	***Protocalliphora azurea***	**Montpellier, France**	**2005**	**12**	**12**
	*Psila rosae*	N/A	N/A	11	0
	*Stomoxys calcitrans*	Le Malzieu, France	2001	11	0
Lepidoptera	*Chilo phragmitellus*	Feurs, France	2006	10	0
	*Euplagia quadripunctaria*	Feurs, France	2006	2	0
	*Pieris brassicae*	Feurs, France	2006	7	0
	*Plodia interpunctella*	Montpellier, France	2006	12	0
	*Thymelicus lineola*	Greater London, UK	2006	15	0
	*Thymelicus sylvestris*	Greater London, UK	2006	2	0
	*Triodia sylvina*	Montpellier, France	2006	4	0
Hymenoptera	*Amblyteles armatorius*	St Nazaire de Pézan, France	2006	1	0
	*Amegilla albigena*	St Nazaire de Pézan, France	2006	13	0
	*Amegilla ochroleuca*	St Nazaire de Pézan, France	2006	3	0
	*Anthidium florentinum*	St Nazaire de Pézan, France	2006	6	0
	*Apis mellifera*	UK	2006	9	0
	*Bombus terrestris*	Northwest Switzerland	2006	20	0
	*Diplolepis rosae*	L'Olme, France	2006	2	0
	*Formica lugubris*	UK	2006	10	0
	***Pachycrepoideus* sp**.	**UK**	**N/A**	**94**	**6‡**
	*Polistes dominulus*	St Nazaire de Pézan, France	2006	4	0
	*Polistes nimpha*	St Nazaire de Pézan, France	2006	19	0
	*Sceliphron caementarium*	St Nazaire de Pézan, France	2006	3	0

A species was deemed positive through PCR and designated to *Rickettsia* group after Sanger sequencing and phylogenetic placement. All strains belong to the Torix group except † = Rhyzobius and ‡ = Belli. Bold entries indicate *Rickettsia*-positive hosts identified in this study. N/A: not available.

## Analyses

### Torix *Rickettsia* is the most common bacterial contaminant sequence currently in BOLD, a major barcoding project

**Figure 2:**
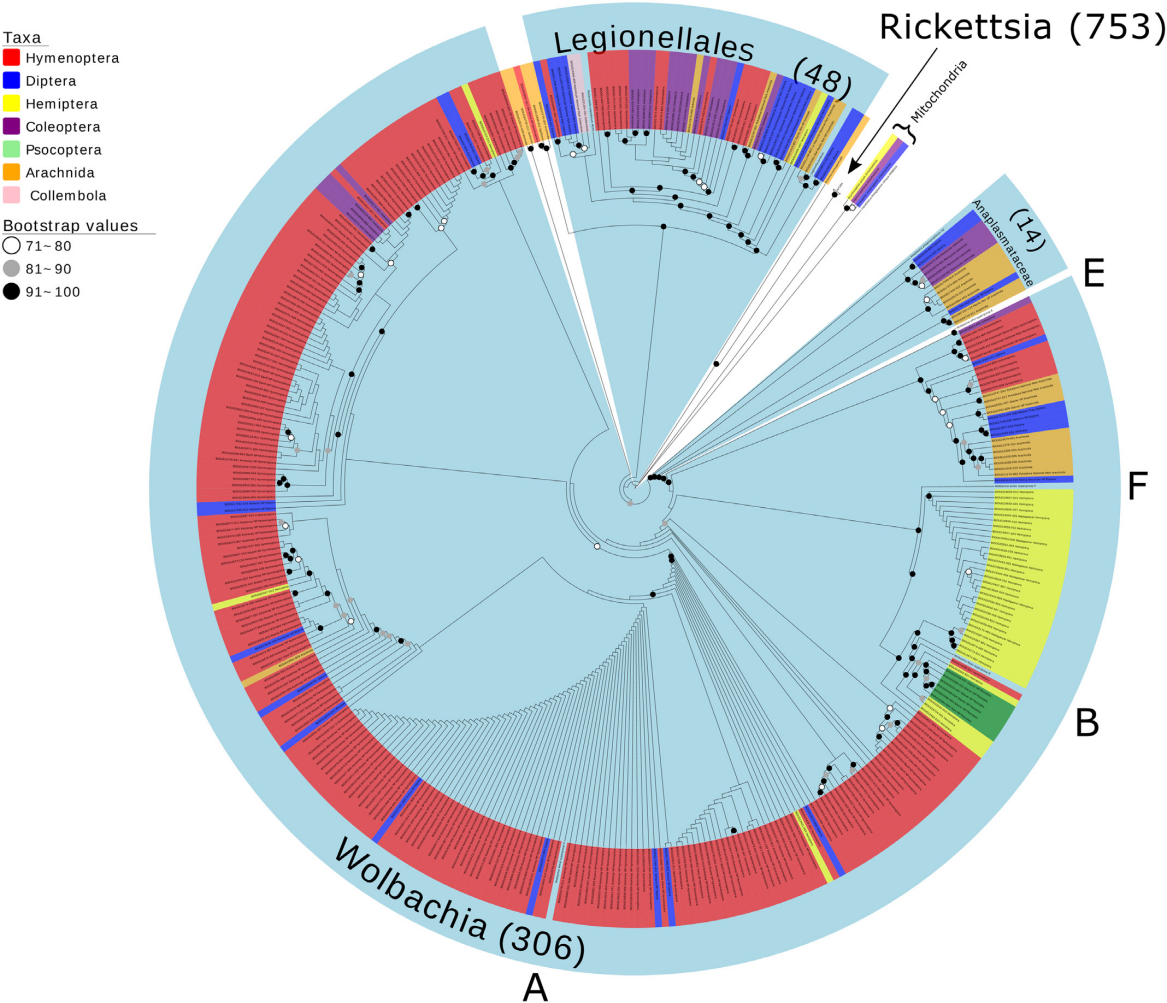
Cladogram of the maximum likelihood (ML) tree of 1,126 proteobacteria *COI* contaminants retrieved from a BOLD project incorporating 184,585 arthropod specimens. The tree is based on 561 bp and is rooted with the free-living alphaproteobacteria *Pelagibacter ubique*. Parentheses indicate the number of BOLD contaminants present in each group. Tips are labelled by BOLD processing ID and host arthropod taxonomy. The Rickettsiales genera of *Anaplasma, Rickettsia* (collapsed node)*, Orientia*, and *Wolbachia* supergroups (A, B, E, and F), as well as the Legionellales genera *Legionella* and *Rickettsiella*, are included as reference sequences (Accession numbers: Additional file 10).

Amongst 3,817 sequences considered as not matching initial morphological assignment, 1,126 of these were deemed by BOLD to be bacterial in origin (Fig. 1 [36]). The taxonomic classification tool, Kaiju, further supported bacterial designation for all sequences except 1 (Additional file 1), although this was later confirmed as *Rickettsia* through phylogenetic placement. Phylogenetic placement further confirmed the correct designation of bacterial sequences (Fig. 2 and Additional file 2). The dominant genus was *Rickettsia* with 753 (66.9%) amplifications, compared to *Wolbachia* with 306 (27.2%). Of the remaining 67 non-target sequences, 14 formed a monophyletic group with other Anaplasmataceae and 48 clustered with the order Legionellales, with 5 sequences remaining undesignated. When considering the 184,585 specimens in the total project, this analysis gave an overall *Rickettsia* and *Wolbachia* frequency of 0.41% and 0.17%, respectively, within the dataset. Through later access to the 55,366 representative data subset from which the contaminants originated, a further 245 unique bacteria contaminants were also detected by Kaiju (possibly missed by BOLD's automated contaminant filtering system) (Additional file 1). This additional finding suggests that these frequencies are conservative estimates.

**Figure 3:**
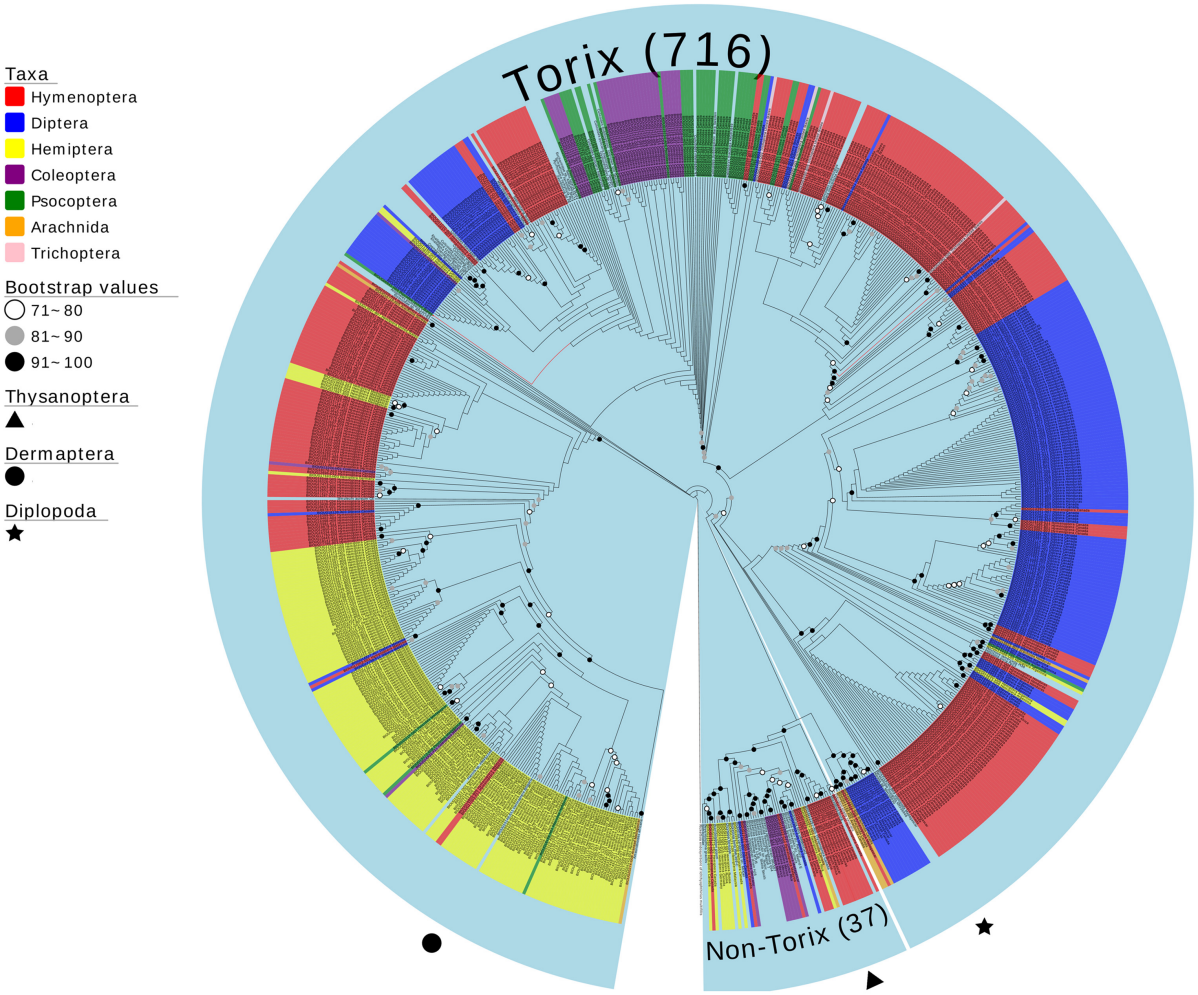
Cladogram of a maximum likelihood (ML) tree of 753 *COI Rickettsia* contaminants retrieved from a BOLD project incorporating 184,585 arthropod specimens. The tree is based on 561 bp and is rooted by the *Rickettsia* endosymbiont of *Ichthyophthirius multifiliis* (*Candidatus* Megaira) using the TVM+F+I+G4 model. Parentheses indicate the number of BOLD contaminants present in Torix and non-Torix *Rickettsia* groups. Tips are labelled by BOLD processing ID and host arthropod taxonomy. The *Rickettsia* groups Spotted Fever, Transitional, Belli, Typhus, Rhyzobius, and Torix are included as references (Accession numbers: Additional file 10).

BOLD *Rickettsia* contaminants were dominated by amplicons from the Torix group of *Rickettsia* (716 of 753 [95.1%]) (Fig. 3 and Additional file 2). The remaining 37 *Rickettsia* clustered with Transitional/Spotted Fever (n = 15), Belli (n = 9), and Rhyzobius (n = 1) groups, while 12 sequences formed 2 unique clades. Across arthropod hosts, 292 (38.8%) were derived from Hymenoptera; 189 (25.1%) from Diptera; 177 from Hemiptera (23.5%); 41 from Psocoptera (5.4%); 40 from Coleoptera (5.3%); 7 from Arachnida (0.9%); 4 from Trichoptera (0.5%); and single cases of Thysanoptera, Diplopoda, and Dermaptera (0.1% each).

We observed that 2 sets of *COI* primers were responsible for 99% of *Rickettsia* amplifications (Additional file 3) with a majority (89%) amplifying with the primer combination C_LepFolF/C_LepFolR [41]. Torix *Rickettsia COI* showed a stronger match to these primers at the 3′ end (the site responsible for efficient primer annealing) compared to *Wolbachia* and other *Rickettsia* groups. Whilst all contained a single-nucleotide polymorphism at the 3′ priming end of C_LepFolR, Torix *Rickettsia* (*Rickettsia* endosymbiont of *Culicoides newsteadi*; MWZE00000000) was the only sequence to not contain a single-nucleotide polymorphism at the 3′ priming site of C_LepFolF (Additional file 4).

### *Rickettsia* multilocus phylogenetic analysis

To better resolve the phylogenetic relationships between BOLD *Rickettsia* contaminants, a multilocus approach was used on a subsample of 186 *Rickettsia*-containing samples chosen on the basis of assorted geographic location, host order, and phylogenetic placement. To this end, 2 further housekeeping genes (*16S rRNA, gltA*) and the antigenic *17KDa* protein gene were amplified and sequenced from the respective DNA extracts.

**Figure 4:**
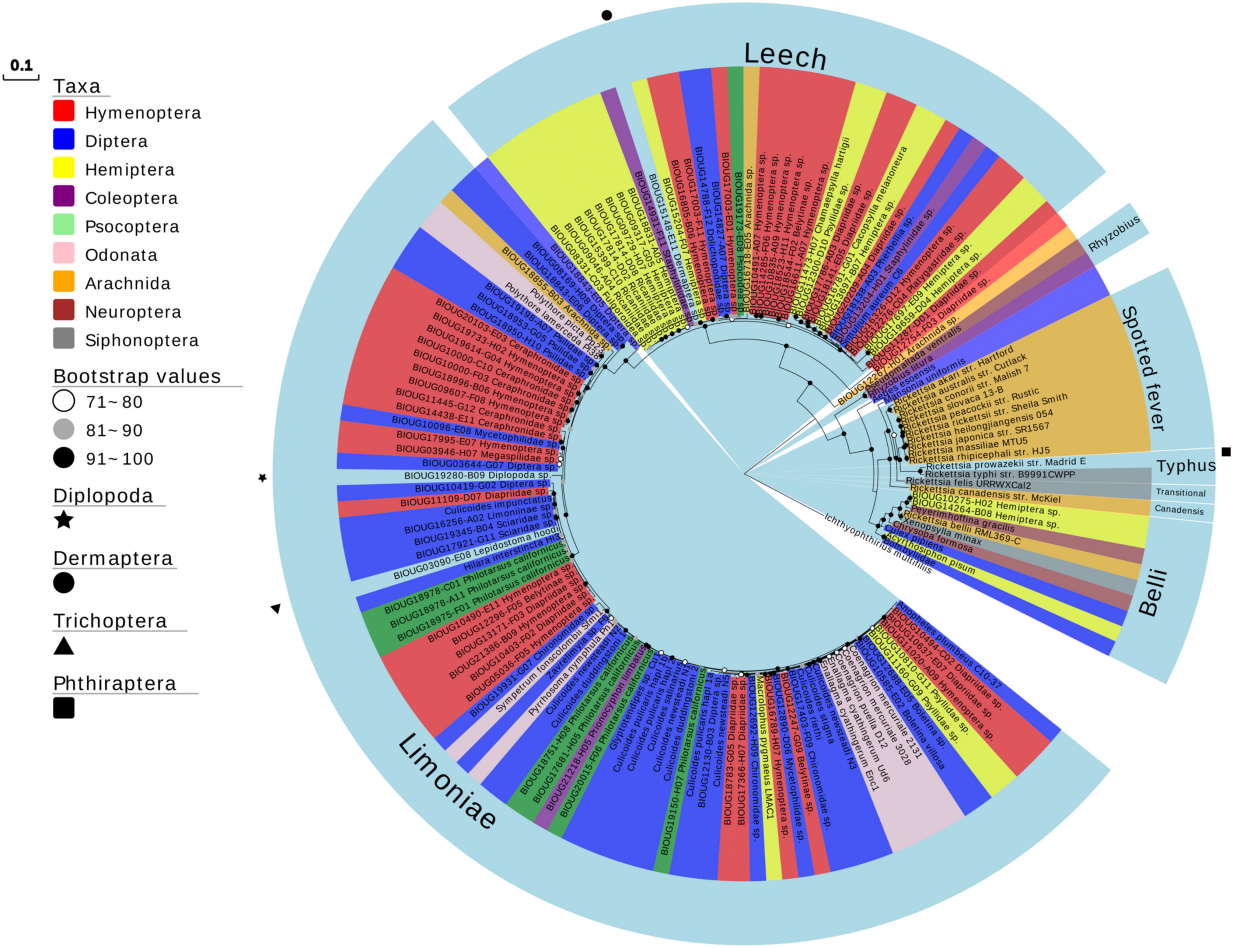
Phylogram of the maximum likelihood (ML) tree of 99 *COI Rickettsia* contaminants (prefix “BIOUG”) used for further phylogenetic analysis and 53 non-BOLD reference profiles (Accession numbers: Additional file 10). The tree is based on the concatenation of 4 loci, *16S rRNA, 17KDa*,  *gltA*, and *COI* under a partition model, with profiles containing ≥3 of 4 sites included in the tree (2,834 bp total) and is rooted by *Rickettsia* endosymbiont of *Ichthyophthirius multifiliis* (*Candidatus* Megaira). Tips are labelled by host arthropod taxonomy.

Overall, 135 extracts successfully amplified and gave a high-quality sequence for ≥1 gene. No intragenic or intergenic recombination was detected for any of the gene profiles. A phylogram, including 99 multilocus profiles containing ≥3 of the 4 *Rickettsia* genes of interest (including *COI*), allocated strains to both Limoniae and Leech subclades of the Torix group (Fig. 4) and these subclades were derived from similar hosts. For example, specific families (Hemiptera: Psyllidae and Hymenoptera: Diapriidae) were present in both Leech and Limoniae groups. Furthermore, similar strains were observed between genetically dissimilar host species. For example, the *Coenagrion mercuriale* (Odonata) strain was 100% identical to the *Culicoides stigma* (Diptera) strain across all 4 loci. This suggests that horizontal transfer of the symbiont is likely to be occurring. A full list of multilocus profiles and *Rickettsia* group designation can be found in Additional file 5.

The multilocus study also provided evidence of co-infection with *Rickettsia*. During Sanger chromatogram analysis, double peaks were occasionally found at third codon sites from protein-coding genes. This pattern was observed in 6 of 10 *Philotarsus californicus* individuals and in 1 member of each of the Psilidae, Sciaridae, Chironomidae, and Diapriidae (Additional file 5). Where double peaks were observed, this was found consistently across markers within an individual specimen. This pattern corroborates a recent finding of double infections in Odonates [28], suggesting that co-infecting *Rickettsia* strains in hosts is a widespread phenomenon of the Torix group.

### Barcoding success of *Rickettsia* host taxa

An available subset of specimens associated with the contaminants contained 55,366 of 184,585 arthropods originally used in the overall study [37]. The 3 classes of Insecta (n = 4, 688), Arachnida (n = 3,626), and Collembola (n = 1,957) accounted for >99.8% of total specimens (Fig. 1). Successful amplification and sequencing of *COI* was achieved in 43,246 specimens (78.1%) of the DNA extracts, but when assessed at the order level success rates varied (Additional file 6). The likely explanation for this variation is taxa-specific divergence of sequences at priming sites.

The number of each taxonomic order giving ≥1 *Rickettsia* amplification was then calculated and adjusted on the basis of the total number of specimens in the project to allow for a frequency estimate. Overall, Hymenoptera, Diptera, and Hemiptera were the 3 taxa most likely to be associated with *Rickettsia COI* amplification (87.4%). Similarly, on assessment of a subsample from the project where the contaminants originated, a majority (77.7%) of the dataset were also accounted for by these 3 orders. After adjusting the frequency to take into account the number of inaccessible specimens, Trichoptera (2.45%), Dermaptera (1.89%), and Psocodea (1.67%) were the most likely taxa to give an inadvertent *Rickettsia* amplification. Whilst Hemiptera and Diptera had a similar estimated frequency of *Rickettsia* amplification (0.58% and 0.56%), Hemiptera were much more likely to fail to barcode (67.2% vs 93.3%), suggesting that the rate of dipteran *Rickettsia* infection in BOLD specimens is likely to be higher than that of hemipterans, as a barcoding failure is necessary to amplify non-target bacteria *COI*. Attempts to re-barcode 186 *Rickettsia*-containing DNA extracts of interest from BOLD resulted in 90 successful arthropod host barcodes (Additional file 5).

### Targeted *Rickettsia*PCR screen and statistical comparison of terrestrial vs aquatic insects

**Figure 5:**
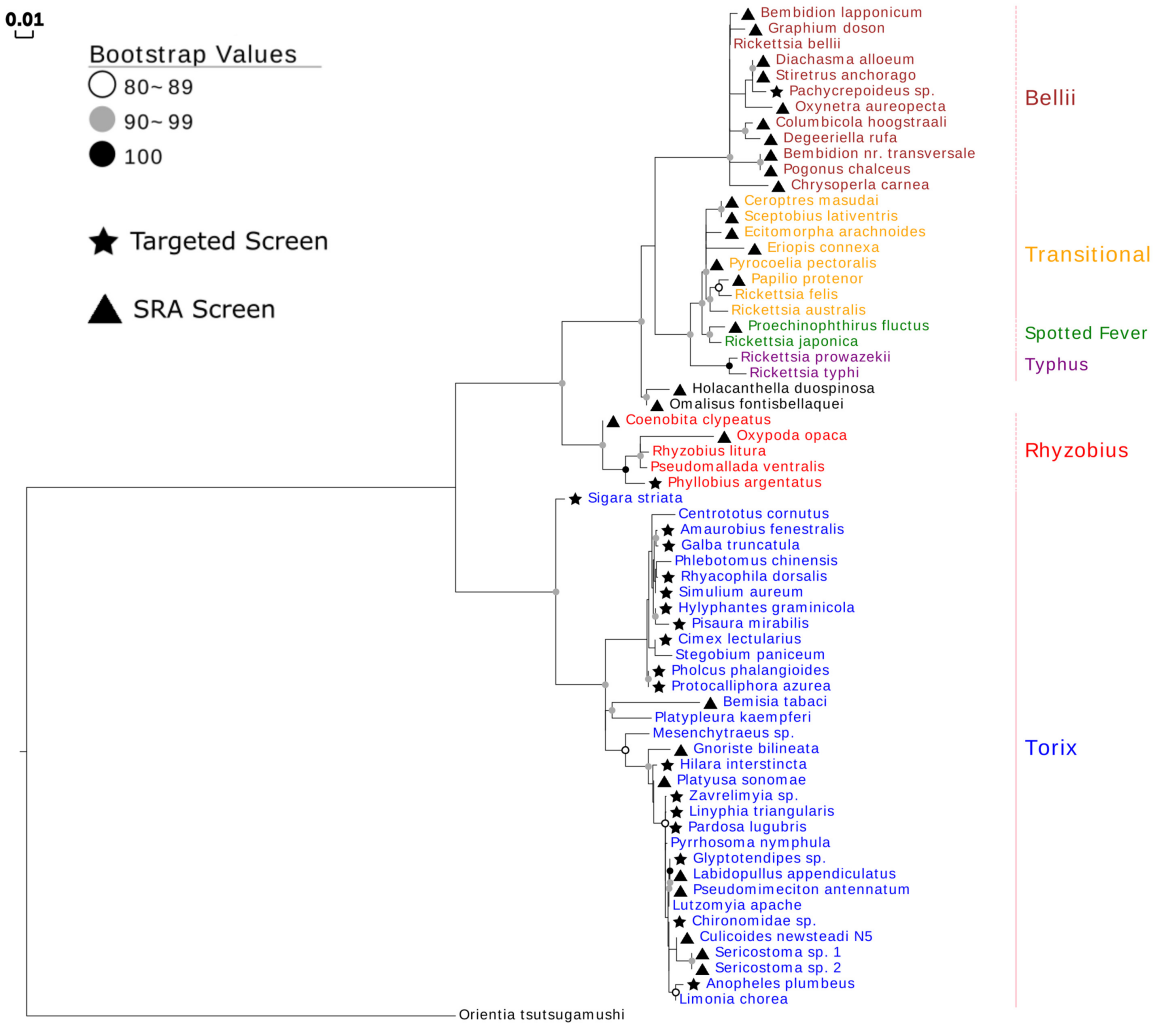
*16S rRNA* and *gltA* concatenated maximum likelihood (ML) phylogram (1,834 bp total) including *Rickettsia* hosts from SRA (triangles) and targeted screens (stars). The TIM3+F+R2 (16S) and K3Pu+F+G4 (gltA) models were chosen as best-fitting models. Rooting is with *Orientia tsutsugamushi*. Accession numbers found in Additional file 10.

From the targeted screen of 169 invertebrate species, a total of 19 *Rickettsia* were discovered from both aquatic and terrestrial pools, with 17 of these identified as belonging to the Torix group. The screening of aquatic invertebrates revealed that 9 of 57 species (16%) were positive in PCR assays (Tables 1 and 2). DNA sequences confirmed that all were *Rickettsia* that lay within the Torix group (Fig. 5), with the positive species deriving from 8 insect species and 1 mollusc. For the terrestrial invertebrates, PCR assays evidenced *Rickettsia* infection in 10 of 112 species (8.9%) with a mix of insect and spider hosts (4 and 6 species, respectively, Table 2). *Rickettsia* from 8 host species (2 insects and 6 spiders) were identified as Torix *Rickettsia* (8 of 112 species, 7.1%), while the other 2 host species carried *Rickettsia* from the Rhyzobius and Belli groups (Fig. 5).

To reduce taxonomic hot spot biases (particularly from spiders), we compared the incidence of *Rickettsia* infection in aquatic vs terrestrial insects. Fisher exact test analysis rejected the null hypothesis of equal representation, with aquatic taxa having a higher representation of species with Torix *Rickettsia* than terrestrial (*P* = 0.013, Additional file 7). Examining the phylogenetically controlled set, with 3 matched insect orders (Coleoptera, Diptera, Hemiptera), again rejected the null hypothesis of equal representation, with aquatic taxa having a higher representation of species with Torix *Rickettsia* than terrestrial (*P* = 0.025, Additional file 7). When comparing all invertebrate species from the targeted screen, no significant difference was observed in Torix *Rickettsia* incidence between terrestrial and aquatic biomes (*P* = 0.11, Additional file 7), suggesting that this pattern of infection may be specific to insects.

### SRA and GenBank *Rickettsia* searches

During the SRA search, phyloFlash flagged 29 *Rickettsia* sequences in the groups: Belli (n = 10), Torix (n = 8), Transitional (n = 6), Rhyzobius (n = 2), and Spotted Fever (n = 1), with the remaining 2 failing to form a monophyletic clade with any group (Fig. 5). In addition, Kraken identified 8 *Rickettsia*-containing arthropod SRA datasets missed by phyloFlash. Two of these were from the Torix group, in phantom midge hosts (Diptera: Chaoboridae: *Mochlonyx cinctipes* and *Chaoborus trivitattus*), with the remaining 6 placed in Belli and Spotted Fever groups [39].

phyloFLash was also used to retrieve 18S rRNA (eukaryotic) sequences that could potentially account for the *Rickettsia* observed in SRA datasets (e.g., through parasitisms or ingestion of *Rickettsia*-infected protists). Of the 29 datasets analysed by phyloFlash, only 1 (SRR6313831) revealed an assembled 18S rRNA sequence aligned to a parasitoid wasp (*Hadrotrichodes waukheon*). Although reads aligned to protists were also present in 19 of 29 datasets flagged by phyloFlash, the read depth for protists was much lower than the number of *Rickettsia* reads [39]. This suggests that *Rickettisa*-infected protists are unlikely to account for the positive results observed in the SRA datasets.

The search of GenBank revealed 11 deposits ascribed to host mtDNA that were in fact Torix *Rickettsia* sequences (Additional files 8 and 9).

### The hidden host diversity of Torix *Rickettsia*

**Figure 6:**
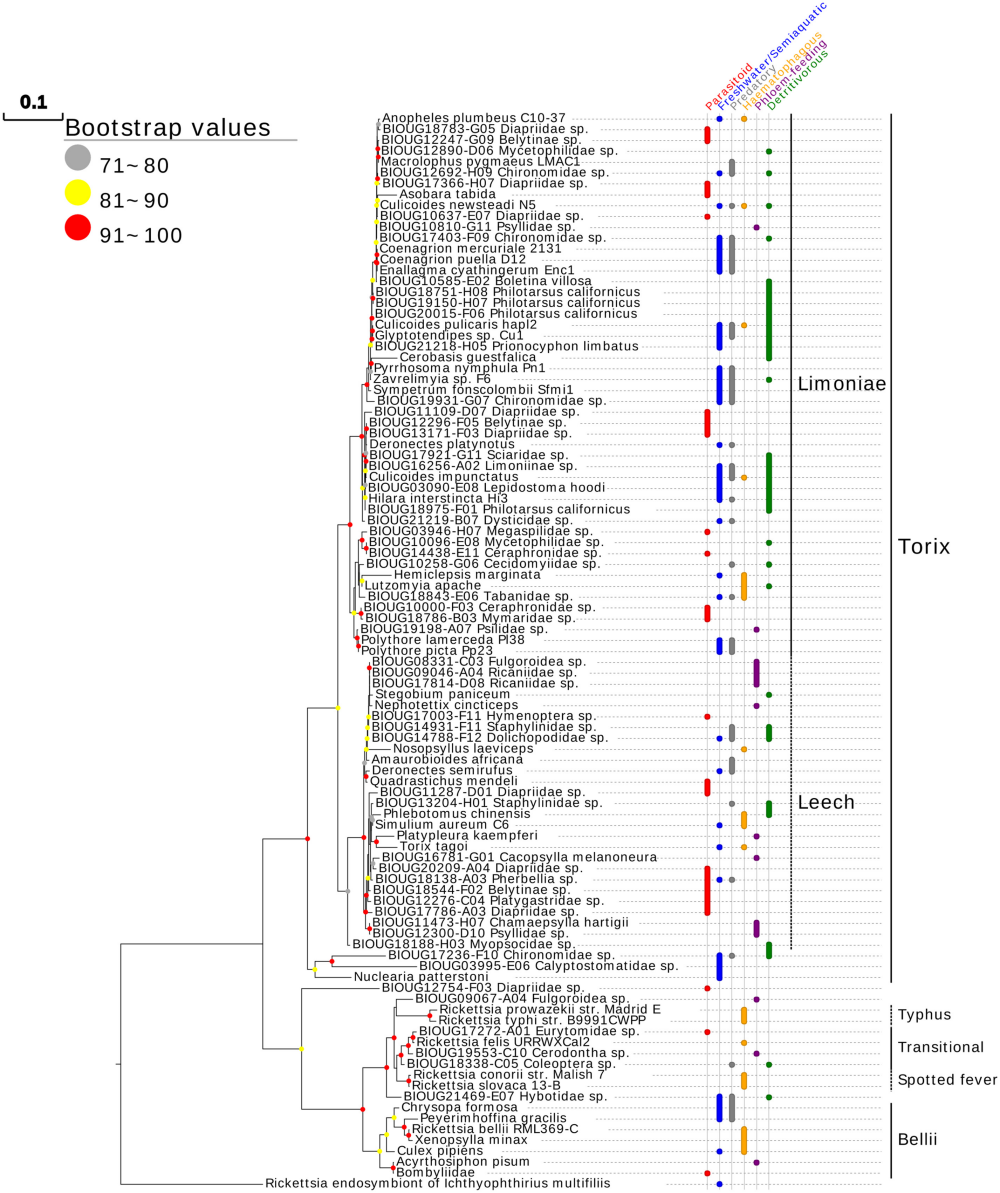
Phylogram of a maximum likelihood (ML) tree of *COI Rickettsia* contaminants (prefix “BIOUG”) giving a host barcode and 43 non-BOLD reference profiles. The tree is based on 4 loci, *16S rRNA, 17KDa*,  *gltA*, and *COI*, under a partition model with profiles containing ≥2 out of 4 sites included in the tree (2,781 bp total) and is rooted by the *Rickettsia* endosymbiont of *Ichthyophthirius multifiliis* (*Candidatus* Megaira). The habitats and lifestyles of the host are given to the right of the phylogeny. Accession numbers found in Additional file 10.

Overall, putative novel Torix hosts detected from all screening methods included taxa from the orders Dermaptera, Gastropoda, Trichoptera, and Trombidiformes. Additionally, new Torix-associated families, genera, and species were identified. These included haematophagous flies (*Simulium aureum, Anopheles plumbeus, Protocalliphora azurea*, Tabanidae), several parasitoid wasp families (e.g., Ceraphronidae, Diapriidae, Mymaridae), forest detritivores (e.g., Sciaridae, Mycetophilidae, Staphylinidae), and phloem-feeding bugs (Psyllidae, Ricaniidae). Feeding habits such as phloem feeding, predation, detritivory, or haematophagy were not correlated with any particular Torix *Rickettsia* subclade (Fig. 6). Furthermore, parasitoid and aquatic lifestyles were seen across the phylogeny. All newly discovered putative Torix *Rickettsia* host taxa are described in Table 3, alongside previously discovered hosts, in order to give an up-to-date overview of Torix-associated taxa.

**Table 3:** Torix *Rickettsia* hosts known to date alongside screening method

Order	Host	Screening method	Reference
Amphipoda	*Paracalliope fluviatilis* (Paracalliopiidae)	GenBank search	This study
	*Paraleptamphopus sp*. (Paraleptamphopidae)	Barcoding	[33]
	Senticaudata sp.	Barcoding	[33]
Araneae	*Amaurobius fenestralis* (Amaurobiidae)	Targeted PCR	This study
	*Amaurobioides africana* (Anyphaenidae)	Barcoding	[32]
	*Araneus diadematus* (Araneidae)	Targeted PCR	[43]
	*Dysdera microdonta* (Dysderidae)	Barcoding	[31]
	*Linyphiidae* spp.	Targeted PCR	[43]
	*Linyphia triangularis* (Linyphiidae)	Targeted PCR	This study
	*Pardosa lugubris* (Lycosidae)	Targeted PCR	This study
	*Pholcus phalangioides* (Pholcidae)	Targeted PCR	This study
	*Pisaura mirabilis* (Pisauridae)	Targeted PCR	This study
	*Metellina mengei* (Tetragnathidae)	Targeted PCR	[43]
Coleoptera	*Deronectes* spp. (Dytiscidae)	Targeted PCR, FISH, and TEM	[24]
	*Dytiscidae* sp.	Barcoding	This study
	*Stegobium paniceum* (Ptinidae)	Non-targeted (16S) PCR	[86]
	*Prionocyphonlimbatus* (Scirtidae)	Barcoding	This study
	*Labidopullus appendiculatus* (Staphylinidae)	SRA search	This study
	*Platyusa sonomae* (Staphylinidae)	SRA search	This study
	*Pseudomimeciton antennatum* (Staphylinidae)	SRA search	This study
	*Staphylinidae* sp.	Barcoding	This study
	*Pimelia* sp. (Tenebrionidae)	GenBank search	This study
Dermaptera	*Forficula* sp. (Forficulidae)	GenBank search	This study
	Unknown sp.	Barcoding	This study
Diplopoda	*Polydesmus complanatus* (Polydesmidae)	Targeted PCR	[87]
	Unknown sp.	Barcoding	This study
Diptera	*Protocalliphora azurea* (Calliphoridae)	Targeted PCR	This study
	*Cecidomyiidae* sp.	Barcoding	This study
	*Chaoborus trivittatus* (Chaoboridae)	SRA search	This study
	*Mochlonyx cinctipes* (Chaoboridae)	SRA search	This study
	*Glyptotendipes* sp. (Chironomidae)	Targeted PCR	This study
	*Zavrelimyia* sp. (Chironomidae)	Targeted PCR	This study
	*Culicoides* spp. (Ceratopogonidae)	Targeted PCR and FISH	[27]
	*Anophelesplumbeus* (Culicidae)	Targeted PCR	This study
	*Dolichopodidae* spp.	Targeted PCR	[44]
	*Empididae* spp.	Targeted PCR	[44]
	*Limonia chorea* (Limoniidae)	N/A	Unpublished (AF322443)
	*Boletinavillosa* (Mycetophilidae)	Barcoding	This study
	Gnoriste bilineata (Mycetophilidae)	SRA search	This study
	*Mycetophilalunata* (Mycetophilidae)	GenBank search	This study
	*Psilidae* sp.	Barcoding	This study
	*Lutzomyia apache* (Psychodidae)	Targeted PCR	[61]
	*Phlebotomus chinensis* (Psychodidae)	Non-targeted (16S) PCR	[60]
	*Sciaridae* sp.	Barcoding	This study
	*Pherbelliatenuipes* (Sciomyzidae)	Barcoding	This study
	*Simuliumaureum* (Simuliidae)	Targeted PCR	This study
	*Tabanidae* sp.	Barcoding	This study
Gastropoda	*Galbatruncatula* (Lymnaeidae)	Targeted PCR	This study
Haplotaxida	*Mesenchytraeus solifugus* (Enchytraediae)	Non-targeted (16S) PCR	[88]
Hemiptera	*Bemisia tabaci* (Aleyrodidae)	Targeted PCR and FISH	[51]
	*Nephotettix cincticeps* (Cicadellidae)	Targeted PCR, FISH, and TEM	[89]
	*Platypleura kaempferi* (Cicadidae)	Non-targeted (16S) PCR	[90]
	*Cimexlectularius* (Cimicidae)	Targeted PCR	This study/[65]
	*Sigarastriata* (Corixidae)	Targeted PCR	This study
	*Metcalfapruinosa* (Flatidae)	GenBank search	This study
	*Flavina* sp. (Issidae)	GenBank search	This study
	*Centrotus cornutus* (Membracidae)	Non-targeted (16S) PCR and TEM	[91]
	*Gargara genistae* (Membracidae)	Non-targeted (16S) PCR and TEM	[91]
	*Macrolophus pygmaeus* (Miridae)	Non-targeted (16S) PCR and FISH	[45]
	*Cacopsyllamelanoneura* (Psyllidae)	Barcoding	This study
	*Chamaepsyllahartigii* (Psyllidae)	Barcoding	This study
	*Ricaniidae* sp.	Barcoding	This study
Hirudinea	*Hemiclepsis* spp. (Glossiphoniidae)	Targeted PCR and TEM	[23]
	*Torix* spp. (Glossiphoniidae)	Targeted PCR and TEM	[23]
Hymenoptera	*Asobara tabida* (Braconidae)	Non-targeted (16S) PCR	[92]
	*Ceraphronidae* sp.	Barcoding	This study
	*Diapriidae* sp.	Barcoding	This study
	*Eucharitidae* sp.	GenBank search	This study
	*Quadrastichus mendeli* (Eulophidae)	Non-targeted (16S) PCR and FISH	[93]
	*Formicidae* sp.	GenBank search	This study
	*Atta colombica* (Formicidae)	Non-targeted (16S) PCR	Unpublished (LN570502)
	*Megaspilidae* sp.	Barcoding	This study
	*Mymaridae* sp.	Barcoding	This study
	*Platygastridae* sp.	Barcoding	This study
Ixodida	*Argas japonica* (Argasidae)	Non-targeted (16S) PCR	[64]
	*Ixodes ricinus* (Ixodidae)	Targeted PCR	[63]
Megaloptera	*Sialis lutaria* (Sialidae)	Targeted PCR	[94]
Neuroptera	*Chrysotropia ciliata* (Chrysopidae)	Targeted PCR	[94]
Nucleariida	*Nuclearia pattersoni* (Nucleariidae)	Non-targeted (16S) PCR	[25]
	*Pompholyxophrys punicea* (Pompholyxophryidae)	Single-cell sequencing	[26]
Odonata	*Calopteryxmaculata* (Calopterygidae)	GenBank search	This study
	*Coenagrionidae* spp.	Targeted PCR and FISH	[28]
	*Sympetrum fonscolombii* (Libellulidae)	Targeted PCR	[28]
	*Polythoridae* spp.	Targeted PCR	[28]
	*Neoneura sylvatica* (Protoneuridae)	Targeted PCR	[28]
Psocoptera	*Myopsocidae* sp.	Barcoding	This study
	*Philotarsuscalifornicus* (Philotarsidae)	Barcoding	This study
	*Cerobasis guestfalica* (Trogiidae)	Targeted PCR and FISH	[95]
Siphonaptera	*Nosopsyllus fasciatus* (Ceratophyllidae)	Targeted PCR	[62]
Trichoptera	*Lepidostomahoodi* (Lepidostomatidae)	Barcoding	This study
	*Rhyacophiladorsalis* (Rhyacophilidae)	Targeted PCR	This study
	*Sericostoma* sp. (Sericostomatidae)	SRA search	This study
Trombidiformes	*Calyptostomatidae* sp.	Barcoding	This study

FISH: fluoresence *in situ* hybridization; SRA: sequence read archive; TEM: transmission electron microscopy. Accession numbers for *Rickettsia* sequences from newly detected hosts can be found in Additional files 8 and 10.

## Discussion

Symbiotic interactions between hosts and microbes are important drivers of host phenotype, with symbionts both contributing to, and degrading, host performance. Heritable microbes are particularly important contributors to arthropod biology, with marked attention focused on *Wolbachia*, the most common associate [9]. Members of the Rickettsiales, like *Wolbachia*, share an evolutionary history with mitochondria [42], such that a previous screen of BOLD submissions of mtDNA submissions observed *Wolbachia* as the main bacterial contaminant associated with DNA barcoding [34]. However, our screen found that *Rickettsia* amplicons were more commonly found in BOLD deposits compared to *Wolbachia* (0.41% vs 0.17% of deposits). Furthermore, Torix group *Rickettsia* were overrepresented in barcode misamplifications (95%) when compared to other groups within the genus. A comparison of the most commonly used barcoding primers to *Wolbachia* and *Rickettsia* genomes suggest that homology of the forward primer 3′ end was likely responsible for this bias towards Torix *Rickettsia* amplification. To gain a clearer understanding of the relative balance of Torix group to other *Rickettsia* within symbioses and habitats, a targeted screen and bioinformatic approach was also undertaken. Through these 3 screens, a broad range of host diversity associated with Torix *Rickettsia* was uncovered.

As the *in silico* and empirical evidence suggests that *Rickettsia COI* amplification is not uncommon [31–33], why has this phenomenon not been described more widely before? The previous large-scale non-target *COI* study using BOLD submissions [34] revealed only *Wolbachia* hits. This screen involved comparison to a *Wolbachia*-specific reference library and was thus likely to miss *Rickettsia*. Additionally, there has been a lack of Torix *Rickettsia COI* homologues to compare barcodes to until recently, when a multilocus identification system including *COI* was devised [27]. Indeed, out of the non-target *COI* dataset received in this study, some of the *Rickettsia* contaminants were tentatively described by BOLD as *Wolbachia* owing to the previous absence of publicly available *Rickettsia COI* to compare.

Although *Rickettsia* will only interfere with barcoding in a minority of cases (∼0.4%), it is likely that alternate screening primers for some studies will need to be considered. In a demonstration of how unintended *Rickettsia* amplifications can affect phylogeographic studies relying on DNA barcoding, a *Rickettsia COI* was conflated with the mtDNA *COI* of a species of freshwater amphipod, *Paracalliope fluvitalis* [30]. Subsequently, supposed unique mtDNA haplotypes were allocated to a particular collection site, whereas this merely demonstrated the presence of Torix *Rickettsia* in host individuals in this lake. Contrastingly, non-target *Rickettsia* amplification can also allow for the elucidation of a novel host range of the symbiont [31–33], and this has been exemplified with our probing of BOLD.

Previously, several host orders have been associated with Torix *Rickettsia*, including Araneae, Coleoptera, Diptera, Hemiptera, and Odonata [24, 28, 43–45]. Newly uncovered putative host orders from this study include Dermaptera, Gastropoda, Trichoptera, and Trombidiformes (Table 2). These data emphasize the broad host range of Torix *Rickettsia* across arthropods and invertebrates, with 2 additional cases from nucleariid amoebae [25, 26]. This host range is complementary to *Rickettsia*’s sister genus “*Candidatus* Megaira” (formerly the Hydra group of *Rickettsia*), which are present in multiple unicellular eukaryote families and in a few invertebrates like *Hydra* [46].

Despite the extensive sampling and multiple screening strategies used in this project, caution must be taken when interpreting to what extent the Torix *Rickettsia* hosts identified are representative of *Rickettsia* hosts in nature. Both BOLD and SRA components of the project rely on secondary data, which come with sampling and methodological biases. For example, most SRA submissions are from laboratory-reared terrestrial insects and it can be argued that the high number of Belli *Rickettsia* infections discovered from arthropod genome projects (compared to the targeted screen, which contains multiple aquatic insect species) could be due to this sampling bias. Likewise, the overrepresentation of Torix *Rickettsia* from BOLD is likely due to an amplification bias as a result of higher primer site homology to that particular group from commonly used barcoding primer sets. Subsequently, the common patterns of infection (or “hot spots”) found in this study are identified as such with these provisos in mind. To counteract these biases and to give a more nuanced and holistic view of Torix *Rickettsia* ecology, a targeted screen was also included to ensure that this study was not overreliant on secondary data.

Further caution needs to be taken when interpreting what these newly found associations mean because the mere presence of *Rickettsia* DNA does not definitively indicate an endosymbiotic association. For example, bacterial DNA integrations into the host nuclear genome have been widely reported [47]. Although none of the protein-coding genes sequenced in this study showed signs of a frameshift, suggesting a lack of pseudogenization that is often typical of a nuclear insertion, this still does not rule out this phenomenon entirely. Furthermore, parasitism or ingestion of symbiont-infected biota (e.g., protists) could also result in bacteria detection [48–50]. Whilst protist reads were found in some datasets, these were usually at a much lower depth compared to the symbiont [39]. In one of the few instances where protist reads were greater than *Rickettsia* (Dataset SRR5298327), this was from our own previous study where a true endosymbiosis between insect and symbiont was confirmed through FISH imaging [27]. Similarly, although an 18S sequence aligned to a parasitoid wasp was observed in the SRA dataset from *Bemisia tabaci* (SRR6313831), previous work has also demonstrated a true endosymbiosis between *B. tabaci* and Torix *Rickettsia* [51]. Overall, these data suggest that detecting contamination from *Rickettsia*-infected taxa such as protists and parasitoid wasps is uncommon within our study.

Model-based estimation techniques suggest that *Rickettsia* are present in 20–42% of terrestrial arthropod species [12]. However, the targeted PCR screen in this study gave an estimated species prevalence of 8.9% for terrestrial species. This discrepancy is likely due to targeted screens often underestimating the incidence of symbiont hosts owing to various methodological biases including small within-species sample sizes (missing low-prevalence infections) [29]. Importantly, the inclusion and exclusion of specific ecological niches can also lead to a skewed view of *Rickettsia* symbioses. A previous review of *Rickettsia* bacterial and host diversity by Weinert et al. [13] suggested a possible (true) bias towards aquatic taxa in the Torix group. In accordance with this, our targeted screen demonstrated that Torix *Rickettsia* infections were more prevalent in aquatic insect species compared to terrestrial (although this is likely not the case for invertebrates in general owing to a Torix *Rickettsia* hot spot in spiders). The observed overrepresentation of Torix group *Rickettsia* (17 of 19 strains) in our targeted screen contrasts with the findings of Weinert et al., which show a predominance of Belli infections, and is likely due to the latter study's near absence of aquatic insects and spiders within the samples screened. Our additional use of a bioinformatics approach based on the SRA appears to corroborate targeted screen data, indicating that Belli and Torix are 2 of the most common *Rickettsia* groups among arthropods. Overall, these multiple screening methods suggest that Torix *Rickettsia* are more widespread than previously thought and their biological significance underestimated.

Previous studies have used either 1 or 2 markers to identify the relatedness of strains found in distinct hosts. In this study, we use the multilocus approach developed in Pilgrim et al. [27] to understand the affiliation of Torix *Rickettsia* from diverse invertebrate hosts. Our analysis of Torix strains indicates that closely related strains are found in distantly related taxa. Closely related *Rickettsia* are also found in putative hosts from different niches and habitats—for instance, the *Rickettsia* strains found in terrestrial blood feeders do not lie in a single clade but rather are allied to strains found in non-blood-feeding host species. Likewise, strains in phloem-feeding insects are diverse rather than commonly shared.

The distribution of Torix *Rickettsia* across a broad host range suggests that host shifts are occurring between distantly related taxa. It is notable that parasitoid wasps are commonly infected with *Rickettsia* and have been associated with enabling symbiont host shifts [48]. Aside from endoparasitoids, it is also possible that plant feeding can allow for endosymbiont horizontal transmission [52, 53]. For example, *Rickettsia* horizontal transmission has been demonstrated in *Bemisia* whiteflies infected by phloem feeding [52, 54]. Finally, ectoparasites like the Torix-infected water mites of the Calyptostomatidae family could also play a role in establishing novel *Rickettsia*-host associations, as feeding by mites has been observed to lead to host shifts for other endosymbiont taxa [55]. Indeed, if multiple horizontal transmission paths do exist, this could account for the diverse plethora of infected taxa, as well as arthropods identified in this study that harbour >1 strain of symbiont [56].

The finding that Torix *Rickettsia* are associated with a broad range of invertebrates leads to an obvious question: what is the impact and importance of these symbiotic associations? Previous work has established that Torix *Rickettsia* represent heritable symbionts, and it is likely that this is true generally. There have, however, been few studies on their impact on the host. In the earliest studies [22, 23], *Torix* spp. leeches infected with *Rickettsia* were observed to be substantially larger than their uninfected counterparts. Since then, the only observation of note, pertaining to the Torix group, is the reduced ballooning (dispersal) behaviour observed in infected *Erigone atra* money spiders [57]. Overall, the incongruencies in host and Torix *Rickettsia* phylogenies (suggesting a lack of co-speciation and obligate mutualism), along with the lack of observed sex bias in carrying the symbiont, indicate that facultative benefits are the most likely symbiotic relationship [29]. However, *Rickettsia* induction of thelytokous parthenogenesis (observed in Belli *Rickettsia* [58, 59]) should not be discounted in Torix-infected parasitoid wasps identified in this study. To add to the challenge of understanding Torix *Rickettsia* symbioses, the challenges of laboratory rearing of many Torix *Rickettsia* hosts have led to difficulties in identifying model systems to work with. However, the large expansion of our Torix group host knowledge can now allow for a focus on cultivable hosts (e.g., phloem-feeding bugs).

To conclude, we have shown that large-scale DNA barcoding initiatives of arthropods can include non-target amplification of Torix *Rickettsia*. By examining these non-target sequences, alongside a targeted screen and SRA search, we have uncovered numerous previously undetected putative host associations. Our findings lay bare multiple new avenues of inquiry for Torix *Rickettsia* symbioses.

## Potential Implications

A particularly important group for future study of Torix *Rickettsia* interactions are haematophagous host species. Our discovery of *Rickettsia*-associated tabanid and simulid flies, alongside *Anopheles plumbeus* mosquitoes, adds to existing blood feeders previously identified as Torix group hosts, which include sand flies [60, 61], fleas [62], ticks [63, 64], bedbugs [65], and biting midges [27]. Some *Rickettsia* strains are known to be transmitted to vertebrates via haematophagy [66]. However, there is no evidence to date for vertebrate pathogenic potential for the Torix group. Despite this, Torix *Rickettsia* could still play a significant role in the ecology of vectors of disease. A key avenue of research is whether these endosymbionts alter vectorial capacity, as found for other associations [67]. In contrast to the widely reported virus-blocking phenotype observed in *Wolbachia*-infected vectors [68, 69], Torix *Rickettsia* has recently been associated with a virus-potentiating effect in *Bemisia* whiteflies vectoring Tomato yellow leaf curl virus [70]. Additionally, we uncovered a *Rickettsia*-infected psyllid (*Cacopsylla melanoneura*), which is a vector of *Phytoplasma mali* (apple proliferation) [71]. Thus, the question of Torix *Rickettsia* vector-competence effects is clearly of widespread relevance and deserves further attention.

## Methods

### Interrogation of BOLD

#### Assessment of non-target microbe amplicons

BOLD data curation involves identifying non-target *COI* sequences from common contaminants (e.g., human and bacteria) or erroneous morphological identifications [38]. The designation of bacterial contaminants by BOLD, from a dataset containing 3,817 non-target sequences [36], was confirmed by the taxonomic classification program, Kaiju, using default parameters [72]. Sequences were then placed phylogenetically to refine taxonomy further. To this end, barcodes confirmed as microbial sequences were aligned using the “L-INS-I” algorithm in MAFFT v7.4 (MAFFT, RRID:SCR_011811) [73]. Gblocks (Gblocks, RRID:SCR_015945) [74] was then used to exclude areas of the alignment with excessive gaps or poor alignment using “options for a less stringent selection”; the inclusion of some missing data in alignments was allowed because missing characters does not often affect phylogenetic resolution for taxa with complete data [75]. ModelFinder [76] then determined the TIM3+F+I+G4 model to be used after selection based on default “auto” parameters using the Bayesian information criteria. A maximum likelihood (ML) phylogeny was then estimated with IQTree [77] using an alignment of 561 nucleotides and 1,000 ultrafast bootstraps [78]. The Rickettsiales genera *Anaplasma, Rickettsia, Orientia*, and *Wolbachia* (Supergroups A, B, E, and F), as well as the Legionellales genera *Legionella* and *Rickettsiella*, were included in the analysis as references (as suggested by Kaiju). Finally, both phylogram and cladogram trees (the latter for ease of presentation) were drawn and annotated on the basis of host taxa (order) using the EvolView [79] online tree annotation and visualization tools. Subsequent phylogenetic workflows detailed below follow this method with the exception being the chosen models by Modelfinder.

A determining factor for non-target amplification of bacteria is primer site matching to microbial associates. Subsequently, pairwise homology of the primer set predominantly used for BOLD barcode screening was compared to *Rickettsia* and *Wolbachia COI* genes.

#### Further phylogenetic analysis

*COI* sequence alone provides an impression of the frequency with which *Rickettsia* associates are found in barcoding studies. However, they have limited value in describing the diversity of the *Rickettsia* found. To provide further insight into the diversity of *Rickettsia* using a multilocus approach, we obtained 186 DNA extracts from the archive at the Centre for Biodiversity Genomics (University of Guelph, Canada) that had provided *Rickettsia* amplicons in the previous screen. DNA extracts were chosen on the basis of assorted geographic location, host order, and phylogenetic placement. Multilocus PCR screening and phylogenetic analysis of *Rickettsia* was then completed, using the methodology in Pilgrim et al. [27], which used primers conserved across all known clades of the *Rickettsia* genus [27]. However, slight variations include the exclusion of the *atpA* gene due to observed recombination at this locus. Furthermore, the amplification conditions for the *17KDa* locus were changed because a Torix *Rickettsia* reference DNA extract (host: *Simulium aureum*) failed to amplify with the primer set Ri_17KD_F/Ri_17KD_R from Pilgrim et al. [27]. Subsequently, a *17KDa* alignment from genomes spanning the Spotted Fever, Typhus, Transitional, Belli, and Limoniae groups and the genus “*Candidatus* Megaira” was generated to design a new set of primers using the online tool PriFi [80].

Once multilocus profiles of the *Rickettsia* had been established, we tested for recombination within and between loci using RDP v4 (Recombination Detection Program, RRID:SCR_018537) [81] using the MaxChi, RDP, Chimaera, Bootscan, and GENECONV algorithms with the following criteria to assess a true recombination positive: a *P*-value of <0.001;  sequences were considered linear with 1,000 permutations being performed. Samples amplifying ≥3 of 4 genes (*16S rRNA, 17KDa*,  *COI, *and *gltA*) were then concatenated and their relatedness estimated using ML as described above. The selected models used in the concatenated partition scheme [82] were as follows: *16S rRNA*: TIM3+F+R2; *17KDa*: GTR+F+I+G4; *COI:* TVM+F+I+G4; *gltA:* TVM+F+I+G4. Accession numbers for all sequences used in phylogenetic analyses can be found in Additional file 10.

#### Re-barcoding Rickettsia-containing BOLD DNA extracts

Aside from phylogenetic placement of these *Rickettsia*-containing samples, attempts were made to extract an mtDNA barcode from these taxa in order to identify the hosts of infected specimens. This is because morphological taxonomic classification of specimens in BOLD is usually only down to the order level before barcoding takes place. Previous non-target amplification of *Rickettsia* through DNA barcoding of arthropod DNA extracts had occurred in the bedbug *Cimex lectularius*, with a recovery of the true barcode after using the primer set C1‐J‐1718/HCO1490, which amplifies a shortened 455-bp sequence within the *COI* locus. Subsequently, all samples were screened using these primers or a further set of secondary *COI* primers (LCOt_1490/MLepR1 and LepF1/C_ANTMR1D) if the first failed to give an adequate host barcode. All *COI* and *Rickettsia* multilocus screening primer details, including references, are available in Additional file 11.

Cycling conditions for *COI* PCRs were as follows: initial denaturation at 95°C for 5 min, followed by 35 cycles of denaturation (94°C, 30 sec), annealing (50°C, 60 sec), extension (72°C, 90 sec), and a final extension at 72°C for 7 min. *Rickettsia* and host amplicons identified by gel electrophoresis were subsequently purified enzymatically (ExoSAP) and Sanger sequenced through both strands using a BigDye® Terminator v3.1 kit (Thermo Scientific, Waltham, MA, USA), and capillary sequenced on a 3500 xL Genetic Analyser (Applied Biosystems, Austin, TX, USA). Forward and reverse reads were assessed in UGENE (UGENE, RRID:SCR_005579) [83] to create a consensus sequence by eye with a cut-off phred (Q) score [84] of 20. Primer regions were trimmed from barcodes before being matched to the GenBank database by BLAST based on default parameters and an e-value threshold of <1e−85. Host taxonomy was determined by a barcode-based assignment of the closest BLAST hit, under the following criteria modified from Ramage et al. [50]:

1. Species-level designation for ≥98% sequence identity.
2. Genus-level designation for ≥95% sequence identity.
3. Family-level designation for ≥85% sequence identity.

Additionally, all sequences were required to be ≥200 bp in length.

#### Assessment of barcoding success

One of the factors determining a successful *COI* bacterial amplification is the initial failure of an extract to amplify mtDNA. Subsequently, to determine the likelihood of this event within taxa, we used the 55,366-specimen representative data subset [37] to evaluate failure rates. To this end, all orders of host that gave ≥1 non-target *Rickettsia COI* hit were assessed. The barcoding success rate was determined as the proportion of specimens that matched initial morphotaxa assignment and were not removed after BOLD quality control [38]. Because the total *Rickettsia* count was from a larger dataset than the one made available, an adjusted infection frequency for each taxon was calculated on the basis of the representative data subset.

### Targeted and bioinformatic *Rickettsia* screens

#### Targeted screen of aquatic and terrestrial arthropods

Overall, 1,612 individuals from 169 species, including both terrestrial (DNA extracts derived from European material, mostly from Duron et al. [11]) and aquatic invertebrates (largely acquired from the UK between 2016 and 2018), were screened. Amplification of mtDNA *COI* was conducted as a control for DNA quality. Some arthropods that could not be identified down to the species level morphologically or from barcoding were referred to as “sp.” To investigate symbiont infection status, rickettsial-specific primers based on *gltA* and *16S rRNA* genes were used for conventional PCR screening [27], with Sanger sequences obtained from ≥1 specimen per *Rickettsia*-positive species to identify any misamplification false-positive results. Newly identified hosts of interest from BOLD and targeted screens were then placed phylogenetically (see sections above) with the models TIM3+F+R2 (16S) and K3Pu+F+G4 (gltA) before being mapped by lifestyle and diet.

It is known that there are taxonomic hot spots for endosymbiont infection, with, e.g., spiders being a hot spot for a range of microbial symbionts [43]. Therefore, analyses were performed that were matched at a taxonomic level (i.e., each taxon was represented in both the aquatic and terrestrial pools). To this end, the incidence of Torix *Rickettsia* was first compared in all insects. However, within insects, there is taxon heterogeneity between aquatic and terrestrial biomes (e.g., Ephemeroptera, Plecoptera in aquatic only, Lepidoptera in terrestrial only). The analysis was therefore narrowed to match insect orders present in both the aquatic and terrestrial community. Three insect orders, Hemiptera, Diptera, and Coleoptera, fulfilled this criterion with good representation from each biome. For each case, the ratios of the infected to non-infected species between aquatic and terrestrial communities were compared in a Fisher exact test with a *P*-value significance level of ≤0.05.

#### Search of the SRA and GenBank

The SRA dataset [39] containing 1 individual from 1,341 arthropod species was screened with phyloFlash [40] using default parameters, which finds, extracts, and identifies SSU rRNA sequences. Reconstructed full *16S rRNA* sequences affiliated to *Rickettsia* were extracted and compared to sequences derived from the targeted screen phylogenetically (see sections above) to assess group representation within the genus. The microbial composition of all SRA datasets that did not result in a reconstructed *Rickettsia 16S rRNA* with phyloFlash were re-evaluated using Kraken2 [85], a *k*-mer–based taxonomic classifier for short DNA sequences. A cut-off of ≥40,000 reads assigned to *Rickettsia* taxa was applied for reporting potential infections (theoretical genome coverage of ∼1–4× assuming an average genome size of ∼1.5 Mb). As *Rickettsia*-infected protists and parasitoids have previously been reported [25, 26, 59], phyloFlash was also used to identify reads aligned to these taxa to account for potential positive results attributed to ingested protists or parasitisms.

We also examined GenBank for *Rickettsia* sequences deposited as invertebrate *COI* barcodes. To this end, a BLAST search of Torix *Rickettsia COI* sequences from previous studies [27, 32] was conducted on 29 June 2020. Sequences were putatively considered to belong to the Torix group if their similarity was >90% and subsequently confirmed phylogenetically as described above with the HKY+F+G4 model.

## Data Availability

The datasets supporting the findings of this study are openly available in the BOLD repository [37] and the Figshare repository [36, 39]. Alignments and trees are also available from the *GigaScience* GigaDB repository [96]. For DNA sequences, accessions are Bioproject No. PRJEB38316; Accession Nos. LR798809-LR800243, LR812141-LR812260, LR812269-LR812283, LR812678, LR813674-LR813676, LR813730.

## Additional Files

**Additional file 1**. Taxonomic classification of BOLD non-target *COI* sequences via Kaiju.

**Additional file 2**.Rectangular phylogram trees of cladograms from Figs 2 and 3.

**Additional file 3**. Primer pairs involved in the unintended amplification of 753 *Rickettsia COI* from BOLD project.

**Additional file 4**. Homology of *Rickettsia* groups and *Wolbachia* to the most common forward primers (C_LepFolF and C_LepFolR) attributed to bacterial *COI* amplification from arthropod DNA extracts.

**Additional file 5**. Re-barcoding status and nearest BLAST hit of mtDNA *COI* arthropod DNA extracts accessed for further analysis, along with the success of multilocus *Rickettsia* profiles with allocated *Rickettsia* group (based on phylogenetic analysis) and co-infection status.

**Additional file 6**. The barcoding success rate of taxa that gave ≥1 bacteria *COI* inadvertent amplification (N = 51,475 accessible specimens) with an adjusted *Rickettsia* frequency based on an estimated total number of arthropods to account for inaccessible specimens (N = 125,402).

**Additional file 7**. Fisher exact test analyses for comparison of Torix *Rickettsia* infection in aquatic vs terrestrial insects.

**Additional file 8**. GenBank matches mistaken for true mtDNA barcodes and their homology to *Rickettsia COI* (accessed 29 June 2020).

**Additional file 9**. Phylogram of a maximum likelihood (ML) tree of *COI Rickettsia* found in the GenBank database erroneously identified as mtDNA barcodes based on 577 bp. The HKY+F+G4 model was chosen as the best-fitting model using Modelfinder with the Bayesian information criterion (BIC).

**Additional file 10**. Accession numbers used for phylogenetic analyses (Figs 2– 6). Accession numbers generated in this study are boldface.

**Additional file 11**. Mitochondrial *COI* and bacterial gene primers used for re-barcoding and multilocus phylogenetic analyses.

## Abbreviations

BLAST: Basic Local Alignment Search Tool; BOLD: Barcode of Life Data System; bp: base pairs; COI: cytochrome c oxidase I; FISH: fluorescence *in situ* hybridization; MAFFT: Multiple Alignment using Fast Fourier Transform; ML: maximum likelihood; mtDNA: mitochondrial DNA; rRNA: ribosomal RNA; SRA: Sequence Read Archive; SSU: single-subunit.

## Competing Interests

The authors declare that they have no competing interests.

## Funding

This work was supported by a BBSRC Doctoral Training Partnership studentship (BB/M011186/1) awarded to J.P.; a Development and Promotion of Science and Technology Talents Project (DPST) of the Institute for the Promotion of Teaching Science and Technology, Thailand to P.T.; and a Harry Smith Vacation studentship (Microbiology Society) and a NERC ACCE DTP studentship (NE/L002450/1) to H.R.D.

## Authors' Contributions

J.P., G.D.D.H., M.B., and M.A.S.: conception and design of the study. M.A.S., E.V.Z., S.R., and J.R.D.: assembling BOLD datasets and providing DNA extracts for laboratory experiments. J.P., C.R.M., and P.T.: field and laboratory work. H.R.D. and S.S.: SRA work. J.P., P.T., H.R.D., G.D.D.H., M.B., and S.S.: Analyses and interpretation of the data, drafting of the manuscript. All authors assisted in critical revision of the manuscript.

## Supplementary Material

giab021_GIGA-D-20-00244_Original_Submission

giab021_GIGA-D-20-00244_Revision_1

giab021_GIGA-D-20-00244_Revision_2

giab021_Response_to_Reviewer_Comments_Original_Submission

giab021_Response_to_Reviewer_Comments_Revision_1

giab021_Reviewer_1_Report_Original_Submission Steve Perlman -- 8/31/2020 Reviewed

giab021_Reviewer_2_Report_Original_Submission Xin Zhou -- 9/6/2020 Reviewed

giab021_Supplemental_Files
