# Supplementary material for: Torix Rickettsia are widespread in arthropods and reflect a neglected symbiosis
Source: Gigascience. 2021 Mar 25;10(3):giab021. doi: 10.1093/gigascience/giab021 (PMC7992394; doi:10.1093/gigascience/giab021)
Supplement: giab021_GIGA-D-20-00244_Original_Submission [file giab021_giga-d-20-00244_original_submission.pdf]

GigaScience

Torix Rickettsia are widespread in arthropods and reflect a neglected symbiosis

--Manuscript Draft--

**Manuscript Number:**	GIGA-D-20-00244
**Full Title:**	Torix Rickettsia are widespread in arthropods and reflect a neglected symbiosis
**Article Type:**	Research
**Funding Information:**	Biotechnology and Biological Sciences Research Council (BB/M011186/1) — Dr. Jack Pilgrim
**Abstract:**	Abstract<br>Background: Rickettsia are intracellular bacteria best known as the causative agents of human and animal diseases. Although these medically important Rickettsia are often transmitted via haematophagous arthropods, other Rickettsia , such as those in the Torix group, appear to reside exclusively in invertebrates and protists with no secondary vertebrate host. Importantly, little is known about the diversity or host range of Torix group Rickettsia .<br>Results: This study describes the serendipitous discovery of Rickettsia amplicons in the Barcode of Life Data System (BOLD), a sequence database specifically designed for the curation of mtDNA barcodes. Out of 184,585 barcode sequences analysed, Rickettsia is observed in approximately 0.41% of barcode submissions and is more likely to be found than Wolbachia (0.17%). The Torix group of Rickettsia are shown to account for 95% of all unintended amplifications from the genus, with a multilocus analysis of these strains revealing this symbiont commonly shifts between distantly related host taxa. A further targeted PCR screen of 1,612 individuals from 169 terrestrial and aquatic arthropod species identified mostly Torix strains and supports the “aquatic hotspot” hypothesis for Torix infection. Furthermore, the analysis of 60,409 Sequence Read Archive (SRA) deposits indicates Torix infections represent a significant proportion of all Rickettsia symbioses.<br>Conclusions: This combination of methods reveals a broad host diversity associated with Torix Rickettsia including phloem-feeding bugs, parasitoid wasps, forest detritivores and vectors of disease. The unknown host effects and transmission strategies of these endosymbionts make these newly discovered associations important to inform future directions of investigation involving the understudied Torix Rickettsia .
**Corresponding Author:**	Jack Pilgrim<br>University of Liverpool Institute of Infection Veterinary and Ecological Sciences<br>Neston, Cheshire, Liverpool UNITED KINGDOM
**Corresponding Author Secondary Information:**	
**Corresponding Author's Institution:**	University of Liverpool Institute of Infection Veterinary and Ecological Sciences
**Corresponding Author's Secondary Institution:**	
**First Author:**	Jack Pilgrim
**First Author Secondary Information:**	
**Order of Authors:**	Jack Pilgrim
	Panupong Thongprem
	Helen R. Davison
	Stefanos Siozios
	Matthew Baylis
	Evgeny V. Zakharov
	Sujeevan Ratnasingham

Powered by Editorial Manager® and ProduXion Manager® from Aries Systems Corporation

	Jeremy R. deWaard
	M. Alex Smith
	Gregory D. D. Hurst
**Order of Authors Secondary Information:**	
**Additional Information:**	
**Question**	**Response**
Are you submitting this manuscript to a special series or article collection?	No
**Experimental design and statistics**<br><br>Full details of the experimental design and statistical methods used should be given in the Methods section, as detailed in our Minimum Standards Reporting Checklist. Information essential to interpreting the data presented should be made available in the figure legends.<br><br>Have you included all the information requested in your manuscript?	Yes
**Resources**<br><br>A description of all resources used, including antibodies, cell lines, animals and software tools, with enough information to allow them to be uniquely identified, should be included in the Methods section. Authors are strongly encouraged to cite Research Resource Identifiers (RRIDs) for antibodies, model organisms and tools, where possible.<br><br>Have you included the information requested as detailed in our Minimum Standards Reporting Checklist?	Yes
**Availability of data and materials**<br><br>All datasets and code on which the conclusions of the paper rely must be either included in your submission or deposited in publicly available repositories (where available and ethically	Yes

appropriate), referencing such data using a unique identifier in the references and in the "Availability of Data and Materials" section of your manuscript.<br><br>Have you have met the above requirement as detailed in our Minimum Standards Reporting Checklist?	

Manuscript
Click here to access/download;Manuscript;Torix Rickettsia are widespread in arthropods 15 8 20.docx

Torix *Rickettsia* are widespread in arthropods and reflect a neglected symbiosis

Jack Pilgrim[1*], Panupong Thongprem[1], Helen R. Davison[1], Stefanos Siozios[1], Matthew Baylis[1,2], Evgeny V. Zakharov[3], Sujeevan Ratnasingham[3], Jeremy R. deWaard[3], M. Alex Smith[3], Gregory D. D. Hurst[1]

1. Institute of Infection, Veterinary and Ecological Sciences, Faculty of Health and Life Sciences, University of Liverpool, Liverpool, U.K.

2. Health Protection Research Unit in Emerging and Zoonotic Infections, Liverpool, U.K.

3. Centre for Biodiversity Genomics, University of Guelph, Guelph, Ontario, Canada.

4. Department of Integrative Biology, University of Guelph, Guelph, Ontario, Canada.

* Corresponding author

Emails:

JP: jack.pilgrim@liverpool.ac.uk

PT: pongthq@liverpool.ac.uk

HRD: hlhdavi5@liverpool.ac.uk

SS: sioziosss@liverpool.ac.uk

MB: baylism@liverpool.ac.uk

EVZ: zakharov@uoguelph.ca

SR: sratnasi@uoguelph.ca

JRD: dewaardj@uoguelph.ca

MAS: salex@uoguelph.ca

GDDH: ghurst@liverpool.ac.uk

Abstract

Background: *Rickettsia* are intracellular bacteria best known as the causative agents of human and animal diseases. Although these medically important *Rickettsia* are often transmitted via haematophagous arthropods, other *Rickettsia*, such as those in the Torix group, appear to reside exclusively in invertebrates and protists with no secondary vertebrate host. Importantly, little is known about the diversity or host range of Torix group *Rickettsia*.

Results: This study describes the serendipitous discovery of *Rickettsia* amplicons in the Barcode of Life Data System (BOLD), a sequence database specifically designed for the curation of mtDNA barcodes. Out of 184,585 barcode sequences analysed, *Rickettsia* is observed in approximately 0.41% of barcode submissions and is more likely to be found than *Wolbachia* (0.17%). The Torix group of *Rickettsia* are shown to account for 95% of all unintended amplifications from the genus, with a multilocus analysis of these strains revealing this symbiont commonly shifts between distantly related host taxa. A further targeted PCR screen of 1,612 individuals from 169 terrestrial and aquatic arthropod species identified mostly Torix strains and supports the “aquatic hotspot” hypothesis for Torix infection. Furthermore, the analysis of 60,409 Sequence Read Archive (SRA) deposits indicates Torix infections represent a significant proportion of all *Rickettsia* symbioses.

Conclusions: This combination of methods reveals a broad host diversity associated with Torix *Rickettsia* including phloem-feeding bugs, parasitoid wasps, forest detritivores and vectors of disease. The unknown host effects and transmission strategies of these endosymbionts make these newly discovered associations important to inform future directions of investigation involving the understudied Torix *Rickettsia*.

Keywords: Rickettsia; symbiosis: arthropods; endosymbiont; DNA barcoding

Background

It is now widely recognized that animals live in a microbial world, and that many aspects of animal biology, ecology and evolution are a product of their symbioses with microorganisms [1]. In invertebrates, these symbioses may be particularly intimate, and involve transmission of the microbe from parent to offspring [2]. The alignment of host reproduction with symbiont transmission produces a correlation between the fitness interests of the parties, reflected in symbionts evolving to play a number of physiological roles within the host, from defence [3,4] through to core anabolic and digestive functions [5,6]. However, the maternal inheritance of these microbes has led to the retention of parasitic phenotypes associated with distortion of reproduction, with symbiont phenotypes including biases towards daughter production and cytoplasmic incompatibility [7]. These diverse individual impacts alter the ecology and evolution of the host, in terms of diet, dynamics of interaction with natural enemies, sexual selection and speciation.

Heritable symbioses have evolved on multiple occasions amongst microbial taxa. In some cases, the microbial lineage is limited to a single clade of related animal hosts, such as *Buchnera* in aphids [8]. In other cases, particular heritable microbes are found across a wide range of arthropod species. *Wolbachia* represents the most common associate, considered to infect nearly half of all species [9], and this commonness is a function in part of the ability of *Wolbachia* to transfer to a broad range of new host species and spread within them (host shift events) [10]. Aside *Wolbachia*, other microbes are found commonly as heritable symbionts of arthropod hosts [11]. *Cardinium* and *Rickettsia*, for instance, have been estimated at being present in 13-55% and 20-42% of species respectively [12].

In this paper, we address the diversity and commonness of symbioses between *Rickettsia* and arthropods. The *Rickettsia* have increasingly been recognized as a genus of bacteria with diverse interactions with arthropods [13,14]. First discovered as the agents underlying several diseases of humans vectored by haematophagous arthropods [15,16], our understanding of the group changed in the 1990s with the recognition that *Rickettsia* were commonly arthropod symbionts [17,18]. *Rickettsia* were recognized first as male-killing reproductive parasites [17,19] and then later as beneficial partners [3,20,21].

Following this extension of our understanding of *Rickettsia*-arthropod interactions, a new clade of *Rickettsia* was discovered from work in *Torix* leeches [22,23]. This clade was sister to all other *Rickettsia* genera, with no evidence to date of any strain having a vertebrate pathogen phase. The host range for Torix *Rickettsia* is broader than that for other members of the genus, going beyond arthropods to include amoeba hosts [24,25]. Targeted PCR based screening have revealed Torix group *Rickettsia* as particularly common in three groups with aquatic association: *Culicoides* biting midges, deronectid beetles and odonates [26–28]. However, some previous hypothesis-free PCR screens that aimed to detect *Rickettsia* in arthropods have likely missed these symbioses, due to divergence of the marker sequence and mismatch with the primers [29].

During our previous work on Torix *Rickettsia* in biting midges [26], we became aware of the presence of *Rickettsia CoxA* sequences deposited in GenBank that derived from studies where the intended target of amplification/sequencing was cytochrome *c* oxidase I (*COI*), the

mitochondrial equivalent of *CoxA*. These deposits derived from studies using mtDNA barcoding for phylogeographic inference [30], or in barcoding based species identification approaches [31,32]. Non-target amplification of *Rickettsia COI* using mitochondrial *COI* barcoding primers has been reported in spiders [31,32] and freshwater amphipods [30,33]. Furthermore, we have noted two cases in our lab where amplicons obtained for mtDNA barcoding of an arthropod have, on sequence analysis, revealed *Rickettsia COI* amplification (Belli group *Rickettsia* from Collembola, and Torix group *Rickettsia* from *Cimex lectularius* bedbugs). Previous work had established barcoding approaches may amplify *COI* from *Wolbachia* symbionts [34], and the data above indicated that non-target *Rickettsia COI* may be likewise amplified during this PCR amplification for mitochondrial *COI*.

In this paper, we use three approaches to reveal the diversity and commonness of Torix *Rickettsia* in arthropods. First, we probed the contaminant bin of the Barcode of Life Data System (BOLD [35]) for *Rickettsia* sequences and used the template from these projects to define the diversity of *Rickettsia* observed using a multilocus approach. Second, we screened DNA templates from multiple individuals from 169 arthropod species for *Rickettsia* presence using PCR assays that function more broadly than previously utilized in screens. Finally, we used bioinformatic approaches to examine the Sequence Read Archive (SRA) depositions for one individual from 1,341 arthropod species for the presence of *Rickettsia* and used this as a means of estimating the relative balance of Torix group to other *Rickettsia* within symbioses.

Data Description

Barcode of Life Data System (BOLD)

While searching the Barcode of Life Data System (BOLD), a depository of >8 million *COI* mtDNA sequences, hundreds of hits were observed with high sequence similarity to Torix group *Rickettsia*. To investigate the diversity and host distribution of these non-target amplicons, access was permitted to analyse *COI* barcoding data deriving from a BOLD screening project totaling 184,585 arthropod specimens from 21 countries and collected between 2010 and 2014. *COI* sequences provided by BOLD were generally derived from templates created from somatic tissues (legs are often used in order to retain most of the specimen for further analyses if necessary), but also rarely included abdominal tissues. The first dataset made available [36] included 3,817 sequences deemed as contaminant sequences as a result of not matching initial morphotaxa assignment. The second dataset included 55,366 specimens judged to not contain non-target amplicons [37]. A remaining 125,402 specimens were not made available, and the 55,366 subsample was used as a representative sample from which the contaminants had originated (Figure 1). The protocols for data collection, data curation and quality control of submitted BOLD samples is described by Ratnasingham & Hebert [38].

Sequence Read Archive (SRA)

Further insights into the balance of *Rickettsia* groups within arthropod symbioses were obtained through searching for *Rickettsia* presence in Illumina datasets associated with arthropod whole genome sequence (WGS) projects in the SRA (60,409 records as of the 20th May 2019). To reduce the bias from over-represented laboratory model species (e.g. *Drosophila* spp., *Anopheles* spp.) a single dataset per species was examined, and where multiple data sets existed for a species, that with the largest read count was retained. The

resultant dataset [39], representing 1,341 arthropod species, was then screened with phyloFlash [40] which finds, extracts and identifies 16S rRNA sequences.

Targeted screen of aquatic and terrestrial arthropods

A targeted PCR *Rickettsia* screen of 1,612 individuals from 169 species (Table 1) was undertaken as an adjunct to the BOLD and SRA searches to increase our understanding of *Rickettsia* ecology. Within this, we included a range of both aquatic and terrestrial taxa, to investigate if the previous work highlighting particular aquatic taxa as hotspots for *Rickettsia* symbiosis (water beetles, biting midges, damselflies) reflects a wider higher incidence in species from this habitat.

Analyses

Torix Rickettsia is the most common bacterial contaminant produced during barcoding projects

Out of 3,817 sequences considered contaminants, 1,126 of these were deemed by BOLD to be bacterial in origin (Figure 1, [36]). Phylogenetic placement supported the correct designation of these sequences as of microbial origin (Figure 2). The dominant genus was *Rickettsia* with 753 (66.9%) amplifications, compared to *Wolbachia* with 306 (27.2%). Of the remaining 67 non-target sequences, 16 formed a monophyletic group with other Anaplasmataceae and 51 were undesignated proteobacteria. When considering the 184,585 specimens in the total project, this analysis gave an overall *Rickettsia* and *Wolbachia* prevalence of 0.41% and 0.17% respectively within the dataset. Through later access to the 55,366 representative data subset from where the contaminants originated, further unique

bacteria contaminants were also detected (possibly missed by BOLD's automated contaminant filtering system). This suggests these prevalences are conservative estimates.

BOLD *Rickettsia* contaminants were dominated by amplicons from the Torix group of *Rickettsia* (716/753; 95.1%) (Figure 3). The remaining 37 *Rickettsia* clustered with Transitional/Spotted Fever (n=15), Belli (n=9), Rhyzobius (n=1) groups, while 12 sequences formed two unique clades. Across arthropod hosts: 292 (38.8%) were derived from Hymenoptera; 189 (25.1%) from Diptera; 177 from Hemiptera (23.5%); 41 from Psocoptera (5.4%); 40 from Coleoptera (5.3%); 7 from Arachnida (0.9%); 4 from Trichoptera (0.5%); and single cases of Thysanoptera, Diplopoda and Dermaptera (0.1% each). Mapping the 753 *Rickettsia* to collection site (Additional file 1) revealed arthropod infections predominantly from Canada with other locations in South/Central America, Europe, Africa and Asia.

We observed that two sets of *COI* primers were responsible for 99% of *Rickettsia* amplifications (Additional file 2) with a majority (89%) amplifying with the primer combination C_LepFolF/C_LepFolR [41]. Torix *Rickettsia COI* showed a stronger match to these primers at the 3' end (the site responsible for efficient primer annealing) compared to *Wolbachia* and other *Rickettsia* groups. Whilst all contained a SNP at the 3' priming end of C_LepFolR, Torix *Rickettsia* (*Rickettsia* endosymbiont of *Culicoides newsteadi*; MWZE00000000) was the only sequence to not contain a similar SNP at the 3' priming site of C_LepFolF (Additional file 3).

Rickettsia multilocus phylogenetic analysis

To better resolve the phylogenetic relationships between BOLD *Rickettsia* contaminants, a multilocus approach was employed on a subsample of 186 *Rickettsia*-containing samples. To this end, 2 further housekeeping genes (*16S rRNA*, *gltA*) and the antigenic *17KDa* protein gene were amplified from the respective templates.

Overall, 135 extracts successfully amplified and gave a high-quality sequence for at least one gene. No intragenic or intergenic recombination was detected for any of the gene profiles. A phylogram, including 99 multilocus profiles containing at least 3 of the 4 *Rickettsia* genes of interest (including *COI*), allocated strains to both Limoniae and Leech subclades of the Torix group (Figure 4) and these subclades were derived from similar hosts. For example, specific families (Hemiptera: Psyliidae and Hymenoptera: Diapriidae) were present in both Leech and Limoniae groups. A full list of multilocus profiles and *Rickettsia* group designation can be found in Additional file 4.

The multilocus study also provided evidence of co-infection with *Rickettsia*. During Sanger chromatogram analysis, double peaks were occasionally found at third codon sites from protein coding genes. This pattern was observed in 6/10 *Philotarsus californicus* individuals and in one member of each of the Psilidae, Sciaridae, Chironomidae and Diapriidae (Additional file 4). Where double peaks were observed, this was found consistently across markers within an individual specimen. This pattern indicates co-infecting *Rickettsia* strains in hosts is a widespread phenomenon of the Torix group.

Barcoding success of host taxa

An available subset of attempted barcodes associated with the contaminants contained 55,366 out of 184,585 arthropods originally used in the overall study [37]. The three classes of Insecta (n=49,688), Arachnida (n=3,626) and Collembola (n=1,957), accounted for >99.8% of total specimens (Figure 1). Successful amplification and sequencing of *COI* was achieved in 43,246 specimens (78.1%) of the genomic extracts, but when assessed at the order level success rates varied (Additional file 5). The likely explanation for this variation is taxa-specific divergence of sequences at priming sites.

The number of each taxonomic order giving at least one *Rickettsia* amplification was then calculated and adjusted based on the total number of specimens in the project to allow for a prevalence estimate. Overall, Hymenoptera, Diptera and Hemiptera were the three taxa most likely to be associated with *Rickettsia COI* amplification (87.4%). Similarly, on assessment of a subsample from the project where the contaminants originated, a majority (77.7%) of the dataset were also accounted for by these three orders. After adjusting the prevalence to take into account the number of inaccessible specimens, Trichoptera (2.45%), Dermaptera (1.89%) and Psocodea (1.67%) were the most likely taxa to give an inadvertent *Rickettsia* amplification. Despite Hemiptera and Diptera having a similar estimated prevalence (0.58% and 0.56%), Hemiptera were much more likely to fail to barcode (67.2% vs 93.3%) indicating the true dipteran prevalence is likely to be higher, as a barcoding failure is necessary to amplify non-target bacteria *COI*. Attempts to re-barcode 186 *Rickettsia*-containing DNA templates of interest from BOLD resulted in 90 successful arthropod host barcodes (Additional file 4).

Rickettsia bacterial diversity detected by Targeted and SRA searches

The targeted *Rickettsia* screen of 1,612 individuals from 169 invertebrate species detected infections in 16 species (9.47%) including both aquatic and terrestrial taxa (Table 1). Of these, 14 profiles clustered within the Torix group with the remaining two placed in the Belli and Rhyzobius groups (Figure 5). Comparisons of Torix *Rickettsia* frequency between aquatic/semiaquatic vs terrestrial arthropods revealed evidence for a higher representation of Torix *Rickettsia* infected species in the aquatic biome (Fisher's Exact, $P = 0.019$).

[Insert Table 1 here]

During the SRA search, phyloFlash flagged 29 *Rickettsia* sequences in the groups: Belli (n=12), Torix (n=8), Transitional (n=6), Rhyzobius (n=2), and Spotted Fever (n=1) (Figure 5). In addition, Kraken identified nine *Rickettsia*-containing arthropod SRA datasets missed by phyloFlash. Two of these were from the Torix group, in phantom midge hosts (Diptera: Chaobaridae: *Mochlonyx cinctipes* and *Chaoborus trivitattus*), with the remaining seven placed in Belli and Spotted Fever groups [39]. The search of GenBank revealed 11 deposits ascribed to host mtDNA that were in fact Torix *Rickettsia* sequences (Additional files 6 and 7).

The hidden host diversity of Torix Rickettsia

Overall, novel Torix hosts detected from all screening methods included taxa from the orders Dermaptera, Gastropoda, Trichoptera and Trombidiformes. Additionally, new Torix-associated families, genera and species were identified. These included haematophagous flies (*Simulim aureum; Anopheles plumbeus; Protocalliphora azurea*; Tabanidae), several parasitoid wasp families (e.g. Ceraphronidae; Diapriidae; Mymaridae), forest detritivores (e.g. Sciaridae;

Mycetophilidae; Staphylinidae) and phloem-feeding bugs (Psyllidae; Ricaniidae). Feeding habits such as phloem-feeding, predation, detritivory or haematophagy were not correlated with any particular Torix *Rickettsia* subclade (Figure 6). Furthermore, parasitoid and aquatic lifestyles were seen across the phylogeny. All newly discovered Torix *Rickettsia* host taxa are described in Table 2, alongside previously discovered hosts in order to give an up to date overview of Torix-associated taxa.

[Insert Table 2 here]

Discussion

Symbiotic interactions between hosts and microbes are important drivers of host phenotype, with symbionts both contributing to, and degrading, host performance. Heritable microbes are particularly important contributors to arthropod biology, with marked attention focused on *Wolbachia*, the most common associate [9]. Members of the Rickettsiales, like *Wolbachia*, share an evolutionary history with mitochondria [42], such that a previous screen of BOLD submissions of mtDNA submissions observed *Wolbachia* as the main bacterial contaminant associated with DNA barcoding [34]. However, our BOLD screen found that *Rickettsia* were more likely to be amplified than *Wolbachia* (0.41% vs 0.17% of deposits). Furthermore, Torix group *Rickettsia* were overrepresented in barcode misamplifications (95%) when compared to other groups within the genus. A comparison of the most commonly used barcoding primers to *Wolbachia* and *Rickettsia* genomes suggest homology of the forward primer 3' end was likely responsible for this bias towards Torix *Rickettsia* amplification. To gain a clearer understanding of the relative balance of Torix group to other *Rickettsia* within symbioses and

habitats, a targeted screen and bioinformatic approach was also undertaken. Through these three screens, a broad range of host diversity associated with Torix *Rickettsia* was uncovered.

As the *in silico* and empirical evidence suggests *Rickettsia COI* amplification is not uncommon [31–33], why has this phenomenon not been described more widely before? The conduction of a previous large-scale non-target *COI* study using BOLD submissions [34], revealed only *Wolbachia* hits. This screen involved comparison to a *Wolbachia*-specific reference library and was thus likely to miss *Rickettsia*. Additionally, there has been a lack of Torix *Rickettsia COI* homologues to compare barcodes to until recently, where a multilocus identification system, including *COI* was devised [26]. Indeed, out of the contaminant dataset received in this study, some of the *Rickettsia* contaminants were tentatively described by BOLD as *Wolbachia* due to the previous absence of publicly available *Rickettsia COI* to compare.

Although *Rickettsia* will only interfere with barcoding in a minority of cases (~0.4%), it is likely that alternate screening primers for some studies will need to be considered. In a demonstration of how unintended *Rickettsia* amplifications can affect phylogeographic studies relying on DNA barcoding, a *Rickettsia COI* was conflated with the mtDNA *COI* of a species of freshwater amphipod, *Paracalliope fluvitalis* [30]. Subsequently, supposed unique mtDNA haplotypes were allocated to a particular collection site, whereas this merely demonstrated the presence of Torix *Rickettsia* in host individuals in this lake. Contrastingly, non-target *Rickettsia* amplification can also allow for the elucidation of a novel host range of the symbiont [31–33] and this has been exemplified with our probing of BOLD.

Previously, several host orders have been associated with Torix *Rickettsia*, including Araneae, Coleoptera, Diptera, Hemiptera and Odonata [27,28,43–45]. However, newly uncovered host orders from this study include Dermaptera (earwigs), Gastropoda (snails), Trichoptera (caddis flies) and Trombidiformes (mites) (Table 2). Caution needs to be taken when interpreting what these newly found associations mean, as mere presence of *Rickettsia* DNA does not definitively indicate an endosymbiotic association. Indeed, parasitism or ingestion of symbiont-infected biota can also result in PCR detection [46–48]. Additionally, by calculating barcode success rate at an order level, Hemiptera were deemed to fail barcoding (either lack of amplification and/or quality sequence) more commonly than Diptera despite having a similar estimated *Rickettsia* prevalence (Additional file 5). As an increased barcoding failure rate is correlated with non-target *COI* amplification, it is probable there is a higher overall proportion of Torix *Rickettsia*-associated Diptera than Hemiptera in BOLD and likely in nature.

Model-based estimation techniques suggest *Rickettsia* are present in between 20-42% of arthropod species [12]. However, targeted screens often underestimate the incidence of *Rickettsia* hosts due to various methodological biases including small within-species sample sizes (missing low-prevalence infections) and the use of non-conserved primers [29]. Importantly, the inclusion and exclusion of specific ecological niches can also lead to a skewed view of *Rickettsia* symbioses. A previous review of *Rickettsia* bacterial and host diversity by Weinert et al. [13] suggested a possible (true) bias towards aquatic taxa in the Torix group. In accordance with this, our targeted screen demonstrated Torix *Rickettsia* infections were more prevalent in aquatic arthropod species compared to terrestrial. However, our observed over-representation of Torix group *Rickettsia* (14/16 strains) contrasts with Weinert's findings

which show a predominance of Belli infections and is likely due to the latter study's absence of screened aquatic taxa. Furthermore, through the additional use of a bioinformatics approach, our SRA search appears to confirm that Belli and Torix are two of the most common *Rickettsia* groups among arthropods. Overall, these multiple screening methods suggest Torix *Rickettsia* are more widespread than previously thought and their biological significance underestimated.

Previous studies have used either one or two markers to identify the relatedness of strains found in distinct hosts. In this study, we use the multilocus approach developed in Pilgrim et al. [26] to understand the affiliation of Torix *Rickettsia* from diverse invertebrate hosts. Our analysis of Torix strains indicates that closely related strains are found in distantly related taxa. Closely related *Rickettsia* are also found in hosts from different niches and habitats – for instance, the *Rickettsia* strains found in terrestrial blood feeders do not lie in a single clade, but rather are allied to strains found in non-blood feeding host species. Likewise, strains in phloem feeding insects are diverse rather than commonly shared.

The distribution of Torix *Rickettsia* across a broad host range suggests host shifts are occurring between distantly related taxa. It is notable that parasitoid wasps are commonly infected with *Rickettsia* and have been associated with enabling symbiont host shifts [49,50]. Aside from endoparasitoids, it is also possible that plant-feeding can allow for endosymbiont horizontal transmission [51,52]. For example, *Rickettsia* horizontal transmission has been demonstrated in *Bemisia* whiteflies infected by phloem-feeding [51,53]. Finally, ectoparasites like the Torix-infected water mites of the Calyptostomatidae family, could also play a role in establishing

novel *Rickettsia*-host associations, as feeding by mites has been observed to lead to host shifts for other endosymbiont taxa [54]. Indeed, if multiple horizontal transmission paths do exist, this could account for the diverse plethora of infected taxa, as well as arthropods identified in this study which harbour more than one strain of symbiont [55].

The finding that Torix *Rickettsia* are associated with a broad range of invertebrates leads to an obvious question: what is the impact and importance of these symbiotic associations? Previous work has established Torix *Rickettsia* represent heritable symbionts and it is likely that this is true generally. There have, however, been few studies on their impact on the host. In the earliest studies [22,23], *Torix* spp. leeches infected with *Rickettsia* were observed to be substantially larger than their uninfected counterparts. Since then, the only observation of note, pertaining to the Torix group, is the reduced ballooning (dispersal) behaviour observed in infected *Erigone atra* money spiders [56]. Overall, the incongruencies in host and Torix *Rickettsia* phylogenies (suggesting a lack of co-speciation and obligate mutualism), along with the lack of observed sex bias in carrying the symbiont, indicate facultative benefits are the most likely symbiotic relationship [29]. However, *Rickettsia* induction of thelytokous parthenogenesis should not be discounted in Torix infected parasitoid wasps identified in this study [57,58]. To add to the challenge of understanding Torix *Rickettsia* symbioses, the challenges of laboratory rearing of many Torix *Rickettsia* hosts has led to difficulties in identifying model systems to work with. However, the large expansion of our Torix group host knowledge can now allow for a focus on cultivatable hosts (e.g phloem-feeding bugs).

To conclude, we have shown that large-scale DNA barcoding initiatives of arthropods can include non-target amplification of Torix *Rickettsia*. By examining these non-target sequences, alongside a targeted screen and SRA search, we have uncovered numerous previously undetected host associations. Our findings lay bare multiple new avenues of inquiry for Torix *Rickettsia* symbioses.

Potential Implications

A particularly important group for future study of Torix *Rickettsia* interactions are haematophagous host species. Our discovery of *Rickettsia*-associated tabanid and simulid flies, alongside *Anopheles plumbeus* mosquitoes, add to existing blood-feeders previously identified as Torix group hosts which include sand flies [59,60], fleas [61], ticks [62,63] bed bugs [64] and biting midges [26]. Some *Rickettsia* strains are known to be transmitted to vertebrates via haematophagy [65]. However, there is no evidence to date for vertebrate pathogenic potential for the Torix group. Despite this, Torix *Rickettsia* could still play a significant role in the ecology of vectors of disease. A key avenue of research is whether these endosymbionts alter vectorial capacity, as found for other associations [66]. In contrast to the widely reported virus blocking phenotype observed in *Wolbachia*-infected vectors [67,68], *Rickettsia* have been associated with a virus potentiating effect in *Bemisia* white flies vectoring Tomato yellow leaf curl virus [69]. Additionally, we uncovered a *Rickettsia*-infected psyllid (*Cacopsylla melanoneura*) which is a vector of *Phytoplasma mali* (apple proliferation) [70]. Thus, the question of Torix *Rickettsia* vector-competence effects is clearly of widespread relevance and deserves further attention.

Methods

a) Interrogation of the Barcode of Life Data System (BOLD)

Assessment of non-target microbe amplicons

The designation of bacterial contaminants from a BOLD contaminant dataset containing 3,817 sequences [36] was refined by phylogenetic placement. To this end, barcodes confirmed as microbial sequences were aligned using the "L-INS-I" algorithm in MAFFT v7.4 [71] before using Gblocks [72] to exclude areas of the alignment with excessive gaps or poor alignment. ModelFinder [73] then determined the TIM3+F+I+G4 model to be used after selection based on default "auto" parameters using the Bayesian information criteria. A maximum likelihood (ML) phylogeny was then estimated with IQTree [74] using an alignment of 561 nucleotides and 1000 ultrafast bootstraps [75]. The Rickettsiales genera *Anaplasma*, *Neorickettsia*, *Rickettsia* and *Wolbachia* (Supergroups A, B, E, F, H) were included in the analysis as references. Finally, phylogenetic trees were drawn and annotated based on host taxa (order) using the EvolView [76] online tree annotation and visualisation tools.

A determining factor for non-target amplification of bacteria is primer site matching to microbial associates. Subsequently, pairwise homology of the primer set predominantly used for BOLD barcode screening was compared to *Rickettsia* and *Wolbachia COI* genes.

Further phylogenetic analysis

COI sequence alone provides an impression of the frequency with which *Rickettsia* associates are found in barcoding studies. However, they have limited value in describing the diversity of the *Rickettsia* found. To provide further insight into the diversity of *Rickettsia* using a

multilocus approach, we obtained 186 DNA extracts from the archive at the Centre for Biodiversity Genomics (University of Guelph, Canada) that had provided *Rickettsia* amplicons in the previous screen. Templates were chosen based on varied collection site, host order and phylogenetic placement. Multilocus PCR screening and phylogenetic analysis of *Rickettsia* was then completed, using the methodology in Pilgrim et al. [26]. However, slight variations include the exclusion of the *atpA* gene due to observed recombination at this locus. Furthermore, the amplification conditions for the *17KDa* locus was changed because a Torix *Rickettsia* reference DNA extract (Host: *Simulium aureum*) failed to amplify with the primer set Ri_17KD_F/ Ri_17KD_R from Pilgrim et al. [26]. Subsequently, a *17KDa* alignment from genomes spanning the Spotted fever, Typhus, Transitional, Belli, Limoniae groups, and the genus *Megaira* was generated to design a new set of primers using the online tool PriFi [77].

Once multilocus profiles of the *Rickettsia* had been established, we tested for recombination within and between loci using RDP v4 [78] using the MaxChi, RDP, Chimaera, Bootscan and GENECONV algorithms with the following criteria to assess a true recombination positive: a p-value of <0.001; sequences were considered linear with 1000 permutations being performed. Samples amplifying at least 3 out of 4 genes (*16S rRNA*, *17KDa*, *COI* and *gltA*) were then concatenated and their relatedness estimated using maximum likelihood as previously described. The selected models used in the concatenated partition scheme [79] were as follows: *16S rRNA*: TIM3+F+R2; *17KDa*: GTR+F+I+G4; *COI:*TVM+F+I+G4; *gltA:* TVM+F+I+G4. Accession numbers for all sequences used in phylogenetic analyses can be found in Additional file 8.

Re-barcoding Rickettsia-containing BOLD DNA extracts

Aside from phylogenetic placement of these *Rickettsia*-containing samples, attempts were made to extract an mtDNA barcode from these taxa in order to identify the hosts of infected specimens. Previous non-target amplification of *Rickettsia* through DNA barcoding of arthropod DNA extracts had occurred in the bed bug *Cimex lectularius*, with a recovery of the true barcode after using the primer set C1-J-1718/HCO1490, which amplifies a shortened 455 bp sequence within the *COI* locus. Subsequently, all samples were screened using these primers or a further set of secondary *COI* primers (LCOt_1490/ MLepR1 and LepF1/C_ANTMR1D) if the first failed to give an adequate host barcode. All *COI* and *Rickettsia* multilocus screening primer details, including references, are available in Additional file 9.

Cycling conditions for *COI* PCRs were as follows: initial denaturation at 95°C for 5 min, followed by 35 cycles of denaturation (94°C, 30 sec), annealing (50°C, 60 sec), extension (72°C, 90 sec), and a final extension at 72°C for 7 min. *Rickettsia* and host amplicons identified by gel electrophoresis were subsequently purified enzymatically (ExoSAP) and Sanger sequenced through both strands using a BigDye® Terminator v3.1 kit (Thermo Scientific, Waltham, USA), and capillary sequenced on a 3500 xL Genetic Analyser (Applied Biosystems, Austin, USA). Forward and reverse reads were assessed in UGENE [80] to create a consensus sequence by eye with a cut-off phred (Q) score [81] of 20. Primer regions were trimmed from barcodes before being matched to the GenBank database by BLAST based on default parameters and an e-value threshold of <1e-85. Host taxonomy was determined by a barcode-based assignment of the closest BLAST hit, under the following criteria modified from Ramage et al. [48]:

1) Species level designation for at least 98% sequence identity.

2) Genus level designation for at least 95% sequence identity.

3) Family level designation for at least 85% sequence identity.

Additionally, all sequences were required to be at least >200 bp in length.

Assessment of barcoding success

One of the factors determining a successful *COI* bacterial amplification is the initial failure of an extract to amplify mtDNA. Subsequently, to determine the likelihood of this event within taxa, we used the 55,366 specimen representative data subset [37] to evaluate failure rates. To this end, all orders of host which gave at least one non-target *Rickettsia COI* hit were assessed. The barcoding success rate was determined as the proportion of specimens which matched initial morphotaxa assignment and were not removed after BOLD quality control [38]. As the total *Rickettsia* count was from a larger dataset than the one made available, an adjusted prevalence for each taxon was calculated based on the representative data subset.

b) Targeted and bioinformatic *Rickettsia* screens

Targeted screen of aquatic and terrestrial arthropods

Overall, 1,612 individuals from 169 species, including both terrestrial (DNA templates derived from European material, mostly from Duron et al. [11])and aquatic invertebrates (largely acquired from the UK between 2016-2018), were screened. mtDNA *COI* amplification was conducted as a control for DNA quality. To investigate symbiont infection status, rickettsial-specific primers based on *gltA* and *16S rRNA* genes were used for conventional PCR screening [26], with Sanger sequences obtained from at least one specimen per *Rickettsia* positive

species to identify any misamplification false positives. Subsequently, the number of species containing at least one Torix *Rickettsia* positive individual were compared between terrestrial and aquatic environments using the Fisher's Exact test. Additionally, newly identified hosts of interest from BOLD and targeted screens were also placed phylogenetically (see sections above) before being mapped by lifestyle and diet.

Search of the Sequence Read Archive (SRA) and GenBank

The SRA dataset [39] containing one individual from 1,341 arthropod species was screened with phyloFlash [40] which finds, extracts and identifies *16S rRNA* sequences. Reconstructed full *16S rRNA* sequences affiliated to *Rickettsia* were extracted and compared to sequences derived from the targeted screen phylogenetically (see sections above) to assess group representation within the genus. The microbial composition of all SRA datasets that did not result in a reconstructed *Rickettsia 16S rRNA* with phyloFlash were re-evaluated using Kraken2 [82], a k-mer based taxonomic classifier for short DNA sequences. A cut-off of at least 40k reads assigned to *Rickettsia* taxa was applied for reporting potential infections (theoretical genome coverage of ~ 1 – 4X assuming an average genome size of ~1.5Mb).

We also examined GenBank for *Rickettsia* sequences deposited as invertebrate *COI* barcodes. To this end, a BLAST search of Torix *Rickettsia COI* sequences from previous studies [26,32] was conducted on the 29th June 2020. Sequences were initially considered belonging to the Torix group if their similarity was >90% and subsequently confirmed phylogenetically as described above.

Table 1.1. Targeted *Rickettsia* screen of aquatic/semiaquatic invertebrates.

Aquatic/Semiaquatic invertebrate group	Species	Location	Year	No. tested	No positive
Ephemeroptera	*Baetis muticus*	Stirling, Scotland, UK	2017	3	0
	Baetis rhodani	Stirling, Scotland, UK	2017	3	0
	Cloeon dipterum	Cheshire, UK	2016	3	0
	Ecdyonurus sp.1	Stirling, Scotland, UK	2017	5	0
	Ecdyonurus sp.2	Cheshire, UK	2016	3	0
	Ecdyonurus venosus	Cheshire, UK	2016	6	0
	Leptophlebia vespertina	Hampshire, UK	2016	1	0
	Paraleptophlebia submaginata	Stirling, Scotland, UK	2017	3	0
	Rhithrogena simicolorata	Stirling, Scotland, UK	2017	3	0
Trichoptera	*Hydrospyche* sp.	Stirling, Scotland, UK	2017	3	0
	Polycentropus flavomaculatus	Cheshire, UK	2017	3	0
	Rhyacophila dorsalis	**Stirling, Scotland, UK**	**2017**	**3**	**2**
Plecoptera	*Amphinemura sulcicollis*	Stirling, Scotland, UK	2017	3	0
	Dinocras cephalotes	Stirling, Scotland, UK	2017	3	0
	Isoperla grammatica	Stirling, Scotland, UK	2017	3	0
	Perla bipunctata	Stirling, Scotland, UK	2017	3	0
Hemiptera	*Corixa punctata*	Cheshire, UK	2016	1	0
	Gerris sp.	Montferrier sur Lez, France	2006	12	0
	Gerris thoracicus	Cheshire, UK	2016	1	0
	Hydrometra stagnorum	Montferrier sur Lez, France	2006	20	0
	Nepa cinerea	Montferrier sur Lez, France	2006	3	0
	Notonecta glauca	Cheshire, UK	2016	2	0
	Plea minutissima	Notre Dame de Londres, France	2006	8	0
	Sigara lateralis	Notre Dame de Londres, France	2006	6	0
	Sigara striata	**Cheshire, UK**	**2006**	**2**	**1**
Diptera	*Aedes* sp.	Cheshire, UK	2017	8	0
	Aedes albopictus	Roma, Italy	2005	20	0
	Anopheles plumbeus	**Chester Zoo, UK**	**2018**	**2**	**2**
	**Chironomidae sp.**	**Cheshire, UK**	**2016**	**4**	**1**
	Chironomus acidophilus	Cheshire, UK	2017	1	0
	Chironomus plumosus	Notre Dame de Londres, France	2006	20	0
	Chironomus sp.	Cheshire, UK	2016	4	0
	Culex pipiens (ssp. quinquefasciatus)	Puerto Viejo de Talamanca, Costa Rica	2006	20	0
	Culex pipiens pipiens	St Nazaire de Pézan, France	2006	20	0
	Eristalinus sp.	Cheshire, UK	2016	3	0
	Eristalis tenax	Montpellier (grotte du zoo), France	2002	7	0
	Glyptotendipes **sp.**	**Cheshire, UK**	**2016**	**1**	**1**
	Hilara **interstincta**	**Cheshire, UK**	**2017**	**3**	**1**
	Medetera petrophila	St Bauzille de Putois, France	2003	12	0
	Simulium aureum	**Hampshire, UK**	**2017**	**1**	**1**
	Simulium ornatum	N/A	2003	12	0
	Tipula sp.	UK	2006	10	0
	Tipula oleracea	UK	2006	13	0
	Zavrelimyia **sp.**	**Northumberland, UK**	**2017**	**1**	**1**
Coleoptera	*Agabus bipustulatus*	Cheshire, UK	2017	3	0
	Guignotus pusillus	Notre Dame de Londres, France	2006	12	0
	Unknown sp.1	Cheshire, UK	2017	2	0
	Unknown sp.2	Cheshire, UK	2017	3	0

Acarina	Unknown sp.	Cheshire, UK	2017	3	0
Isopoda	*Asellus aquaticus*	Cheshire, UK	2016	3	0
Amphipoda	*Gammarus pulex*	Stirling, Scotland, UK	2017	3	0
	Crangonyx pseudogracilis	Cheshire, UK	2016	6	0
	Radix balthica	Cheshire, UK	2016	3	0
Gastropoda	*Planorbis* sp.	Cheshire, UK	2016	3	0
	Galba truncatula	**Cheshire, UK**	**2017**	**20**	**3**
Hirudinea	*Erpobdella octaculata*	Cheshire, UK	2016	2	0
	Hemiclepsis marginata	Cheshire, UK	2017	1	0
Tricladida	Unknown sp.	Cheshire, UK	2016	1	0

A species was deemed positive through PCR and designated to *Rickettsia* group after Sanger sequencing and phylogenetic placement. All strains belong to the Torix group.

Table 1.2. Targeted *Rickettsia* screen of terrestrial invertebrates.

Terrestrial Invertebrate group	Species	Location	Year	Number tested	Number positive
	Agelenopsis aperta	Tennessee, USA	N/A	12	0
	Allopecosa pulverulenta	Berne, Germany	N/A	16	0
	Amaurobius fenestralis	Montpellier, France	2006	16	0
	Araneus diadematus	Beerse, Belgium	N/A	19	0
	Araneus diadematus	Greater London, UK	N/A	8	0
	Argiope bruennichi	Hamburg, Germany	N/A	7	0
	Argiope lobata	Spain	N/A	7	0
	Argiope lobata	Israel	N/A	4	0
	Cyclosa conica	Brandenburg, Germany	N/A	11	0
	Dysdera crocata	Montpellier, France	2006	2	0
	Enoplognatha ovata	Greater London, UK	N/A	20	0
	Erigone atra	Cheshire, UK	2017	1	0
	Evarcha falcata	Beerse, Belgium	N/A	5	0
	Holochnemus pluchei	Montpellier, France	2006	7	0
	Hylyphantes graminicola	Cheshire, UK	2017	1	0
	Larinioides cornutus	Greater London, UK	N/A	6	0
	Larinoides sclopetarius	Hamburg, Germany	N/A	17	0
	Linyphia triangularis	**Berlin, Germany**	**N/A**	**9**	**9**
	Linyphia triangularis	Greater London, UK	N/A	6	0
Araneae	*Lycosa* sp.	Cheshire, UK	2017	2	0
	Meta mengei	Greater London, UK	N/A	13	0
	Meta segmentata	Brandenburg, Germany	N/A	9	0
	Neriene clathrata	Beerse, Belgium	N/A	13	0
	Neriene peltata	Cheshire, UK	2017	1	0
	Pachygnatha degeeri	Berne, Germany	N/A	11	0
	Pachygnatha listeri	Beerse, Belgium	N/A	17	0
	Pardosa lugubris	Darmstadt, Germany	N/A	20	0
	Pardosa pullata	Brandenburg, Germany	N/A	20	0
	Pardosa purbeckensis	Belgium	N/A	19	0
	Pholcus phalangioides	**Berlin, Germany**	**N/A**	**20**	**17**
	Pisaura mirabilis	**Greater London, UK**	**N/A**	**12**	**1**
	Tetragnatha montana	Greater London, UK	N/A	20	0
	Tetragnatha sp.	Hampshire, UK	2017	3	0
	Unknown sp.	Cheshire, UK	2017	2	0
	Xysticus cristatus	Cambridgeshire, UK	N/A	16	0

Opiliones	*Leiobunum rotundum*	Feurs, France	2006	6	0
Ixodida	*Ixodes uriae*	Hornøya, Norway	2005	19	0
	Rhipicephalus microplus	New Caledonia, France	2003	1	0
Scorpiones	*Euscorpius flavicauda*	St Nazaire de Pézan, France	2006	1	0
Diplopoda	*Ommatoiulus* sp.	Cheshire, UK	2016	1	0
Neuroptera	Unknown sp.	Cheshire, UK	2017	1	0
Mecoptera	*Panorpa* sp.	Cheshire, UK	2017	2	0
	Calliptamus italicus	Notre Dame de Londres, France	2016	18	0
Orthoptera	*Chorthippus brunneus*	Uk	2006	20	0
	Gryllomorpha dalmatina	Montpellier, France	2006	2	0
Blattaria	*Loboptera decipiens*	Montpellier, France	2006	17	0
Mantodae	*Iris oratoria*	St Nazaire de Pézan, France	2006	6	0
	Mantis religiosa	Feurs, France	2006	3	0
Dermaptera	*Forficula Auricularia*	Feurs, France	2006	9	0
	Aphis fabae	Montpellier, France	2006	12	0
	Aphis nerii	Montpellier, France	2006	8	0
	Baizongia pistaciae	Viols le Fort, France	2006	12	0
	Cicadella viridis	L'Olme, France	2006	16	0
	Cimex lectularius	**Yorkshire, UK**	**2008**	**12**	**12**
Hemiptera	*Elasmucha grisea*	Greater London, UK	2006	16	0
	Graphosoma italicum	Montpellier, France	2006	12	0
	Lygaeus equestris	Montpellier, France	2006	12	0
	Notostira elongata	L'Olme, France	2006	11	0
	Pyrrhocoris apterus	Montpellier, France	2006	11	0
	Rhyparochromus vulgaris	Castelnaudary, France	2006	20	0
	Anaspis frontalis	Mont Barri, France	2004	12	0
	Anthaxia nitidula	Mont Barri, France	2004	20	0
	Anthaxia sp.	Mont Barri, France	2004	16	0
	Calvia 14-guttata	Greater London, UK	2006	6	0
	Capnodis tenebrionis	Montpellier, France	2006	1	0
	Cetonia aurata	Feurs, France	2006	3	0
	Cetonia aurata	Mont Barri, France	2004	12	0
	Chrysolina varians	Mont Barri, France	2004	18	0
	Clytus arietis	Mont Barri, France	2004	20	0
	Dermestes sp.	Mont Barri, France	2004	20	0
Coleoptera	*Dermestes tessellatocollis*	Cheshire, UK	2016	2	0
	Gastrophysa sp.	Greater London, UK	2006	20	0
	Geotrupes stercorarius	Mont Barri, France	2004	3	0
	Larinus scolymi	Aldira de Irmeros, Spain	2005	12	0
	Leptinotarsa decemlineata	Feurs, France	2006	10	0
	Leptura livida	Mont Barri, France	2004	19	0
	Mordellistena sp.	Mont Barri, France	2004	10	0
	Oedemera sp.	Mont Barri, France	2004	20	0
	Oncocerna sp.	Mont Barri, France	2004	20	0
	Phyllobius argentatus	**Mont Barri, France**	**2004**	**15**	**4†**
	Stenopterus sp.	Mont Barri, France	2004	20	0
	Braula coeca	Ouessant, France	2002	4	0
	Chorisops tunisiae	Montpellier, France	2003	8	0
	Delia antiqua	N/A	N/A	11	0
	Delia platura	N/A	N/A	11	0
	Delia radiacum	N/A	N/A	10	0
	Gasterophilus intestinalis	France	N/A	10	0
	Hippobosca equina	Restinclières, France	2006	15	0
Diptera	*Lonchoptera lutea*	Cheshire, UK	2017	3	0
	Musca domestica	L'Olme, France	2006	20	0
	Musca vitripennis	Notre Dame de Londres, France	2003	8	0

	Neomyia cornicina	Notre Dame de Londres, France	2003	8	0
	Protocalliphora sp.	Corse, France	2003	2	0
	Protocalliphora azurea	**Montpellier, France**	**2005**	**12**	**12**
	Psila rosae	N/A	N/A	11	0
	Stomoxys calcitrans	Le Malzieu, France	2001	11	0
Lepidoptera	*Chilo phragmitellus*	Feurs, France	2006	10	0
	Euplagia quadripunctaria	Feurs, France	2006	2	0
	Pieris brassicae	Feurs, France	2006	7	0
	Plodia interpunctella	Montpellier, France	2006	12	0
	Thymelicus lineola	Greater London, UK	2006	15	0
	Thymelicus sylvestris	Greater London, UK	2006	2	0
	Triodia sylvina	Montpellier, France	2006	4	0
Hymenoptera	*Amblyteles armatorius*	St Nazaire de Pézan, France	2006	1	0
	Amegilla albigena	St Nazaire de Pézan, France	2006	13	0
	Amegilla ochroleuca	St Nazaire de Pézan, France	2006	3	0
	Anthidium florentinum	St Nazaire de Pézan, France	2006	6	0
	Apis mellifera	UK	2006	9	0
	Bombus terrestris	North West, Switzerland	2006	20	0
	Diplolepis rosae	L'Olme, France	2006	2	0
	Formica lugubris	UK	2006	10	0
	Pachycrepoideus **sp.**	**UK**	**N/A**	**94**	**6‡**
	Polistes dominulus	St Nazaire de Pézan, France	2006	4	0
	Polistes nimpha	St Nazaire de Pézan, France	2006	19	0
	Sceliphron caementarium	St Nazaire de Pézan, France	2006	3	0

A species was deemed positive through PCR and designated to *Rickettsia* group after Sanger sequencing and phylogenetic placement. All strains belong to the Torix group except †=Rhyzobius and ‡=Belli.

Table 2. Torix *Rickettsia* hosts known to date alongside screening method.

Order	Host	Screening method	Reference
Amphipoda	***Paracalliope fluviatilis*** **(Paracalliopiidae)**	**GenBank search**	**This study**
	Paraleptamphopus sp. (Paraleptamphopidae)	Barcoding	[33]
	Senticaudata sp.	Barcoding	[33]
Araneae	*Amaurobioides africana* (Anyphaenidae)	Barcoding	[32]

	Araneus diadematus (Araneidae)	Targeted PCR	[43]
	Dysdera microdonta (Dysderidae)	Barcoding	[31]
	Linyphiidae spp.	Targeted PCR	[43]
	***Linyphia triangularis* (Linyphiidae)**	**Targeted PCR**	**This study**
	***Pholcus phalangioides* (Pholcidae)**	**Targeted PCR**	**This study**
	***Pisaura mirabilis* (Pisauridae)**	**Targeted PCR**	**This study**
	Meta mengei (Tetragnathidae)	Targeted PCR	[43]
Coleoptera	*Deronectes* spp. (Dytiscidae)	Targeted PCR, FISH and TEM	[27]
	***Dytiscidae* sp.**	**Barcoding**	**This study**
	Stegobium paniceum (Ptinidae)	Non-targeted (16S) PCR	[83]
	***Prionocyphon limbatus* (Scirtidae)**	**Barcoding**	**This study**
	***Labidopullus appendiculatus* (Staphylinidae)**	**SRA search**	**This study**
	***Platyusa sonomae* (Staphylinidae)**	**SRA search**	**This study**
	***Pseudomimeciton antennatum* (Staphylinidae)**	**SRA search**	**This study**
	***Staphylinidae* sp.**	**Barcoding**	**This study**
	***Pimelia* sp. (Tenebrionidae)**	**GenBank search**	**This study**
Dermaptera	***Forficula* sp. (Forficulidae**)	**GenBank search**	**This study**
	**unknown sp.**	**Barcoding**	**This study**
Diplopoda	*Polydesmus complanatus* (Polydesmidae)	Targeted PCR	[84]
	**unknown sp.**	**Barcoding**	**This study**
	***Protocalliphora azurea* (Calliphoridae)**	**Targeted PCR**	**This study**
	***Cecidomyiidae* sp.**	**Barcoding**	**This study**

Diptera	***Chaoborus trivitattus* (Chaobaridae)**	**SRA search**	**This study**
	***Mochlonyx cinctipes* (Chaobaridae)**	**SRA search**	**This study**
	***Glyptotendipes* sp. (Chironomidae)**	**Targeted PCR**	**This study**
	***Zavrelimyia* sp. (Chironomidae)**	**Targeted PCR**	**This study**
	Culicoides spp. (Ceratopogonidae)	Targeted PCR and FISH	[26]
	***Anopheles plumbeus* (Culicidae)**	**Targeted PCR**	**This study**
	Dolichopodidae spp.	Targeted PCR	[44]
	Empididae spp.	Targeted PCR	[44]
	Limonia chorea (Limoniidae)	N/A	Unpublished (AF322443)
	***Boletina villosa* (Mycetophilidae)**	**Barcoding**	**This study**
	**Gnoriste bilineata (Mycetophilidae)**	**SRA search**	**This study**
	***Mycetophila lunata* (Mycetophilidae)**	**GenBank search**	**This study**
	***Psilidae* sp.**	**Barcoding**	**This study**
	Lutzomyia apache (Psychodidae)	Targeted PCR	[60]
	Phlebotomus chinensis (Psychodidae)	Non-targeted (16S) PCR	[59]
	***Sciaridae* sp.**	**Barcoding**	**This study**
	***Pherbellia tenuipes* (Sciomyzidae)**	**Barcoding**	**This study**
	***Simulium aureum* (Simuliidae)**	**Targeted PCR**	**This study**
	***Tabanidae* sp.**	**Barcoding**	**This study**
Gastropoda	***Galba truncatula* (Lymnaeidae)**	**Targeted PCR**	**This study**
Haplotaxida	*Mesenchytraeus solifugus* (Enchytraediae)	Non-targeted (16S) PCR	[85]
	Bemisia tabaci (Aleyrodidae)	Targeted PCR and FISH	[86]
	Nephotettix cincticeps (Cicadellidae)	Targeted PCR, FISH and TEM	[87]
	Platypleura kaempferi (Cicadidae)	Non-targeted (16S) PCR	[88]

Hemiptera	***Cimex lectularius* (Cimicidae)**	**Targeted PCR**	**This study/**[64]
	***Sigara* striata (Corixidae)**	**Targeted PCR**	**This study**
	***Metcalfa pruinosa* (Flatidae)**	**GenBank search**	**This study**
	***Flavina* sp. (Issidae)**	**GenBank search**	**This study**
	Centrotus cornutus (Membracidae)	Non-targeted (16S) PCR and TEM	[89]
	Gargara genistae (Membracidae)	Non-targeted (16S) PCR and TEM	[89]
	Macrolophus pygmaeus (Miridae)	Non-targeted (16S) PCR and FISH	[45]
	***Cacopsylla melanoneura* (Psyllidae)**	**Barcoding**	**This study**
	***Chamaepsylla hartigii* (Psyllidae)**	**Barcoding**	**This study**
	***Ricaniidae* sp.**	**Barcoding**	**This study**
Hirudinea	*Hemiclepsis* spp. (Glossiphoniidae)	Targeted PCR and TEM	[23]
	Torix spp. (Glossiphoniidae)	Targeted PCR and TEM	[23]
Hymenoptera	*Asobara tabida* (Braconidae)	Non-targeted (16S) PCR	[90]
	***Ceraphronidae* sp.**	**Barcoding**	**This study**
	***Diapriidae* sp.**	**Barcoding**	**This study**
	***Eucharitidae* sp.**	**GenBank search**	**This study**
	Quadrastichus mendeli (Eulophidae)	Non-targeted (16S) PCR and FISH	[91]
	***Formicidae* sp.**	**GenBank search**	**This study**
	Atta colombica (Formicidae)	Non-targeted (16S) PCR	Unpublished (LN570502)
	***Megaspilidae* sp.**	**Barcoding**	**This study**
	***Mymaridae* sp.**	**Barcoding**	**This study**
	***Platygastridae* sp.**	**Barcoding**	**This study**
Ixodida	*Argas japonica* (Argasidae)	Non-targeted (16S) PCR	[63]

	Ixodes ricinus (Ixodidae)	Targeted PCR	[62]
Megaloptera	*Sialis lutaria* (Sialidae)	Targeted PCR	[92]
Neuroptera	*Chrysotropia ciliata* (Chrysopidae)	Targeted PCR	[92]
Nucleariida	*Nuclearia pattersoni* (Nucleariidae)	Non-targeted (16S) PCR	[24]
	Pompholyxophrys punicea (Pompholyxophryidae)	Single cell sequencing	[25]
Odonata	***Calopteryx maculata* (Calopterygidae)**	**GenBank search**	**This study**
	Coenagrionidae spp.	Targeted PCR and FISH	[28]
	Sympetrum fonscolombii (Libellulidae)	Targeted PCR	[28]
	Polythoridae spp.	Targeted PCR	[28]
	Neoneura sylvatica (Protoneuridae)	Targeted PCR	[28]
Psocoptera	***Myopsocidae* sp.**	**Barcoding**	**This study**
	***Philotarsus californicus* (Philotarsidae)**	**Barcoding**	**This study**
	Cerobasis guestfalica (Trogiidae)	Targeted PCR and FISH	[93]
Siphonoptera	*Nosopsyllus fasciatus* (Ceratophyllidae)	Targeted PCR	[61]
Trichoptera	***Lepidostoma hoodi* (Lepidostomatidae)**	**Barcoding**	**This study**
	***Rhyacophila dorsalis* (Rhyacophilidae)**	**Targeted PCR**	**This study**
	***Sericostoma* sp. (Sericostomatidae)**	**SRA search**	**This study**
Trombidiformes	***Calyptostomatidae* sp.**	**Barcoding**	**This study**

Bold entries indicate hosts identified in this study. FISH=fluoresence *in-situ* hybridisation; TEM=transmission electron microscopy; SRA=sequence read archive. Accession numbers for *Rickettsia* sequences from newly detected hosts can be found in Additional files 6 and 8.

Availability of Supporting Data and Materials

The data sets supporting the findings of this study are openly available in:

The Barcode of Life Data System (BOLD) repository at http://dx.doi.org/10.5883/DS-RICKET and the Figshare repository at http://dx.doi.org/10.6084/m9.figshare.12801107 and http://dx.doi.org/10.6084/m9.figshare.12801140.

For DNA sequences, EMBL accession codes are: LR798809-LR800243; LR812141-LR812260; LR812269-LR812283; LR812678; LR813674-LR813676; LR813730.

Declarations

List of Abbreviations

BOLD=Barcode of Life Data System

COI=cytochrome c oxidase I

SRA=Sequence Read Archive

Ethics Approval

Not applicable.

Consent for Publication

Not applicable.

Competing Interests

The authors declare that they have no competing interests.

Funding

This work was supported by: a BBSRC Doctoral Training Partnership studentship (BB/M011186/1) awarded to JP; a Development and Promotion of Science and Technology Talents Project (DPST) of the Institute for the Promotion of Teaching Science and Technology, Thailand to PT and a Harry Smith Vacation studentship (Microbiology society) to HRD.

Acknowledgments

We would like to thank Dr. Michael Gerth for kindly providing comments on the manuscript.

Author contributions

JP, GDDH, MB and MAS assisted in the conception and design of the study. MAS, EVZ, SR and JRD assisted in assembling BOLD datasets and providing DNA extracts for laboratory experiments. Field and laboratory work was undertaken by JP and PT. SRA work was undertaken by HRD and SS. Analyses and interpretation of the data were undertaken by JP, PT, HRD, GDDH, MB and SS, as well as drafting of the manuscript. All authors assisted in critical revision of the manuscript.

References

1. McFall-Ngai M, Hadfield MG, Bosch TCG, Carey H V., Domazet-Lošo T, Douglas AE, et al. Animals in a bacterial world, a new imperative for the life sciences. Proc Natl Acad Sci. 2013;110:3229–36.

2. Hurst GDD. Extended genomes: symbiosis and evolution. Interface Focus. 2017;7:20170001.

3. Łukasik P, Guo H, van Asch M, Ferrari J, Godfray HCJ. Protection against a fungal pathogen conferred by the aphid facultative endosymbionts Rickettsia and Spiroplasma is expressed in multiple host genotypes and species and is not influenced by co-infection with another symbiont. J Evol Biol. 2013;26:2654–61.

4. Teixeira L, Ferreira A, Ashburner M. The bacterial symbiont Wolbachia induces resistance to RNA viral infections in Drosophila melanogaster. PLoS Biol. 2008;6:2753–63.

5. Rio RVM, Attardo GM, Weiss BL. Grandeur Alliances: Symbiont metabolic integration and obligate arthropod hematophagy. Trends Parasitol. 2016;32:739–49.

6. Douglas AE. The microbial dimension in insect nutritional ecology. Funct Ecol. 2009;23:38–47.

7. Hurst GDD, Frost CL. Reproductive parasitism: Maternally inherited symbionts in a biparental world. Cold Spring Harb Perspect Biol. 2015;7:a017699.

8. Munson MA, Baumann P, Kinsey MG. Buchnera gen. nov. and Buchnera aphidicola sp. nov., a taxon consisting of the mycetocyte-associated, primary endosymbionts of aphids. Int J Syst Bacteriol. 1991;41:566–8.

9. Zug R, Hammerstein P. Still a host of hosts for Wolbachia: Analysis of recent data suggests that 40% of terrestrial arthropod species are infected. PLoS One. 2012;7:e38544.

10. Siozios S, Gerth M, Griffin JS, Hurst GDD. Symbiosis: Wolbachia host shifts in the fast lane. Curr Biol. 2018;28:R269–71.

11. Duron O, Bouchon D, Boutin S, Bellamy L, Zhou L, Engelstädter J, et al. The diversity of reproductive parasites among arthropods: Wolbachia do not walk alone. BMC Biol. 2008;6:27.

12. Weinert LA, Araujo-Jnr E V, Ahmed MZ, Welch JJ. The incidence of bacterial endosymbionts in terrestrial arthropods. Proc R Soc B. 2015;282:20150249.

13. Weinert LA, Werren JH, Aebi A, Stone GN, Jiggins FM. Evolution and diversity of Rickettsia bacteria. BMC Biol. 2009;7:6.

14. Perlman SJ, Hunter MS, Zchori-Fein E. The emerging diversity of Rickettsia. Proc R Soc B. 2006;273:2097–106.

15. Ricketts HT. A micro-organism which apparently has a specific relationship to Rocky Mountain spotted fever. J Am Med Assoc. 1909;52:379–80.

16. da Rocha-Lima H. Zur Aetiologie des Fleckfiebers. Dtsch Medizinische Wochenschrift. 1916;53:567–9.

17. Werren JH, Hurst GD, Zhang W, Breeuwer JA, Stouthamer R, Majerus ME. Rickettsial relative associated with male killing in the ladybird beetle (Adalia bipunctata). J Bacteriol. 1994;176:388–94.

18. Chen D-Q, Campbell BC, Purcell AH. A new Rickettsia from a herbivorous insect, the pea aphid Acyrthosiphon pisum (Harris). Curr Microbiol. 1996;33:123–8.

19. Hurst GDD, Walker LE, Majerus MEN. Bacterial infections of hemocytes associated with the maternally inherited male-killing trait in British populations of the two spot ladybird, Adalia bipunctata. J Invertebr Pathol. 1996;68:286–92.

20. Hendry TA, Hunter MS, Baltrus DA. The facultative symbiont Rickettsia protects an invasive whitefly against entomopathogenic Pseudomonas syringae strains. Appl Environ Microbiol. 2014;80:7161–8.

21. Himler AG, Adachi-Hagimori T, Bergen JE, Kozuch A, Kelly SE, Tabashnik BE, et al. Rapid spread of a bacterial symbiont in an invasive whitefly is driven by fitness benefits and female bias. Science. 2011;332:254–6.

22. Kikuchi Y, Fukatsu T. Rickettsia infection in natural leech populations. Microb Ecol. 2005;49:265–71.

23. Kikuchi Y, Sameshima S, Kitade O, Kojima J, Fukatsu T. Novel clade of Rickettsia spp. from leeches. Appl Environ Microbiol. 2002;68:999–1004.

24. Dyková I, Veverková M, Fiala I, Macháčková B, Pecková H. Nuclearia pattersoni sp. n. (Filosea), a new species of amphizoic amoeba isolated from gills of roach (Rutilus rutilus), and its Rickettsial endosymbiont. Folia Parasitol (Praha). 2003;50:161–70.

25. Galindo LJ, Torruella G, Moreira D, Eglit Y, Simpson AGB, Völcker E, et al. Combined cultivation and single-cell approaches to the phylogenomics of nucleariid amoebae, close relatives of fungi. Philos Trans R Soc B Biol Sci. 2019;374:20190094.

26. Pilgrim J, Ander M, Garros C, Baylis M, Hurst GDD, Siozios S. Torix group Rickettsia are widespread in Culicoides biting midges (Diptera: Ceratopogonidae), reach high frequency and carry unique genomic features. Environ Microbiol. 2017;19:4238–55.

27. Küchler SM, Kehl S, Dettner K. Characterization and localization of Rickettsia sp. in water beetles of genus Deronectes (Coleoptera: Dytiscidae). FEMS Microbiol Ecol. 2009;68:201–11.

28. Thongprem P, Davison HR, Thompson DJ, Lorenzo-Carballa MO, Hurst GDD. Incidence and diversity of Torix Rickettsia–Odonata symbioses. Microb Ecol. 2020;In Press. DOI: 10.1007/s00248-020-01568-9

29. Weinert LA. The diversity and phylogeny of Rickettsia. In: Morand S, Krasnov BR, Littlewood DTJ, editors. Parasite diversity and diversification. Cambridge: Cambridge University Press; 2015. p. 150–81.

30. Lagrue C, Joannes A, Poulin R, Blasco-Costa I. Genetic structure and host-parasite co-divergence: evidence for trait-specific local adaptation. Biol J Linn Soc. 2016;118:344–58.

31. Řezáč M, Gasparo F, Král J, Heneberg P. Integrative taxonomy and evolutionary history of a newly revealed spider Dysdera ninnii complex (Araneae: Dysderidae). Zool J Linn Soc. 2014;172:451–74.

32. Ceccarelli FS, Haddad CR, Ramírez MJ. Endosymbiotic Rickettsiales (Alphaproteobacteria) from the spider genus Amaurobioides (Araneae: Anyphaenidae). J Arachnol. 2016;44:251–3.

33. Park E, Poulin R. Torix group Rickettsia are widespread in New Zealand freshwater amphipods: using blocking primers to rescue host COI sequences. BioRxiv. 2020; DOI: 10.1101/2020.05.28.120196

34. Smith MA, Bertrand C, Crosby K, Eveleigh ES, Fernandez-Triana J, Fisher BL, et al. Wolbachia and DNA barcoding insects: Patterns, potential, and problems. PLoS One. 2012;7:e36514.

35. BOLD: Barcode of Life Data System. 2007. https://www.boldsystems.org/ Accessed 2 January 2018.

36. Smith MA, Pilgrim J, Zakharov E V., Dewaard JR, Ratnasingham S. BOLD contaminant pool (3,817 specimens) data. Figshare. 2020. DOI: 10.6084/m9.figshare.12801107

37. Smith MA, Pilgrim J, Zakharov E V., Dewaard JR, Ratnasingham S. BOLD non-contaminant pool (55,366 specimens) data. Barcoding Of Life Data System. 2020. DOI: 10.5883/DS-RICKET

38. Ratnasingham S, Hebert PDN. BOLD: The Barcode of Life Data System. Mol Ecol Notes. 2007;7:355–64.

39. Davison HR, Siozios S. Rickettsia PhyloFlash and Kraken data from arthropod whole genome projects in the Sequence Read Archive. Figshare. 2020. DOI: 10.6084/m9.figshare.12801140

40. Gruber-Vodicka HR, Seah BK, Pruesse E. phyloFlash — Rapid SSU rRNA profiling and targeted assembly from metagenomes. bioRxiv. 2019. DOI: 10.1101/521922

41. Hernández-Triana LM, Prosser SW, Rodríguez-Perez MA, Chaverri LG, Hebert PDN, Ryan Gregory T. Recovery of DNA barcodes from blackfly museum specimens (Diptera: Simuliidae) using primer sets that target a variety of sequence lengths. Mol Ecol Resour. 2014;14:508–18.

42. Wang Z, Wu M. An integrated phylogenomic approach toward pinpointing the origin of mitochondria. Sci Rep. 2015;5:7949.

43. Goodacre SL, Martin OY, Thomas CFG, Hewitt GM. Wolbachia and other endosymbiont infections in spiders. Mol Ecol. 2006;15:517–27.

44. Martin OY, Puniamoorthy N, Gubler A, Wimmer C, Bernasconi M V. Infections with Wolbachia, Spiroplasma, and Rickettsia in the Dolichopodidae and other Empidoidea. Infect Genet Evol. 2013;13:317–30.

45. Machtelinckx T, Van Leeuwen T, Van De Wiele T, Boon N, De Vos WH, Sanchez J-A, et al. Microbial community of predatory bugs of the genus Macrolophus (Hemiptera: Miridae). BMC Microbiol. 2012;12:S9.

46. Plantard O, Bouju-Albert A, Malard MA, Hermouet A, Capron G, Verheyden H. Detection of Wolbachia in the tick Ixodes ricinus is due to the presence of the hymenoptera endoparasitoid Ixodiphagus hookeri. PLoS One. 2012;7:e30692.

47. Le Clec'h W, Chevalier FD, Genty L, Bertaux J, Bouchon D, Sicard M. Cannibalism and predation as paths for horizontal passage of Wolbachia between terrestrial isopods.

48. Ramage T, Martins-Simoes P, Mialdea G, Allemand R, Duplouy A, Rousse P, et al. A DNA barcode-based survey of terrestrial arthropods in the Society Islands of French Polynesia: host diversity within the SymbioCode Project. Eur J Taxon. 2017;272.

49. Vavre F, Fleury F, Lepetit D, Fouillet P, Bouletreau M. Phylogenetic evidence for horizontal transmission of Wolbachia in host- parasitoid associations. Mol Biol Evol. 1999;16:1711–23.

50. Ahmed MZ, Li S-J, Xue X, Yin X-J, Ren S-X, Jiggins FM, et al. The intracellular bacterium Wolbachia uses parasitoid wasps as phoretic vectors for efficient horizontal transmission. Hurst G, editor. PLOS Pathog. 2015;11:e1004672.

51. Caspi-Fluger A, Inbar M, Mozes-Daube N, Katzir N, Portnoy V, Belausov E, et al. Horizontal transmission of the insect symbiont Rickettsia is plant-mediated. Proc R Soc B Biol Sci. 2012;279:1791–6.

52. Gonella E, Pajoro M, Marzorati M, Crotti E, Mandrioli M, Pontini M, et al. Plant-mediated interspecific horizontal transmission of an intracellular symbiont in insects. Sci Rep. 2015;5:15811.

53. Li Y-H, Ahmed MZ, Li S-J, Lv N, Shi P-Q, Chen X-S, et al. Plant-mediated horizontal transmission of Rickettsia endosymbiont between different whitefly species. FEMS Microbiol Ecol. 2017;93.

54. Jaenike J, Polak M, Fiskin A, Helou M, Minhas M. Interspecific transmission of endosymbiotic Spiroplasma by mites. Biol Lett. 2007;3:23–5.

55. Morrow JL, Frommer M, Shearman DCA, Riegler M. Tropical tephritid fruit fly community with high incidence of shared Wolbachia strains as platform for horizontal transmission of endosymbionts. Environ Microbiol. 2014;16:3622–37.

56. Goodacre SL, Martin OY, Bonte D, Hutchings L, Woolley C, Ibrahim K, et al. Microbial modification of host long-distance dispersal capacity. BMC Biol. 2009;7:32.

57. Giorgini M, Bernardo U, Monti MM, Nappo AG, Gebiola M. Rickettsia symbionts cause parthenogenetic reproduction in the parasitoid wasp Pnigalio soemius (Hymenoptera: Eulophidae). Appl Environ Microbiol. 2010;76:2589–99.

58. Hagimori T, Abe Y, Date S, Miura K. The first finding of a Rickettsia bacterium associated with parthenogenesis induction among insects. Curr Microbiol. 2006;52:97–101.

59. Li K, Chen H, Jiang J, Li X, Xu J, Ma Y. Diversity of bacteriome associated with Phlebotomus chinensis (Diptera: Psychodidae) sand flies in two wild populations from China. Sci Rep. 2016;6:36406.

60. Reeves WK, Kato CY, Gilchriest T. Pathogen screening and bionomics of Lutzomyia apache (Diptera: Psychodidae) in Wyoming, USA. J Am Mosq Control Assoc. 2008;24:444–7.

61. Song S, Chen C, Yang M, Zhao S, Wang B, Hornok S, et al. Diversity of Rickettsia species in border regions of northwestern China. Parasit Vectors. 2018;11:634.

62. Yan P, Qiu Z, Zhang T, Li Y, Wang W, Li M, et al. Microbial diversity in the tick Argas japonicus (Acari: Argasidae) with a focus on Rickettsia pathogens. Med Vet Entomol. 2019;33:327–35.

63. Floris R, Yurtman AN, Margoni EF, Mignozzi K, Boemo B, Altobelli A, et al. Detection and identification of Rickettsia species in the Northeast of Italy. Vector-Borne Zoonotic Dis. 2008;8:777–82.

64. Potts R, Molina I, Sheele JM, Pietri JE. Molecular detection of Rickettsia infection in field-collected bed bugs. New Microbes New Infect. 2020;34:100646.

65. Parola P, Paddock CD, Raoult D. Tick-borne Rickettsioses around the world: Emerging diseases challenging old concepts. Clin Microbiol Rev. 2005;18:719–56.

66. Hoffmann AA, Ross PA, Rašić G. Wolbachia strains for disease control: ecological and evolutionary considerations. Evol Appl. 2015;8:751–68.

67. Iturbe-Ormaetxe I, Walker T, O' Neill SL. Wolbachia and the biological control of mosquito-borne disease. EMBO Rep. 2011;12:508–18.

68. van den Hurk AF, Hall-Mendelin S, Pyke AT, Frentiu FD, McElroy K, Day A, et al. Impact of Wolbachia on infection with chikungunya and yellow fever viruses in the mosquito vector Aedes aegypti. PLoS Negl Trop Dis. 2012;6.

69. Kliot A, Cilia M, Czosnek H, Ghanim M. Implication of the bacterial endosymbiont Rickettsia spp. in interactions of the whitefly Bemisia tabaci with Tomato yellow leaf curl virus. J Virol. 2014;88:5652–60.

70. Tedeschi R, Visentin C, Alam A, Bosco D. Epidemiology of apple proliferation (AP) in northwestern Italy: evaluation of the frequency of AP-positive psyllids in naturally infected populations of Cacopsylla melanoneura (Homoptera: Psyllidae). Ann Appl Biol.

71. Katoh K, Standley DM. MAFFT Multiple sequence alignment software version 7: Improvements in performance and usability. Mol Biol Evol. 2013;30:772–80.

72. Castresana J. Selection of conserved blocks from multiple alignments for their use in phylogenetic analysis. Mol Biol Evol. 2000;17:540–52.

73. Kalyaanamoorthy S, Minh BQ, Wong TKF, von Haeseler A, Jermiin LS. ModelFinder: fast model selection for accurate phylogenetic estimates. Nat Methods. 2017;14:587–9.

74. Nguyen LT, Schmidt HA, Von Haeseler A, Minh BQ. IQ-TREE: A fast and effective stochastic algorithm for estimating maximum-likelihood phylogenies. Mol Biol Evol. 2015;32:268–74.

75. Hoang DT, Chernomor O, Haeseler A von, Minh BQ, Vinh LS. UFBoot2: Improving the ultrafast bootstrap approximation. Mol Biol Evol. 2017;35:518–22.

76. He Z, Zhang H, Gao S, Lercher MJ, Chen W-H, Hu S. Evolview v2: an online visualization and management tool for customized and annotated phylogenetic trees. Nucleic Acids Res. 2016;44:W236–41.

77. Fredslund J, Schauser L, Madsen LH, Sandal N, Stougaard J. PriFi: using a multiple alignment of related sequences to find primers for amplification of homologs. Nucleic Acids Res. 2005;33:W516–20.

78. Martin DP, Murrell B, Golden M, Khoosal A, Muhire B. RDP4: Detection and analysis of recombination patterns in virus genomes. Virus Evol. 2015;1:1–5.

79. Chernomor O, von Haeseler A, Minh BQ. Terrace aware data structure for phylogenomic inference from supermatrices. Syst Biol. 2016;65:997–1008.

80. Okonechnikov K, Golosova O, Fursov M. Unipro UGENE: a unified bioinformatics toolkit. Bioinformatics. 2012;28:1166–7.

81. Ewing B, Hillier L, Wendl MC, Green P. Base-calling of automated sequencer traces using Phred. I. Accuracy Assessment. Genome Res. 1998;8:175–85.

82. Wood DE, Lu J, Langmead B. Improved metagenomic analysis with Kraken 2. Genome Biol. 2019;20:257.

83. Kölsch G, Synefiaridou D. Shared ancestry of symbionts? Sagrinae and Donaciinae (Coleoptera, Chrysomelidae) harbor similar bacteria. Insects. 2012;3:473–91.

84. Li K, Stanojević M, Stamenković G, Ilić B, Paunović M, Lu M, et al. Insight into diversity of bacteria belonging to the order Rickettsiales in 9 arthropods species collected in Serbia. Sci Rep. 2019;9:18680.

85. Murakami T, Segawa T, Bodington D, Dial R, Takeuchi N, Kohshima S, et al. Census of bacterial microbiota associated with the glacier ice worm Mesenchytraeus solifugus. FEMS Microbiol Ecol. 2015;91.

86. Wang H, Lei T, Wang X, Maruthi MN, Zhu D, Cameron SL, et al. A newly recorded Rickettsia of the Torix group is a recent intruder and an endosymbiont in the whitefly Bemisia tabaci. Environ Microbiol. 2020;22:1207–21.

87. Noda H, Watanabe K, Kawai S, Yukuhiro F, Miyoshi T, Tomizawa M, et al. Bacteriome-associated endosymbionts of the green rice leafhopper Nephotettix cincticeps (Hemiptera: Cicadellidae). Appl Entomol Zool. 2012;47:217–25.

88. Zheng Z, Wang D, He H, Wei C. Bacterial diversity of bacteriomes and organs of reproductive, digestive and excretory systems in two cicada species (Hemiptera: Cicadidae). Munderloh UG, editor. PLoS One. 2017;12:e0175903.

89. Kobiałka M, Michalik A, Świerczewski D, Szklarzewicz T. Complex symbiotic systems of two treehopper species: Centrotus cornutus (Linnaeus, 1758) and Gargara genistae (Fabricius, 1775) (Hemiptera: Cicadomorpha: Membracoidea: Membracidae). Protoplasma. 2020;257:819–31.

90. Zouache K, Voronin D, Tran-Van V, Mavingui P. Composition of bacterial communities associated with natural and laboratory populations of Asobara tabida infected with Wolbachia. Appl Environ Microbiol. 2009;75:3755–64.

91. Gualtieri L, Nugnes F, Nappo AG, Gebiola M, Bernardo U. Life inside a gall: closeness does not favour horizontal transmission of Rickettsia between a gall wasp and its parasitoid. FEMS Microbiol Ecol. 2017;93.

92. Gerth M, Wolf R, Bleidorn C, Richter J, Sontowski R, Unrein J, et al. Green lacewings (Neuroptera: Chrysopidae) are commonly associated with a diversity of Rickettsial endosymbionts. Zool Lett. 2017;3:12.

93. Perotti MA, Clarke HK, Turner BD, Braig HR. Rickettsia as obligate and mycetomic bacteria. FASEB J. 2006;20:2372–4.

Figure Legends

Figure 1. Workflow of the BOLD project demonstrating the acquisition and fates of contaminant and non-contaminant *COI* barcoding sequences.

Figure 2. Cladogram of the maximum likelihood (ML) tree of 1,126 proteobacteria *COI* contaminants retrieved from a BOLD project incorporating 184,585 arthropod specimens. The tree is based on 561 bp and is rooted with the free-living alphaproteobacteria *Pelagibacter ubique*. Parantheses indicate the number of BOLD contaminants present in each group. Tips are labelled by BOLD processing ID and host arthropod taxonomy. No colour=Non-BOLD Reference. The Rickettsiales sequences of *Anaplasma*, *Neorickettsia*, *Rickettsia* and *Wolbachia* supergroups (A, B, E, F and H) are included as references (Accession numbers: Additional file 8).

Figure 3. Cladogram of a maximum likelihood (ML) tree of 753 *COI Rickettsia* contaminants retrieved from a BOLD project incorporating 184,585 arthropod specimens. The tree is based on 561 bp and is rooted by the *Rickettsia* endosymbiont of *Ichthyophthirius multifiliis* (Candidatus Megaira) using the TVM+F+I+G4 model. Parantheses indicate the number of BOLD contaminants present in Torix and non-Torix *Rickettsia* groups. Tips are labelled by BOLD processing ID and host arthropod taxonomy. No colour=Non-BOLD reference sequence unless designated by a circle (Dermaptera), star (Diplopoda), triangle (Thysanoptera). The *Rickettsia* groups: Spotted fever, Transitional, Belli, Typhus, Rhyzobius and Torix are included as references (Accession numbers: Additional file 8).

Figure 4. Phylogram of the maximum likelihood (ML) tree of 99 *COI Rickettsia* contaminants (prefix "BIOUG") used for further phylogenetic analysis and 53 Non-BOLD reference profiles (Accession numbers: Additional file 8). The tree is based on the concatenation of 4 loci; *16S rRNA*, *17KDa*, *gltA* and *COI* under a partition model, with profiles containing at least 3 out of 4 sites included in the tree (2,834 bp total) and is rooted by *Rickettsia* endosymbiont of *Ichthyophthirius multifiliis* (Candidatus Megaira). Tips are labelled by host arthropod taxonomy.

Figure 5. *16S rRNA* and *gltA* concatenated maximum likelihood (ML) phylogram (1,834 bp total) including *Rickettsia* hosts from SRA (Triangles) and targeted screens (Stars). The TIM3+F+R2 (16S) and K3Pu+F+G4 (gltA) models were chosen as best fitting models. Rooting is with *Orientia tsutsugamushi*. Accession numbers found in Additional file 8.

Figure 6. Phylogram of a maximum likelihood (ML) tree of *COI Rickettsia* contaminants (prefix “BIOUG”) giving a host barcode and 43 Non-BOLD reference profiles. The tree is based on 4 loci; *16S rRNA*, *17KDa*, *gltA* and *COI* under a partition model with profiles containing at least 2 out of 4 sites included in the tree (2,781 bp total) and is rooted by the *Rickettsia* endosymbiont of *Ichthyophthirius multifiliis* (Candidatus Megaira). The habitats and lifestyles of the host are given to the right of the phylogeny. Accession numbers found in Additional file 8.

Additional file information

Additional file 1.png Collection sites of the 753 *COI Rickettsia* contaminants retrieved from BOLD projects.

Additional file 2.docx Primer pairs involved in the unintended amplification of 753 *Rickettsia COI* from BOLD project.

Additional file 3.docx Homology of *Rickettsia* groups and *Wolbachia* to the most common forward primers (C_LepFolF and C_LepFolR) attributed to bacterial *COI* amplification from arthropod DNA extracts.

Additional file 4.xlsx Re-barcoding status and nearest BLAST hit of mtDNA *COI* arthropod DNA extracts accessed for further analysis, along with the success of multilocus *Rickettsia* profiles with allocated *Rickettsia* group (based on phylogenetic analysis) and co-infection status.

Additional file 5.docx The barcoding success rate of taxa which gave at least one bacteria *COI* inadvertent amplification (N=51,475 accessible specimens) with an adjusted *Rickettsia* prevalence based on an estimated total number of arthropods to account for inaccessible specimens (N=184,585).

Additional file 6.docx GenBank matches mistaken for true mtDNA barcodes and their homology to *Rickettsia COI* (Accessed 29th June 2020).

Additional file 7.pdf Phylogram of a maximum likelihood (ML) tree of *COI Rickettsia* found in the GenBank database erroneously identified as mtDNA barcodes based on 577 bp. The HKY+F+G4 model was chosen as the best fitting model using Modelfinder with the Bayesian information criterion (BIC).

Additional file 8.xlsx Accession numbers used for phylogenetic analyses (Figures 2, 3, 4 ,5 and 6). Accession numbers generated in this study are marked in BOLD.

Additional file 9.docx Mitochondrial *COI* and bacterial gene primers used for re-barcoding and multilocus phylogenetic analyses.

Figure 1

Click here to access/download;Figure;Figure 1.tif

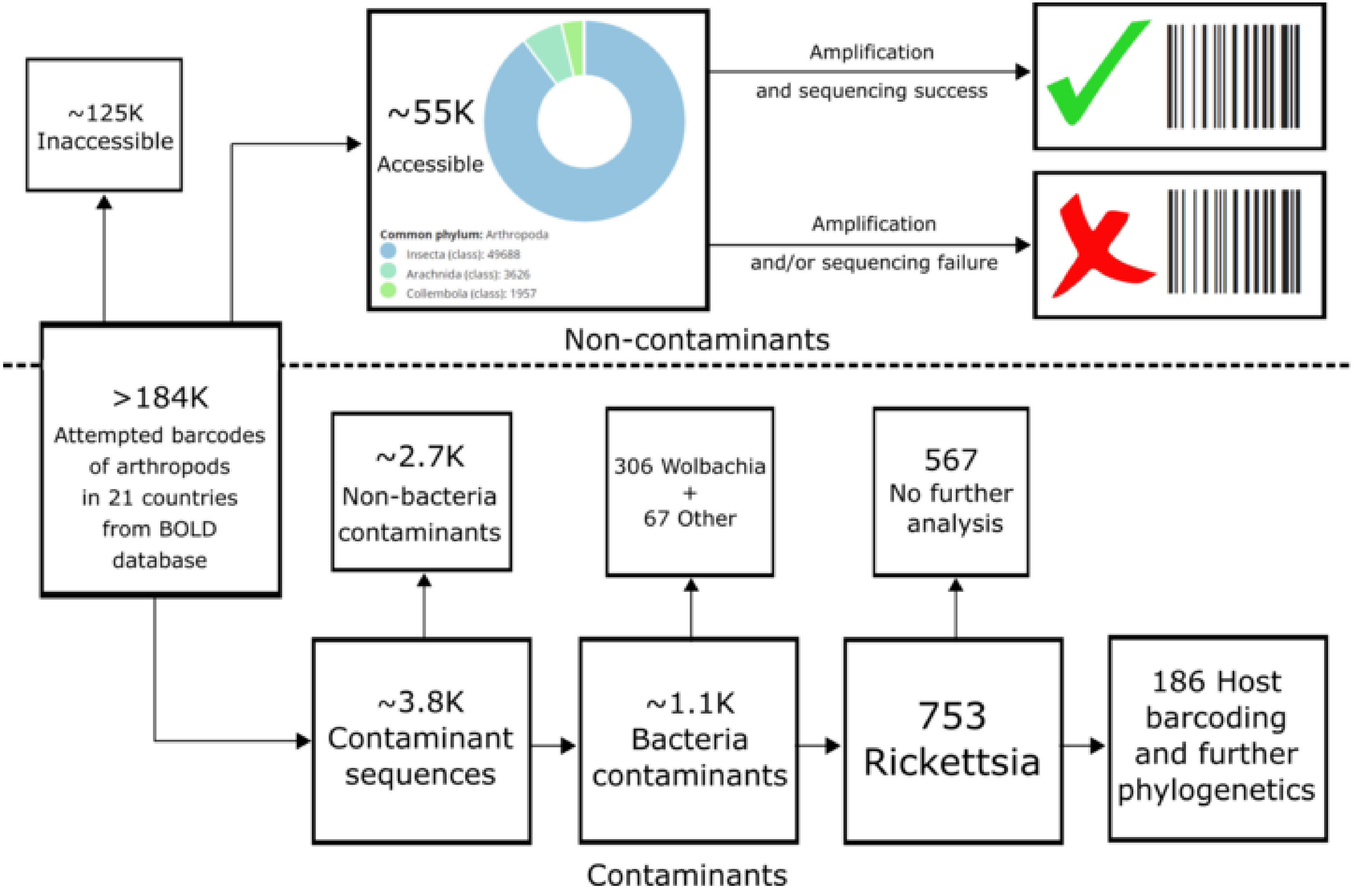

Figure 2

Click here to access/download;Figure;Figure 2.png

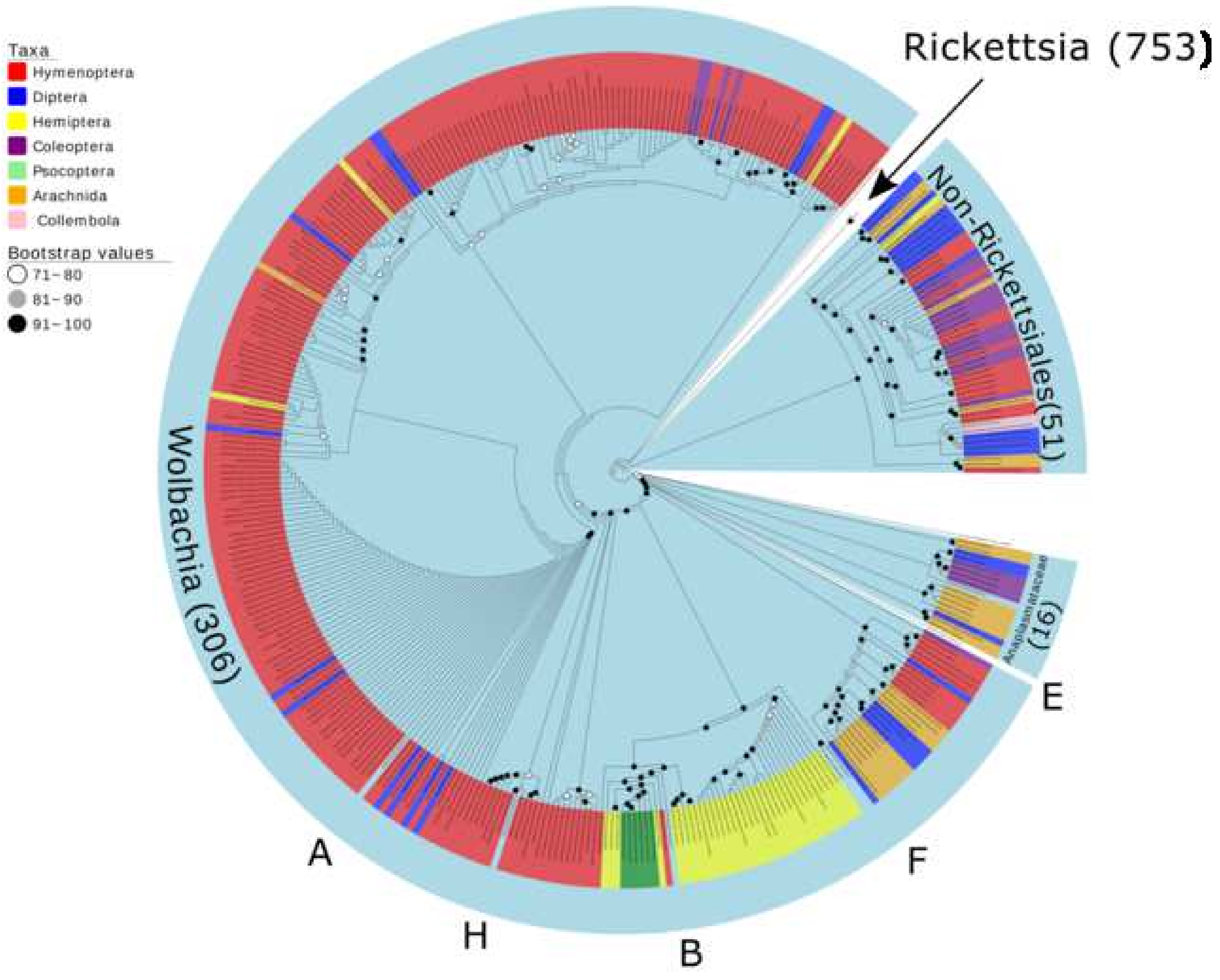

Figure 3

Click here to access/download;Figure;Figure 3.png

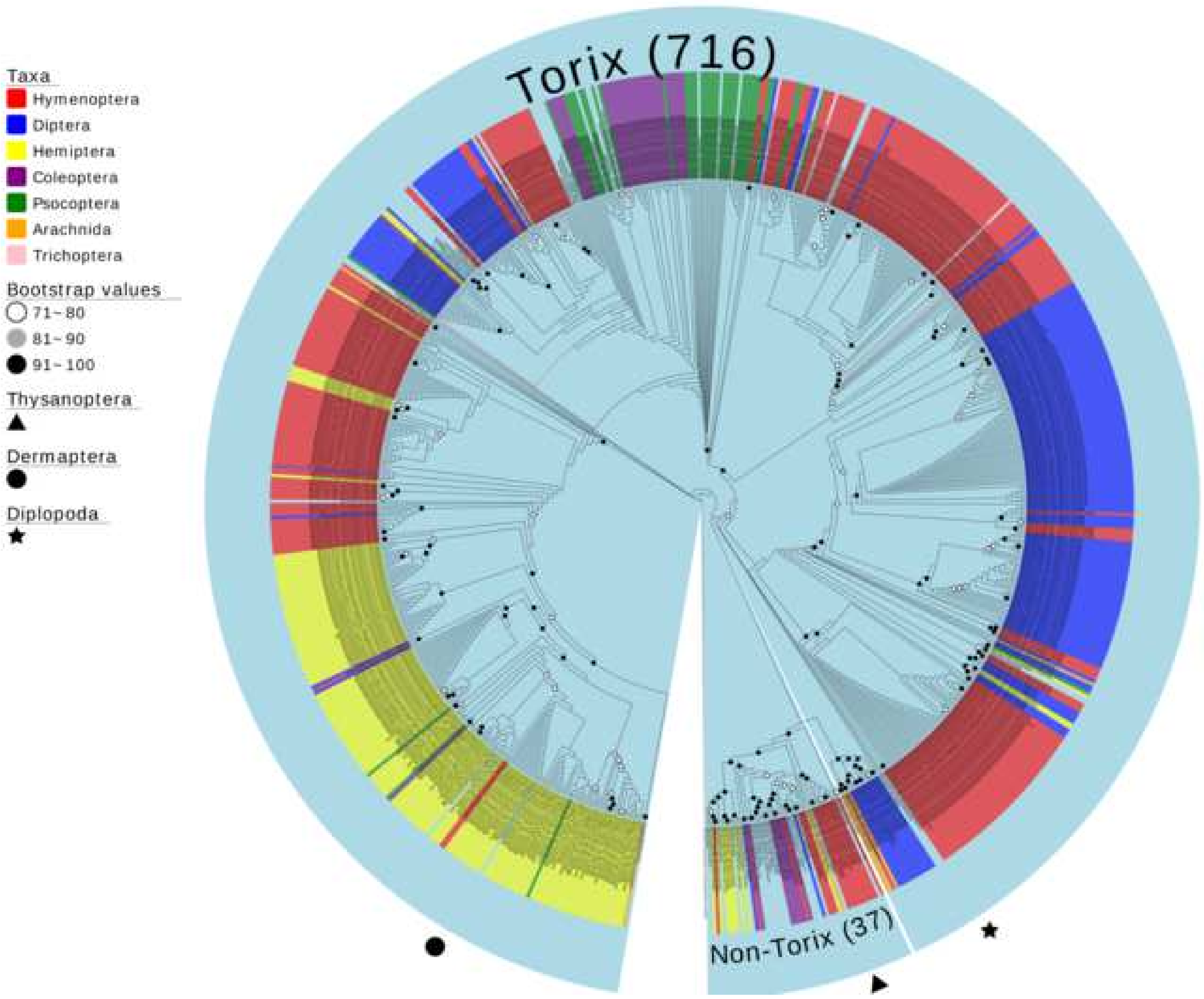

Figure 4

Click here to access/download;Figure;Figure 4.png

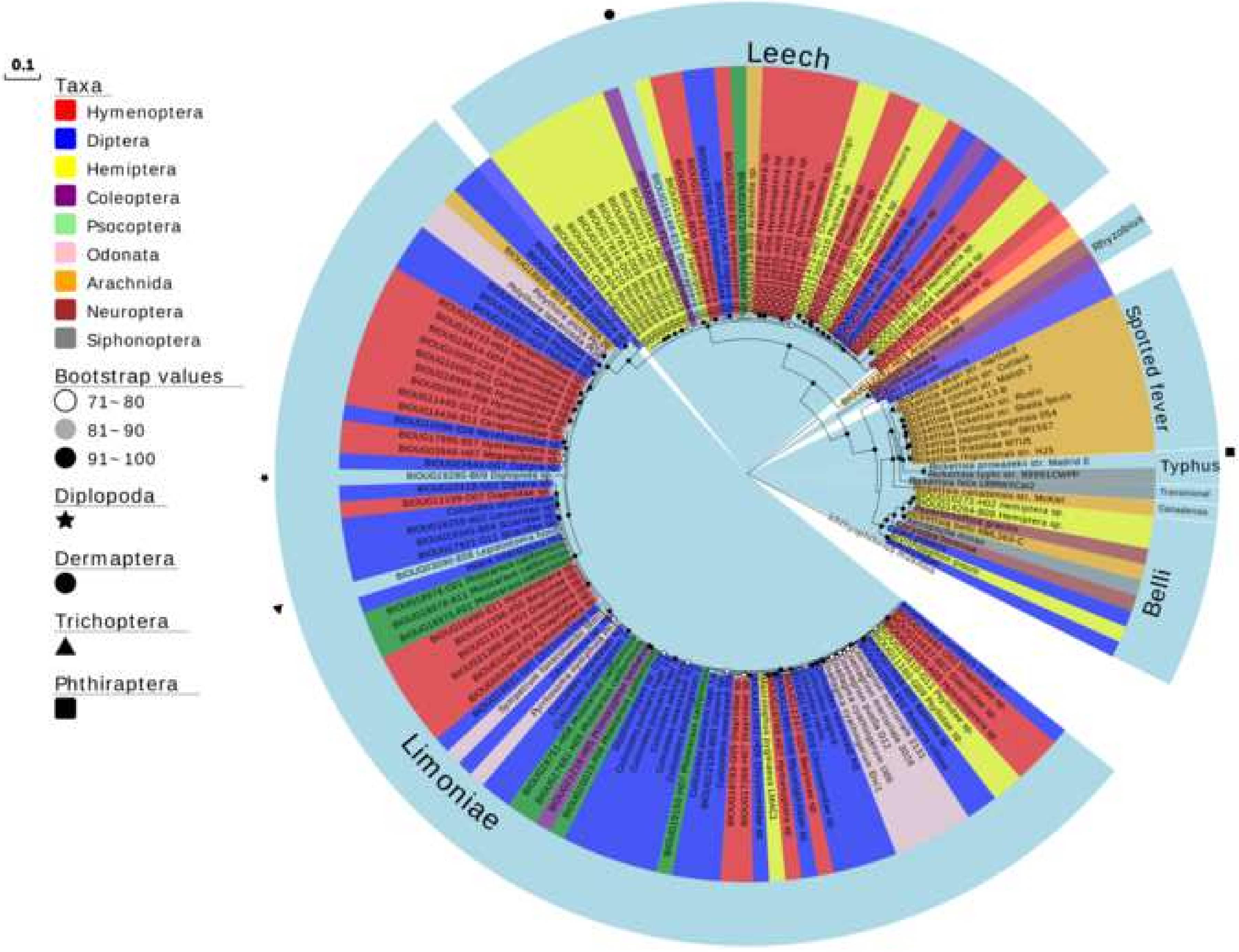

Figure 5

Click here to access/download;Figure;Figure 5.png

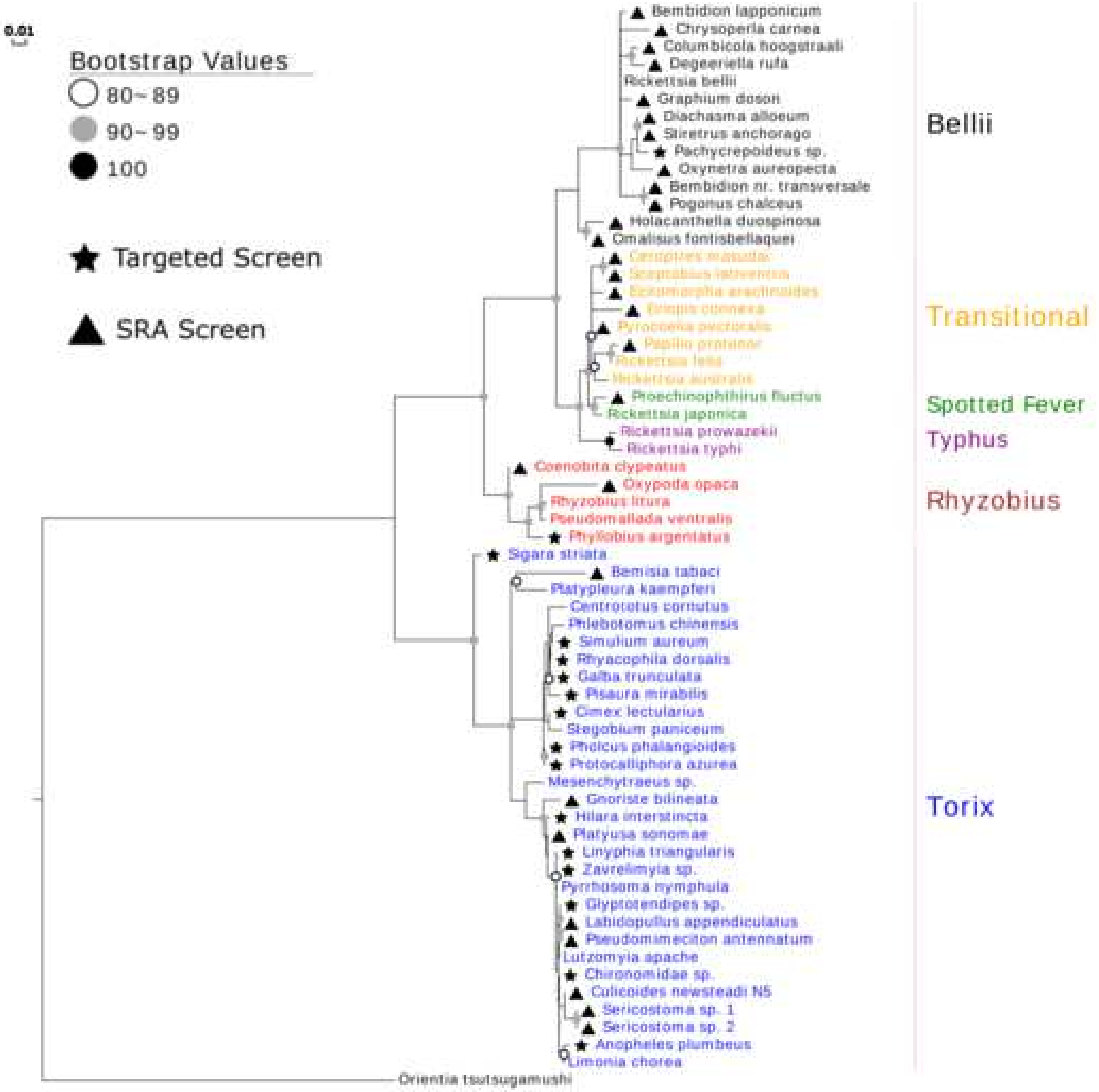

Figure 6 Click here to access/download;Figure;Figure 6.png

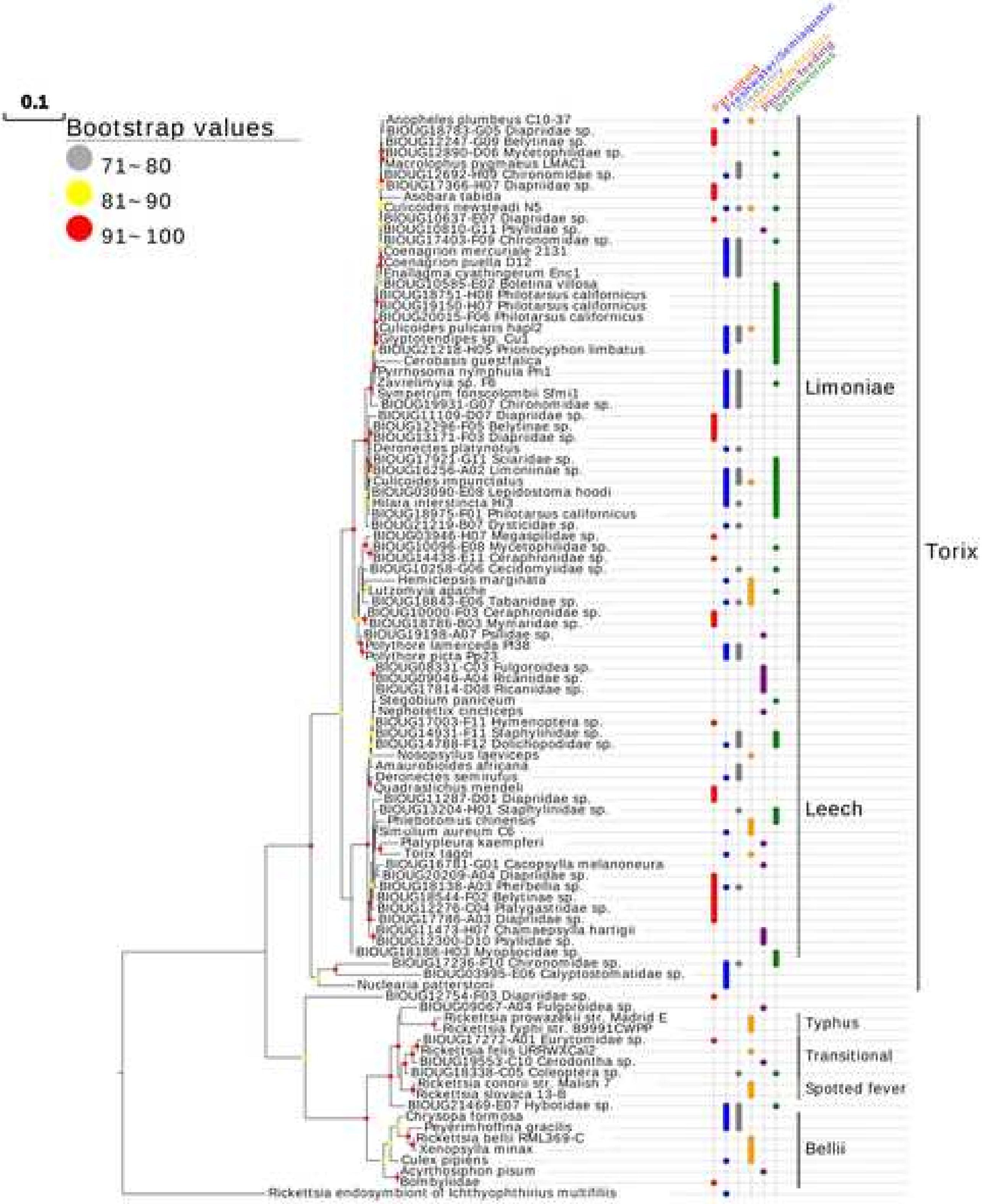

Additional file 1

Click here to access/download
Supplementary Material
Additional file 1.png

Additional file 2

Click here to access/download
Supplementary Material
Additional file 2.docx

Additional file 3

Click here to access/download
Supplementary Material
Additional file 3.docx

Additional file 4

Click here to access/download
Supplementary Material
Additional file 4.xlsx

Additional file 5

Click here to access/download

Supplementary Material

Additional file 5.docx

Additional file 6

Click here to access/download
Supplementary Material
Additional file 6.docx

Additional file 7

Click here to access/download
Supplementary Material
Additional file 7.pdf

Additional file 8

Click here to access/download
Supplementary Material
Additional file 8.xlsx

Additional file 9

Click here to access/download
Supplementary Material
Additional file 9.docx
