# Supplementary material for: Torix Rickettsia are widespread in arthropods and reflect a neglected symbiosis
Source: Gigascience. 2021 Mar 25;10(3):giab021. doi: 10.1093/gigascience/giab021 (PMC7992394; doi:10.1093/gigascience/giab021)
Supplement: giab021_GIGA-D-20-00244_Revision_1 [file giab021_giga-d-20-00244_revision_1.pdf]

GigaScience

Torix Rickettsia are widespread in arthropods and reflect a neglected symbiosis

--Manuscript Draft--

**Manuscript Number:**	GIGA-D-20-00244R1
**Full Title:**	Torix Rickettsia are widespread in arthropods and reflect a neglected symbiosis
**Article Type:**	Research
**Funding Information:**	Biotechnology and Biological Sciences Research Council (BB/M011186/1) — Dr. Jack Pilgrim<br>Natural Environment Research Council (NE/L002450/1) — Ms. Helen R. Davison
**Abstract:**	Background<br><br>Rickettsia are intracellular bacteria best known as the causative agents of human and animal diseases. Although these medically important Rickettsia are often transmitted via haematophagous arthropods, other Rickettsia , such as those in the Torix group, appear to reside exclusively in invertebrates and protists with no secondary vertebrate host. Importantly, little is known about the diversity or host range of Torix group Rickettsia .<br><br>Results<br><br>This study describes the serendipitous discovery of Rickettsia amplicons in the Barcode of Life Data System (BOLD), a sequence database specifically designed for the curation of mtDNA barcodes. Out of 184,585 barcode sequences analysed, Rickettsia is observed in approximately 0.41% of barcode submissions and is more likely to be found than Wolbachia (0.17%). The Torix group of Rickettsia are shown to account for 95% of all unintended amplifications from the genus. A further targeted PCR screen of 1,612 individuals from 169 terrestrial and aquatic invertebrate species identified mostly Torix strains and supports the ‘aquatic hot spot’ hypothesis for Torix infection. Furthermore, the analysis of 1,341 Sequence Read Archive (SRA) deposits indicates Torix infections represent a significant proportion of all Rickettsia symbioses.<br><br>Conclusions<br><br>This study supports a previous hypothesis which suggests Torix Rickettsia are overrepresented in aquatic insects. In addition, multiple methods reveal further putative hot spots of Torix Rickettsia infection; including in phloem-feeding bugs, parasitoid wasps, spiders, and vectors of disease. The unknown host effects and transmission strategies of these endosymbionts make these newly discovered associations important to inform future directions of investigation involving the understudied Torix Rickettsia .
**Corresponding Author:**	Jack Pilgrim<br>University of Liverpool Institute of Infection Veterinary and Ecological Sciences<br>Neston, Cheshire, Liverpool UNITED KINGDOM
**Corresponding Author Secondary Information:**	
**Corresponding Author's Institution:**	University of Liverpool Institute of Infection Veterinary and Ecological Sciences
**Corresponding Author's Secondary Institution:**	
**First Author:**	Jack Pilgrim
**First Author Secondary Information:**	
**Order of Authors:**	Jack Pilgrim<br>Panupong Thongprem<br>Helen R. Davison

Powered by Editorial Manager® and ProduXion Manager® from Aries Systems Corporation

	Stefanos Siozios
	Matthew Baylis
	Evgeny V. Zakharov
	Sujeevan Ratnasingham
	Jeremy R. deWaard
	Craig R. Macadam
	M. Alex Smith
	Gregory D. D. Hurst
**Order of Authors Secondary Information:**	
**Response to Reviewers:**	Dear Dr. Edmunds,<br><br>Thank you for considering a revised version of “Torix Rickettsia are widespread in arthropods and reflect a neglected symbiosis”. The authors would like to thank the three reviewers for their time and comments on the manuscript. Please find below a point-by-point response to reviewer comments. Aside from clarificatory points, the major changes to the manuscript include:<br><br>•The attempted retrieval of both parasitoid and protist reads from the SRA datasets to ascertain the likelihood of these taxa being responsible for the Rickettsia positives observed in the study.<br>•The additional analytical step of using the Kaiju bioinformatic tool to confirm COI sequences from the BOLD dataset as being bacterial.<br>•A more detailed analysis of comparing the presence of Torix Rickettsia in aquatic and terrestrial biomes.<br>•The inclusion of phylograms for figures 2 and 3 to avoid confusion over long branch attractions.<br><br><br>Reviewer#1<br>My only concern is that Torix group Rickettsia and their relatives have also been identified in protists, such as nucleariid amoebae. So I wonder how many of these Rickettsia, particularly in aquatic hosts, are symbionts of protists residing in animal guts. Have the authors tried to pull out protist 18S sequences from the SRA datasets (or tried to amplify protist genes via PCR, although that would be much more difficult)?<br><br>We thank the reviewer for this insight which we agree with. phlyoFLash analysis retrieved 16S (microbe) and 18S (eukaryote) sequences for each SRA dataset where present, and we have now included this information on the FTP server under the directory name “phyloFlash html files”. One instance of an assembled parasitoid 18S rRNA sequence was found in dataset ID SRR6313831 from Bemisia tabaci. However, a B. tabaci-Rickettsia true endosymbiosis has already been confirmed though FISH imaging (Wang et al. 2020; doi:10.1111/1462-2920.14927) suggesting the parasitoid is likely not responsible for the presence of Rickettsia in this case.<br><br>Protist sequences were also identified in some of the SRA datasets but these were a significant minority of reads compared to Rickettsia reads (doi:10.6084/m9.figshare.12801140). Intriguingly, one of the highest numbers of protist reads came from our previous study (SRA dataset SRR5298327) which was shown by FISH to be a true endosymbiosis between insect and Rickettsia (Pilgrim et al. 2017; doi:10.1111/1462-2920.13887). Overall, these data suggest that detecting contamination from Rickettsia-infected protists or parasitoids is uncommon. This new information has been added on lines 274-281, 355-364 and 576-578.<br><br><br>Minor comments:<br><br>Line 194 - Psyllidae spelling<br><br>Line 242 & Table 2 - Chaoboridae spelling

Powered by Editorial Manager® and ProduXion Manager® from Aries Systems Corporation

Line 251 - Simulium spelling

Spellings of these taxa have been now rectified.

Lines 340 - I would replace refs 49 and 50 with Gehrer & Vorburger, Biol. Lett., 2012

The references have now been changed per the reviewer's suggestion.

Line 362 - this sentence is confusing because the citations refer to Rickettsia in the belli group

For clarity the sentence has been changed to specify the references refer to the belli group only (line 417).

Table 2 - Siphonaptera spelling

Line 819 - Parentheses spelling

These spellings have now been changed.

Reviewer#2
Abstract 38, 42-43: the introduction of the "aquatic hotspot" hypothesis and that the results were supporting this hypothesis was very appealing (l38), yet this was not addressed in the conclusion, which instead claimed that Rickettsia was associated with a number of habits (l42-43). As these habits were not linked to aquatic, and not introduced previously in the background, the logic flow here is rather difficult to follow.

We thank the reviewer for flagging this. We have now changed the conclusion of the abstract to show that new hotspots of infection were revealed as well as confirming a bias towards aquatic insects (lines 44-47).

69: Rickettsia has been estimated as being present in 20-24% of species. One would be very interested in learning whether this is confirmed/disapproved by the findings of the current study. Which part of the experimental design is set to answer this question? If no, what needs to be done to get a better idea?

The 20-42% prevalence figure for terrestrial arthropod species is derived from model-based estimation techniques which assume populations infected have a minimum of 1/1000 individuals infected. Thus, our figure of ~9% from the targeted PCR screen is likely lower due to small within-species sample sizes. This has been highlighted in lines 366-370.

79-88: It might be a good idea to add something here about the diversity of subgroups of Torix. The results later on revealed two subgroups (Leech and Limoniae), but are these good representatives of the diversity within Torix? How many subgroups are already known?

Previous studies on Torix Rickettsia have highlighted two subgroups: "Leech" and "Limoniae". This was initially based on limited phylogenetic markers but by extension of using multiple markers we confirm in this study that a majority of Torix strains fall into these two subgroups. We have highlighted this on line 85.

90-102: The use of terms Rickettsia CoxA, COI, Rickettsia COI are confusing. If Rickettsia CoxA and Rickettsia COI are actually referring to the same Rickettsia gene,

Powered by Editorial Manager® and ProduXion Manager® from Aries Systems Corporation

	the term needs to be standardized.<br><br>We thank the reviewer for making this point. We agree that terms should be standardised as much as possible. Therefore, we have removed any reference to 'CoxA' in the manuscript.<br><br>106: does the "template" here refer to DNA extract/aliquot? "Template" in the context of DNA template is primarily used in the description of amplification reaction, which doesn't seem to be the case here. This term is somewhat confusing. As you used "DNA extract" later in the text, I would suggest that these terms be unified.<br><br>The term "template" has been swapped for "DNA extract" throughout the manuscript.<br><br>109: "function more broadly" here is also vague. Do you mean that the primers used in these PCR assays are more degenerate or specifically designed to target Rickettsia genes? Please clarify.<br><br>The primers function more broadly as they were designed from our previous work based on Rickettsia genomes from multiple clades, including the first available Torix genome. This information has been removed from the introduction and is instead clarified in the data description (lines 153-155) and methods (lines 478-480).<br><br>123-125: "...deemed as contaminant sequences as a result of not matching initial morphotaxa assignment". I don't think that this is entirely accurate. A significant proportion of barcodes in BOLD are not matching initial morphotaxa assignment, at varied taxonomic levels. These include mis-identification, ambiguous/unstable taxonomic status, lab contaminations, etc. I would assume that BOLD uses an algorithm to confirm the sequence as being contaminants, only when they are matched to the most common non-target contaminants, e.g., bacteria, human etc.<br><br>We thank the reviewer for their comment. Yes, this dataset contained both contaminant sequences, as well as misidentified taxa and we have now changed the wording of this sentence to reflect this on line 130-132 and in Figure 1. Information on how contaminants were confirmed as bacterial are also now described in lines 450-465.<br><br>125-128: the term "specimens" needs to be clarified. Do these include those that didn't yield a DNA sequence?<br><br>Yes-this included some specimens where barcoding had failed to yield a DNA sequence. This has now been clarified on line 126.<br><br>142: Explain targeted PCR Rickettsia screen. Does it employ specific primer sets designed for Rickettsia? Although this was described in the method section, a brief explaining of the method would help the readers to understand the context.<br><br>Yes, as mentioned above, the primers function more broadly as they were designed from our previous work based on Rickettsia genomes from multiple clades and including the first available Torix genome. This has now been clarified in lines 153-155 and 478-480.<br><br>149: Should "Analyses" be "Results"?<br><br>The formatting of gigaScience uses "analyses" in place of "results".<br><br>160-161: "further unique bacteria contaminants were also detected", where are these results? Please cite.<br><br>These results have now been added in Additional file 1 (graphic representation of taxonomic classification as bacteria) and the FTP server file "Kaiju_misc_bacteria_detection" (sequence information). These were sequences flagged as bacterial by the bioinformatics tool Kaiju (lines 173-176).<br><br>167-170 : if the BOLD results does not seem to support the aquatic hotspot theory, why?

Powered by Editorial Manager® and ProduXion Manager® from Aries Systems Corporation

Both the BOLD and SRA datasets have inherent biases which make them unsuitable to assess whether Torix Rickettsia are more common in aquatic or terrestrial biomes. For example, most SRA submissions are from lab-reared terrestrial insects. Likewise, a majority of the specimens from BOLD containing Rickettsia have limited taxonomic/ecological information, by virtue of not returning an mtDNA COI sequence. Therefore, a PCR-based study targeting both terrestrial and aquatic taxa was implemented in order to specifically test this 'aquatic hot spot hypothesis' (lines 149-158).

170-172: the predominance report of Rickettsia from Canada seems meaningless, given the strongly biased sampling in BOLD (supplementary Fig. 1)

The authors agree. This has now been removed.

180: this is confusing, does it mean that the Torix sequence is identical to that of C_LepFolR at the 3' end? Or does it have a SNP but different from that of other bacteria?

The Torix sequence has a SNP at the same site as all the other Wolbachia/Rickettsia genomes compared to C_LepFolR at the 3' end. However, all the Wolbachia/Rickettisa genomes assessed apart from the Torix Rickettsia have a SNP at the 3' priming end for C_LepFolF. For clarity, this can be viewed in Additional file 4.

185: How were these 186 Rickettsia-containing samples selected from 753 samples?

These DNA extracts were chosen based on assorted geographic location, host order and diverse phylogenetic placement. This has been clarified on line 196-198.

192: So how many subgroups of Torix are known? How well the findings represent the diversity?

As noted in a previous reply, to date only two subgroups of Torix Rickettsia have been uncovered: "Leech" and "Limoniae". This was initially based on limited phylogenetic markers but by extension of using multiple markers we confirm in this study that a majority of Torix strains fall into these two subgroups. We have highlighted this on line 85.

207: define attempted barcodes

In this context, an "attempted barcode" is an attempt to retrieve a mtDNA COI barcode from the approximately 185,000 arthropods in the study. As mentioned above and indicated in figure 1, not all DNA extracts produced a COI sequence to interpret. Now that the term "specimen" has been clarified on line 126 we have replaced "attempted barcodes" with "specimens" to avoid confusion.

211: Here you used "genomic extracts", is this equivalent to "template"? Try to standardize terms.

We have standardised terms to only "DNA extracts" throughout the manuscript.

217: again, why BOLD taxa with the most presence of Rickettsia NOT associated with aquatic lifestyle?
233-235: why did the comparison between aquatic/terrestrial arthropods only consider the targeted Rickettsia screen results, NOT that of SRA search?

We refer the reviewer back to our earlier response (167-170) to address both of these points.

269-270: This is somewhat misleading. This might imply that these two groups of bacteria cooccur in the same organisms, and the amplification of R is easier than W. I don't think the current experimental design is able to proof or deny this possibility.

The wording has now been changed on lines 310-312 to avoid this confusion.

Powered by Editorial Manager® and ProduXion Manager® from Aries Systems Corporation

308-310: we know that there are many other possibilities that might cause barcoding failure. At least provide some alternative causes to avoid biased argument.

We have deleted this argument from the paragraph.

415-416: what are the exact criteria when choosing these DNA templates?

This point has been addressed above (reviewer comment 185)

428: does "linear" mean non-recombined sequence?

In this context, “linear” refers to a parameter of the recombination detection program which refers to the sequences not being circular.

438-439: does this mean that the hosts were NOT identifiable by morphology?

That is correct, the metadata provided for specimens before barcoding is a general morphological classification usually down to the order level. Subsequently, more refined classification can only be achieved from the mtDNA barcode. This has been highlighted on lines 501-504.

459-461: What if the sequence was matched to more than one barcode at >98% identity?

This did not occur.

489-497: Please provide more details on the analysis of phyloFlash, e.g., parameters used. I am a bit concerned about the assembling process employed here. 16S assembling can be difficult/impossible when metagenomics data contain more than 1 bacterial species or multiple variable copies of 16S, both of which might be the case for Rickettsia.

Default parameters were used for phyloFlash (lines 567-578). Phyloflash uses a combination of SPAdes and BBmap to assemble rRNA SSU and references a curated database (SILVA). BBmap cut off for identification is a minimum identity >70% and phyloflash recommends SPAdes as the best method for cases where there may be a lack of close relatives in the reference database. The recent paper (Gruber-Vodika et al. 2020; doi:10.1128/mSystems.00920-20) goes into further details about chimeras, false positives and dataset preparation. While the defaults do what they can to minimise risk of false positives, it cannot be entirely eliminated.

We have attempted to address this by flagging the instances where Wolbachia sequences or other symbionts were also found in the phyloflash notes, though these sequences were not always assembled. This information can be seen in the phyloflash html files on the FTP server.

Table 1: for species without a definite identification to the species level (e.g., Pachycrepoideus sp.), do we know that all specimens analyzed here actually belong to the same species? I assume this can be confirmed using barcodes.

Some arthropods without a definite identification were referred to as “sp.” because barcoding was not successful or did not match any known species in the database (lines 546-547).

Figure legends for Figs. 2 and 3: the term "No colour" is misleading. I thought these would refer to those without any background colors (e.g., Rickettsia lineage in Fig. 2).

We have removed the term “no colour” from the legend.

Fig. 2: So all Rickettsia in this tree were not from non-BOLD reference (says the Fig legend)? If the number in parenthesis represent the number of sequences, why is there only a single tip for Rickettsia? Are they collapsed? If yes, does it mean that the genetic divergence within Rickettsia is much smaller than that within Wolbachia?

Powered by Editorial Manager® and ProduXion Manager® from Aries Systems Corporation

Yes, Rickettsia is collapsed and this is now mentioned in the legend (Line 890). Genetic divergence of Rickettsia is deliberately shown in Figure 3 (and Additional file 2) and not in Figure 2 for ease of presentation, due to the number of taxa in the phylogenies.

Fig. 5: Is the lineage distribution associated with methodology used in discovering these sequences (SRA vs. targeted PCR screening)? Provide statistics.

The SRA datasets contain more Belli strains than the targeted screen but this seems irrelevant information as both datasets cannot be reasonably compared. As mentioned above, the SRA dataset contain very few aquatic insects with most depositions deriving from terrestrial insects and/or lab cultivated insects. In contrast, the targeted screen represents mostly wild-caught insects with a mixture of aquatic and terrestrial arthropods. Subsequently, even if it was shown that specific lineages were associated with the two methods for the SRA and targeted screens, it is just a likely that this is due to sampling bias rather than other methodological biases. Thus, our conclusions are measured

1)The BOLD screen demonstrates that Rickettsia (specifically from the Torix group) are overrepresented in barcoding projects and can help identify new hosts.

2)The SRA screen demonstrates that both Torix and Belli clades of Rickettsia are common.

3)The targeted screen provides evidence to suggest Torix Rickettsia are more common in aquatic insects.

Fig. 6: Move the vertical bars representing Typhus, Transitional, Spotted fever, and Bellii, further to the right so that they are in line with that of Torix. My understanding is that these lineages belong to the same hierarchic level under Rickettsia.

We thank the reviewer for pointing this out and have changed figure 6 accordingly.

Reviewer #3

This study relies heavily on secondary data usage, identifying the presence of Rickettisa symbionts in host samples using discarded data from the BOLD database. This is great, and we should have more studies like this. However, largely, the authors fail to discuss the limitations of their study which comes from secondary data usage. For example, lack of control for cross-contamination of samples, the fact that there may be incomplete taxa sampling, and other biases in the underlying database used. For example, they failed to do a comprehensive analysis looking for batch effects to ensure that samples were not systematically contaminated in data deposited from one organization.

We thank the reviewer for highlighting this. Although this study does use secondary data in the BOLD and SRA screens, our own primary dataset was generated via the targeted screen to prevent an overreliance on secondary data and of course its biases. Regarding the prospect of cross-contamination, this is unlikely for two reasons.

1)A majority of the multilocus profiles assessed from BOLD tend to give unique profiles which is reflected in our phylogenetic trees. Significant cross-contamination would tend to give identical strains.

2)If cross-contamination occurred between DNA extracts then it is likely that an mtDNA COI sequence would be retrieved (either from the original DNA extract or the contaminating one) rather than a Rickettsia COI sequence, as mtDNA is far more likely to amplify than Rickettsia when in competition.

Additionally, due to the aforementioned biases of using secondary data we have tried to be measured in our conclusions as a result of this. Specifically, we are not trying to claim that the Rickettsia sequences discovered in these databases are completely representative of Torix hosts in nature. Merely, that they allow for the discovery of new putative hosts and through combining several methods there is an indication that Torix Rickettsia are more widespread than previously thought and are overrepresented in aquatic insects.

Powered by Editorial Manager® and ProduXion Manager® from Aries Systems Corporation

I also have significant concerns over the lack of detail in the methods and not having access to the multiple sequence alignment used.

Sequence alignments, tree files etc. should already be available to the reviewer via the data management team (in the FTP server) at the journal. If this is not the case, we are happy to reupload the relevant data.

Other concerns/criticisms I had, include:

There are no methods for how samples were binned in Figure 1 either in the manuscript or in the figure. For example, how were bacteria contaminants v. non-bacteria contaminants determined? Was it a BLAST search. If so, what were the criteria? I suspect based on results presented Figures 2 and 3 that the criteria were not stringent enough.

BOLD compares COI sequences to common contaminants (e.g. human, bacteria) using BLAST-details can be found in Ratnasingham and Hebert, 2007 (doi:10.1111/j.1471-8286.2007.01678.x). The designation of bacterial contaminants by BOLD, from the dataset containing 3,817 non-target sequences, was confirmed by the taxonomic classification program, Kaiju, using default parameters. We took the sequences provisionally identified as bacterial before placing them phylogenetically with reference bacteria suggested by Kaiju. This has been highlighted in lines 450-465.

Line 154: Phylogenetic placement does not demonstrate these are of microbial origin. If I put a random sequence into the multiple sequence alignment, it would align and it would be in the phylogeny, by nature of the methods. Nothing about the tree or the topology suggests that didn't happen. In fact, some of the long branches may indicate that it did.

We have now included the usage of Kaiju which is a software program designed to designate taxonomic classification of sequences. For all sequences in the alignment used to create Figure 2, these were all identified as bacteria except one erroneously identified as eukaryotic which was later identified as Rickettsia on our phylogeny. Kaiju also allowed us to choose more specific reference sequences to include in our phylogenies. Aside from Rickettsia and Wolbachia, a significant minority of sequences formed a monophyletic clade with the order Legionellales. In addition, we have now also included mitochondria in the tree on figure 2 to further verify the sequences are bacterial. This is discussed in lines 163-168 and 450-465.

With regards to long branches being problematic, Figures 2 and 3 were constructed as cladograms and not phylograms for neat presentation: branch lengths tell us nothing about clade designation. For transparency we have now included phylograms of figures 2 and 3 in Additional file 2 which demonstrate no long branches.

Since COI is derived from the mitochondrial genome, which is a microbe, language about "microbial origin" needs to be fixed throughout. Many consider organelles to still be microbes. If nothing else, their sequences (including COI) are of microbial origin.

We thank the reviewer for noting this. “Microbial origin” references have now been removed and we now refer to “bacteria” to distinguish from mitochondria throughout the manuscript.

The letters mean in Figure 2 are supposed to be the Wolbachia supergroups. But their placement seems quasi random. The sequences don't appear to be assigned to supergroups. If their placement corresponds to representative sequences, please specify that is the case, and make clear what the representative sequences are, and where they are on the tree.

The supergroup letters are for individual sequences. This has now been noted in the figure 2’s legend with accession details for sequences also clarified as being available in additional file 10.

Regardless, the phylogeny shows issues with very long branches around "A" from

Powered by Editorial Manager® and ProduXion Manager® from Aries Systems Corporation

around 7 o'clock to 9 o'clock if the phylogeny were a 12-hour clock. This is peculiar. Is this an artifact of the tree rendering? Or the outgroup selection? Or some other problem—like the presence of Wolbachia lateral gene transfers that are no longer under selection? Or were sequences included in the analysis that aren't really from bacteria and is an methodological artifact?

As mentioned above, branch lengths do not say anything about genetic distance on cladograms. We have included phylograms in Additional file 2 for transparency and to show a lack of long branches within clades.

In general, there is no discussion or acknowledgement of the extensive literature on bacterial DNA integrations in host genomes, which for Wolbachia is extensive.

This has now been addressed in lines 352-355.

How much support is there for branches/nodes in the tree? I can see bootstrapping in the methods, but I don't see any indication of bootstrap support.

Bootstrapping is present on all trees in this manuscript and graphically represented as black, white and grey circles in figures 2 , 3, 4 and 5 and coloured circles in 6. This is indicated in the top left corner of all figures.

The multiple sequence alignment and unmodified phylogenetic files need to be made available to the reviewers and the readers either as online supplementary material or in a public repository with a permanent DOI.

As mentioned above, all of these files should already be available to reviewers via the FTP server of the journal.

Line 215-227, using the term prevalence is not correct. You do not know the full extent of prevalence of any of these organisms since you weren't targeting them with more specific primers with rigorous sampling. It is easy for this to be misconstrued and alternate terminology is needed.

“Prevalence” has now been changed to “frequency” throughout the manuscript when referring to the proportion of Rickettsia and Wolbachia deposits within the BOLD dataset.

Line 224: "indicating". There are other explanations as well, so I think using the word "suggesting" is more appropriate.

This has now been changed accordingly.

Line 235: The statement is too definitive for the data used. Yes, the stated p-value may be significant, but the statement and conclusions do not take into account the significant sampling bias in the SRA. But in addition, when I do the Fisher's Exact test I get 0.0550, which is not significant. The methods for the Fisher's Exact test and summary of the matrix is missing. My two by two matrix that yields a p-value of 0.0550 used presence/absence in the taxa in the table:

	Aquatic	Terrestrial
Has Torix Rickettsia	9	7
Does not have	49	107

Intuitively it isn't surprising it wouldn't be significant he difference is 20% v. 10% with more limited sampling of one than the other and low levels of detection overall.

We appreciate the reviewer’s diligence in checking the Fisher’s Exact test. However, the matrix presented by the reviewer does not consider Rickettsia subgroup and fails to account for multiple rows containing the same species (be it from a different population).

Subsequently, when taking these factors into account this is the matrix which was used in the submitted manuscript.

Powered by Editorial Manager® and ProduXion Manager® from Aries Systems Corporation

	Aquatic	Terrestrial
Has Torix Rickettsia	9	5
Does not have	49	106

Note that only 5 Torix Rickettsia are present in this matrix for terrestrial species because 2 of the 7 Rickettsia positive strains from the terrestrial species are not from the Torix group.

Since submission of the initial manuscript, table 1 has been updated to reflect previously missing Rickettsia positives detected in 3 spiders. With the addition of these spider positives, there is no significant difference between aquatic taxa and terrestrial taxa ($p=0.1038$).

However, when considering insects alone, this results in a p value of 0.0131. When controlled for taxonomic group (not all insect orders are represented in terrestrial and aquatic pools) the p value is still significant at 0.025. Subsequently, we have now suggested that the aquatic hotspot for Torix Rickettsia appears to apply for insects but not invertebrates in general. It should also be noted that the within-species sample sizes of terrestrial taxa in this study are often greater than aquatic suggesting that p values are conservative (positives are more likely to be found with greater sample sizes).

Details of Fisher's exact analyses have now been included in Additional file 7 and discussed in lines 245-261 and 554-564.

Line 300-301: what was the minimum criteria to say that a taxa has it? Merely a COI sequence? Or more? It seems given cross contamination of sequencing projects and other issues, that you need more than just the COI sequence in the BOLD database. Making it clear here is important to the discussion and interpretation of results.

The issue of cross-contamination has been addressed in our first response to the reviewer. Of course, ideally to confirm a true endosymbiosis, direct visualisation of the symbiont in the host's tissues is needed due to potential for the bacteria to come from ingested food or parasitism. However, previous studies have predominantly relied solely on PCR to identify putative hosts (as demonstrated in Table 2). To reflect this, we have changed the language accordingly to mention "putative hosts" where appropriate (lines 287, 296, 342, 389, 427). Additionally, we direct the reviewer to our response to reviewer 1, where we have screened SRA datasets to assess how likely contamination from ingested biota and parasitism is. Rickettsia-insertions into the host nuclear genome is also unlikely because all protein-coding genes from this study showed no signs of a frameshift, suggesting a lack of pseudogenization. Further, there are no well supported cases of Rickettsia inserts in the nuclear genome in the literature to date, a marked contrast to Wolbachia.

We agree with the reviewer that these points are important for the interpretation of the results and now mention them in lines 337-350

Line 310: I'm not sure I agree with your logic. It might be that they fail because of Rickettsia or other bacterial DNA replication.

This argument has been removed from the paragraph.

Line 329: these conclusions seem premature given the data presented, since bootstrap support values or missing in this version reviewed.

We refer the reviewer to our previous response to bootstrapping.

Please check the legends in the additional files. I think Additional File 3 has a legend stating it is "Additional File 2". Likewise Additional File 2 has a legend stating it is "Additional File 1"

We thank the reviewer for flagging this. We have changed the legends accordingly.

Additional Information:

Powered by Editorial Manager® and ProduXion Manager® from Aries Systems Corporation

Question	Response
Are you submitting this manuscript to a special series or article collection?	No
**Experimental design and statistics**<br><br>Full details of the experimental design and statistical methods used should be given in the Methods section, as detailed in our Minimum Standards Reporting Checklist. Information essential to interpreting the data presented should be made available in the figure legends.<br><br>Have you included all the information requested in your manuscript?	Yes
**Resources**<br><br>A description of all resources used, including antibodies, cell lines, animals and software tools, with enough information to allow them to be uniquely identified, should be included in the Methods section. Authors are strongly encouraged to cite Research Resource Identifiers (RRIDs) for antibodies, model organisms and tools, where possible.<br><br>Have you included the information requested as detailed in our Minimum Standards Reporting Checklist?	Yes
**Availability of data and materials**<br><br>All datasets and code on which the conclusions of the paper rely must be either included in your submission or deposited in publicly available repositories (where available and ethically appropriate), referencing such data using a unique identifier in the references and in the "Availability of Data and Materials" section of your manuscript.	Yes

Powered by Editorial Manager® and ProduXion Manager® from Aries Systems Corporation

Have you have met the above requirement as detailed in our Minimum Standards Reporting Checklist?	

Powered by Editorial Manager® and ProduXion Manager® from Aries Systems Corporation

Manuscript

Click here to access/download;Manuscript;Torix Rickettsia are widespread revised 5 11 20 .docx

Torix *Rickettsia* are widespread in arthropods and reflect a neglected symbiosis

Jack Pilgrim[1*], Panupong Thongprem[1], Helen R. Davison[1], Stefanos Siozios[1], Matthew Baylis[1,2], Evgeny V. Zakharov[3], Sujeevan Ratnasingham[3], Jeremy R. deWaard[3], Craig R. Macadam[4], M. Alex Smith[5], Gregory D. D. Hurst[1]

1. Institute of Infection, Veterinary and Ecological Sciences, Faculty of Health and Life Sciences, University of Liverpool, Liverpool, U.K.

2. Health Protection Research Unit in Emerging and Zoonotic Infections, Liverpool, U.K.

3. Centre for Biodiversity Genomics, University of Guelph, Guelph, Ontario, Canada.

4. Buglife – The Invertebrate Conservation Trust, Balallan House, 24 Allan Park, Stirling, U.K.

5. Department of Integrative Biology, University of Guelph, Guelph, Ontario, Canada.

* Corresponding author

Emails:

JP: jack.pilgrim@liverpool.ac.uk

PT: pongthq@liverpool.ac.uk

HRD: hlhdavi5@liverpool.ac.uk

SS: siozioss@liverpool.ac.uk

MB: baylism@liverpool.ac.uk

EVZ: zakharov@uoguelph.ca

SR: sratnasi@uoguelph.ca

JRD: dewaardj@uoguelph.ca

CRM: craig.macadam@buglife.org.uk

MAS: salex@uoguelph.ca

GDDH: ghurst@liverpool.ac.uk

Abstract

Background: *Rickettsia* are intracellular bacteria best known as the causative agents of human and animal diseases. Although these medically important *Rickettsia* are often transmitted via haematophagous arthropods, other *Rickettsia*, such as those in the Torix group, appear to reside exclusively in invertebrates and protists with no secondary vertebrate host. Importantly, little is known about the diversity or host range of Torix group *Rickettsia*.

Results: This study describes the serendipitous discovery of *Rickettsia* amplicons in the Barcode of Life Data System (BOLD), a sequence database specifically designed for the curation of mtDNA barcodes. Out of 184,585 barcode sequences analysed, *Rickettsia* is observed in approximately 0.41% of barcode submissions and is more likely to be found than *Wolbachia* (0.17%). The Torix group of *Rickettsia* are shown to account for 95% of all unintended amplifications from the genus. A further targeted PCR screen of 1,612 individuals from 169 terrestrial and aquatic invertebrate species identified mostly Torix strains and supports the ‘aquatic hot spot’ hypothesis for Torix infection. Furthermore, the analysis of 1,341 Sequence Read Archive (SRA) deposits indicates Torix infections represent a significant proportion of all *Rickettsia* symbioses.

Conclusions: This study supports a previous hypothesis which suggests Torix *Rickettsia* are overrepresented in aquatic insects. In addition, multiple methods reveal further putative hot spots of Torix *Rickettsia* infection; including in phloem-feeding bugs, parasitoid wasps, spiders, and vectors of disease. The unknown host effects and transmission strategies of these endosymbionts make these newly discovered associations important to inform future directions of investigation involving the understudied Torix *Rickettsia*.

Keywords: Rickettsia; symbiosis: arthropods; endosymbiont; DNA barcoding

Background

It is now widely recognized that animals live in a microbial world, and that many aspects of animal biology, ecology and evolution are a product of their symbioses with microorganisms [1]. In invertebrates, these symbioses may be particularly intimate, and involve transmission of the microbe from parent to offspring [2]. The alignment of host reproduction with symbiont transmission produces a correlation between the fitness interests of the parties, reflected in symbionts evolving to play a number of physiological roles within the host, from defence [3,4] through to core anabolic and digestive functions [5,6]. However, the maternal inheritance of these microbes has led to the retention of parasitic phenotypes associated with distortion of reproduction, with symbiont phenotypes including biases towards daughter production and cytoplasmic incompatibility [7]. These diverse individual impacts alter the ecology and evolution of the host, in terms of diet, dynamics of interaction with natural enemies, sexual selection and speciation.

Heritable symbioses have evolved on multiple occasions amongst microbial taxa. In some cases, the microbial lineage is limited to a single clade of related animal hosts, such as *Buchnera* in aphids [8]. In other cases, particular heritable microbes are found across a wide range of arthropod species. *Wolbachia* represents the most common associate, considered to infect nearly half of all species [9], and this commonness is a function in part of the ability of *Wolbachia* to transfer to a broad range of new host species and spread within them (host shift events) [10]. Aside *Wolbachia*, other microbes are found commonly as heritable symbionts of arthropod hosts [11]. *Cardinium* and *Rickettsia*, for instance, have been estimated at being present in 13-55% and 20-42% of terrestrial arthropod species respectively [12].

In this paper, we address the diversity and commonness of symbioses between *Rickettsia* and arthropods. The *Rickettsia* have increasingly been recognized as a genus of bacteria with diverse interactions with arthropods [13,14]. First discovered as the agents underlying several diseases of humans vectored by haematophagous arthropods [15,16], our understanding of the group changed in the 1990s with the recognition that *Rickettsia* were commonly arthropod symbionts [17,18]. *Rickettsia* were recognized first as male-killing reproductive parasites [17,19] and then later as beneficial partners [3,20,21].

Following this extension of our understanding of *Rickettsia*-arthropod interactions, a new clade of *Rickettsia* was discovered from work in *Torix* leeches [22,23]. This clade was sister to all other *Rickettsia* genera and contained two subgroups (Leech and Limoniae [24]), with no evidence to date of any strain having a vertebrate pathogen phase. The host range for Torix *Rickettsia* is broader than that for other members of the genus, going beyond arthropods to include amoeba hosts [25,26]. Targeted PCR based screening have revealed Torix group *Rickettsia* as particularly common in three groups with aquatic association: *Culicoides* biting midges, deronectid beetles and odonates [24,27,28]. However, some previous hypothesis-free PCR screens that aimed to detect *Rickettsia* in arthropods have likely missed these symbioses, due to divergence of the marker sequence and mismatch with the primers [29].

During our previous work on Torix *Rickettsia* in biting midges [27], we became aware of the presence of *Rickettsia* cytochrome *c* oxidase I (*COI*) sequences deposited in GenBank that derived from studies where the intended target of amplification/sequencing was

mitochondrial *COI*. These deposits derived from studies using mtDNA barcoding for phylogeographic inference [30], or in barcoding based species identification approaches [31,32]. Non-target amplification of *Rickettsia COI* using mitochondrial *COI* barcoding primers has been reported in spiders [31,32] and freshwater amphipods [30,33]. Furthermore, we have noted two cases in our lab where amplicons obtained for mtDNA barcoding of an arthropod have, on sequence analysis, revealed *Rickettsia COI* amplification (Belli group *Rickettsia* from Collembola, and Torix group *Rickettsia* from *Cimex lectularius* bedbugs). Previous work had established barcoding approaches may amplify *COI* from *Wolbachia* symbionts [34], and the data above indicated that non-target *Rickettsia COI* may be likewise amplified during this PCR amplification for mitochondrial *COI*.

In this paper, we use three approaches to reveal the diversity and commonness of Torix *Rickettsia* in arthropods. First, we probed a bin from the Barcode of Life Data System (BOLD [35]), containing non-target *COI* sequences, for *Rickettsia* amplicons and then used the DNA extracts from these projects to define the diversity of *Rickettsia* observed using a multilocus approach. Second, we screened DNA extracts from multiple individuals from 169 invertebrate species for *Rickettsia* presence to determine the distribution of the symbiont in both terrestrial and aquatic biomes. Finally, we used bioinformatic approaches to examine the Sequence Read Archive (SRA) depositions for one individual from 1,341 arthropod species for the presence of *Rickettsia* and used this as a means of estimating the relative balance of Torix group to other *Rickettsia* within symbioses.

Data Description

Barcode of Life Data System (BOLD)

While searching the Barcode of Life Data System (BOLD), a depository of >8 million *COI* mtDNA sequences, hundreds of hits were observed with high sequence similarity to Torix group *Rickettsia*. To investigate the diversity and host distribution of these non-target amplicons, access was permitted to analyse *COI* barcoding data deriving from a BOLD screening project totaling 184,585 arthropod specimens (including individuals where barcoding had failed) from 21 countries and collected between 2010 and 2014. *COI* sequences provided by BOLD were generally derived from DNA extracts created from somatic tissues (legs are often used in order to retain most of the specimen for further analyses if necessary), but also rarely included abdominal tissues. The first dataset made available [36] included 3,817 specimens containing sequences not matching initial morphological assignment (and likely to contain contaminant sequences). The second dataset included 55,366 specimens judged to not contain non-target amplicons [37]. A remaining 125,402 specimens were not made available, and the 55,366 subsample was used as a representative sample from which the contaminants had originated (Figure 1). The protocols for data collection, data curation and quality control of submitted BOLD samples is described by Ratnasingham & Hebert [38].

Sequence Read Archive (SRA)

Further insights into the balance of *Rickettsia* groups within arthropod symbioses were obtained through searching for *Rickettsia* presence in Illumina datasets associated with arthropod whole genome sequence (WGS) projects in the SRA (60,409 records as of the 20th May 2019). To reduce the bias from over-represented laboratory model species (e.g.

Drosophila spp., *Anopheles* spp.) a single dataset per species was examined, and where multiple data sets existed for a species, that with the largest read count was retained. The resultant dataset [39], representing 1,341 arthropod species, was then screened with phyloFlash [40] which finds, extracts and identifies SSU rRNA sequences.

Targeted screen of aquatic and terrestrial arthropods

Both the BOLD and SRA datasets have inherent biases which make them unsuitable to assess whether Torix *Rickettsia* are more common in aquatic or terrestrial biomes. For example, most SRA submissions are from lab-reared terrestrial insects. Likewise, a majority of the BOLD specimens containing *Rickettsia* have limited taxonomic and ecological information, by virtue of not returning an mtDNA *COI* sequence. Therefore, a targeted PCR screen of 1,612 individuals from 169 species was undertaken (Table 1) using primers which hybridise with all known clades of *Rickettsia* [27]. Within this, we included a range of both aquatic and terrestrial taxa, to investigate if the previous work highlighting particular aquatic taxa as hot spots for *Rickettsia* symbiosis (water beetles, biting midges, damselflies) reflects a wider higher incidence in species from this habitat.

Analyses

Torix Rickettsia is the most common bacterial contaminant produced during barcoding projects

Out of 3,817 sequences considered as not matching initial morphological assignment, 1,126 of these were deemed by BOLD to be bacterial in origin (Figure 1, [36]). The taxonomic classification tool, Kaiju, further supported bacterial designation for all sequences except one

(Additional file 1), although this was later confirmed as *Rickettsia* through phylogenetic placement. Phylogenetic placement further confirmed the correct designation of bacterial sequences (Figure 2 and Additional file 2). The dominant genus was *Rickettsia* with 753 (66.9%) amplifications, compared to *Wolbachia* with 306 (27.2%). Of the remaining 67 non-target sequences, 14 formed a monophyletic group with other Anaplasmataceae and 48 clustered with the order Legionellales, with 5 sequences remaining undesignated. When considering the 184,585 specimens in the total project, this analysis gave an overall *Rickettsia* and *Wolbachia* frequency of 0.41% and 0.17% respectively within the dataset. Through later access to the 55,366 representative data subset from where the contaminants originated, a further 245 unique bacteria contaminants were also detected by Kaiju (possibly missed by BOLD's automated contaminant filtering system) (Additional file 1). This additional finding suggests these frequencies are conservative estimates.

BOLD *Rickettsia* contaminants were dominated by amplicons from the Torix group of *Rickettsia* (716/753; 95.1%) (Figure 3 and Additional file 2). The remaining 37 *Rickettsia* clustered with Transitional/Spotted Fever (n=15), Belli (n=9), Rhyzobius (n=1) groups, while 12 sequences formed two unique clades. Across arthropod hosts: 292 (38.8%) were derived from Hymenoptera; 189 (25.1%) from Diptera; 177 from Hemiptera (23.5%); 41 from Psocoptera (5.4%); 40 from Coleoptera (5.3%); 7 from Arachnida (0.9%); 4 from Trichoptera (0.5%); and single cases of Thysanoptera, Diplopoda and Dermaptera (0.1% each).

We observed that two sets of *COI* primers were responsible for 99% of *Rickettsia* amplifications (Additional file 3) with a majority (89%) amplifying with the primer combination

C_LepFolF/C_LepFolR [41]. Torix *Rickettsia COI* showed a stronger match to these primers at the 3' end (the site responsible for efficient primer annealing) compared to *Wolbachia* and other *Rickettsia* groups. Whilst all contained a SNP at the 3' priming end of C_LepFolR, Torix *Rickettsia* (*Rickettsia* endosymbiont of *Culicoides newsteadi*; MWZE00000000) was the only sequence to not contain a SNP at the 3' priming site of C_LepFolF (Additional file 4).

Rickettsia multilocus phylogenetic analysis

To better resolve the phylogenetic relationships between BOLD *Rickettsia* contaminants, a multilocus approach was employed on a subsample of 186 *Rickettsia*-containing samples chosen based on assorted geographic location, host order and phylogenetic placement. To this end, 2 further housekeeping genes (*16S rRNA*, *gltA*) and the antigenic *17KDa* protein gene were amplified and sequenced from the respective DNA extracts.

Overall, 135 extracts successfully amplified and gave a high-quality sequence for at least one gene. No intragenic or intergenic recombination was detected for any of the gene profiles. A phylogram, including 99 multilocus profiles containing at least 3 of the 4 *Rickettsia* genes of interest (including *COI*), allocated strains to both Limoniae and Leech subclades of the Torix group (Figure 4) and these subclades were derived from similar hosts. For example, specific families (Hemiptera: Psyllidae and Hymenoptera: Diapriidae) were present in both Leech and Limoniae groups. A full list of multilocus profiles and *Rickettsia* group designation can be found in Additional file 5.

The multilocus study also provided evidence of co-infection with *Rickettsia*. During Sanger chromatogram analysis, double peaks were occasionally found at third codon sites from protein coding genes. This pattern was observed in 6/10 *Philotarsus californicus* individuals and in one member of each of the Psilidae, Sciaridae, Chironomidae and Diapriidae (Additional file 5). Where double peaks were observed, this was found consistently across markers within an individual specimen. This pattern corroborates a recent finding of double infections in Odoantes [28], suggesting co-infecting *Rickettsia* strains in hosts is a widespread phenomenon of the Torix group.

Barcoding success of Rickettsia host taxa

An available subset of specimens associated with the contaminants contained 55,366 out of 184,585 arthropods originally used in the overall study [37]. The three classes of Insecta (n=49,688), Arachnida (n=3,626) and Collembola (n=1,957), accounted for >99.8% of total specimens (Figure 1). Successful amplification and sequencing of *COI* was achieved in 43,246 specimens (78.1%) of the DNA extracts, but when assessed at the order level success rates varied (Additional file 6). The likely explanation for this variation is taxa-specific divergence of sequences at priming sites.

The number of each taxonomic order giving at least one *Rickettsia* amplification was then calculated and adjusted based on the total number of specimens in the project to allow for a frequency estimate. Overall, Hymenoptera, Diptera and Hemiptera were the three taxa most likely to be associated with *Rickettsia COI* amplification (87.4%). Similarly, on assessment of a subsample from the project where the contaminants originated, a majority (77.7%) of the

dataset were also accounted for by these three orders. After adjusting the frequency to take into account the number of inaccessible specimens, Trichoptera (2.45%), Dermaptera (1.89%) and Psocodea (1.67%) were the most likely taxa to give an inadvertent *Rickettsia* amplification. Whilst Hemiptera and Diptera had a similar estimated frequency of *Rickettsia* amplification (0.58% and 0.56%), Hemiptera were much more likely to fail to barcode (67.2% vs 93.3%), suggesting dipteran *Rickettsia* infection in BOLD specimens is likely to be higher than that of hemipterans, as a barcoding failure is necessary to amplify non-target bacteria *COI*. Attempts to re-barcode 186 *Rickettsia*-containing DNA extracts of interest from BOLD resulted in 90 successful arthropod host barcodes (Additional file 5).

Targeted Rickettsia PCR screen and statistical comparison of terrestrial vs aquatic insects

The screening of aquatic invertebrates revealed 9 out of 57 species (15.79%) were positive in PCR assays (Table 1.1). DNA sequences confirmed that all were *Rickettsia* which lay within the Torix group (Figure 5), with the positive species comprising of 8 insect species and one mollusc. For the terrestrial invertebrates, PCR assays evidenced *Rickettsia* infection in 10 out of 112 species (8.93%) with a mix of insect and spider hosts (4 and 6 species respectively, Table 1.2). *Rickettsia* from 8 host species (2 insects and 6 spiders) were identified as Torix *Rickettsia* (8 of 112 species, 7.14%), while the other two host species carried *Rickettsia* from the Rhyzobius and Belli groups (Figure 5).

To reduce taxonomic hot spot biases (particularly from spiders), we compared the incidence of *Rickettsia* infection in aquatic vs terrestrial insects. Fisher's exact test analysis rejected the null hypothesis of equal representation, with aquatic taxa having a higher representation of

species with Torix *Rickettsia* than terrestrial (*p*-value = 0.013, Additional file 7). Examining the phylogenetically controlled set, with three matched insect orders (Coleoptera, Diptera, Hemiptera), again rejected the null hypothesis of equal representation, with aquatic taxa having a higher representation of species with Torix *Rickettsia* than terrestrial (*p*-value = 0.025, Additional file 7).

[Insert Table 1 here]

SRA and GenBank Rickettsia searches

During the SRA search, phyloFlash flagged 29 *Rickettsia* sequences in the groups: Belli (n=10), Torix (n=8), Transitional (n=6), Rhyzobius (n=2), and Spotted Fever (n=1), with the remaining two failing to form a monophyletic clade with any group (Figure 5). In addition, Kraken identified eight *Rickettsia*-containing arthropod SRA datasets missed by phyloFlash. Two of these were from the Torix group, in phantom midge hosts (Diptera: Chaoboridae: *Mochlonyx cinctipes* and *Chaoborus trivitattus*), with the remaining six placed in Belli and Spotted Fever groups [39].

phyloFLash was also used to retrieve 18S rRNA (eukaryotic) sequences which could potentially account for the *Rickettsia* observed in SRA datasets (e.g. through parasitisms or ingestion of *Rickettsia*-infected protists). Out of the 29 datasets analysed by phyloFlash, only one (SRR6313831) revealed an assembled 18S rRNA sequence aligned to a parasitoid wasp (*Hadrotrichodes waukheon*). Although reads aligned to protists were also present in 19/29 datasets flagged by phyloFlash, the read depth for protists was much lower than the number

of *Rickettsia* reads [39]. This suggests that *Rickettisa*-infected protists are unlikely to account for the positives observed in the SRA datasets.

The search of GenBank revealed 11 deposits ascribed to host mtDNA that were in fact Torix *Rickettsia* sequences (Additional files 8 and 9).

The hidden host diversity of Torix Rickettsia

Overall, putative novel Torix hosts detected from all screening methods included taxa from the orders Dermaptera, Gastropoda, Trichoptera and Trombidiformes. Additionally, new Torix-associated families, genera and species were identified. These included haematophagous flies (*Simulium aureum; Anopheles plumbeus; Protocalliphora azurea*; Tabanidae), several parasitoid wasp families (e.g. Ceraphronidae; Diapriidae; Mymaridae), forest detritivores (e.g. Sciaridae; Mycetophilidae; Staphylinidae) and phloem-feeding bugs (Psyllidae; Ricaniidae). Feeding habits such as phloem-feeding, predation, detritivory or haematophagy were not correlated with any particular Torix *Rickettsia* subclade (Figure 6). Furthermore, parasitoid and aquatic lifestyles were seen across the phylogeny. All newly discovered putative Torix *Rickettsia* host taxa are described in Table 2, alongside previously discovered hosts in order to give an up to date overview of Torix-associated taxa.

[Insert Table 2 here]

Discussion

Symbiotic interactions between hosts and microbes are important drivers of host phenotype, with symbionts both contributing to, and degrading, host performance. Heritable microbes are particularly important contributors to arthropod biology, with marked attention focused on *Wolbachia*, the most common associate [9]. Members of the Rickettsiales, like *Wolbachia*, share an evolutionary history with mitochondria [42], such that a previous screen of BOLD submissions of mtDNA submissions observed *Wolbachia* as the main bacterial contaminant associated with DNA barcoding [34]. However, our screen found that *Rickettsia* amplicons were more commonly found in BOLD deposits compared to *Wolbachia* (0.41% vs 0.17% of deposits). Furthermore, Torix group *Rickettsia* were overrepresented in barcode misamplifications (95%) when compared to other groups within the genus. A comparison of the most commonly used barcoding primers to *Wolbachia* and *Rickettsia* genomes suggest homology of the forward primer 3’ end was likely responsible for this bias towards Torix *Rickettsia* amplification. To gain a clearer understanding of the relative balance of Torix group to other *Rickettsia* within symbioses and habitats, a targeted screen and bioinformatic approach was also undertaken. Through these three screens, a broad range of host diversity associated with Torix *Rickettsia* was uncovered.

As the *in silico* and empirical evidence suggests *Rickettsia COI* amplification is not uncommon [31–33], why has this phenomenon not been described more widely before? The previous large-scale non-target *COI* study using BOLD submissions [34], revealed only *Wolbachia* hits. This screen involved comparison to a *Wolbachia*-specific reference library and was thus likely to miss *Rickettsia*. Additionally, there has been a lack of Torix *Rickettsia COI* homologues to

compare barcodes to until recently, where a multilocus identification system, including *COI* was devised [27]. Indeed, out of the non-target *COI* dataset received in this study, some of the *Rickettsia* contaminants were tentatively described by BOLD as *Wolbachia* due to the previous absence of publicly available *Rickettsia COI* to compare.

Although *Rickettsia* will only interfere with barcoding in a minority of cases (~0.4%), it is likely that alternate screening primers for some studies will need to be considered. In a demonstration of how unintended *Rickettsia* amplifications can affect phylogeographic studies relying on DNA barcoding, a *Rickettsia COI* was conflated with the mtDNA *COI* of a species of freshwater amphipod, *Paracalliope fluvitalis* [30]. Subsequently, supposed unique mtDNA haplotypes were allocated to a particular collection site, whereas this merely demonstrated the presence of Torix *Rickettsia* in host individuals in this lake. Contrastingly, non-target *Rickettsia* amplification can also allow for the elucidation of a novel host range of the symbiont [31–33] and this has been exemplified with our probing of BOLD.

Previously, several host orders have been associated with Torix *Rickettsia*, including Araneae, Coleoptera, Diptera, Hemiptera and Odonata [24,28,43–45]. Newly uncovered putative host orders from this study include Dermaptera, Gastropoda, Trichoptera and Trombidiformes (Table 2). These data emphasise the broad host range of Torix *Rickettsia* across arthropods and invertebrates, with two additional cases from nucleariid amoebae [25,26]. This host range is complementary to *Rickettsia*'s sister genus '*Candidatus* Megaira' (formally the Hydra group of *Rickettsia*) which are present in multiple unicellular eukaryote families, and in a few invertebrates like *Hydra* [46].

Caution needs to be taken when interpreting what these newly found associations mean, as mere presence of *Rickettsia* DNA does not definitively indicate an endosymbiotic association. For example, bacterial DNA integrations into the host nuclear genome have been widely reported [47]. However, none of the protein-coding genes sequenced in this study showed signs of a frameshift, suggesting a lack of pseudogenization that is typical of a nuclear insertion. Furthermore, parasitism or ingestion of symbiont-infected biota (e.g. protists) could also result in bacteria detection [48–50]. Whilst protist reads were found in some datasets, these were usually at a much lower depth compared to the symbiont [39]. In one of the few instances where protist reads were greater than *Rickettsia* (Dataset SRR5298327), this was from our own previous study where a true endosymbiosis between insect and symbiont was confirmed through FISH imaging [27]. Similarly, although an 18S sequence aligned to a parasitoid wasp was observed in the SRA dataset from *Bemisia tabaci* (SRR6313831), previous work has also demonstrated a true endosymbiosis between *B. tabaci* and Torix *Rickettsia* [51]. Overall, these data suggest that detecting contamination from *Rickettsia*-infected taxa such as protists and parasitoid wasps is uncommon within our study.

Model-based estimation techniques suggest *Rickettsia* are present in between 20-42% of terrestrial arthropod species [12]. However, the targeted PCR screen in this study gave an estimated species prevalence of 8.9% for terrestrial species. This discrepancy is likely due to targeted screens often underestimating the incidence of symbiont hosts due to various methodological biases including small within-species sample sizes (missing low-prevalence infections) [29]. Importantly, the inclusion and exclusion of specific ecological niches can also

lead to a skewed view of *Rickettsia* symbioses. A previous review of *Rickettsia* bacterial and host diversity by Weinert et al. [13] suggested a possible (true) bias towards aquatic taxa in the Torix group. In accordance with this, our targeted screen demonstrated Torix *Rickettsia* infections were more prevalent in aquatic insect species compared to terrestrial (although this is likely not the case for invertebrates in general due to a Torix *Rickettsia* hot spot in spiders). Our observed over-representation of Torix group *Rickettsia* (17/19 strains) contrasts with Weinert's findings which show a predominance of Belli infections and is likely due to the latter study's near absence of screened aquatic insects and spiders. Our additional use of a bioinformatics approach based on the SRA appears to confirm that Belli and Torix are two of the most common *Rickettsia* groups among arthropods. Overall, these multiple screening methods suggest Torix *Rickettsia* are more widespread than previously thought and their biological significance underestimated.

Previous studies have used either one or two markers to identify the relatedness of strains found in distinct hosts. In this study, we use the multilocus approach developed in Pilgrim et al. [27] to understand the affiliation of Torix *Rickettsia* from diverse invertebrate hosts. Our analysis of Torix strains indicates that closely related strains are found in distantly related taxa. Closely related *Rickettsia* are also found in putative hosts from different niches and habitats – for instance, the *Rickettsia* strains found in terrestrial blood feeders do not lie in a single clade, but rather are allied to strains found in non-blood feeding host species. Likewise, strains in phloem-feeding insects are diverse rather than commonly shared.

The distribution of Torix *Rickettsia* across a broad host range suggests host shifts are occurring between distantly related taxa. It is notable that parasitoid wasps are commonly infected with *Rickettsia* and have been associated with enabling symbiont host shifts [48]. Aside from endoparasitoids, it is also possible that plant-feeding can allow for endosymbiont horizontal transmission [52,53]. For example, *Rickettsia* horizontal transmission has been demonstrated in *Bemisia* whiteflies infected by phloem-feeding [52,54]. Finally, ectoparasites like the Torix-infected water mites of the Calyptostomatidae family, could also play a role in establishing novel *Rickettsia*-host associations, as feeding by mites has been observed to lead to host shifts for other endosymbiont taxa [55]. Indeed, if multiple horizontal transmission paths do exist, this could account for the diverse plethora of infected taxa, as well as arthropods identified in this study which harbour more than one strain of symbiont [56].

The finding that Torix *Rickettsia* are associated with a broad range of invertebrates leads to an obvious question: what is the impact and importance of these symbiotic associations? Previous work has established Torix *Rickettsia* represent heritable symbionts and it is likely that this is true generally. There have, however, been few studies on their impact on the host. In the earliest studies [22,23], *Torix* spp. leeches infected with *Rickettsia* were observed to be substantially larger than their uninfected counterparts. Since then, the only observation of note, pertaining to the Torix group, is the reduced ballooning (dispersal) behaviour observed in infected *Erigone atra* money spiders [57]. Overall, the incongruencies in host and Torix *Rickettsia* phylogenies (suggesting a lack of co-speciation and obligate mutualism), along with the lack of observed sex bias in carrying the symbiont, indicate facultative benefits are the most likely symbiotic relationship [29]. However, *Rickettsia* induction of thelytokous

parthenogenesis (observed in Belli *Rickettsia* [58,59]) should not be discounted in Torix infected parasitoid wasps identified in this study. To add to the challenge of understanding Torix *Rickettsia* symbioses, the challenges of laboratory rearing of many Torix *Rickettsia* hosts has led to difficulties in identifying model systems to work with. However, the large expansion of our Torix group host knowledge can now allow for a focus on cultivatable hosts (e.g phloem-feeding bugs).

To conclude, we have shown that large-scale DNA barcoding initiatives of arthropods can include non-target amplification of Torix *Rickettsia*. By examining these non-target sequences, alongside a targeted screen and SRA search, we have uncovered numerous previously undetected putative host associations. Our findings lay bare multiple new avenues of inquiry for Torix *Rickettsia* symbioses.

Potential Implications

A particularly important group for future study of Torix *Rickettsia* interactions are haematophagous host species. Our discovery of *Rickettsia*-associated tabanid and simulid flies, alongside *Anopheles plumbeus* mosquitoes, add to existing blood-feeders previously identified as Torix group hosts which include sand flies [60,61], fleas [62], ticks [63,64] bed bugs [65] and biting midges [27]. Some *Rickettsia* strains are known to be transmitted to vertebrates via haematophagy [66]. However, there is no evidence to date for vertebrate pathogenic potential for the Torix group. Despite this, Torix *Rickettsia* could still play a significant role in the ecology of vectors of disease. A key avenue of research is whether these endosymbionts alter vectorial capacity, as found for other associations [67]. In contrast to the

widely reported virus blocking phenotype observed in *Wolbachia*-infected vectors [68,69], Torix *Rickettsia* has recently been associated with a virus potentiating effect in *Bemisia* white flies vectoring Tomato yellow leaf curl virus [70]. Additionally, we uncovered a *Rickettsia*-infected psyllid (*Cacopsylla melanoneura*) which is a vector of *Phytoplasma mali* (apple proliferation) [71]. Thus, the question of Torix *Rickettsia* vector-competence effects is clearly of widespread relevance and deserves further attention.

Methods

a) Interrogation of the Barcode of Life Data System (BOLD)

Assessment of non-target microbe amplicons

BOLD data curation involves identifying non-target *COI* sequences from common contaminants (e.g. human and bacteria) or erroneous morphological identifications [38]. The designation of bacterial contaminants by BOLD, from a dataset containing 3,817 non-target sequences [36], was confirmed by the taxonomic classification program, Kaiju, using default parameters [72]. Sequences were then placed phylogenetically to refine taxonomy further. To this end, barcodes confirmed as microbial sequences were aligned using the "L-INS-I" algorithm in MAFFT v7.4 [73] before using Gblocks [74] to exclude areas of the alignment with excessive gaps or poor alignment. ModelFinder [75] then determined the TIM3+F+I+G4 model to be used after selection based on default "auto" parameters using the Bayesian information criteria. A maximum likelihood (ML) phylogeny was then estimated with IQTree [76] using an alignment of 561 nucleotides and 1000 ultrafast bootstraps [77]. The Rickettsiales genera *Anaplasma*, *Rickettsia*, *Orientia* and *Wolbachia* (Supergroups A, B, E and F), as well as the Legionellales genera *Legionella* and *Rickettsiella*, were included in the analysis as references

(as suggested by Kaiju). Finally, both phylogram and cladogram trees (the latter for ease of presentation) were drawn and annotated based on host taxa (order) using the EvolView [78] online tree annotation and visualisation tools.

A determining factor for non-target amplification of bacteria is primer site matching to microbial associates. Subsequently, pairwise homology of the primer set predominantly used for BOLD barcode screening was compared to *Rickettsia* and *Wolbachia COI* genes.

Further phylogenetic analysis

COI sequence alone provides an impression of the frequency with which *Rickettsia* associates are found in barcoding studies. However, they have limited value in describing the diversity of the *Rickettsia* found. To provide further insight into the diversity of *Rickettsia* using a multilocus approach, we obtained 186 DNA extracts from the archive at the Centre for Biodiversity Genomics (University of Guelph, Canada) that had provided *Rickettsia* amplicons in the previous screen. DNA extracts were chosen based on assorted geographic location, host order and phylogenetic placement. Multilocus PCR screening and phylogenetic analysis of *Rickettsia* was then completed, using the methodology in Pilgrim et al. which utilised primers conserved across all known clades of the *Rickettsia* genus [27]. However, slight variations include the exclusion of the *atpA* gene due to observed recombination at this locus. Furthermore, the amplification conditions for the *17KDa* locus was changed because a Torix *Rickettsia* reference DNA extract (Host: *Simulium aureum*) failed to amplify with the primer set Ri_17KD_F/ Ri_17KD_R from Pilgrim et al. [27]. Subsequently, a *17KDa* alignment from genomes spanning the Spotted fever, Typhus, Transitional, Belli, Limoniae groups, and the

genus '*Candidatus* Megaira' was generated to design a new set of primers using the online tool PriFi [79].

Once multilocus profiles of the *Rickettsia* had been established, we tested for recombination within and between loci using RDP v4 [80] using the MaxChi, RDP, Chimaera, Bootscan and GENECONV algorithms with the following criteria to assess a true recombination positive: a p-value of <0.001; sequences were considered linear with 1000 permutations being performed. Samples amplifying at least 3 out of 4 genes (*16S rRNA*, *17KDa*, *COI* and *gltA*) were then concatenated and their relatedness estimated using maximum likelihood as previously described. The selected models used in the concatenated partition scheme [81] were as follows: *16S rRNA*: TIM3+F+R2; *17KDa*: GTR+F+I+G4; *COI:*TVM+F+I+G4; *gltA:* TVM+F+I+G4. Accession numbers for all sequences used in phylogenetic analyses can be found in Additional file 10.

Re-barcoding Rickettsia-containing BOLD DNA extracts

Aside from phylogenetic placement of these *Rickettsia*-containing samples, attempts were made to extract an mtDNA barcode from these taxa in order to identify the hosts of infected specimens. This is because morphological taxonomic classification of specimens in BOLD is usually only down to the order level before barcoding takes place. Previous non-target amplification of *Rickettsia* through DNA barcoding of arthropod DNA extracts had occurred in the bed bug *Cimex lectularius*, with a recovery of the true barcode after using the primer set C1-J-1718/HCO1490, which amplifies a shortened 455 bp sequence within the *COI* locus. Subsequently, all samples were screened using these primers or a further set of secondary *COI*

primers (LCOt_1490/ MLepR1 and LepF1/C_ANTMR1D) if the first failed to give an adequate host barcode. All *COI* and *Rickettsia* multilocus screening primer details, including references, are available in Additional file 11.

Cycling conditions for *COI* PCRs were as follows: initial denaturation at 95°C for 5 min, followed by 35 cycles of denaturation (94°C, 30 sec), annealing (50°C, 60 sec), extension (72°C, 90 sec), and a final extension at 72°C for 7 min. *Rickettsia* and host amplicons identified by gel electrophoresis were subsequently purified enzymatically (ExoSAP) and Sanger sequenced through both strands using a BigDye® Terminator v3.1 kit (Thermo Scientific, Waltham, USA), and capillary sequenced on a 3500 xL Genetic Analyser (Applied Biosystems, Austin, USA). Forward and reverse reads were assessed in UGENE [82] to create a consensus sequence by eye with a cut-off phred (Q) score [83] of 20. Primer regions were trimmed from barcodes before being matched to the GenBank database by BLAST based on default parameters and an e-value threshold of <1e-85. Host taxonomy was determined by a barcode-based assignment of the closest BLAST hit, under the following criteria modified from Ramage et al. [50]:

1) Species level designation for at least 98% sequence identity.
2) Genus level designation for at least 95% sequence identity.
3) Family level designation for at least 85% sequence identity.

Additionally, all sequences were required to be at least >200 bp in length.

Assessment of barcoding success

One of the factors determining a successful *COI* bacterial amplification is the initial failure of an extract to amplify mtDNA. Subsequently, to determine the likelihood of this event within taxa, we used the 55,366 specimen representative data subset [37] to evaluate failure rates. To this end, all orders of host which gave at least one non-target *Rickettsia COI* hit were assessed. The barcoding success rate was determined as the proportion of specimens which matched initial morphotaxa assignment and were not removed after BOLD quality control [38]. As the total *Rickettsia* count was from a larger dataset than the one made available, an adjusted infection frequency for each taxon was calculated based on the representative data subset.

b) Targeted and bioinformatic *Rickettsia* screens

Targeted screen of aquatic and terrestrial arthropods

Overall, 1,612 individuals from 169 species, including both terrestrial (DNA extracts derived from European material, mostly from Duron et al. [11]) and aquatic invertebrates (largely acquired from the UK between 2016-2018), were screened. mtDNA *COI* amplification was conducted as a control for DNA quality. Some arthropods which could not be identified down to the species level morphologically or from barcoding were referred to as 'sp.'. To investigate symbiont infection status, rickettsial-specific primers based on *gltA* and *16S rRNA* genes were used for conventional PCR screening [27], with Sanger sequences obtained from at least one specimen per *Rickettsia* positive species to identify any misamplification false positives. Newly identified hosts of interest from BOLD and targeted screens were then placed phylogenetically (see sections above) before being mapped by lifestyle and diet.

It is known that there are taxonomic hot spots for endosymbiont infection, with for instance spiders being a hot spot for a range of microbial symbionts [43]. Therefore, analyses were performed that were matched at a taxonomic level (i.e. each taxon was represented in both the aquatic and terrestrial pools). To this end, the incidence of Torix *Rickettsia* was first compared in all insects. However, within insects, there is taxon heterogeneity between aquatic and terrestrial biomes (e.g. Ephemeroptera, Plecoptera in aquatic only, Lepidoptera in terrestrial only). The analysis was therefore narrowed to match insect orders present in both the aquatic and terrestrial community. Three insect orders, Hemiptera, Diptera and Coleoptera, fulfilled this criterion with good representation from each biome. For each case, the ratios of the infected:non-infected species between aquatic and terrestrial communities were compared in a Fisher's exact test with a *p*-value significance level of ≤0.05.

Search of the Sequence Read Archive (SRA) and GenBank

The SRA dataset [39] containing one individual from 1,341 arthropod species was screened with phyloFlash [40] using default primers, which finds, extracts and identifies SSU rRNA sequences. Reconstructed full *16S rRNA* sequences affiliated to *Rickettsia* were extracted and compared to sequences derived from the targeted screen phylogenetically (see sections above) to assess group representation within the genus. The microbial composition of all SRA datasets that did not result in a reconstructed *Rickettsia 16S rRNA* with phyloFlash were re-evaluated using Kraken2 [84], a k-mer based taxonomic classifier for short DNA sequences. A cut-off of at least 40k reads assigned to *Rickettsia* taxa was applied for reporting potential infections (theoretical genome coverage of ~ 1 – 4X assuming an average genome size of ~1.5Mb). As *Rickettsia*-infected protists and parasitoids have previously been reported

[25,26,59], phyloFlash was also used to identify reads aligned to these taxa to account for potential positives attributed to ingested protists or parasitisms.

We also examined GenBank for *Rickettsia* sequences deposited as invertebrate *COI* barcodes. To this end, a BLAST search of Torix *Rickettsia COI* sequences from previous studies [27,32] was conducted on the 29th June 2020. Sequences were putatively considered belonging to the Torix group if their similarity was >90% and subsequently confirmed phylogenetically as described above.

Table 1.1. Targeted *Rickettsia* screen of aquatic/semiaquatic invertebrates.

Aquatic/Semiaquatic invertebrate group	Species	Location	Year	No. tested	No positive
	Baetis muticus	Stirling, Scotland, UK	2017	3	0
	Baetis rhodani	Stirling, Scotland, UK	2017	3	0
	Cloeon dipterum	Cheshire, UK	2016	3	0
	Ecdyonurus sp.1	Stirling, Scotland, UK	2017	5	0
Ephemeroptera	*Ecdyonurus* sp.2	Cheshire, UK	2016	3	0
	Ecdyonurus venosus	Cheshire, UK	2016	6	0
	Leptophlebia vespertina	Hampshire, UK	2016	1	0
	Paraleptophlebia submarginata	Stirling, Scotland, UK	2017	3	0
	Rhithrogena semicolorata	Stirling, Scotland, UK	2017	3	0
	Hydropsyche sp.	Stirling, Scotland, UK	2017	3	0
Trichoptera	*Polycentropus flavomaculatus*	Cheshire, UK	2017	3	0
	Rhyacophila dorsalis	**Stirling, Scotland, UK**	**2017**	**3**	**2**
	Amphinemura sulcicollis	Stirling, Scotland, UK	2017	3	0
Plecoptera	*Dinocras cephalotes*	Stirling, Scotland, UK	2017	3	0
	Isoperla grammatica	Stirling, Scotland, UK	2017	3	0
	Perla bipunctata	Stirling, Scotland, UK	2017	3	0
	Corixa punctata	Cheshire, UK	2016	1	0
	Gerris sp.	Montferrier sur Lez, France	2006	12	0
	Gerris thoracicus	Cheshire, UK	2016	1	0
	Hydrometra stagnorum	Montferrier sur Lez, France	2006	20	0
Hemiptera	*Nepa cinerea*	Montferrier sur Lez, France	2006	3	0
	Notonecta glauca	Cheshire, UK	2016	2	0
	Plea minutissima	Notre Dame de Londres, France	2006	8	0
	Sigara lateralis	Notre Dame de Londres, France	2006	6	0
	Sigara striata	**Cheshire, UK**	**2006**	**2**	**1**
	Aedes sp.	Cheshire, UK	2017	8	0
	Aedes albopictus	Roma, Italy	2005	20	0
	Anopheles plumbeus	**Chester Zoo, UK**	**2018**	**2**	**2**

Diptera	**Chironomidae sp.**	**Cheshire, UK**	**2016**	**4**	**1**
	Chironomus acidophilus	Cheshire, UK	2017	1	0
	Chironomus plumosus	Notre Dame de Londres, France	2006	20	0
	Chironomus sp.	Cheshire, UK	2016	4	0
	Culex pipiens (ssp. quinquefasciatus)	Puerto Viejo de Talamanca, Costa Rica	2006	20	0
	Culex pipiens pipiens	St Nazaire de Pézan, France	2006	20	0
	Eristalinus sp.	Cheshire, UK	2016	3	0
	Eristalis tenax	Montpellier (grotte du zoo), France	2002	7	0
	***Glyptotendipes* sp.**	**Cheshire, UK**	**2016**	**1**	**1**
	***Hilara* interstincta**	**Cheshire, UK**	**2017**	**3**	**1**
	Simulium aureum	**Hampshire, UK**	**2017**	**1**	**1**
	Simulium ornatum	N/A	2003	12	0
	Tipula sp.	UK	2006	10	0
	Tipula oleracea	UK	2006	13	0
	***Zavrelimyia* sp.**	**Northumberland, UK**	**2017**	**1**	**1**
Coleoptera	*Agabus bipustulatus*	Cheshire, UK	2017	3	0
	Guignotus pusillus	Notre Dame de Londres, France	2006	12	0
	Unknown sp.1	Cheshire, UK	2017	2	0
	Unknown sp.2	Cheshire, UK	2017	3	0
Acarina	Unknown sp.	Cheshire, UK	2017	3	0
Isopoda	*Asellus aquaticus*	Cheshire, UK	2016	3	0
Amphipoda	*Gammarus pulex*	Stirling, Scotland, UK	2017	3	0
	Crangonyx pseudogracilis	Cheshire, UK	2016	6	0
Gastropoda	*Radix balthica*	Cheshire, UK	2016	3	0
	Planorbis sp.	Cheshire, UK	2016	3	0
	Galba truncatula	**Cheshire, UK**	**2017**	**20**	**3**
Hirudinea	*Erpobdella octoculata*	Cheshire, UK	2016	2	0
	Hemiclepsis marginata	Cheshire, UK	2017	1	0
Tricladida	Unknown sp.	Cheshire, UK	2016	1	0

A species was deemed positive through PCR and designated to *Rickettsia* group after Sanger sequencing and phylogenetic placement. All strains belong to the Torix group.

Table 1.2. Targeted *Rickettsia* screen of terrestrial invertebrates.

Terrestrial Invertebrate group	Species	Location	Year	Number tested	Number positive
	Agelenopsis aperta	Tennessee, USA	N/A	12	0
	Allopecosa pulverulenta	Berne, Germany	N/A	16	0
	Amaurobius fenestralis	**Montpellier, France**	**2006**	**16**	**1**
	Araneus diadematus	Beerse, Belgium	N/A	19	0
	Araneus diadematus	Greater London, UK	N/A	8	0
	Argiope bruennichi	Hamburg, Germany	N/A	7	0
	Argiope lobata	Spain	N/A	7	0
	Argiope lobata	Israel	N/A	4	0
	Cyclosa conica	Brandenburg, Germany	N/A	11	0
	Dysdera crocata	Montpellier, France	2006	2	0
	Enoplognatha ovata	Greater London, UK	N/A	20	0
	Erigone atra	Cheshire, UK	2017	1	0

Araneae	*Evarcha falcata*	Beerse, Belgium	N/A	5	0
	Holochnemus pluchei	Montpellier, France	2006	7	0
	Hylyphantes graminicola	**Cheshire, UK**	**2017**	**1**	**1**
	Larinioides cornutus	Greater London, UK	N/A	6	0
	Larinoides sclopetarius	Hamburg, Germany	N/A	17	0
	Linyphia triangularis	**Berlin, Germany**	**N/A**	**9**	**9**
	Linyphia triangularis	Greater London, UK	N/A	6	0
	Lycosa sp.	Cheshire, UK	2017	2	0
	Metellina mengei	Greater London, UK	N/A	13	0
	Metellina segmentata	Brandenburg, Germany	N/A	9	0
	Neriene clathrata	Beerse, Belgium	N/A	13	0
	Neriene peltata	Cheshire, UK	2017	1	0
	Pachygnatha degeeri	Berne, Germany	N/A	11	0
	Pachygnatha listeri	Beerse, Belgium	N/A	17	0
	Pardosa lugubris	**Darmstadt, Germany**	**N/A**	**20**	**1**
	Pardosa pullata	Brandenburg, Germany	N/A	20	0
	Pardosa purbeckensis	Belgium	N/A	19	0
	Pholcus phalangioides	**Berlin, Germany**	**N/A**	**20**	**17**
	Pisaura mirabilis	**Greater London, UK**	**N/A**	**12**	**1**
	Tetragnatha montana	Greater London, UK	N/A	20	0
	Tetragnatha sp.	Hampshire, UK	2017	3	0
	Unknown sp.	Cheshire, UK	2017	2	0
	Xysticus cristatus	Cambridgeshire, UK	N/A	16	0
Opiliones	*Leiobunum rotundum*	Feurs, France	2006	6	0
Ixodida	*Ixodes uriae*	Hornøya, Norway	2005	19	0
	Rhipicephalus microplus	New Caledonia, France	2003	1	0
Scorpiones	*Euscorpius flavicauda*	St Nazaire de Pézan, France	2006	1	0
Diplopoda	*Ommatoiulus* sp.	Cheshire, UK	2016	1	0
Neuroptera	Unknown sp.	Cheshire, UK	2017	1	0
Mecoptera	*Panorpa* sp.	Cheshire, UK	2017	2	0
Orthoptera	*Calliptamus italicus*	Notre Dame de Londres, France	2016	18	0
	Chorthippus brunneus	Uk	2006	20	0
	Gryllomorpha dalmatina	Montpellier, France	2006	2	0
Blattaria	*Loboptera decipiens*	Montpellier, France	2006	17	0
Mantodae	*Iris oratoria*	St Nazaire de Pézan, France	2006	6	0
	Mantis religiosa	Feurs, France	2006	3	0
Dermaptera	*Forficula Auricularia*	Feurs, France	2006	9	0
Hemiptera	*Aphis fabae*	Montpellier, France	2006	12	0
	Aphis nerii	Montpellier, France	2006	8	0
	Baizongia pistaciae	Viols le Fort, France	2006	12	0
	Cicadella viridis	L'Olme, France	2006	16	0
	Cimex lectularius	**Yorkshire, UK**	**2008**	**12**	**12**
	Elasmucha grisea	Greater London, UK	2006	16	0
	Graphosoma italicum	Montpellier, France	2006	12	0
	Lygaeus equestris	Montpellier, France	2006	12	0
	Notostira elongata	L'Olme, France	2006	11	0
	Pyrrhocoris apterus	Montpellier, France	2006	11	0
	Rhyparochromus vulgaris	Castelnaudary, France	2006	20	0
	Anaspis frontalis	Mont Barri, France	2004	12	0
	Anthaxia nitidula	Mont Barri, France	2004	20	0
	Anthaxia sp.	Mont Barri, France	2004	16	0
	Calvia 14-guttata	Greater London, UK	2006	6	0
	Capnodis tenebrionis	Montpellier, France	2006	1	0
	Cetonia aurata	Feurs, France	2006	3	0
	Cetonia aurata	Mont Barri, France	2004	12	0
	Chrysolina varians	Mont Barri, France	2004	18	0
	Clytus arietis	Mont Barri, France	2004	20	0

Coleoptera	*Dermestes* sp.	Mont Barri, France	2004	20	0
	Dermestes tessellatocollis	Cheshire, UK	2016	2	0
	Gastrophysa sp.	Greater London, UK	2006	20	0
	Geotrupes stercorarius	Mont Barri, France	2004	3	0
	Larinus scolymi	Aldira de Irmeros, Spain	2005	12	0
	Leptinotarsa decemlineata	Feurs, France	2006	10	0
	Mordellistena sp.	Mont Barri, France	2004	10	0
	Oedemera sp.	Mont Barri, France	2004	20	0
	Oncocerna sp.	Mont Barri, France	2004	20	0
	Phyllobius argentatus	**Mont Barri, France**	**2004**	**15**	**4†**
	Pseudovadonia livida	Mont Barri, France	2004	19	0
	Stenopterus sp.	Mont Barri, France	2004	20	0
Diptera	*Braula coeca*	Ouessant, France	2002	4	0
	Chorisops tunisiae	Montpellier, France	2003	8	0
	Delia antiqua	N/A	N/A	11	0
	Delia platura	N/A	N/A	11	0
	Delia radiacum	N/A	N/A	10	0
	Gasterophilus intestinalis	France	N/A	10	0
	Hippobosca equina	Restinclières, France	2006	15	0
	Lonchoptera lutea	Cheshire, UK	2017	3	0
	Medetera petrophila	St Bauzille de Putois, France	2003	12	0
	Musca domestica	L'Olme, France	2006	20	0
	Musca vitripennis	Notre Dame de Londres, France	2003	8	0
	Neomyia cornicina	Notre Dame de Londres, France	2003	8	0
	Protocalliphora sp.	Corse, France	2003	2	0
	Protocalliphora azurea	**Montpellier, France**	**2005**	**12**	**12**
	Psila rosae	N/A	N/A	11	0
	Stomoxys calcitrans	Le Malzieu, France	2001	11	0
Lepidoptera	*Chilo phragmitellus*	Feurs, France	2006	10	0
	Euplagia quadripunctaria	Feurs, France	2006	2	0
	Pieris brassicae	Feurs, France	2006	7	0
	Plodia interpunctella	Montpellier, France	2006	12	0
	Thymelicus lineola	Greater London, UK	2006	15	0
	Thymelicus sylvestris	Greater London, UK	2006	2	0
	Triodia sylvina	Montpellier, France	2006	4	0
Hymenoptera	*Amblyteles armatorius*	St Nazaire de Pézan, France	2006	1	0
	Amegilla albigena	St Nazaire de Pézan, France	2006	13	0
	Amegilla ochroleuca	St Nazaire de Pézan, France	2006	3	0
	Anthidium florentinum	St Nazaire de Pézan, France	2006	6	0
	Apis mellifera	UK	2006	9	0
	Bombus terrestris	North West, Switzerland	2006	20	0
	Diplolepis rosae	L'Olme, France	2006	2	0
	Formica lugubris	UK	2006	10	0
	***Pachycrepoideus* sp.**	**UK**	**N/A**	**94**	**6‡**
	Polistes dominulus	St Nazaire de Pézan, France	2006	4	0
	Polistes nimpha	St Nazaire de Pézan, France	2006	19	0
	Sceliphron caementarium	St Nazaire de Pézan, France	2006	3	0

A species was deemed positive through PCR and designated to *Rickettsia* group after Sanger sequencing and phylogenetic placement. All strains belong to the Torix group except †=Rhyzobius and ‡=Belli.

Table 2. Torix *Rickettsia* hosts known to date alongside screening method.

Order	Host	Screening method	Reference
Amphipoda	***Paracalliope fluviatilis* (Paracalliopiidae)**	**GenBank search**	**This study**
	Paraleptamphopus sp. (Paraleptamphopidae)	Barcoding	[33]
	Senticaudata sp.	Barcoding	[33]
Araneae	***Amaurobius fenestralis* (Amaurobiidae)**	**Targeted PCR**	**This study**
	Amaurobioides africana (Anyphaenidae)	Barcoding	[32]
	Araneus diadematus (Araneidae)	Targeted PCR	[43]
	Dysdera microdonta (Dysderidae)	Barcoding	[31]
	Linyphiidae spp.	Targeted PCR	[43]
	***Linyphia triangularis* (Linyphiidae)**	**Targeted PCR**	**This study**
	***Pardosa lugubris* (Lycosidae)**	**Targeted PCR**	**This study**
	***Pholcus phalangioides* (Pholcidae)**	**Targeted PCR**	**This study**
	***Pisaura mirabilis* (Pisauridae)**	**Targeted PCR**	**This study**
	Metellina mengei (Tetragnathidae)	Targeted PCR	[43]
Coleoptera	*Deronectes* spp. (Dytiscidae)	Targeted PCR, FISH and TEM	[24]
	***Dytiscidae* sp.**	**Barcoding**	**This study**
	Stegobium paniceum (Ptinidae)	Non-targeted (16S) PCR	[85]
	***Prionocyphon limbatus* (Scirtidae)**	**Barcoding**	**This study**

	***Labidopullus appendiculatus* (Staphylinidae)**	**SRA search**	**This study**
	***Platyusa sonomae* (Staphylinidae)**	**SRA search**	**This study**
	***Pseudomimeciton antennatum* (Staphylinidae)**	**SRA search**	**This study**
	***Staphylinidae* sp.**	**Barcoding**	**This study**
	***Pimelia* sp. (Tenebrionidae)**	**GenBank search**	**This study**
Dermaptera	***Forficula* sp. (Forficulidae**)	**GenBank search**	**This study**
	**unknown sp.**	**Barcoding**	**This study**
Diplopoda	*Polydesmus complanatus* (Polydesmidae)	Targeted PCR	[86]
	**unknown sp.**	**Barcoding**	**This study**
Diptera	***Protocalliphora azurea* (Calliphoridae)**	**Targeted PCR**	**This study**
	***Cecidomyiidae* sp.**	**Barcoding**	**This study**
	***Chaoborus trivittatus* (Chaoboridae)**	**SRA search**	**This study**
	***Mochlonyx cinctipes* (Chaoboridae)**	**SRA search**	**This study**
	***Glyptotendipes* sp. (Chironomidae)**	**Targeted PCR**	**This study**
	***Zavrelimyia* sp. (Chironomidae)**	**Targeted PCR**	**This study**
	Culicoides spp. (Ceratopogonidae)	Targeted PCR and FISH	[27]
	***Anopheles plumbeus* (Culicidae)**	**Targeted PCR**	**This study**
	Dolichopodidae spp.	Targeted PCR	[44]
	Empididae spp.	Targeted PCR	[44]
	Limonia chorea (Limoniidae)	N/A	Unpublished (AF322443)
	***Boletina villosa* (Mycetophilidae)**	**Barcoding**	**This study**
	**Gnoriste bilineata (Mycetophilidae)**	**SRA search**	**This study**
	***Mycetophila lunata* (Mycetophilidae)**	**GenBank search**	**This study**
	***Psilidae* sp.**	**Barcoding**	**This study**

	Lutzomyia apache (Psychodidae)	Targeted PCR	[61]
	Phlebotomus chinensis (Psychodidae)	Non-targeted (16S) PCR	[60]
	***Sciaridae* sp.**	**Barcoding**	**This study**
	***Pherbellia tenuipes* (Sciomyzidae)**	**Barcoding**	**This study**
	***Simulium aureum* (Simuliidae)**	**Targeted PCR**	**This study**
	***Tabanidae* sp.**	**Barcoding**	**This study**
Gastropoda	***Galba truncatula* (Lymnaeidae)**	**Targeted PCR**	**This study**
Haplotaxida	*Mesenchytraeus solifugus* (Enchytraediae)	Non-targeted (16S) PCR	[87]
Hemiptera	*Bemisia tabaci* (Aleyrodidae)	Targeted PCR and FISH	[51]
	Nephotettix cincticeps (Cicadellidae)	Targeted PCR, FISH and TEM	[88]
	Platypleura kaempferi (Cicadidae)	Non-targeted (16S) PCR	[89]
	***Cimex lectularius* (Cimicidae)**	**Targeted PCR**	**This study/**[65]
	***Sigara striata* (Corixidae)**	**Targeted PCR**	**This study**
	***Metcalfa pruinosa* (Flatidae)**	**GenBank search**	**This study**
	***Flavina* sp. (Issidae)**	**GenBank search**	**This study**
	Centrotus cornutus (Membracidae)	Non-targeted (16S) PCR and TEM	[90]
	Gargara genistae (Membracidae)	Non-targeted (16S) PCR and TEM	[90]
	Macrolophus pygmaeus (Miridae)	Non-targeted (16S) PCR and FISH	[45]
	***Cacopsylla melanoneura* (Psyllidae)**	**Barcoding**	**This study**
	***Chamaepsylla hartigii* (Psyllidae)**	**Barcoding**	**This study**
	***Ricaniidae* sp.**	**Barcoding**	**This study**

Hirudinea	*Hemiclepsis* spp. (Glossiphoniidae)	Targeted PCR and TEM	[23]
	Torix spp. (Glossiphoniidae)	Targeted PCR and TEM	[23]
Hymenoptera	*Asobara tabida* (Braconidae)	Non-targeted (16S) PCR	[91]
	***Ceraphronidae* sp.**	**Barcoding**	**This study**
	***Diapriidae* sp.**	**Barcoding**	**This study**
	***Eucharitidae* sp.**	**GenBank search**	**This study**
	Quadrastichus mendeli (Eulophidae)	Non-targeted (16S) PCR and FISH	[92]
	***Formicidae* sp.**	**GenBank search**	**This study**
	Atta colombica (Formicidae)	Non-targeted (16S) PCR	Unpublished (LN570502)
	***Megaspilidae* sp.**	**Barcoding**	**This study**
	***Mymaridae* sp.**	**Barcoding**	**This study**
	***Platygastridae* sp.**	**Barcoding**	**This study**
Ixodida	*Argas japonica* (Argasidae)	Non-targeted (16S) PCR	[64]
	Ixodes ricinus (Ixodidae)	Targeted PCR	[63]
Megaloptera	*Sialis lutaria* (Sialidae)	Targeted PCR	[93]
Neuroptera	*Chrysotropia ciliata* (Chrysopidae)	Targeted PCR	[93]
Nucleariida	*Nuclearia pattersoni* (Nucleariidae)	Non-targeted (16S) PCR	[25]
	Pompholyxophrys punicea (Pompholyxophryidae)	Single cell sequencing	[26]
Odonata	***Calopteryx maculata* (Calopterygidae)**	**GenBank search**	**This study**
	Coenagrionidae spp.	Targeted PCR and FISH	[28]
	Sympetrum fonscolombii (Libellulidae)	Targeted PCR	[28]
	Polythoridae spp.	Targeted PCR	[28]
	Neoneura sylvatica (Protoneuridae)	Targeted PCR	[28]
Psocoptera	***Myopsocidae* sp.**	**Barcoding**	**This study**
	***Philotarsus californicus* (Philotarsidae)**	**Barcoding**	**This study**
	Cerobasis guestfalica (Trogiidae)	Targeted PCR and FISH	[94]

Siphonaptera	*Nosopsyllus fasciatus* (Ceratophyllidae)	Targeted PCR	[62]
Trichoptera	***Lepidostoma hoodi* (Lepidostomatidae)**	**Barcoding**	**This study**
	***Rhyacophila dorsalis* (Rhyacophilidae)**	**Targeted PCR**	**This study**
	***Sericostoma* sp. (Sericostomatidae)**	**SRA search**	**This study**
Trombidiformes	***Calyptostomatidae* sp.**	**Barcoding**	**This study**

Bold entries indicate hosts identified in this study. FISH=fluoresence *in-situ* hybridisation; TEM=transmission electron microscopy; SRA=sequence read archive. Accession numbers for *Rickettsia* sequences from newly detected hosts can be found in Additional files 8 and 10.

Availability of Supporting Data and Materials

The data sets supporting the findings of this study are openly available in:

The Barcode of Life Data System (BOLD) repository at http://dx.doi.org/10.5883/DS-RICKET and the Figshare repository at http://dx.doi.org/10.6084/m9.figshare.12801107 and http://dx.doi.org/10.6084/m9.figshare.12801140.

For DNA sequences, accessions are: Bioproject number PRJEB38316; LR798809-LR800243; LR812141-LR812260; LR812269-LR812283; LR812678; LR813674-LR813676; LR813730.

Declarations

List of Abbreviations

BOLD = Barcode of Life Data System

COI = cytochrome c oxidase I

FISH = fluorescence *in-situ* hybridisation

SRA = Sequence Read Archive

Ethics Approval

Not applicable.

Consent for Publication

Not applicable.

Competing Interests

The authors declare that they have no competing interests.

Funding

This work was supported by: a BBSRC Doctoral Training Partnership studentship (BB/M011186/1) awarded to JP; a Development and Promotion of Science and Technology Talents Project (DPST) of the Institute for the Promotion of Teaching Science and Technology, Thailand to PT and a Harry Smith Vacation studentship (Microbiology society) and a NERC ACCE DTP studentship (NE/L002450/1) to HRD.

Acknowledgments

We would like to thank Dr. Michael Gerth for kindly providing comments on the manuscript.

Author contributions

JP, GDDH, MB and MAS: conception and design of the study. MAS, EVZ, SR and JRD:

assembling BOLD datasets and providing DNA extracts for laboratory experiments. Field and laboratory work: JP, CRM and PT. SRA work: HRD and SS. Analyses and interpretation of the data, drafting of the manuscript: JP, PT, HRD, GDDH, MB and SS. All authors assisted in critical revision of the manuscript.

References

1. McFall-Ngai M, Hadfield MG, Bosch TCG, Carey H V., Domazet-Lošo T, Douglas AE, et al. Animals in a bacterial world, a new imperative for the life sciences. Proc Natl Acad Sci. 2013;110:3229–36.

2. Hurst GDD. Extended genomes: symbiosis and evolution. Interface Focus. 2017;7:20170001.

3. Łukasik P, Guo H, van Asch M, Ferrari J, Godfray HCJ. Protection against a fungal pathogen conferred by the aphid facultative endosymbionts Rickettsia and Spiroplasma is expressed in multiple host genotypes and species and is not influenced by co-infection with another symbiont. J Evol Biol. 2013;26:2654–61.

4. Teixeira L, Ferreira A, Ashburner M. The bacterial symbiont Wolbachia induces resistance to RNA viral infections in Drosophila melanogaster. PLoS Biol. 2008;6:2753–63.

5. Rio RVM, Attardo GM, Weiss BL. Grandeur Alliances: Symbiont metabolic integration and obligate arthropod hematophagy. Trends Parasitol. 2016;32:739–49.

6. Douglas AE. The microbial dimension in insect nutritional ecology. Funct Ecol. 2009;23:38–47.

7. Hurst GDD, Frost CL. Reproductive parasitism: Maternally inherited symbionts in a biparental world. Cold Spring Harb Perspect Biol. 2015;7:a017699.

8. Munson MA, Baumann P, Kinsey MG. Buchnera gen. nov. and Buchnera aphidicola sp. nov., a taxon consisting of the mycetocyte-associated, primary endosymbionts of aphids. Int J Syst Bacteriol. 1991;41:566–8.

9. Zug R, Hammerstein P. Still a host of hosts for Wolbachia: Analysis of recent data suggests that 40% of terrestrial arthropod species are infected. PLoS One. 2012;7:e38544.

10. Siozios S, Gerth M, Griffin JS, Hurst GDD. Symbiosis: Wolbachia host shifts in the fast lane. Curr Biol. 2018;28:R269–71.

11. Duron O, Bouchon D, Boutin S, Bellamy L, Zhou L, Engelstädter J, et al. The diversity of reproductive parasites among arthropods: Wolbachia do not walk alone. BMC Biol. 2008;6:27.

12. Weinert LA, Araujo-Jnr E V, Ahmed MZ, Welch JJ. The incidence of bacterial endosymbionts in terrestrial arthropods. Proc R Soc B. 2015;282:20150249.

13. Weinert LA, Werren JH, Aebi A, Stone GN, Jiggins FM. Evolution and diversity of Rickettsia bacteria. BMC Biol. 2009;7:6.

14. Perlman SJ, Hunter MS, Zchori-Fein E. The emerging diversity of Rickettsia. Proc R Soc B. 2006;273:2097–106.

15. Ricketts HT. A micro-organism which apparently has a specific relationship to Rocky Mountain spotted fever. J Am Med Assoc. 1909;52:379–80.

16. da Rocha-Lima H. Zur Aetiologie des Fleckfiebers. Dtsch Medizinische Wochenschrift. 1916;53:567–9.

17. Werren JH, Hurst GD, Zhang W, Breeuwer JA, Stouthamer R, Majerus ME. Rickettsial relative associated with male killing in the ladybird beetle (Adalia bipunctata). J Bacteriol. 1994;176:388–94.

18. Chen D-Q, Campbell BC, Purcell AH. A new Rickettsia from a herbivorous insect, the pea aphid Acyrthosiphon pisum (Harris). Curr Microbiol. 1996;33:123–8.

19. Hurst GDD, Walker LE, Majerus MEN. Bacterial infections of hemocytes associated with the maternally inherited male-killing trait in British populations of the two spot ladybird, Adalia bipunctata. J Invertebr Pathol. 1996;68:286–92.

20. Hendry TA, Hunter MS, Baltrus DA. The facultative symbiont Rickettsia protects an invasive whitefly against entomopathogenic Pseudomonas syringae strains. Appl Environ Microbiol. 2014;80:7161–8.

21. Himler AG, Adachi-Hagimori T, Bergen JE, Kozuch A, Kelly SE, Tabashnik BE, et al. Rapid spread of a bacterial symbiont in an invasive whitefly is driven by fitness benefits and female bias. Science. 2011;332:254–6.

22. Kikuchi Y, Fukatsu T. Rickettsia infection in natural leech populations. Microb Ecol. 2005;49:265–71.

23. Kikuchi Y, Sameshima S, Kitade O, Kojima J, Fukatsu T. Novel clade of Rickettsia spp. from leeches. Appl Environ Microbiol. 2002;68:999–1004.

24. Küchler SM, Kehl S, Dettner K. Characterization and localization of Rickettsia sp. in water beetles of genus Deronectes (Coleoptera: Dytiscidae). FEMS Microbiol Ecol. 2009;68:201–11.

25. Dyková I, Veverková M, Fiala I, Macháčková B, Pecková H. Nuclearia pattersoni sp. n. (Filosea), a new species of amphizoic amoeba isolated from gills of roach (Rutilus rutilus), and its Rickettsial endosymbiont. Folia Parasitol (Praha). 2003;50:161–70.

26. Galindo LJ, Torruella G, Moreira D, Eglit Y, Simpson AGB, Völcker E, et al. Combined cultivation and single-cell approaches to the phylogenomics of nucleariid amoebae, close relatives of fungi. Philos Trans R Soc B Biol Sci. 2019;374:20190094.

27. Pilgrim J, Ander M, Garros C, Baylis M, Hurst GDD, Siozios S. Torix group Rickettsia are widespread in Culicoides biting midges (Diptera: Ceratopogonidae), reach high frequency and carry unique genomic features. Environ Microbiol. 2017;19:4238–55.

28. Thongprem P, Davison HR, Thompson DJ, Lorenzo-Carballa MO, Hurst GDD. Incidence and diversity of Torix Rickettsia–Odonata symbioses. Microb Ecol. 2020; DOI:10.1007/s00248-020-01568-9

29. Weinert LA. The diversity and phylogeny of Rickettsia. In: Morand S, Krasnov BR, Littlewood DTJ, editors. Parasite diversity and diversification. Cambridge: Cambridge University Press; 2015. p. 150–81.

30. Lagrue C, Joannes A, Poulin R, Blasco-Costa I. Genetic structure and host-parasite co-divergence: evidence for trait-specific local adaptation. Biol J Linn Soc. 2016;118:344–58.

31. Řezáč M, Gasparo F, Král J, Heneberg P. Integrative taxonomy and evolutionary history of a newly revealed spider Dysdera ninnii complex (Araneae: Dysderidae). Zool J Linn Soc. 2014;172:451–74.

32. Ceccarelli FS, Haddad CR, Ramírez MJ. Endosymbiotic Rickettsiales (Alphaproteobacteria) from the spider genus Amaurobioides (Araneae: Anyphaenidae). J Arachnol. 2016;44:251–3.

33. Park E, Poulin R. Widespread Torix Rickettsia in New Zealand amphipods and the use of blocking primers to rescue host COI sequences. Sci Rep. 2020;10:16842.

34. Smith MA, Bertrand C, Crosby K, Eveleigh ES, Fernandez-Triana J, Fisher BL, et al. Wolbachia and DNA barcoding insects: Patterns, potential, and problems. PLoS One. 2012;7:e36514.

35. BOLD: Barcode of Life Data System. 2007. https://www.boldsystems.org/ Accessed 2 January 2018.

36. Smith MA, Pilgrim J, Zakharov E V., Dewaard JR, Ratnasingham S. BOLD contaminant pool (3,817 specimens) data. Figshare. 2020; DOI:10.6084/m9.figshare.12801107

37. Smith MA, Pilgrim J, Zakharov E V., Dewaard JR, Ratnasingham S. BOLD non-contaminant pool (55,366 specimens) data. Barcoding Of Life Data System. 2020; DOI:10.5883/DS-RICKET

38. Ratnasingham S, Hebert PDN. BOLD: The Barcode of Life Data System. Mol Ecol Notes. 2007;7:355–64.

39. Davison HR, Siozios S. Rickettsia PhyloFlash and Kraken data from arthropod whole genome projects in the Sequence Read Archive. Figshare. 2020; DOI:10.6084/m9.figshare.12801140

40. Gruber-Vodicka HR, Seah BK, Pruesse E. phyloFlash: Rapid small-subunit rRNA profiling and targeted assembly from metagenomes. mSystems. 2020; DOI:10.1128/mSystems.00920-20

41. Hernández-Triana LM, Prosser SW, Rodríguez-Perez MA, Chaverri LG, Hebert PDN, Ryan Gregory T. Recovery of DNA barcodes from blackfly museum specimens (Diptera: Simuliidae) using primer sets that target a variety of sequence lengths. Mol Ecol Resour. 2014;14:508–18.

42. Wang Z, Wu M. An integrated phylogenomic approach toward pinpointing the origin of mitochondria. Sci Rep. 2015;5:7949.

43. Goodacre SL, Martin OY, Thomas CFG, Hewitt GM. Wolbachia and other endosymbiont infections in spiders. Mol Ecol. 2006;15:517–27.

44. Martin OY, Puniamoorthy N, Gubler A, Wimmer C, Bernasconi M V. Infections with Wolbachia, Spiroplasma, and Rickettsia in the Dolichopodidae and other Empidoidea. Infect Genet Evol. 2013;13:317–30.

45. Machtelinckx T, Van Leeuwen T, Van De Wiele T, Boon N, De Vos WH, Sanchez J-A, et al. Microbial community of predatory bugs of the genus Macrolophus (Hemiptera: Miridae). BMC Microbiol. 2012;12:S9.

46. Lanzoni O, Sabaneyeva E, Modeo L, Castelli M, Lebedeva N, Verni F, et al. Diversity and environmental distribution of the cosmopolitan endosymbiont "Candidatus Megaira". Sci Rep. 2019;9:1179.

47. Blaxter M. Symbiont genes in host genomes: Fragments with a future? Cell Host Microbe. 2007;2:211-3.

48. Gehrer L, Vorburger C. Parasitoids as vectors of facultative bacterial endosymbionts in aphids. Biol Lett. 2012;8:613-5.

49. Le Clec'h W, Chevalier FD, Genty L, Bertaux J, Bouchon D, Sicard M. Cannibalism and predation as paths for horizontal passage of Wolbachia between terrestrial isopods.

50. Ramage T, Martins-Simoes P, Mialdea G, Allemand R, Duplouy A, Rousse P, et al. A DNA barcode-based survey of terrestrial arthropods in the Society Islands of French Polynesia: host diversity within the SymbioCode Project. Eur J Taxon. 2017;272.

51. Wang H, Lei T, Wang X, Maruthi MN, Zhu D, Cameron SL, et al. A newly recorded Rickettsia of the Torix group is a recent intruder and an endosymbiont in the whitefly Bemisia tabaci. Environ Microbiol. 2020;22:1207–21.

52. Caspi-Fluger A, Inbar M, Mozes-Daube N, Katzir N, Portnoy V, Belausov E, et al. Horizontal transmission of the insect symbiont Rickettsia is plant-mediated. Proc R Soc B Biol Sci. 2012;279:1791–6.

53. Gonella E, Pajoro M, Marzorati M, Crotti E, Mandrioli M, Pontini M, et al. Plant-mediated interspecific horizontal transmission of an intracellular symbiont in insects. Sci Rep. 2015;5:15811.

54. Li Y-H, Ahmed MZ, Li S-J, Lv N, Shi P-Q, Chen X-S, et al. Plant-mediated horizontal transmission of Rickettsia endosymbiont between different whitefly species. FEMS Microbiol Ecol. 2017;93.

55. Jaenike J, Polak M, Fiskin A, Helou M, Minhas M. Interspecific transmission of endosymbiotic Spiroplasma by mites. Biol Lett. 2007;3:23–5.

56. Morrow JL, Frommer M, Shearman DCA, Riegler M. Tropical tephritid fruit fly community with high incidence of shared Wolbachia strains as platform for horizontal transmission of endosymbionts. Environ Microbiol. 2014;16:3622–37.

57. Goodacre SL, Martin OY, Bonte D, Hutchings L, Woolley C, Ibrahim K, et al. Microbial modification of host long-distance dispersal capacity. BMC Biol. 2009;7:32.

58. Giorgini M, Bernardo U, Monti MM, Nappo AG, Gebiola M. Rickettsia symbionts cause parthenogenetic reproduction in the parasitoid wasp Pnigalio soemius (Hymenoptera: Eulophidae). Appl Environ Microbiol. 2010;76:2589–99.

59. Hagimori T, Abe Y, Date S, Miura K. The first finding of a Rickettsia bacterium associated with parthenogenesis induction among insects. Curr Microbiol. 2006;52:97–101.

60. Li K, Chen H, Jiang J, Li X, Xu J, Ma Y. Diversity of bacteriome associated with Phlebotomus chinensis (Diptera: Psychodidae) sand flies in two wild populations from China. Sci Rep. 2016;6:36406.

61. Reeves WK, Kato CY, Gilchriest T. Pathogen screening and bionomics of Lutzomyia apache (Diptera: Psychodidae) in Wyoming, USA. J Am Mosq Control Assoc. 2008;24:444–7.

62. Song S, Chen C, Yang M, Zhao S, Wang B, Hornok S, et al. Diversity of Rickettsia species in border regions of northwestern China. Parasit Vectors. 2018;11:634.

63. Floris R, Yurtman AN, Margoni EF, Mignozzi K, Boemo B, Altobelli A, et al. Detection and identification of Rickettsia species in the Northeast of Italy. Vector-Borne Zoonotic Dis. 2008;8:777–82.

64. Yan P, Qiu Z, Zhang T, Li Y, Wang W, Li M, et al. Microbial diversity in the tick Argas japonicus (Acari: Argasidae) with a focus on Rickettsia pathogens. Med Vet Entomol. 2019;33:327–35.

65. Potts R, Molina I, Sheele JM, Pietri JE. Molecular detection of Rickettsia infection in field-collected bed bugs. New Microbes New Infect. 2020;34:100646.

66. Parola P, Paddock CD, Raoult D. Tick-borne Rickettsioses around the world: Emerging diseases challenging old concepts. Clin Microbiol Rev. 2005;18:719–56.

67. Hoffmann AA, Ross PA, Rašić G. Wolbachia strains for disease control: ecological and evolutionary considerations. Evol Appl. 2015;8:751–68.

68. Iturbe-Ormaetxe I, Walker T, O' Neill SL. Wolbachia and the biological control of mosquito-borne disease. EMBO Rep. 2011;12:508–18.

69. van den Hurk AF, Hall-Mendelin S, Pyke AT, Frentiu FD, McElroy K, Day A, et al. Impact of Wolbachia on infection with chikungunya and yellow fever viruses in the mosquito vector Aedes aegypti. PLoS Negl Trop Dis. 2012;6.

70. Kliot A, Cilia M, Czosnek H, Ghanim M. Implication of the bacterial endosymbiont Rickettsia spp. in interactions of the whitefly Bemisia tabaci with Tomato yellow leaf curl virus. J Virol. 2014;88:5652–60.

71. Tedeschi R, Visentin C, Alam A, Bosco D. Epidemiology of apple proliferation (AP) in northwestern Italy: evaluation of the frequency of AP-positive psyllids in naturally infected populations of Cacopsylla melanoneura (Homoptera: Psyllidae). Ann Appl Biol. 2003;142:285-90.

72. Menzel P, Ng KL, Krogh A. Fast and sensitive taxonomic classification for metagenomics with Kaiju. Nat Commun. 2016;7:11257.

73. Katoh K, Standley DM. MAFFT Multiple sequence alignment software version 7: Improvements in performance and usability. Mol Biol Evol. 2013;30:772–80.

74. Castresana J. Selection of conserved blocks from multiple alignments for their use in phylogenetic analysis. Mol Biol Evol. 2000;17:540–52.

75. Kalyaanamoorthy S, Minh BQ, Wong TKF, von Haeseler A, Jermiin LS. ModelFinder: fast model selection for accurate phylogenetic estimates. Nat Methods. 2017;14:587–9.

76. Nguyen LT, Schmidt HA, Von Haeseler A, Minh BQ. IQ-TREE: A fast and effective stochastic algorithm for estimating maximum-likelihood phylogenies. Mol Biol Evol. 2015;32:268–74.

77. Hoang DT, Chernomor O, Haeseler A von, Minh BQ, Vinh LS. UFBoot2: Improving the ultrafast bootstrap approximation. Mol Biol Evol. 2017;35:518–22.

78. He Z, Zhang H, Gao S, Lercher MJ, Chen W-H, Hu S. Evolview v2: an online visualization and management tool for customized and annotated phylogenetic trees. Nucleic Acids Res. 2016;44:W236–41.

79. Fredslund J, Schauser L, Madsen LH, Sandal N, Stougaard J. PriFi: using a multiple alignment of related sequences to find primers for amplification of homologs. Nucleic Acids Res. 2005;33:W516–20.

80. Martin DP, Murrell B, Golden M, Khoosal A, Muhire B. RDP4: Detection and analysis of recombination patterns in virus genomes. Virus Evol. 2015;1:1–5.

81. Chernomor O, von Haeseler A, Minh BQ. Terrace aware data structure for phylogenomic inference from supermatrices. Syst Biol. 2016;65:997–1008.

82. Okonechnikov K, Golosova O, Fursov M. Unipro UGENE: a unified bioinformatics toolkit. Bioinformatics. 2012;28:1166–7.

83. Ewing B, Hillier L, Wendl MC, Green P. Base-calling of automated sequencer traces using Phred. I. Accuracy Assessment. Genome Res. 1998;8:175–85.

84. Wood DE, Lu J, Langmead B. Improved metagenomic analysis with Kraken 2. Genome Biol. 2019;20:257.

85. Kölsch G, Synefiaridou D. Shared ancestry of symbionts? Sagrinae and Donaciinae (Coleoptera, Chrysomelidae) harbor similar bacteria. Insects. 2012;3:473–91.

86. Li K, Stanojević M, Stamenković G, Ilić B, Paunović M, Lu M, et al. Insight into diversity of bacteria belonging to the order Rickettsiales in 9 arthropods species collected in Serbia. Sci Rep. 2019;9:18680.

87. Murakami T, Segawa T, Bodington D, Dial R, Takeuchi N, Kohshima S, et al. Census of bacterial microbiota associated with the glacier ice worm Mesenchytraeus solifugus. FEMS Microbiol Ecol. 2015;91.

88. Noda H, Watanabe K, Kawai S, Yukuhiro F, Miyoshi T, Tomizawa M, et al. Bacteriome-associated endosymbionts of the green rice leafhopper Nephotettix cincticeps (Hemiptera: Cicadellidae). Appl Entomol Zool. 2012;47:217–25.

89. Zheng Z, Wang D, He H, Wei C. Bacterial diversity of bacteriomes and organs of reproductive, digestive and excretory systems in two cicada species (Hemiptera: Cicadidae). PLoS One. 2017;12:e0175903.

90. Kobiałka M, Michalik A, Świerczewski D, Szklarzewicz T. Complex symbiotic systems of two treehopper species: Centrotus cornutus (Linnaeus, 1758) and Gargara genistae (Fabricius, 1775) (Hemiptera: Cicadomorpha: Membracoidea: Membracidae). Protoplasma. 2020;257:819–31.

91. Zouache K, Voronin D, Tran-Van V, Mavingui P. Composition of bacterial communities associated with natural and laboratory populations of Asobara tabida infected with Wolbachia. Appl Environ Microbiol. 2009;75:3755–64.

92. Gualtieri L, Nugnes F, Nappo AG, Gebiola M, Bernardo U. Life inside a gall: closeness does not favour horizontal transmission of Rickettsia between a gall wasp and its parasitoid. FEMS Microbiol Ecol. 2017;93.

93. Gerth M, Wolf R, Bleidorn C, Richter J, Sontowski R, Unrein J, et al. Green lacewings (Neuroptera: Chrysopidae) are commonly associated with a diversity of Rickettsial endosymbionts. Zool Lett. 2017;3:12.

94. Perotti MA, Clarke HK, Turner BD, Braig HR. Rickettsia as obligate and mycetomic bacteria. FASEB J. 2006;20:2372–4.

Figure Legends

Figure 1. Workflow of the BOLD project demonstrating the acquisition and fates of contaminant and non-contaminant *COI* barcoding sequences.

Figure 2. Cladogram of the maximum likelihood (ML) tree of 1,126 proteobacteria *COI* contaminants retrieved from a BOLD project incorporating 184,585 arthropod specimens. The tree is based on 561 bp and is rooted with the free-living alphaproteobacteria *Pelagibacter ubique*. Parentheses indicate the number of BOLD contaminants present in each group. Tips are labelled by BOLD processing ID and host arthropod taxonomy. The Rickettsiales genera of *Anaplasma*, *Rickettsia* (collapsed node), *Orientia* and *Wolbachia* supergroups (A, B, E and F), as well as the Legionellales genera *Legionella* and *Rickettsiella*, are included as reference sequences (Accession numbers: Additional file 10).

Figure 3. Cladogram of a maximum likelihood (ML) tree of 753 *COI Rickettsia* contaminants retrieved from a BOLD project incorporating 184,585 arthropod specimens. The tree is based on 561 bp and is rooted by the *Rickettsia* endosymbiont of *Ichthyophthirius multifiliis* (Candidatus Megaira) using the TVM+F+I+G4 model. Parentheses indicate the number of BOLD contaminants present in Torix and non-Torix *Rickettsia* groups. Tips are labelled by BOLD processing ID and host arthropod taxonomy. The *Rickettsia* groups: Spotted fever, Transitional, Belli, Typhus, Rhyzobius and Torix are included as references (Accession numbers: Additional file 10).

Figure 4. Phylogram of the maximum likelihood (ML) tree of 99 *COI Rickettsia* contaminants (prefix "BIOUG") used for further phylogenetic analysis and 53 Non-BOLD reference profiles (Accession numbers: Additional file 10). The tree is based on the concatenation of 4 loci; *16S rRNA*, *17KDa*, *gltA* and *COI* under a partition model, with profiles containing at least 3 out of 4 sites included in the tree (2,834 bp total) and is rooted by *Rickettsia* endosymbiont of *Ichthyophthirius multifiliis* (*Candidatus* Megaira). Tips are labelled by host arthropod taxonomy.

Figure 5. *16S rRNA* and *gltA* concatenated maximum likelihood (ML) phylogram (1,834 bp total) including *Rickettsia* hosts from SRA (Triangles) and targeted screens (Stars). The TIM3+F+R2 (16S) and K3Pu+F+G4 (gltA) models were chosen as best fitting models. Rooting is with *Orientia tsutsugamushi*. Accession numbers found in Additional file 10.

Figure 6. Phylogram of a maximum likelihood (ML) tree of *COI Rickettsia* contaminants (prefix "BIOUG") giving a host barcode and 43 Non-BOLD reference profiles. The tree is based on 4 loci; *16S rRNA*, *17KDa*, *gltA* and *COI* under a partition model with profiles containing at least 2 out of 4 sites included in the tree (2,781 bp total) and is rooted by the *Rickettsia* endosymbiont of *Ichthyophthirius multifiliis* (*Candidatus* Megaira). The habitats and lifestyles of the host are given to the right of the phylogeny. Accession numbers found in Additional file 10.

Additional file information

Additional file 1.docx Taxonomic classification of BOLD non-target *COI* sequences via Kaiju.

Additional file 2.7z Rectangular phylogram trees of cladograms from Figures 2 and 3.

Additional file 3.docx Primer pairs involved in the unintended amplification of 753 *Rickettsia COI* from BOLD project.

Additional file 4.docx Homology of *Rickettsia* groups and *Wolbachia* to the most common forward primers (C_LepFolF and C_LepFolR) attributed to bacterial *COI* amplification from arthropod DNA extracts.

Additional file 5.xlsx Re-barcoding status and nearest BLAST hit of mtDNA *COI* arthropod DNA extracts accessed for further analysis, along with the success of multilocus *Rickettsia* profiles with allocated *Rickettsia* group (based on phylogenetic analysis) and co-infection status.

Additional file 6.docx The barcoding success rate of taxa which gave at least one bacteria *COI* inadvertent amplification (N=51,475 accessible specimens) with an adjusted *Rickettsia* frequency based on an estimated total number of arthropods to account for inaccessible specimens (N=125,402).

Additional file 7.docx Fisher's Exact analyses for comparison of Torix *Rickettsia* infection in aquatic versus terrestrial insects.

A**dditional file 8.docx** GenBank matches mistaken for true mtDNA barcodes and their homology to *Rickettsia COI* (Accessed 29th June 2020).

Additional file 9.pdf Phylogram of a maximum likelihood (ML) tree of *COI Rickettsia* found in the GenBank database erroneously identified as mtDNA barcodes based on 577 bp. The HKY+F+G4 model was chosen as the best fitting model using Modelfinder with the Bayesian information criterion (BIC).

Additional file 10.xlsx Accession numbers used for phylogenetic analyses (Figures 2, 3, 4 ,5 and 6). Accession numbers generated in this study are marked in BOLD.

Additional file 11.docx Mitochondrial *COI* and bacterial gene primers used for re-barcoding and multilocus phylogenetic analyses.

Figure 1

Click here to access/download;Figure;Figure 1.tif

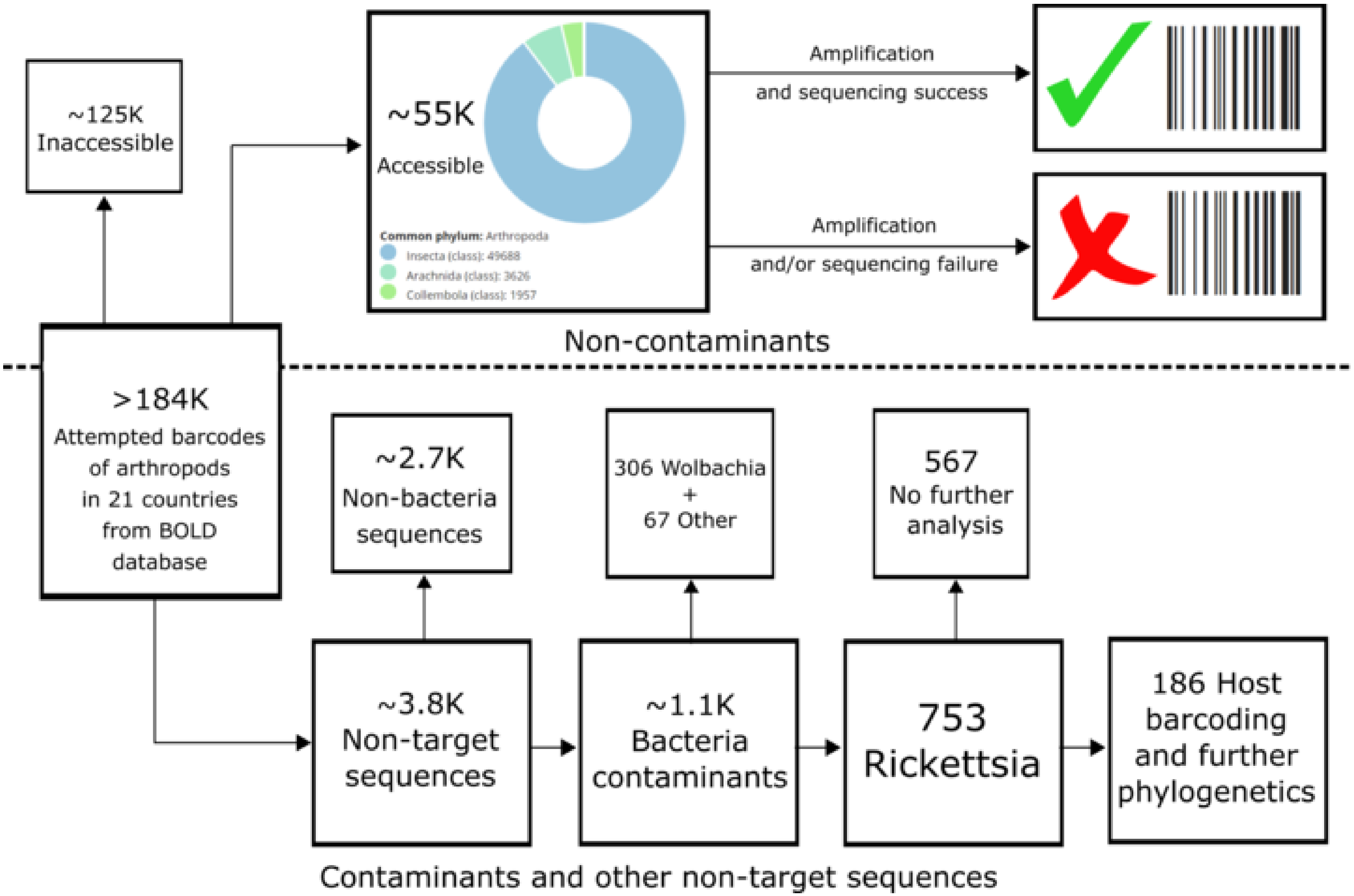

Figure 2

Click here to access/download;Figure;Figure 2.png

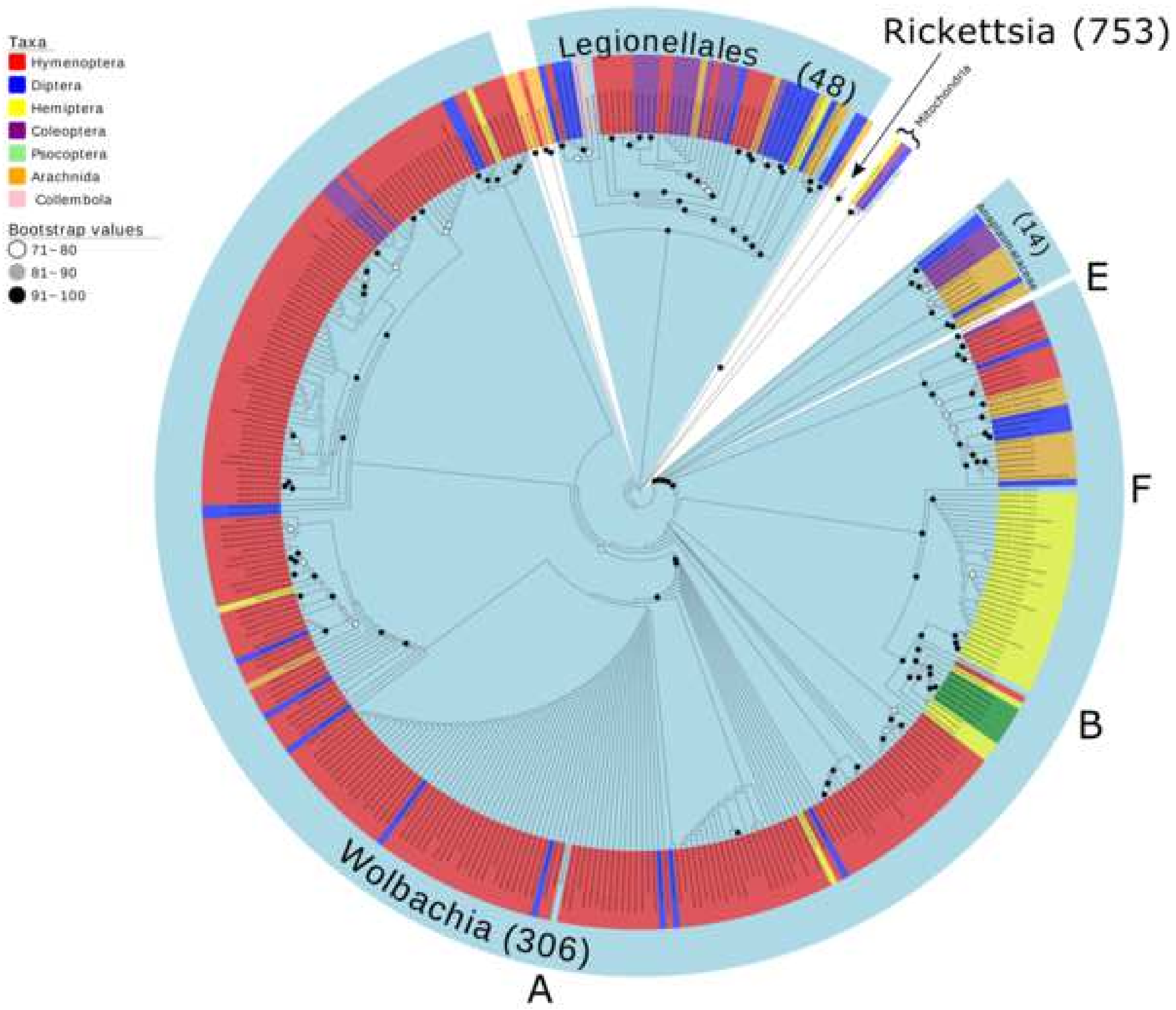

Figure 3

Click here to access/download;Figure;Figure 3.png

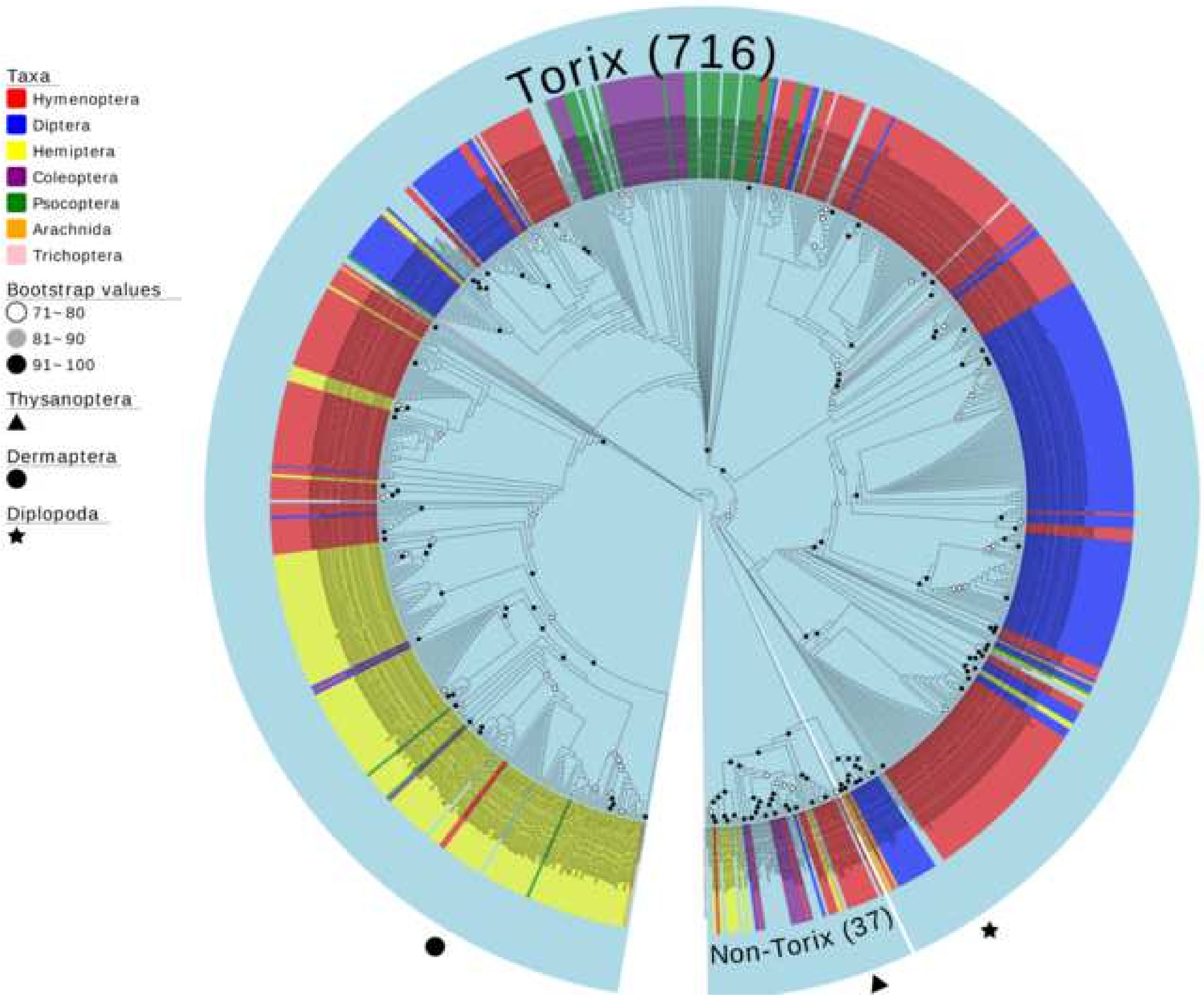

Figure 4

Click here to access/download;Figure;Figure 4.png

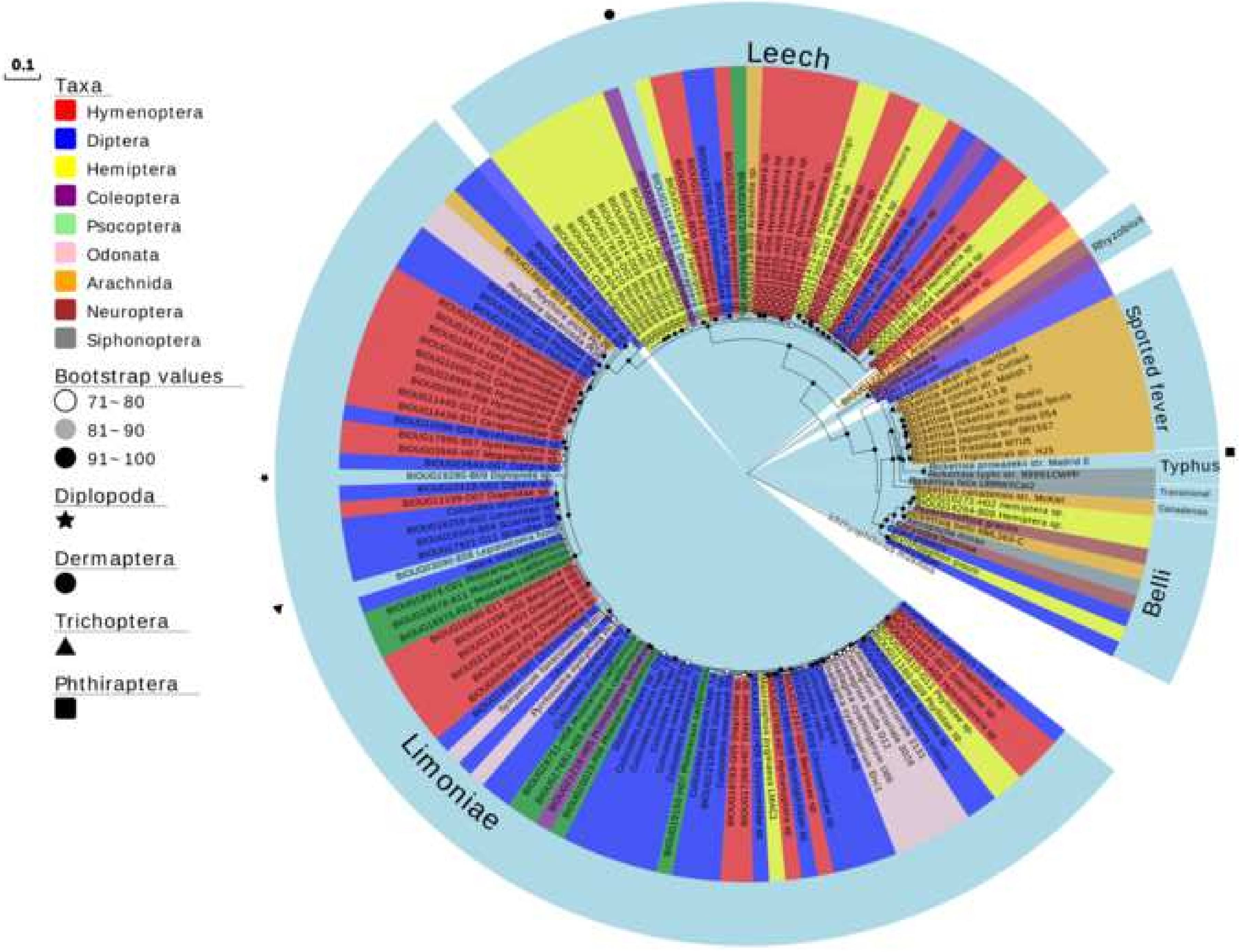

Click here to access/download;Figure;Figure 5 annotated 2.png

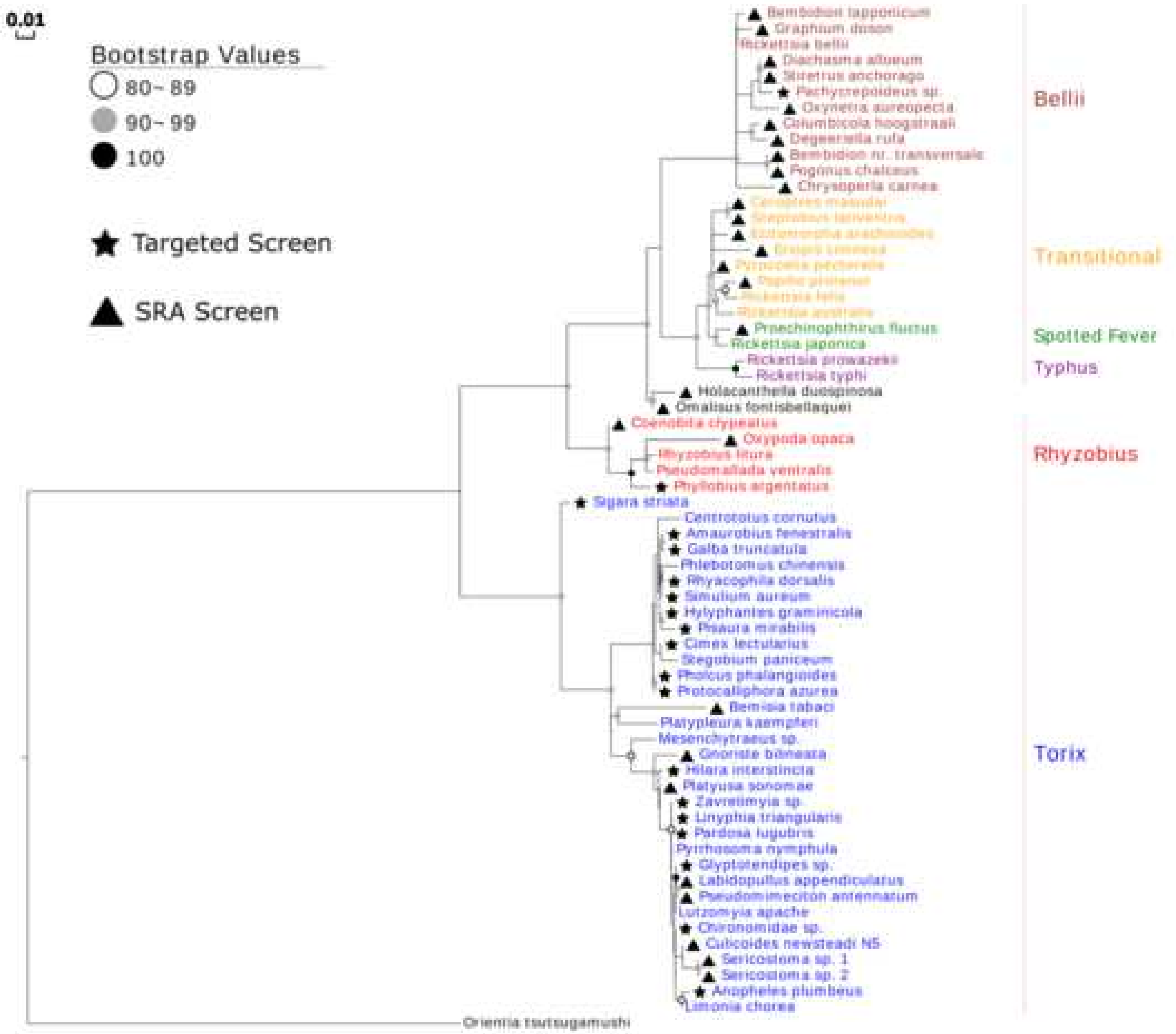

Figure 6

Click here to access/download;Figure;Figure 6.png

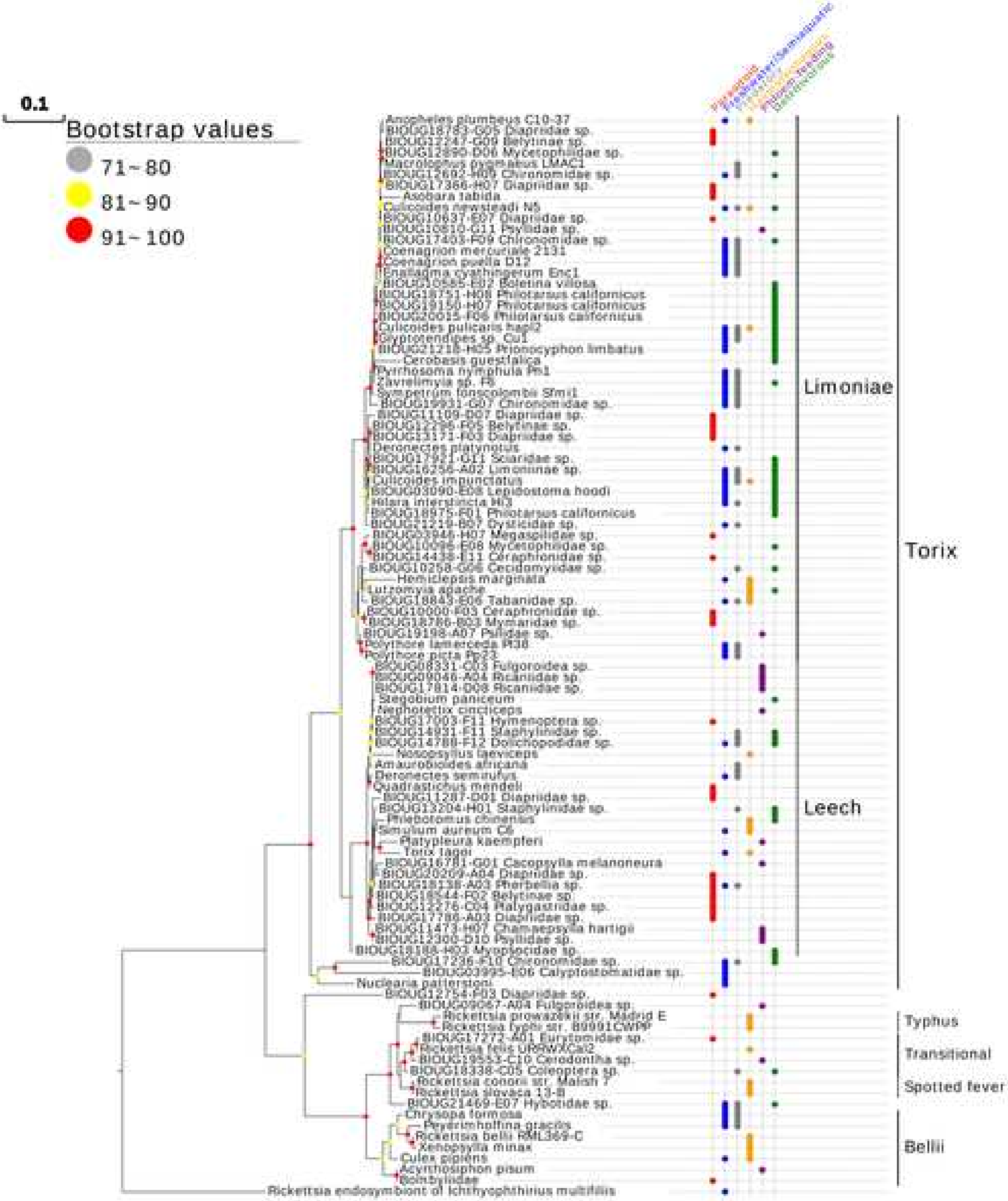

Additional file 1

Click here to access/download
Supplementary Material
Additional file 1.docx

Additional file 2

Click here to access/download
Supplementary Material
Additional file 2.7z

Additional file 3

Click here to access/download
Supplementary Material
Additional file 3.docx

Additional file 4

Click here to access/download
Supplementary Material
Additional file 4.docx

Additional file 5

Click here to access/download
Supplementary Material
Additional file 5.xlsx

Additional file 6

Click here to access/download
Supplementary Material
Additional file 6.docx

Additional file 7

Click here to access/download
Supplementary Material
Additional file 7.docx

Additional file 8

Click here to access/download
Supplementary Material
Additional file 8.docx

Additional file 9

Click here to access/download
Supplementary Material
Additional file 9.pdf

Additional file 10

Click here to access/download

Supplementary Material

Additional file 10.xlsx

Additional file 11

Click here to access/download
Supplementary Material
Additional file 11.docx
