# Supplementary material for: Torix Rickettsia are widespread in arthropods and reflect a neglected symbiosis
Source: Gigascience. 2021 Mar 25;10(3):giab021. doi: 10.1093/gigascience/giab021 (PMC7992394; doi:10.1093/gigascience/giab021)
Supplement: giab021_GIGA-D-20-00244_Revision_2 [file giab021_giga-d-20-00244_revision_2.pdf]

GigaScience

Torix Rickettsia are widespread in arthropods and reflect a neglected symbiosis

--Manuscript Draft--

**Manuscript Number:**	GIGA-D-20-00244R2
**Full Title:**	Torix Rickettsia are widespread in arthropods and reflect a neglected symbiosis
**Article Type:**	Research
**Funding Information:**	Biotechnology and Biological Sciences Research Council (BB/M011186/1) — Dr. Jack Pilgrim<br>Natural Environment Research Council (NE/L002450/1) — Ms. Helen R. Davison
**Abstract:**	Background<br><br>Rickettsia are intracellular bacteria best known as the causative agents of human and animal diseases. Although these medically important Rickettsia are often transmitted via haematophagous arthropods, other Rickettsia , such as those in the Torix group, appear to reside exclusively in invertebrates and protists with no secondary vertebrate host. Importantly, little is known about the diversity or host range of Torix group Rickettsia .<br><br>Results<br><br>This study describes the serendipitous discovery of Rickettsia amplicons in the Barcode of Life Data System (BOLD), a sequence database specifically designed for the curation of mtDNA barcodes. Out of 184,585 barcode sequences analysed, Rickettsia is observed in approximately 0.41% of barcode submissions and is more likely to be found than Wolbachia (0.17%). The Torix group of Rickettsia are shown to account for 95% of all unintended amplifications from the genus. A further targeted PCR screen of 1,612 individuals from 169 terrestrial and aquatic invertebrate species identified mostly Torix strains and supports the ‘aquatic hot spot’ hypothesis for Torix infection. Furthermore, the analysis of 1,341 Sequence Read Archive (SRA) deposits indicates Torix infections represent a significant proportion of all Rickettsia symbioses found in arthropod genome projects.<br><br>Conclusions<br><br>This study supports a previous hypothesis which suggests Torix Rickettsia are overrepresented in aquatic insects. In addition, multiple methods reveal further putative hot spots of Torix Rickettsia infection; including in phloem-feeding bugs, parasitoid wasps, spiders, and vectors of disease. The unknown host effects and transmission strategies of these endosymbionts make these newly discovered associations important to inform future directions of investigation involving the understudied Torix Rickettsia .
**Corresponding Author:**	Jack Pilgrim<br>University of Liverpool Institute of Infection Veterinary and Ecological Sciences<br>Neston, Cheshire, Liverpool UNITED KINGDOM
**Corresponding Author Secondary Information:**	
**Corresponding Author's Institution:**	University of Liverpool Institute of Infection Veterinary and Ecological Sciences
**Corresponding Author's Secondary Institution:**	
**First Author:**	Jack Pilgrim
**First Author Secondary Information:**	
**Order of Authors:**	Jack Pilgrim<br>Panupong Thongprem<br>Helen R. Davison

Powered by Editorial Manager® and ProduXion Manager® from Aries Systems Corporation

	Stefanos Siozios
	Matthew Baylis
	Evgeny V. Zakharov
	Sujeevan Ratnasingham
	Jeremy R. deWaard
	Craig R. Macadam
	M. Alex Smith
	Gregory D. D. Hurst
**Order of Authors Secondary Information:**	
**Response to Reviewers:**	We would like to thank the reviewer for their comments. Please find below a point-by-point response from the authors.<br><br>“The alignments seem to interchange N/n/-, but I checked the IQTree manual and IQTree treats them all the same. But I don't see how IQ tree handles phylogenetic inference at positions with N/n/-, which it calls "unknowns". For example, in the multigene alignment, I don't believe there is any position without gaps, so presumably it handles them. I'm assuming the alignments I'm looking at are the ones fed into IQTree and not the ones coming out of MAFFT and before Gblocks, but it might be good for you to confirm using this example: looking at the alignment BOLD_just_Rciekttsia_COI_contaminants_alignment.fas, these two sequences have absolutely no overlap, and are significant truncated relative to the complete alignment (and these aren't the only two with this problem). How is the algorithm dealing with this, and does it introduce any artifacts? Do you get the same result if you remove sequences like these and use only sequences where all positions of the alignment have a<br>character (both by removing short sequences like this and also trimming the ends of the alignment)?”<br><br>The reasoning behind including sequences with missing character data in our alignments is based on previous work demonstrating that missing data in most cases should not decrease phylogenetic resolution for taxa with complete data (Wiens 2006, DOI: 10.1016/j.jbi.2005.04.001). To confirm this, we reran a modified alignment of the BOLD_just_Rickettsia_COI_contaminants_alignment.fas file by trimming ends by 50 nucleotides and removing any remaining truncated sequences to get rid of missing data (169 of the original 807 sequences removed), as suggested by the reviewer. In accordance with Wiens’ observations, the generated tree (see FTP file ‘BOLD Rickettsia trimmed.png’) placed taxa into the same designated groups as the phylogeny with missing data in Figure 3 (Designations can be found at DOI:10.6084/m9.figshare.12801107). Additionally, the study cited above ran simulations to show that highly incomplete data can be accurately placed in phylogenetic trees as long as at least 50% of sequences contain complete data. Furthermore, it is suggested that the inclusion of sequences with missing data can also sometimes be better than exclusion as this additional data can subdivide misleading long branches. We have now included the reasoning behind using incomplete data in the methods section (lines 480-483).<br><br>“How do double infections confound the results? Do they behave erratically in the cladograms, like a chimera would? Can this be a wider problem in interpreting the results? It seems like it would have to be, but you have chimeras you can use to examine this. Using those, is there a way to address this? With SRA data it seems like you should be able to look for sequence heterogeneity in the reads. Not sure about BOLD data.”<br><br>Where double peaks were observed in 10/753 Rickettsia-associated taxa from BOLD, the base call was designated as ‘N’ (See FTP file ‘BOLD_multigene_Rickettsia_alignment.fas’). This prevents erroneous placement of chimeric strains on the phylogeny. For BOLD data we unfortunately cannot reconstruct trees by teasing apart the individual strains of the double-infections because we cannot know what phase the double-peaks are in.

The use of ‘N’ characters at double-peak sites could lead to potential problems in the interpretation of these 10 taxa at terminal branches of the phylogeny but the placement as Torix Rickettsia is not likely to be affected. Furthermore, these double-infections are a minority of the total taxa meaning their effects on interpreting results are likely to be minimal.

“Line 254-261: I think you need to add in a correction for multiple testing. You do at least two tests that are in the main text, but it sounds from the response to reviewer's comments that you did many more than that and are only reporting the ones that are P<0.05. However, you should report how many tests you did to find those two and adjust for multiple testing. Otherwise, if you do 20 tests, you would expect to have 1 that is "significant" (for more information on multiple testing: http://www.biostathandbook.com/multiplecomparisons.html). In addition, I think it is important for people to know what comparisons were done that were not significant as these are also results. Addressing multiple testing seems like an issue throughout. In the methods other statistical tests were clearly undertaken where there was multiple testing.”

Two Fisher’s exact tests (aquatic vs terrestrial insects-1 controlled for insect order and 1 uncontrolled) were detailed in the main text and additional file 7, as these were the only taxonomically ‘matched’ pairs. However, one additional test was performed initially to compare terrestrial vs aquatic invertebrates in general which did not give a significant p-value due to a hotspot of Rickettsia in spiders, which are known to be a hotspot for all inherited symbionts tested to date (Wolbachia, Spiroplasma, Rickettsia: Goodacre et al. 2006, doi: 10.1111/j.1365-294X.2005.02802.x.; Cardinium: Duron et al. 2008, doi: 10.1111/j.1365-294X.2008.03689.x.). This detail has now been added in Additional file 7 and lines 266-269. Overall, only 3 tests were done (2 significant and 1 not significant) and this indicates that Torix Rickettsia are over-represented in aquatic insects but this may not be the case for invertebrates in general.

“Line 354: Several, maybe many, of the Wolbachia integrations have no mutations or frameshifts, particularly in insects. Those with frameshifts and mutations are easier to find and identify as integrations such that the number of integrations without frameshifts and mutations is likely an underestimate, particularly given how many groups are still screening Wolbachia sequences out before assembling insect genomes. I have no idea how often that happens with Rickettsia, but it seems like, particularly as more groups use tools like blobplots.”

We thank the reviewer for raising this issue. We have now put a caveat at the end of this sentence to indicate that despite no frameshifts or mutations, it is still possible the sequences from this study are host integrations (lines 376-378).
The problem is likely to be less for Rickettsia than Wolbachia, due to differences in the mode of vertical transmission. Wolbachia is present in the germline stem cell niche, such DNA from the symbiont is available for incorporation into the germline. Rickettsia, in contrasts, usually invades the egg after meiosis, through the follicular epithelium. Thus, Rickettsia DNA is much less present in the germline of insects, making integration less likely.

“Line 366-379: This section still has issues with respect to the study design being secondary data analysis. These lines are in the discussion, it is the time to say things like on line 377 that the over-representation here in BOLD data (if that is the data you are referring to, because I can't remember which one was 17/19 and there is not here that clarifies) could be the result of an amplification bias—in not producing the host copy of the gene, amplifying the Rickettsia gene, or both. Those issues are profound in secondary data usage and need to be addressed head-on so that others who read the paper do not misconstrue the results. Likewise, the SRA data is not random, so I am not sure the statement on line 379-381 is correct, and at very least it needs qualifications. If it is correct, you need to better argue in the manuscript why it is correct, like that you used a sampling scheme to reduce bias, or something like that. Personally, I think it is better to acknowledge
the limitations that try to justify, as even if you have a sampling scheme, it can be biased. The PCR screen listed in the table of this manuscripts seems biased from my

quick look (e.g. an over-representation of mosquitoes).”

The “17/19 strains” being Torix is a reference to the targeted screen (not the BOLD screen) which was used alongside the BOLD data because of the aforementioned biases relating to amplification bias and this has now been clarified on lines 401-402. Additionally, we have added a sentence to the results section explicitly quoting the 17 strains of Rickettsia found in the targeted screen (lines 248-250). Although 95% of Rickettsia amplifications from BOLD are Torix, we already mention that this is likely due to primer bias (lines 321-324). Subsequently, the targeted screen is used in part to negate the problems of relying entirely on secondary data.
Of course, many studies which aim to investigate the distribution of a symbiont will have sampling and methodological biases. However, having multiple screening strategies, as we have here, is likely to give a more nuanced and holistic view of Torix Rickettsia ecology. We believe that the combined use of several screening methods is a strength and not a weakness of the study. Despite this, we have now added a separate section detailing the limitations of the study (lines 358-388).

Specifically, regarding lines 379-381 (of the 1st revision), this statement is based not just on SRA data but also the targeted screen from this study and Weinert’s study as mentioned in the previous lines. Thus, the SRA is corroborating two separate targeted screens (one which lacked spiders and aquatic insects demonstrating a high number of Belli infections, and another which included spiders and aquatic insects demonstrating a high number of Torix infections.). Subsequently, for clarity we have now changed the statement “Our additional use of a bioinformatics approach based on the SRA appears to confirm that Belli and Torix are two of the most common Rickettsia groups among arthropods.” to “Our additional use of a bioinformatics approach based on the SRA appears to corroborate targeted screen data indicating that Belli and Torix are two of the most common Rickettsia groups among arthropods.” (lines 403-406).

“Line 387-388: please provide more details. I don't remember reading that. Pointing to an exact result, for instance of how many strains of the same MLST type are in different insect orders is necessary. It should have been in the results if it is in the discussion. In fact, if I look at the figures, the Wolbachia in Figure 2 actually seem to be grouped by insect host taxa at this level. The same is ture for Figure 3 for the Rickettsia. There are a few interleaved colors, but without knowing more I'm not convinced that it can't be explained in another way (like a mite on a host or in the gut from a carnivorous insect or even a double infection); I also can't tell which ones are identical and which ones are just similar. But even if I should infer it from the figures, it should be reported in the results and I didn't find it there. Maybe you are trying to state it in the subsequent sentence if one assumes that all blood feeders are the same taxa and all phloem-feeders are
the same taxa, but that isn't clear. (And at least blood feeding is a trait found in multiple diverse taxa).”

The inferences related to similar strains in distantly-related hosts is best observed in the multigene tree in figure 4 rather than the single gene trees of figures 2 and 3. For example, odonate strains are clearly interleaved between strains from other host orders. More specifically, the two Coenagrion strains have 100% identity to the Culicoides stigma strain in contrast to two other odoante (Polythore) strains where multiple SNPs are observed at all loci (See ftp file ‘BOLD_multigene_Rickettsia_alignment.fas’). We thank the reviewer as this was not mentioned in the results but we have now included this on lines 209-211. Furthermore, regardless of exact MLST profiles for strains, taxa from most orders are represented in both Limoniae and Leech Torix subclades indicating a lack of grouping based on insect host taxa. The authors believe this concept is better represented in a phylogeny rather than a list of MLST profiles.

“And once again, I'm left wondering if there is a sampling bias. Are mosquitoes over-represented in the database? It some of the tables they seem over-sampled. Blood feeders and phloem feeders are often well sampled, given their important to human health and agriculture, respectively. But maybe more problematically, these results are being described but they are not clearly described in the results section. If I search for blood, I do not find any results that support this statement. When I search for phloem, there is a mention of them being found in phloem-feeding insects, but not that they are

Powered by Editorial Manager® and ProduXion Manager® from Aries Systems Corporation

	diverse, and I have no way of assessing that as a reviewer or reader. Yet, this result for phloem-feeding is also in the abstract as a taxa that is a hot-spot. I don't see it in the figures in a way that I understand (e.g. phloem-feeding isn't annotated). Additionally, there is no assessment here that convinces me it is a hot spot; there are no statistics to suggest it is overabundant, which would be required to be a hotspot (and any such statistics would need correction for multiple testing or some sort of FDR calculation)."<br><br>Mosquitoes are likely to be over-represented in the sequence read archive but as mentioned already in lines 142-144, a single dataset per species was extracted for analysis to negate oversampling of the same species. Although certain genera may still be oversampled, the only instance of mosquito Rickettsia being detected is in the Anopheles plumbeus population of the targeted screen. With regards to phloem-feeding insect strains, psyllids and other phloem-feeders are present in both Limoniae and Leech subclades suggesting again that strains are diverse within similar lifestyles. This is best seen in Figure 6 where both phloem-feeding and blood-feeding are annotated. The common patterns of infection in phloem-feeding bugs and blood-feeders are also already mentioned in lines 296-302 of the results. We agree that the common patterns or 'hot-spots' found in our data should come with caveats and we have now included this in the limitations part of the discussion where we clarify that common patterns of infection refer specifically to our datasets which although extensive, have some biases and may not completely represent Torix Rickettsia infection in nature (lines 358-371).<br><br>""as previously described" Is this in a different manuscript, or earlier in the manuscript? I suspect this means earlier in the paper, but it needs to be clear. Was the same alignment method used with MAFFT and Gblocks? Same for ModelFinder? If it is and all ML trees were inferred the same way, I would recommend that you have a methods section that describes this one for all alignments, maybe concluding with what is different (like the model)."<br>This refers to methods described earlier in the manuscript and yes, the same methods were used for all ML trees. This suggestion is welcomed and has been included in lines 492-493.<br><br>"I've outlined some examples where the statements don't reflect what is presented in the results and the limitations of a secondary data analysis. But they are actually more numerous and pervasive than this. For instance, line 39-41 and 41-43 in the abstract have these issues. Likewise, Line 161-162 should read "Torix Rickettsia is the most common bacterial contaminant sequence currently in BOLD, a major barcoding project". This change reflects that this only holds for what has been barcoded thus far, and the issues with the fact you need both failed host amplification and successful bacterial amplification, and that the biodiversity represented in such projects have their own biases. It is so pervasive, I am not sure I found all the instances. Honestly, I think the paper would really benefit from a large clearly labeled section that more explicitly deals with all the limitations of the study, so others do not misconstrue the results for years into the future. It would make<br>the paper much stronger and definitely more rigorous."<br><br>With regard to line 39-41 describing how our targeted PCR data supports the aquatic hotspot hypothesis we refer the reviewer to our response to the 'multiple testing' above. For lines 41-43, we have changed this sentence to include the caveat that this applies only to arthropod genome projects: "Furthermore, the analysis of 1,341 Sequence Read Archive (SRA) deposits indicates Torix infections represent a significant proportion of all Rickettsia symbioses found in arthropod genome projects." We have also changed lines 161-162 to reflect a similar caveat for the BOLD data as suggested by the reviewer. As previously mentioned, we have now included a specific section in the discussion detailing the limitations of our datasets (lines 358-388).
**Additional Information:**	
**Question**	**Response**
Are you submitting this manuscript to a special series or article collection?	No

**Experimental design and statistics**<br><br>Full details of the experimental design and statistical methods used should be given in the Methods section, as detailed in our Minimum Standards Reporting Checklist. Information essential to interpreting the data presented should be made available in the figure legends.<br><br>Have you included all the information requested in your manuscript?	Yes
**Resources**<br><br>A description of all resources used, including antibodies, cell lines, animals and software tools, with enough information to allow them to be uniquely identified, should be included in the Methods section. Authors are strongly encouraged to cite Research Resource Identifiers (RRIDs) for antibodies, model organisms and tools, where possible.<br><br>Have you included the information requested as detailed in our Minimum Standards Reporting Checklist?	Yes
**Availability of data and materials**<br><br>All datasets and code on which the conclusions of the paper rely must be either included in your submission or deposited in publicly available repositories (where available and ethically appropriate), referencing such data using a unique identifier in the references and in the “Availability of Data and Materials” section of your manuscript.<br><br>Have you have met the above requirement as detailed in our Minimum Standards Reporting Checklist?	Yes

Manuscript

Click here to access/download;Manuscript;Torix Rickettsia-forupload .docx

Torix *Rickettsia* are widespread in arthropods and reflect a neglected symbiosis

Jack Pilgrim[1*], Panupong Thongprem[1], Helen R. Davison[1], Stefanos Siozios[1], Matthew Baylis[1,2], Evgeny V. Zakharov[3], Sujeevan Ratnasingham[3], Jeremy R. deWaard[3], Craig R. Macadam[4], M. Alex Smith[5], Gregory D. D. Hurst[1]

1. Institute of Infection, Veterinary and Ecological Sciences, Faculty of Health and Life Sciences, University of Liverpool, Liverpool, U.K.

2. Health Protection Research Unit in Emerging and Zoonotic Infections, Liverpool, U.K.

3. Centre for Biodiversity Genomics, University of Guelph, Guelph, Ontario, Canada.

4. Buglife – The Invertebrate Conservation Trust, Balallan House, 24 Allan Park, Stirling, U.K.

5. Department of Integrative Biology, University of Guelph, Guelph, Ontario, Canada.

* Corresponding author

Emails:

JP: jack.pilgrim@liverpool.ac.uk

PT: pongthq@liverpool.ac.uk

HRD: hlhdavi5@liverpool.ac.uk

SS: sioziosss@liverpool.ac.uk

MB: baylism@liverpool.ac.uk

EVZ: zakharov@uoguelph.ca

SR: sratnasi@uoguelph.ca

JRD: dewaardj@uoguelph.ca

CRM: craig.macadam@buglife.org.uk
MAS: salex@uoguelph.ca
GDDH: ghurst@liverpool.ac.uk

ORCID IDs:
Jack Pilgrim: 0000-0002-2941-1482; Panupong Thongprem: 0000-0001-6542-235X; Helen Davison: 0000-0002-4302-5756; Stefanos Siozios: 0000-0002-1104-7061; Matthew Baylis; Sujeevan Ratnasingham: 0000-0002-3694-0171; Gregory Hurst: 0000-0002-7163-7784

Abstract

Background: *Rickettsia* are intracellular bacteria best known as the causative agents of human and animal diseases. Although these medically important *Rickettsia* are often transmitted via haematophagous arthropods, other *Rickettsia*, such as those in the Torix group, appear to reside exclusively in invertebrates and protists with no secondary vertebrate host. Importantly, little is known about the diversity or host range of Torix group *Rickettsia*.

Results: This study describes the serendipitous discovery of *Rickettsia* amplicons in the Barcode of Life Data System (BOLD), a sequence database specifically designed for the curation of mtDNA barcodes. Out of 184,585 barcode sequences analysed, *Rickettsia* is observed in approximately 0.41% of barcode submissions and is more likely to be found than *Wolbachia* (0.17%). The Torix group of *Rickettsia* are shown to account for 95% of all unintended amplifications from the genus. A further targeted PCR screen of 1,612 individuals from 169 terrestrial and aquatic invertebrate species identified mostly Torix strains and supports the 'aquatic hot spot' hypothesis for Torix infection. Furthermore, the analysis of 1,341 Sequence Read Archive (SRA) deposits indicates Torix infections represent a significant proportion of all *Rickettsia* symbioses found in arthropod genome projects.

Conclusions: This study supports a previous hypothesis which suggests Torix *Rickettsia* are overrepresented in aquatic insects. In addition, multiple methods reveal further putative hot spots of Torix *Rickettsia* infection; including in phloem-feeding bugs, parasitoid wasps, spiders, and vectors of disease. The unknown host effects and transmission strategies of these endosymbionts make these newly discovered associations important to inform future directions of investigation involving the understudied Torix *Rickettsia*.

Keywords: Rickettsia; symbiosis: arthropods; endosymbiont; DNA barcoding

Background

It is now widely recognized that animals live in a microbial world, and that many aspects of animal biology, ecology and evolution are a product of their symbioses with microorganisms [1]. In invertebrates, these symbioses may be particularly intimate, and involve transmission of the microbe from parent to offspring [2]. The alignment of host reproduction with symbiont transmission produces a correlation between the fitness interests of the parties, reflected in symbionts evolving to play a number of physiological roles within the host, from defence [3,4] through to core anabolic and digestive functions [5,6]. However, the maternal inheritance of these microbes has led to the retention of parasitic phenotypes associated with distortion of reproduction, with symbiont phenotypes including biases towards daughter production and cytoplasmic incompatibility [7]. These diverse individual impacts alter the ecology and evolution of the host, in terms of diet, dynamics of interaction with natural enemies, sexual selection and speciation.

Heritable symbioses have evolved on multiple occasions amongst microbial taxa. In some cases, the microbial lineage is limited to a single clade of related animal hosts, such as *Buchnera* in aphids [8]. In other cases, particular heritable microbes are found across a wide range of arthropod species. *Wolbachia* represents the most common associate, considered to infect nearly half of all species [9], and this commonness is a function in part of the ability of *Wolbachia* to transfer to a broad range of new host species and spread within them (host shift events) [10]. Aside *Wolbachia*, other microbes are found commonly as heritable symbionts of arthropod hosts [11]. *Cardinium* and *Rickettsia*, for instance, have been estimated at being present in 13-55% and 20-42% of terrestrial arthropod species respectively [12].

In this paper, we address the diversity and commonness of symbioses between *Rickettsia* and arthropods. The *Rickettsia* have increasingly been recognized as a genus of bacteria with diverse interactions with arthropods [13,14]. First discovered as the agents underlying several diseases of humans vectored by haematophagous arthropods [15,16], our understanding of the group changed in the 1990s with the recognition that *Rickettsia* were commonly arthropod symbionts [17,18]. *Rickettsia* were recognized first as male-killing reproductive parasites [17,19] and then later as beneficial partners [3,20,21].

Following this extension of our understanding of *Rickettsia*-arthropod interactions, a new clade of *Rickettsia* was discovered from work in *Torix* leeches [22,23]. This clade was sister to all other *Rickettsia* genera and contained two subgroups (Leech and Limoniae [24]), with no evidence to date of any strain having a vertebrate pathogen phase. The host range for Torix *Rickettsia* is broader than that for other members of the genus, going beyond arthropods to include amoeba hosts [25,26]. Targeted PCR based screening have revealed Torix group *Rickettsia* as particularly common in three groups with aquatic association: *Culicoides* biting midges, deronectid beetles and odonates [24,27,28]. However, some previous hypothesis-free PCR screens that aimed to detect *Rickettsia* in arthropods have likely missed these symbioses, due to divergence of the marker sequence and mismatch with the primers [29].

During our previous work on Torix *Rickettsia* in biting midges [27], we became aware of the presence of *Rickettsia* cytochrome *c* oxidase I (*COI*) sequences deposited in GenBank that derived from studies where the intended target of amplification/sequencing was

mitochondrial *COI*. These deposits derived from studies using mtDNA barcoding for phylogeographic inference [30], or in barcoding based species identification approaches [31,32]. Non-target amplification of *Rickettsia COI* using mitochondrial *COI* barcoding primers has been reported in spiders [31,32] and freshwater amphipods [30,33]. Furthermore, we have noted two cases in our lab where amplicons obtained for mtDNA barcoding of an arthropod have, on sequence analysis, revealed *Rickettsia COI* amplification (Belli group *Rickettsia* from Collembola, and Torix group *Rickettsia* from *Cimex lectularius* bedbugs). Previous work had established barcoding approaches may amplify *COI* from *Wolbachia* symbionts [34], and the data above indicated that non-target *Rickettsia COI* may be likewise amplified during this PCR amplification for mitochondrial *COI*.

In this paper, we use three approaches to reveal the diversity and commonness of Torix *Rickettsia* in arthropods. First, we probed a bin from the Barcode of Life Data System (BOLD [35]), containing non-target *COI* sequences, for *Rickettsia* amplicons and then used the DNA extracts from these projects to define the diversity of *Rickettsia* observed using a multilocus approach. Second, we screened DNA extracts from multiple individuals from 169 invertebrate species for *Rickettsia* presence to determine the distribution of the symbiont in both terrestrial and aquatic biomes. Finally, we used bioinformatic approaches to examine the Sequence Read Archive (SRA) depositions for one individual from 1,341 arthropod species for the presence of *Rickettsia* and used this as a means of estimating the relative balance of Torix group to other *Rickettsia* within symbioses.

Data Description

Barcode of Life Data System (BOLD)

While searching the Barcode of Life Data System (BOLD), a depository of >8 million *COI* mtDNA sequences, hundreds of hits were observed with high sequence similarity to Torix group *Rickettsia*. To investigate the diversity and host distribution of these non-target amplicons, access was permitted to analyse *COI* barcoding data deriving from a BOLD screening project totaling 184,585 arthropod specimens (including individuals where barcoding had failed) from 21 countries and collected between 2010 and 2014. *COI* sequences provided by BOLD were generally derived from DNA extracts created from somatic tissues (legs are often used in order to retain most of the specimen for further analyses if necessary), but also rarely included abdominal tissues. The first dataset made available [36] included 3,817 specimens containing sequences not matching initial morphological assignment (and likely to contain contaminant sequences). The second dataset included 55,366 specimens judged to not contain non-target amplicons [37]. A remaining 125,402 specimens were not made available, and the 55,366 subsample was used as a representative sample from which the contaminants had originated (Figure 1). The protocols for data collection, data curation and quality control of submitted BOLD samples is described by Ratnasingham & Hebert [38].

Sequence Read Archive (SRA)

Further insights into the balance of *Rickettsia* groups within arthropod symbioses were obtained through searching for *Rickettsia* presence in Illumina datasets associated with arthropod whole genome sequence (WGS) projects in the SRA (60,409 records as of the 20th May 2019). To reduce the bias from over-represented laboratory model species (e.g.

Drosophila spp., *Anopheles* spp.) a single dataset per species was examined, and where multiple data sets existed for a species, that with the largest read count was retained. The resultant dataset [39], representing 1,341 arthropod species, was then screened with phyloFlash [40] which finds, extracts and identifies SSU rRNA sequences.

Targeted screen of aquatic and terrestrial arthropods

Both the BOLD and SRA datasets have inherent biases which make them unsuitable to assess whether Torix *Rickettsia* are more common in aquatic or terrestrial biomes. For example, most SRA submissions are from lab-reared terrestrial insects. Likewise, a majority of the BOLD specimens containing *Rickettsia* have limited taxonomic and ecological information, by virtue of not returning an mtDNA *COI* sequence. Therefore, a targeted PCR screen of 1,612 individuals from 169 species was undertaken (Table 1) using primers which hybridise with all known clades of *Rickettsia* [27]. Within this, we included a range of both aquatic and terrestrial taxa, to investigate if the previous work highlighting particular aquatic taxa as hot spots for *Rickettsia* symbiosis (water beetles, biting midges, damselflies) reflects a wider higher incidence in species from this habitat.

Analyses

Torix Rickettsia is the most common bacterial contaminant sequence currently in BOLD, a major barcoding project

Out of 3,817 sequences considered as not matching initial morphological assignment, 1,126 of these were deemed by BOLD to be bacterial in origin (Figure 1, [36]). The taxonomic classification tool, Kaiju, further supported bacterial designation for all sequences except one

(Additional file 1), although this was later confirmed as *Rickettsia* through phylogenetic placement. Phylogenetic placement further confirmed the correct designation of bacterial sequences (Figure 2 and Additional file 2). The dominant genus was *Rickettsia* with 753 (66.9%) amplifications, compared to *Wolbachia* with 306 (27.2%). Of the remaining 67 non-target sequences, 14 formed a monophyletic group with other Anaplasmataceae and 48 clustered with the order Legionellales, with 5 sequences remaining undesignated. When considering the 184,585 specimens in the total project, this analysis gave an overall *Rickettsia* and *Wolbachia* frequency of 0.41% and 0.17% respectively within the dataset. Through later access to the 55,366 representative data subset from where the contaminants originated, a further 245 unique bacteria contaminants were also detected by Kaiju (possibly missed by BOLD's automated contaminant filtering system) (Additional file 1). This additional finding suggests these frequencies are conservative estimates.

BOLD *Rickettsia* contaminants were dominated by amplicons from the Torix group of *Rickettsia* (716/753; 95.1%) (Figure 3 and Additional file 2). The remaining 37 *Rickettsia* clustered with Transitional/Spotted Fever (n=15), Belli (n=9), Rhyzobius (n=1) groups, while 12 sequences formed two unique clades. Across arthropod hosts: 292 (38.8%) were derived from Hymenoptera; 189 (25.1%) from Diptera; 177 from Hemiptera (23.5%); 41 from Psocoptera (5.4%); 40 from Coleoptera (5.3%); 7 from Arachnida (0.9%); 4 from Trichoptera (0.5%); and single cases of Thysanoptera, Diplopoda and Dermaptera (0.1% each).

We observed that two sets of *COI* primers were responsible for 99% of *Rickettsia* amplifications (Additional file 3) with a majority (89%) amplifying with the primer combination

C_LepFolF/C_LepFolR [41]. Torix *Rickettsia COI* showed a stronger match to these primers at the 3' end (the site responsible for efficient primer annealing) compared to *Wolbachia* and other *Rickettsia* groups. Whilst all contained a SNP at the 3' priming end of C_LepFolR, Torix *Rickettsia* (*Rickettsia* endosymbiont of *Culicoides newsteadi*; MWZE00000000) was the only sequence to not contain a SNP at the 3' priming site of C_LepFolF (Additional file 4).

Rickettsia multilocus phylogenetic analysis

To better resolve the phylogenetic relationships between BOLD *Rickettsia* contaminants, a multilocus approach was employed on a subsample of 186 *Rickettsia*-containing samples chosen based on assorted geographic location, host order and phylogenetic placement. To this end, 2 further housekeeping genes (*16S rRNA*, *gltA*) and the antigenic *17KDa* protein gene were amplified and sequenced from the respective DNA extracts.

Overall, 135 extracts successfully amplified and gave a high-quality sequence for at least one gene. No intragenic or intergenic recombination was detected for any of the gene profiles. A phylogram, including 99 multilocus profiles containing at least 3 of the 4 *Rickettsia* genes of interest (including *COI*), allocated strains to both Limoniae and Leech subclades of the Torix group (Figure 4) and these subclades were derived from similar hosts. For example, specific families (Hemiptera: Psyllidae and Hymenoptera: Diapriidae) were present in both Leech and Limoniae groups. Furthermore, similar strains were observed between genetically dissimilar host species. For example, the *Coenagrion mercuriale* (Odonata) strain was 100% identical to the *Culicoides stigma* (Diptera) strain across all four loci. This suggests horizontal transfer of

the symbiont is likely to be occurring. A full list of multilocus profiles and *Rickettsia* group designation can be found in Additional file 5.

The multilocus study also provided evidence of co-infection with *Rickettsia*. During Sanger chromatogram analysis, double peaks were occasionally found at third codon sites from protein coding genes. This pattern was observed in 6/10 *Philotarsus californicus* individuals and in one member of each of the Psilidae, Sciaridae, Chironomidae and Diapriidae (Additional file 5). Where double peaks were observed, this was found consistently across markers within an individual specimen. This pattern corroborates a recent finding of double infections in Odoantes [28], suggesting co-infecting *Rickettsia* strains in hosts is a widespread phenomenon of the Torix group.

Barcoding success of Rickettsia host taxa

An available subset of specimens associated with the contaminants contained 55,366 out of 184,585 arthropods originally used in the overall study [37]. The three classes of Insecta (n=49,688), Arachnida (n=3,626) and Collembola (n=1,957), accounted for >99.8% of total specimens (Figure 1). Successful amplification and sequencing of *COI* was achieved in 43,246 specimens (78.1%) of the DNA extracts, but when assessed at the order level success rates varied (Additional file 6). The likely explanation for this variation is taxa-specific divergence of sequences at priming sites.

The number of each taxonomic order giving at least one *Rickettsia* amplification was then calculated and adjusted based on the total number of specimens in the project to allow for a

frequency estimate. Overall, Hymenoptera, Diptera and Hemiptera were the three taxa most likely to be associated with *Rickettsia COI* amplification (87.4%). Similarly, on assessment of a subsample from the project where the contaminants originated, a majority (77.7%) of the dataset were also accounted for by these three orders. After adjusting the frequency to take into account the number of inaccessible specimens, Trichoptera (2.45%), Dermaptera (1.89%) and Psocodea (1.67%) were the most likely taxa to give an inadvertent *Rickettsia* amplification. Whilst Hemiptera and Diptera had a similar estimated frequency of *Rickettsia* amplification (0.58% and 0.56%), Hemiptera were much more likely to fail to barcode (67.2% vs 93.3%), suggesting dipteran *Rickettsia* infection in BOLD specimens is likely to be higher than that of hemipterans, as a barcoding failure is necessary to amplify non-target bacteria *COI*. Attempts to re-barcode 186 *Rickettsia*-containing DNA extracts of interest from BOLD resulted in 90 successful arthropod host barcodes (Additional file 5).

Targeted Rickettsia PCR screen and statistical comparison of terrestrial vs aquatic insects

From the targeted screen of 169 invertebrate species, a total of 19 *Rickettsia* were discovered from both aquatic and terrestrial pools, with 17 of these identified as belonging to the Torix group. The screening of aquatic invertebrates revealed 9 out of 57 species (15.79%) were positive in PCR assays (Table 1.1). DNA sequences confirmed that all were *Rickettsia* which lay within the Torix group (Figure 5), with the positive species deriving from 8 insect species and one mollusc. For the terrestrial invertebrates, PCR assays evidenced *Rickettsia* infection in 10 out of 112 species (8.93%) with a mix of insect and spider hosts (4 and 6 species respectively, Table 1.2). *Rickettsia* from 8 host species (2 insects and 6 spiders) were identified as Torix

Rickettsia (8 of 112 species, 7.14%), while the other two host species carried *Rickettsia* from the Rhyzobius and Belli groups (Figure 5).

To reduce taxonomic hot spot biases (particularly from spiders), we compared the incidence of *Rickettsia* infection in aquatic vs terrestrial insects. Fisher's exact test analysis rejected the null hypothesis of equal representation, with aquatic taxa having a higher representation of species with Torix *Rickettsia* than terrestrial (*p*-value = 0.013, Additional file 7). Examining the phylogenetically controlled set, with three matched insect orders (Coleoptera, Diptera, Hemiptera), again rejected the null hypothesis of equal representation, with aquatic taxa having a higher representation of species with Torix *Rickettsia* than terrestrial (*p*-value = 0.025, Additional file 7). When comparing all invertebrate species from the targeted screen, no significant difference was observed in Torix *Rickettsia* incidence between terrestrial and aquatic biomes (*p*-value = 0.11, Additional file 7) suggesting this pattern of infection may be specific to insects.

[Insert Table 1 here]

SRA and GenBank Rickettsia searches

During the SRA search, phyloFlash flagged 29 *Rickettsia* sequences in the groups: Belli (n=10), Torix (n=8), Transitional (n=6), Rhyzobius (n=2), and Spotted Fever (n=1), with the remaining two failing to form a monophyletic clade with any group (Figure 5). In addition, Kraken identified eight *Rickettsia*-containing arthropod SRA datasets missed by phyloFlash. Two of these were from the Torix group, in phantom midge hosts (Diptera: Chaoboridae: *Mochlonyx*

cinctipes and *Chaoborus trivitattus*), with the remaining six placed in Belli and Spotted Fever groups [39].

phyloFLash was also used to retrieve 18S rRNA (eukaryotic) sequences which could potentially account for the *Rickettsia* observed in SRA datasets (e.g. through parasitisms or ingestion of *Rickettsia*-infected protists). Out of the 29 datasets analysed by phyloFlash, only one (SRR6313831) revealed an assembled 18S rRNA sequence aligned to a parasitoid wasp (*Hadrotrichodes waukheon*). Although reads aligned to protists were also present in 19/29 datasets flagged by phyloFlash, the read depth for protists was much lower than the number of *Rickettsia* reads [39]. This suggests that *Rickettisa*-infected protists are unlikely to account for the positives observed in the SRA datasets.

The search of GenBank revealed 11 deposits ascribed to host mtDNA that were in fact Torix *Rickettsia* sequences (Additional files 8 and 9).

The hidden host diversity of Torix Rickettsia

Overall, putative novel Torix hosts detected from all screening methods included taxa from the orders Dermaptera, Gastropoda, Trichoptera and Trombidiformes. Additionally, new Torix-associated families, genera and species were identified. These included haematophagous flies (*Simulium aureum; Anopheles plumbeus; Protocalliphora azurea*; Tabanidae), several parasitoid wasp families (e.g. Ceraphronidae; Diapriidae; Mymaridae), forest detritivores (e.g. Sciaridae; Mycetophilidae; Staphylinidae) and phloem-feeding bugs (Psyllidae; Ricaniidae). Feeding habits such as phloem-feeding, predation, detritivory or

haematophagy were not correlated with any particular Torix *Rickettsia* subclade (Figure 6). Furthermore, parasitoid and aquatic lifestyles were seen across the phylogeny. All newly discovered putative Torix *Rickettsia* host taxa are described in Table 2, alongside previously discovered hosts in order to give an up to date overview of Torix-associated taxa.

[Insert Table 2 here]

Discussion

Symbiotic interactions between hosts and microbes are important drivers of host phenotype, with symbionts both contributing to, and degrading, host performance. Heritable microbes are particularly important contributors to arthropod biology, with marked attention focused on *Wolbachia*, the most common associate [9]. Members of the Rickettsiales, like *Wolbachia*, share an evolutionary history with mitochondria [42], such that a previous screen of BOLD submissions of mtDNA submissions observed *Wolbachia* as the main bacterial contaminant associated with DNA barcoding [34]. However, our screen found that *Rickettsia* amplicons were more commonly found in BOLD deposits compared to *Wolbachia* (0.41% vs 0.17% of deposits). Furthermore, Torix group *Rickettsia* were overrepresented in barcode misamplifications (95%) when compared to other groups within the genus. A comparison of the most commonly used barcoding primers to *Wolbachia* and *Rickettsia* genomes suggest homology of the forward primer 3’ end was likely responsible for this bias towards Torix

Rickettsia amplification. To gain a clearer understanding of the relative balance of Torix group to other *Rickettsia* within symbioses and habitats, a targeted screen and bioinformatic approach was also undertaken. Through these three screens, a broad range of host diversity associated with Torix *Rickettsia* was uncovered.

As the *in silico* and empirical evidence suggests *Rickettsia COI* amplification is not uncommon [31–33], why has this phenomenon not been described more widely before? The previous large-scale non-target *COI* study using BOLD submissions [34], revealed only *Wolbachia* hits. This screen involved comparison to a *Wolbachia*-specific reference library and was thus likely to miss *Rickettsia*. Additionally, there has been a lack of Torix *Rickettsia COI* homologues to compare barcodes to until recently, where a multilocus identification system, including *COI* was devised [27]. Indeed, out of the non-target *COI* dataset received in this study, some of the *Rickettsia* contaminants were tentatively described by BOLD as *Wolbachia* due to the previous absence of publicly available *Rickettsia COI* to compare.

Although *Rickettsia* will only interfere with barcoding in a minority of cases (~0.4%), it is likely that alternate screening primers for some studies will need to be considered. In a demonstration of how unintended *Rickettsia* amplifications can affect phylogeographic studies relying on DNA barcoding, a *Rickettsia COI* was conflated with the mtDNA *COI* of a species of freshwater amphipod, *Paracalliope fluvitalis* [30]. Subsequently, supposed unique mtDNA haplotypes were allocated to a particular collection site, whereas this merely demonstrated the presence of Torix *Rickettsia* in host individuals in this lake. Contrastingly,

non-target *Rickettsia* amplification can also allow for the elucidation of a novel host range of the symbiont [31–33] and this has been exemplified with our probing of BOLD.

Previously, several host orders have been associated with Torix *Rickettsia*, including Araneae, Coleoptera, Diptera, Hemiptera and Odonata [24,28,43–45]. Newly uncovered putative host orders from this study include Dermaptera, Gastropoda, Trichoptera and Trombidiformes (Table 2). These data emphasise the broad host range of Torix *Rickettsia* across arthropods and invertebrates, with two additional cases from nucleariid amoebae [25,26]. This host range is complementary to *Rickettsia*'s sister genus '*Candidatus* Megaira' (formally the Hydra group of *Rickettsia*) which are present in multiple unicellular eukaryote families, and in a few invertebrates like *Hydra* [46].

Despite the extensive sampling and multiple screening strategies employed in this project, caution must be taken when interpreting to what extent the Torix *Rickettsia* hosts identified are representative of *Rickettsia* hosts in nature. Both BOLD and SRA components of the project rely on secondary data which come with sampling and methodological biases. For example, most SRA submissions are from lab-reared terrestrial insects and it can be argued that the high number of Belli *Rickettsia* infections discovered from arthropod genome projects (compared to the targeted screen which contains multiple aquatic insect species) could be due to this sampling bias. Likewise, the over-representation of Torix *Rickettsia* from BOLD is likely due to an amplification bias as a result of higher primer site homology to that particular group from commonly used barcoding primer sets. Subsequently, the common patterns of infection (or 'hot spots') found in this study are identified as such with these provisos in mind.

To counteract these biases and to give a more nuanced and holistic view of Torix *Rickettsia* ecology, a targeted screen was also included to ensure this study was not over-reliant on secondary data.

Further caution needs to be taken when interpreting what these newly found associations mean, as mere presence of *Rickettsia* DNA does not definitively indicate an endosymbiotic association. For example, bacterial DNA integrations into the host nuclear genome have been widely reported [47]. Although none of the protein-coding genes sequenced in this study showed signs of a frameshift, suggesting a lack of pseudogenization that is often typical of a nuclear insertion, this still does not rule out this phenomenon entirely. Furthermore, parasitism or ingestion of symbiont-infected biota (e.g. protists) could also result in bacteria detection [48–50]. Whilst protist reads were found in some datasets, these were usually at a much lower depth compared to the symbiont [39]. In one of the few instances where protist reads were greater than *Rickettsia* (Dataset SRR5298327), this was from our own previous study where a true endosymbiosis between insect and symbiont was confirmed through FISH imaging [27]. Similarly, although an 18S sequence aligned to a parasitoid wasp was observed in the SRA dataset from *Bemisia tabaci* (SRR6313831), previous work has also demonstrated a true endosymbiosis between *B. tabaci* and Torix *Rickettsia* [51]. Overall, these data suggest that detecting contamination from *Rickettsia*-infected taxa such as protists and parasitoid wasps is uncommon within our study.

Model-based estimation techniques suggest *Rickettsia* are present in between 20-42% of terrestrial arthropod species [12]. However, the targeted PCR screen in this study gave an

estimated species prevalence of 8.9% for terrestrial species. This discrepancy is likely due to targeted screens often underestimating the incidence of symbiont hosts due to various methodological biases including small within-species sample sizes (missing low-prevalence infections) [29]. Importantly, the inclusion and exclusion of specific ecological niches can also lead to a skewed view of *Rickettsia* symbioses. A previous review of *Rickettsia* bacterial and host diversity by Weinert et al. [13] suggested a possible (true) bias towards aquatic taxa in the Torix group. In accordance with this, our targeted screen demonstrated Torix *Rickettsia* infections were more prevalent in aquatic insect species compared to terrestrial (although this is likely not the case for invertebrates in general due to a Torix *Rickettsia* hot spot in spiders). The observed over-representation of Torix group *Rickettsia* (17/19 strains) in our targeted screen contrasts with Weinert's findings which show a predominance of Belli infections and is likely due to the latter study's near absence of aquatic insects and spiders within the samples screened. Our additional use of a bioinformatics approach based on the SRA appears to corroborate targeted screen data indicating that Belli and Torix are two of the most common *Rickettsia* groups among arthropods. Overall, these multiple screening methods suggest Torix *Rickettsia* are more widespread than previously thought and their biological significance underestimated.

Previous studies have used either one or two markers to identify the relatedness of strains found in distinct hosts. In this study, we use the multilocus approach developed in Pilgrim et al. [27] to understand the affiliation of Torix *Rickettsia* from diverse invertebrate hosts. Our analysis of Torix strains indicates that closely related strains are found in distantly related taxa. Closely related *Rickettsia* are also found in putative hosts from different niches and habitats –

for instance, the *Rickettsia* strains found in terrestrial blood feeders do not lie in a single clade, but rather are allied to strains found in non-blood feeding host species. Likewise, strains in phloem-feeding insects are diverse rather than commonly shared.

The distribution of Torix *Rickettsia* across a broad host range suggests host shifts are occurring between distantly related taxa. It is notable that parasitoid wasps are commonly infected with *Rickettsia* and have been associated with enabling symbiont host shifts [48]. Aside from endoparasitoids, it is also possible that plant-feeding can allow for endosymbiont horizontal transmission [52,53]. For example, *Rickettsia* horizontal transmission has been demonstrated in *Bemisia* whiteflies infected by phloem-feeding [52,54]. Finally, ectoparasites like the Torix-infected water mites of the Calyptostomatidae family, could also play a role in establishing novel *Rickettsia*-host associations, as feeding by mites has been observed to lead to host shifts for other endosymbiont taxa [55]. Indeed, if multiple horizontal transmission paths do exist, this could account for the diverse plethora of infected taxa, as well as arthropods identified in this study which harbour more than one strain of symbiont [56].

The finding that Torix *Rickettsia* are associated with a broad range of invertebrates leads to an obvious question: what is the impact and importance of these symbiotic associations? Previous work has established Torix *Rickettsia* represent heritable symbionts and it is likely that this is true generally. There have, however, been few studies on their impact on the host. In the earliest studies [22,23], *Torix* spp. leeches infected with *Rickettsia* were observed to be substantially larger than their uninfected counterparts. Since then, the only observation of note, pertaining to the Torix group, is the reduced ballooning (dispersal) behaviour observed

in infected *Erigone atra* money spiders [57]. Overall, the incongruencies in host and Torix *Rickettsia* phylogenies (suggesting a lack of co-speciation and obligate mutualism), along with the lack of observed sex bias in carrying the symbiont, indicate facultative benefits are the most likely symbiotic relationship [29]. However, *Rickettsia* induction of thelytokous parthenogenesis (observed in Belli *Rickettsia* [58,59]) should not be discounted in Torix infected parasitoid wasps identified in this study. To add to the challenge of understanding Torix *Rickettsia* symbioses, the challenges of laboratory rearing of many Torix *Rickettsia* hosts has led to difficulties in identifying model systems to work with. However, the large expansion of our Torix group host knowledge can now allow for a focus on cultivatable hosts (e.g phloem-feeding bugs).

To conclude, we have shown that large-scale DNA barcoding initiatives of arthropods can include non-target amplification of Torix *Rickettsia*. By examining these non-target sequences, alongside a targeted screen and SRA search, we have uncovered numerous previously undetected putative host associations. Our findings lay bare multiple new avenues of inquiry for Torix *Rickettsia* symbioses.

Potential Implications

A particularly important group for future study of Torix *Rickettsia* interactions are haematophagous host species. Our discovery of *Rickettsia*-associated tabanid and simulid flies, alongside *Anopheles plumbeus* mosquitoes, add to existing blood-feeders previously identified as Torix group hosts which include sand flies [60,61], fleas [62], ticks [63,64] bed bugs [65] and biting midges [27]. Some *Rickettsia* strains are known to be transmitted to

vertebrates via haematophagy [66]. However, there is no evidence to date for vertebrate pathogenic potential for the Torix group. Despite this, Torix *Rickettsia* could still play a significant role in the ecology of vectors of disease. A key avenue of research is whether these endosymbionts alter vectorial capacity, as found for other associations [67]. In contrast to the widely reported virus blocking phenotype observed in *Wolbachia*-infected vectors [68,69], Torix *Rickettsia* has recently been associated with a virus potentiating effect in *Bemisia* white flies vectoring Tomato yellow leaf curl virus [70]. Additionally, we uncovered a *Rickettsia*-infected psyllid (*Cacopsylla melanoneura*) which is a vector of *Phytoplasma mali* (apple proliferation) [71]. Thus, the question of Torix *Rickettsia* vector-competence effects is clearly of widespread relevance and deserves further attention.

Methods

a) Interrogation of the Barcode of Life Data System (BOLD)

Assessment of non-target microbe amplicons

BOLD data curation involves identifying non-target *COI* sequences from common contaminants (e.g. human and bacteria) or erroneous morphological identifications [38]. The designation of bacterial contaminants by BOLD, from a dataset containing 3,817 non-target sequences [36], was confirmed by the taxonomic classification program, Kaiju, using default parameters [72]. Sequences were then placed phylogenetically to refine taxonomy further. To this end, barcodes confirmed as microbial sequences were aligned using the "L-INS-I" algorithm in MAFFT v7.4 (RRID:SCR_011811) [73]. Gblocks (RRID:SCR_015945) [74] was then used to exclude areas of the alignment with excessive gaps or poor alignment using 'options for a less stringent selection'; the inclusion of some missing data in alignments was allowed as

missing characters does not often affect phylogenetic resolution for taxa with complete data [75]. ModelFinder [76] then determined the TIM3+F+I+G4 model to be used after selection based on default “auto” parameters using the Bayesian information criteria. A maximum likelihood (ML) phylogeny was then estimated with IQTree [77] using an alignment of 561 nucleotides and 1000 ultrafast bootstraps [78]. The Rickettsiales genera *Anaplasma*, *Rickettsia*, *Orientia* and *Wolbachia* (Supergroups A, B, E and F), as well as the Legionellales genera *Legionella* and *Rickettsiella*, were included in the analysis as references (as suggested by Kaiju). Finally, both phylogram and cladogram trees (the latter for ease of presentation) were drawn and annotated based on host taxa (order) using the EvolView [79] online tree annotation and visualisation tools. Subsequent phylogenetic workflows detailed below follow this method with the exception being the chosen models by Modelfinder.

A determining factor for non-target amplification of bacteria is primer site matching to microbial associates. Subsequently, pairwise homology of the primer set predominantly used for BOLD barcode screening was compared to *Rickettsia* and *Wolbachia COI* genes.

Further phylogenetic analysis

COI sequence alone provides an impression of the frequency with which *Rickettsia* associates are found in barcoding studies. However, they have limited value in describing the diversity of the *Rickettsia* found. To provide further insight into the diversity of *Rickettsia* using a multilocus approach, we obtained 186 DNA extracts from the archive at the Centre for Biodiversity Genomics (University of Guelph, Canada) that had provided *Rickettsia* amplicons in the previous screen. DNA extracts were chosen based on assorted geographic location, host

order and phylogenetic placement. Multilocus PCR screening and phylogenetic analysis of *Rickettsia* was then completed, using the methodology in Pilgrim et al. which utilised primers conserved across all known clades of the *Rickettsia* genus [27]. However, slight variations include the exclusion of the *atpA* gene due to observed recombination at this locus. Furthermore, the amplification conditions for the *17KDa* locus was changed because a Torix *Rickettsia* reference DNA extract (Host: *Simulium aureum*) failed to amplify with the primer set Ri_17KD_F/ Ri_17KD_R from Pilgrim et al. [27]. Subsequently, a *17KDa* alignment from genomes spanning the Spotted fever, Typhus, Transitional, Belli, Limoniae groups, and the genus '*Candidatus* Megaira' was generated to design a new set of primers using the online tool PriFi [80].

Once multilocus profiles of the *Rickettsia* had been established, we tested for recombination within and between loci using RDP v4 (Recombination Detection Program, RRID:SCR_018537) [81] using the MaxChi, RDP, Chimaera, Bootscan and GENECONV algorithms with the following criteria to assess a true recombination positive: a p-value of <0.001; sequences were considered linear with 1000 permutations being performed. Samples amplifying at least 3 out of 4 genes (*16S rRNA*, *17KDa*, *COI* and *gltA*) were then concatenated and their relatedness estimated using maximum likelihood as described above. The selected models used in the concatenated partition scheme [82] were as follows: *16S rRNA*: TIM3+F+R2; *17KDa*: GTR+F+I+G4; *COI:*TVM+F+I+G4; *gltA:* TVM+F+I+G4. Accession numbers for all sequences used in phylogenetic analyses can be found in Additional file 10.

Re-barcoding Rickettsia-containing BOLD DNA extracts

Aside from phylogenetic placement of these *Rickettsia*-containing samples, attempts were made to extract an mtDNA barcode from these taxa in order to identify the hosts of infected specimens. This is because morphological taxonomic classification of specimens in BOLD is usually only down to the order level before barcoding takes place. Previous non-target amplification of *Rickettsia* through DNA barcoding of arthropod DNA extracts had occurred in the bed bug *Cimex lectularius*, with a recovery of the true barcode after using the primer set C1-J-1718/HCO1490, which amplifies a shortened 455 bp sequence within the *COI* locus. Subsequently, all samples were screened using these primers or a further set of secondary *COI* primers (LCOt_1490/ MLepR1 and LepF1/C_ANTMR1D) if the first failed to give an adequate host barcode. All *COI* and *Rickettsia* multilocus screening primer details, including references, are available in Additional file 11.

Cycling conditions for *COI* PCRs were as follows: initial denaturation at 95°C for 5 min, followed by 35 cycles of denaturation (94°C, 30 sec), annealing (50°C, 60 sec), extension (72°C, 90 sec), and a final extension at 72°C for 7 min. *Rickettsia* and host amplicons identified by gel electrophoresis were subsequently purified enzymatically (ExoSAP) and Sanger sequenced through both strands using a BigDye® Terminator v3.1 kit (Thermo Scientific, Waltham, USA), and capillary sequenced on a 3500 xL Genetic Analyser (Applied Biosystems, Austin, USA). Forward and reverse reads were assessed in UGENE (RRID:SCR_005579)[83] to create a consensus sequence by eye with a cut-off phred (Q) score [84] of 20. Primer regions were trimmed from barcodes before being matched to the GenBank database by BLAST based on default parameters and an e-value threshold of <1e-85. Host taxonomy was determined by a

barcode-based assignment of the closest BLAST hit, under the following criteria modified from Ramage et al. [50]:

1) Species level designation for at least 98% sequence identity.

2) Genus level designation for at least 95% sequence identity.

3) Family level designation for at least 85% sequence identity.

Additionally, all sequences were required to be at least >200 bp in length.

Assessment of barcoding success

One of the factors determining a successful *COI* bacterial amplification is the initial failure of an extract to amplify mtDNA. Subsequently, to determine the likelihood of this event within taxa, we used the 55,366 specimen representative data subset [37] to evaluate failure rates. To this end, all orders of host which gave at least one non-target *Rickettsia COI* hit were assessed. The barcoding success rate was determined as the proportion of specimens which matched initial morphotaxa assignment and were not removed after BOLD quality control [38]. As the total *Rickettsia* count was from a larger dataset than the one made available, an adjusted infection frequency for each taxon was calculated based on the representative data subset.

b) Targeted and bioinformatic *Rickettsia* screens

Targeted screen of aquatic and terrestrial arthropods

Overall, 1,612 individuals from 169 species, including both terrestrial (DNA extracts derived from European material, mostly from Duron et al. [11]) and aquatic invertebrates (largely acquired from the UK between 2016-2018), were screened. mtDNA *COI* amplification was

conducted as a control for DNA quality. Some arthropods which could not be identified down to the species level morphologically or from barcoding were referred to as 'sp.'. To investigate symbiont infection status, rickettsial-specific primers based on *gltA* and *16S rRNA* genes were used for conventional PCR screening [27], with Sanger sequences obtained from at least one specimen per *Rickettsia* positive species to identify any misamplification false positives. Newly identified hosts of interest from BOLD and targeted screens were then placed phylogenetically (see sections above) with the models TIM3+F+R2 (16S) and K3Pu+F+G4 (gltA) before being mapped by lifestyle and diet.

It is known that there are taxonomic hot spots for endosymbiont infection, with for instance spiders being a hot spot for a range of microbial symbionts [43]. Therefore, analyses were performed that were matched at a taxonomic level (i.e. each taxon was represented in both the aquatic and terrestrial pools). To this end, the incidence of Torix *Rickettsia* was first compared in all insects. However, within insects, there is taxon heterogeneity between aquatic and terrestrial biomes (e.g. Ephemeroptera, Plecoptera in aquatic only, Lepidoptera in terrestrial only). The analysis was therefore narrowed to match insect orders present in both the aquatic and terrestrial community. Three insect orders, Hemiptera, Diptera and Coleoptera, fulfilled this criterion with good representation from each biome. For each case, the ratios of the infected:non-infected species between aquatic and terrestrial communities were compared in a Fisher's exact test with a p-value significance level of ≤ 0.05.

Search of the Sequence Read Archive (SRA) and GenBank

The SRA dataset [39] containing one individual from 1,341 arthropod species was screened with phyloFlash [40] using default parameters, which finds, extracts and identifies SSU rRNA sequences. Reconstructed full *16S rRNA* sequences affiliated to *Rickettsia* were extracted and compared to sequences derived from the targeted screen phylogenetically (see sections above) to assess group representation within the genus. The microbial composition of all SRA datasets that did not result in a reconstructed *Rickettsia 16S rRNA* with phyloFlash were re-evaluated using Kraken2 [85], a k-mer based taxonomic classifier for short DNA sequences. A cut-off of at least 40k reads assigned to *Rickettsia* taxa was applied for reporting potential infections (theoretical genome coverage of ~ 1 – 4X assuming an average genome size of ~1.5Mb). As *Rickettsia*-infected protists and parasitoids have previously been reported [25,26,59], phyloFlash was also used to identify reads aligned to these taxa to account for potential positives attributed to ingested protists or parasitisms.

We also examined GenBank for *Rickettsia* sequences deposited as invertebrate *COI* barcodes. To this end, a BLAST search of Torix *Rickettsia COI* sequences from previous studies [27,32] was conducted on the 29th June 2020. Sequences were putatively considered belonging to the Torix group if their similarity was >90% and subsequently confirmed phylogenetically as described above with the HKY+F+G4 model.

Table 1.1. Targeted *Rickettsia* screen of aquatic/semiaquatic invertebrates.

Aquatic/Semiaquatic invertebrate group	Species	Location	Year	No. tested	No positive
	Baetis muticus	Stirling, Scotland, UK	2017	3	0
	Baetis rhodani	Stirling, Scotland, UK	2017	3	0
	Cloeon dipterum	Cheshire, UK	2016	3	0
	Ecdyonurus sp.1	Stirling, Scotland, UK	2017	5	0

Ephemeroptera	*Ecdyonurus* sp.2	Cheshire, UK	2016	3	0
	Ecdyonurus venosus	Cheshire, UK	2016	6	0
	Leptophlebia vespertina	Hampshire, UK	2016	1	0
	Paraleptophlebia submarginata	Stirling, Scotland, UK	2017	3	0
	Rhithrogena semicolorata	Stirling, Scotland, UK	2017	3	0
	Hydropsyche sp.	Stirling, Scotland, UK	2017	3	0
Trichoptera	*Polycentropus flavomaculatus*	Cheshire, UK	2017	3	0
	Rhyacophila dorsalis	**Stirling, Scotland, UK**	**2017**	**3**	**2**
	Amphinemura sulcicollis	Stirling, Scotland, UK	2017	3	0
Plecoptera	*Dinocras cephalotes*	Stirling, Scotland, UK	2017	3	0
	Isoperla grammatica	Stirling, Scotland, UK	2017	3	0
	Perla bipunctata	Stirling, Scotland, UK	2017	3	0
	Corixa punctata	Cheshire, UK	2016	1	0
	Gerris sp.	Montferrier sur Lez, France	2006	12	0
	Gerris thoracicus	Cheshire, UK	2016	1	0
	Hydrometra stagnorum	Montferrier sur Lez, France	2006	20	0
Hemiptera	*Nepa cinerea*	Montferrier sur Lez, France	2006	3	0
	Notonecta glauca	Cheshire, UK	2016	2	0
	Plea minutissima	Notre Dame de Londres, France	2006	8	0
	Sigara lateralis	Notre Dame de Londres, France	2006	6	0
	Sigara striata	**Cheshire, UK**	**2006**	**2**	**1**
	Aedes sp.	Cheshire, UK	2017	8	0
	Aedes albopictus	Roma, Italy	2005	20	0
	Anopheles plumbeus	**Chester Zoo, UK**	**2018**	**2**	**2**
	**Chironomidae sp.**	**Cheshire, UK**	**2016**	**4**	**1**
	Chironomus acidophilus	Cheshire, UK	2017	1	0
	Chironomus plumosus	Notre Dame de Londres, France	2006	20	0
	Chironomus sp.	Cheshire, UK	2016	4	0
	Culex pipiens (ssp. quinquefasciatus)	Puerto Viejo de Talamanca, Costa Rica	2006	20	0
Diptera	*Culex pipiens*	St Nazaire de Pézan, France	2006	20	0
	Eristalinus sp.	Cheshire, UK	2016	3	0
	Eristalis tenax	Montpellier (grotte du zoo), France	2002	7	0
	***Glyptotendipes* sp.**	**Cheshire, UK**	**2016**	**1**	**1**
	***Hilara* interstincta**	**Cheshire, UK**	**2017**	**3**	**1**
	Simulium aureum	**Hampshire, UK**	**2017**	**1**	**1**
	Simulium ornatum	N/A	2003	12	0
	Tipula sp.	UK	2006	10	0
	Tipula oleracea	UK	2006	13	0
	***Zavrelimyia* sp.**	**Northumberland, UK**	**2017**	**1**	**1**
	Agabus bipustulatus	Cheshire, UK	2017	3	0
Coleoptera	*Guignotus pusillus*	Notre Dame de Londres, France	2006	12	0
	Unknown sp.1	Cheshire, UK	2017	2	0
	Unknown sp.2	Cheshire, UK	2017	3	0
Acarina	Unknown sp.	Cheshire, UK	2017	3	0
Isopoda	*Asellus aquaticus*	Cheshire, UK	2016	3	0
Amphipoda	*Gammarus pulex*	Stirling, Scotland, UK	2017	3	0
	Crangonyx pseudogracilis	Cheshire, UK	2016	6	0
	Radix balthica	Cheshire, UK	2016	3	0
Gastropoda	*Planorbis* sp.	Cheshire, UK	2016	3	0
	Galba truncatula	**Cheshire, UK**	**2017**	**20**	**3**
Hirudinea	*Erpobdella octoculata*	Cheshire, UK	2016	2	0
	Hemiclepsis marginata	Cheshire, UK	2017	1	0
Tricladida	Unknown sp.	Cheshire, UK	2016	1	0

A species was deemed positive through PCR and designated to *Rickettsia* group after Sanger sequencing and phylogenetic placement. All strains belong to the Torix group.

Table 1.2. Targeted *Rickettsia* screen of terrestrial invertebrates.

Terrestrial Invertebrate group	Species	Location	Year	Number tested	Number positive
	Agelenopsis aperta	Tennessee, USA	N/A	12	0
	Allopecosa pulverulenta	Berne, Germany	N/A	16	0
	Amaurobius fenestralis	**Montpellier, France**	**2006**	**16**	**1**
	Araneus diadematus	Beerse, Belgium	N/A	19	0
	Araneus diadematus	Greater London, UK	N/A	8	0
	Argiope bruennichi	Hamburg, Germany	N/A	7	0
	Argiope lobata	Spain	N/A	7	0
	Argiope lobata	Israel	N/A	4	0
	Cyclosa conica	Brandenburg, Germany	N/A	11	0
	Dysdera crocata	Montpellier, France	2006	2	0
	Enoplognatha ovata	Greater London, UK	N/A	20	0
	Erigone atra	Cheshire, UK	2017	1	0
	Evarcha falcata	Beerse, Belgium	N/A	5	0
	Holochnemus pluchei	Montpellier, France	2006	7	0
	Hylyphantes graminicola	**Cheshire, UK**	**2017**	**1**	**1**
	Larinioides cornutus	Greater London, UK	N/A	6	0
	Larinoides sclopetarius	Hamburg, Germany	N/A	17	0
	Linyphia triangularis	**Berlin, Germany**	**N/A**	**9**	**9**
	Linyphia triangularis	Greater London, UK	N/A	6	0
Araneae	*Lycosa* sp.	Cheshire, UK	2017	2	0
	Metellina mengei	Greater London, UK	N/A	13	0
	Metellina segmentata	Brandenburg, Germany	N/A	9	0
	Neriene clathrata	Beerse, Belgium	N/A	13	0
	Neriene peltata	Cheshire, UK	2017	1	0
	Pachygnatha degeeri	Berne, Germany	N/A	11	0
	Pachygnatha listeri	Beerse, Belgium	N/A	17	0
	Pardosa lugubris	**Darmstadt, Germany**	**N/A**	**20**	**1**
	Pardosa pullata	Brandenburg, Germany	N/A	20	0
	Pardosa purbeckensis	Belgium	N/A	19	0
	Pholcus phalangioides	**Berlin, Germany**	**N/A**	**20**	**17**
	Pisaura mirabilis	**Greater London, UK**	**N/A**	**12**	**1**
	Tetragnatha montana	Greater London, UK	N/A	20	0
	Tetragnatha sp.	Hampshire, UK	2017	3	0
	Unknown sp.	Cheshire, UK	2017	2	0
	Xysticus cristatus	Cambridgeshire, UK	N/A	16	0
Opiliones	*Leiobunum rotundum*	Feurs, France	2006	6	0
Ixodida	*Ixodes uriae*	Hornøya, Norway	2005	19	0
	Rhipicephalus microplus	New Caledonia, France	2003	1	0
Scorpiones	*Euscorpius flavicauda*	St Nazaire de Pézan, France	2006	1	0
Diplopoda	*Ommatoiulus* sp.	Cheshire, UK	2016	1	0
Neuroptera	Unknown sp.	Cheshire, UK	2017	1	0
Mecoptera	*Panorpa* sp.	Cheshire, UK	2017	2	0
	Calliptamus italicus	Notre Dame de Londres, France	2016	18	0
Orthoptera	*Chorthippus brunneus*	Uk	2006	20	0
	Gryllomorpha dalmatina	Montpellier, France	2006	2	0

Blattaria	*Loboptera decipiens*	Montpellier, France	2006	17	0
Mantodae	*Iris oratoria*	St Nazaire de Pézan, France	2006	6	0
	Mantis religiosa	Feurs, France	2006	3	0
Dermaptera	*Forficula Auricularia*	Feurs, France	2006	9	0
Hemiptera	*Aphis fabae*	Montpellier, France	2006	12	0
	Aphis nerii	Montpellier, France	2006	8	0
	Baizongia pistaciae	Viols le Fort, France	2006	12	0
	Cicadella viridis	L'Olme, France	2006	16	0
	Cimex lectularius	**Yorkshire, UK**	**2008**	**12**	**12**
	Elasmucha grisea	Greater London, UK	2006	16	0
	Graphosoma italicum	Montpellier, France	2006	12	0
	Lygaeus equestris	Montpellier, France	2006	12	0
	Notostira elongata	L'Olme, France	2006	11	0
	Pyrrhocoris apterus	Montpellier, France	2006	11	0
	Rhyparochromus vulgaris	Castelnaudary, France	2006	20	0
Coleoptera	*Anaspis frontalis*	Mont Barri, France	2004	12	0
	Anthaxia nitidula	Mont Barri, France	2004	20	0
	Anthaxia sp.	Mont Barri, France	2004	16	0
	Calvia 14-guttata	Greater London, UK	2006	6	0
	Capnodis tenebrionis	Montpellier, France	2006	1	0
	Cetonia aurata	Feurs, France	2006	3	0
	Cetonia aurata	Mont Barri, France	2004	12	0
	Chrysolina varians	Mont Barri, France	2004	18	0
	Clytus arietis	Mont Barri, France	2004	20	0
	Dermestes sp.	Mont Barri, France	2004	20	0
	Dermestes tessellatocollis	Cheshire, UK	2016	2	0
	Gastrophysa sp.	Greater London, UK	2006	20	0
	Geotrupes stercorarius	Mont Barri, France	2004	3	0
	Larinus scolymi	Aldira de Irmeros, Spain	2005	12	0
	Leptinotarsa decemlineata	Feurs, France	2006	10	0
	Mordellistena sp.	Mont Barri, France	2004	10	0
	Oedemera sp.	Mont Barri, France	2004	20	0
	Oncocerna sp.	Mont Barri, France	2004	20	0
	Phyllobius argentatus	**Mont Barri, France**	**2004**	**15**	**4†**
	Pseudovadonia livida	Mont Barri, France	2004	19	0
	Stenopterus sp.	Mont Barri, France	2004	20	0
Diptera	*Braula coeca*	Ouessant, France	2002	4	0
	Chorisops tunisiae	Montpellier, France	2003	8	0
	Delia antiqua	N/A	N/A	11	0
	Delia platura	N/A	N/A	11	0
	Delia radiacum	N/A	N/A	10	0
	Gasterophilus intestinalis	France	N/A	10	0
	Hippobosca equina	Restinclières, France	2006	15	0
	Lonchoptera lutea	Cheshire, UK	2017	3	0
	Medetera petrophila	St Bauzille de Putois, France	2003	12	0
	Musca domestica	L'Olme, France	2006	20	0
	Musca vitripennis	Notre Dame de Londres, France	2003	8	0
	Neomyia cornicina	Notre Dame de Londres, France	2003	8	0
	Protocalliphora sp.	Corse, France	2003	2	0
	Protocalliphora azurea	**Montpellier, France**	**2005**	**12**	**12**
	Psila rosae	N/A	N/A	11	0
	Stomoxys calcitrans	Le Malzieu, France	2001	11	0
Lepidoptera	*Chilo phragmitellus*	Feurs, France	2006	10	0
	Euplagia quadripunctaria	Feurs, France	2006	2	0
	Pieris brassicae	Feurs, France	2006	7	0
	Plodia interpunctella	Montpellier, France	2006	12	0

	Thymelicus lineola	Greater London, UK	2006	15	0
	Thymelicus sylvestris	Greater London, UK	2006	2	0
	Triodia sylvina	Montpellier, France	2006	4	0
Hymenoptera	*Amblyteles armatorius*	St Nazaire de Pézan, France	2006	1	0
	Amegilla albigena	St Nazaire de Pézan, France	2006	13	0
	Amegilla ochroleuca	St Nazaire de Pézan, France	2006	3	0
	Anthidium florentinum	St Nazaire de Pézan, France	2006	6	0
	Apis mellifera	UK	2006	9	0
	Bombus terrestris	North West, Switzerland	2006	20	0
	Diplolepis rosae	L'Olme, France	2006	2	0
	Formica lugubris	UK	2006	10	0
	***Pachycrepoideus* sp.**	**UK**	**N/A**	**94**	**6‡**
	Polistes dominulus	St Nazaire de Pézan, France	2006	4	0
	Polistes nimpha	St Nazaire de Pézan, France	2006	19	0
	Sceliphron caementarium	St Nazaire de Pézan, France	2006	3	0

A species was deemed positive through PCR and designated to *Rickettsia* group after Sanger sequencing and phylogenetic placement. All strains belong to the Torix group except †=Rhyzobius and ‡=Belli.

Table 2. Torix *Rickettsia* hosts known to date alongside screening method.

Order	Host	Screening method	Reference
Amphipoda	***Paracalliope fluviatilis* (Paracalliopiidae)**	**GenBank search**	**This study**
	Paraleptamphopus sp. (Paraleptamphopidae)	Barcoding	[33]
	Senticaudata sp.	Barcoding	[33]
Araneae	***Amaurobius fenestralis* (Amaurobiidae)**	**Targeted PCR**	**This study**
	Amaurobioides africana (Anyphaenidae)	Barcoding	[32]
	Araneus diadematus (Araneidae)	Targeted PCR	[43]
	Dysdera microdonta (Dysderidae)	Barcoding	[31]
	Linyphiidae spp.	Targeted PCR	[43]

	***Linyphia triangularis* (Linyphiidae)**	**Targeted PCR**	**This study**
	***Pardosa lugubris* (Lycosidae)**	**Targeted PCR**	**This study**
	***Pholcus phalangioides* (Pholcidae)**	**Targeted PCR**	**This study**
	***Pisaura mirabilis* (Pisauridae)**	**Targeted PCR**	**This study**
	Metellina mengei (Tetragnathidae)	Targeted PCR	[43]
Coleoptera	*Deronectes* spp. (Dytiscidae)	Targeted PCR, FISH and TEM	[24]
	***Dytiscidae* sp.**	**Barcoding**	**This study**
	Stegobium paniceum (Ptinidae)	Non-targeted (16S) PCR	[86]
	***Prionocyphon limbatus* (Scirtidae)**	**Barcoding**	**This study**
	***Labidopullus appendiculatus* (Staphylinidae)**	**SRA search**	**This study**
	***Platyusa sonomae* (Staphylinidae)**	**SRA search**	**This study**
	***Pseudomimeciton antennatum* (Staphylinidae)**	**SRA search**	**This study**
	***Staphylinidae* sp.**	**Barcoding**	**This study**
	***Pimelia* sp. (Tenebrionidae)**	**GenBank search**	**This study**
Dermaptera	***Forficula* sp. (Forficulidae)**	**GenBank search**	**This study**
	**unknown sp.**	**Barcoding**	**This study**
Diplopoda	*Polydesmus complanatus* (Polydesmidae)	Targeted PCR	[87]
	**unknown sp.**	**Barcoding**	**This study**
	***Protocalliphora azurea* (Calliphoridae)**	**Targeted PCR**	**This study**
	***Cecidomyiidae* sp.**	**Barcoding**	**This study**
	***Chaoborus trivittatus* (Chaoboridae)**	**SRA search**	**This study**
	***Mochlonyx cinctipes* (Chaoboridae)**	**SRA search**	**This study**

Diptera	***Glyptotendipes* sp. (Chironomidae)**	**Targeted PCR**	**This study**
	***Zavrelimyia* sp. (Chironomidae)**	**Targeted PCR**	**This study**
	Culicoides spp. (Ceratopogonidae)	Targeted PCR and FISH	[27]
	***Anopheles plumbeus* (Culicidae)**	**Targeted PCR**	**This study**
	Dolichopodidae spp.	Targeted PCR	[44]
	Empididae spp.	Targeted PCR	[44]
	Limonia chorea (Limoniidae)	N/A	Unpublished (AF322443)
	***Boletina villosa* (Mycetophilidae)**	**Barcoding**	**This study**
	**Gnoriste bilineata (Mycetophilidae)**	**SRA search**	**This study**
	***Mycetophila lunata* (Mycetophilidae)**	**GenBank search**	**This study**
	***Psilidae* sp.**	**Barcoding**	**This study**
	Lutzomyia apache (Psychodidae)	Targeted PCR	[61]
	Phlebotomus chinensis (Psychodidae)	Non-targeted (16S) PCR	[60]
	***Sciaridae* sp.**	**Barcoding**	**This study**
	***Pherbellia tenuipes* (Sciomyzidae)**	**Barcoding**	**This study**
	***Simulium aureum* (Simuliidae)**	**Targeted PCR**	**This study**
	***Tabanidae* sp.**	**Barcoding**	**This study**
Gastropoda	***Galba truncatula* (Lymnaeidae)**	**Targeted PCR**	**This study**
Haplotaxida	*Mesenchytraeus solifugus* (Enchytraediae)	Non-targeted (16S) PCR	[88]
	Bemisia tabaci (Aleyrodidae)	Targeted PCR and FISH	[51]
	Nephotettix cincticeps (Cicadellidae)	Targeted PCR, FISH and TEM	[89]
	Platypleura kaempferi (Cicadidae)	Non-targeted (16S) PCR	[90]
	***Cimex lectularius* (Cimicidae)**	**Targeted PCR**	**This study/**[65]
	***Sigara striata* (Corixidae)**	**Targeted PCR**	**This study**

Hemiptera	***Metcalfa pruinosa* (Flatidae)**	**GenBank search**	**This study**
	***Flavina* sp. (Issidae)**	**GenBank search**	**This study**
	Centrotus cornutus (Membracidae)	Non-targeted (16S) PCR and TEM	[91]
	Gargara genistae (Membracidae)	Non-targeted (16S) PCR and TEM	[91]
	Macrolophus pygmaeus (Miridae)	Non-targeted (16S) PCR and FISH	[45]
	***Cacopsylla melanoneura* (Psyllidae)**	**Barcoding**	**This study**
	***Chamaepsylla hartigii* (Psyllidae)**	**Barcoding**	**This study**
	***Ricaniidae* sp.**	**Barcoding**	**This study**
Hirudinea	*Hemiclepsis* spp. (Glossiphoniidae)	Targeted PCR and TEM	[23]
	Torix spp. (Glossiphoniidae)	Targeted PCR and TEM	[23]
Hymenoptera	*Asobara tabida* (Braconidae)	Non-targeted (16S) PCR	[92]
	***Ceraphronidae* sp.**	**Barcoding**	**This study**
	***Diapriidae* sp.**	**Barcoding**	**This study**
	***Eucharitidae* sp.**	**GenBank search**	**This study**
	Quadrastichus mendeli (Eulophidae)	Non-targeted (16S) PCR and FISH	[93]
	***Formicidae* sp.**	**GenBank search**	**This study**
	Atta colombica (Formicidae)	Non-targeted (16S) PCR	Unpublished (LN570502)
	***Megaspilidae* sp.**	**Barcoding**	**This study**
	***Mymaridae* sp.**	**Barcoding**	**This study**
	***Platygastridae* sp.**	**Barcoding**	**This study**
Ixodida	*Argas japonica* (Argasidae)	Non-targeted (16S) PCR	[64]
	Ixodes ricinus (Ixodidae)	Targeted PCR	[63]
Megaloptera	*Sialis lutaria* (Sialidae)	Targeted PCR	[94]
Neuroptera	*Chrysotropia ciliata* (Chrysopidae)	Targeted PCR	[94]

Nucleariida	*Nuclearia pattersoni* (Nucleariidae)	Non-targeted (16S) PCR	[25]
	Pompholyxophrys punicea (Pompholyxophryidae)	Single cell sequencing	[26]
Odonata	***Calopteryx maculata* (Calopterygidae)**	**GenBank search**	**This study**
	Coenagrionidae spp.	Targeted PCR and FISH	[28]
	Sympetrum fonscolombii (Libellulidae)	Targeted PCR	[28]
	Polythoridae spp.	Targeted PCR	[28]
	Neoneura sylvatica (Protoneuridae)	Targeted PCR	[28]
Psocoptera	***Myopsocidae* sp.**	**Barcoding**	**This study**
	***Philotarsus californicus* (Philotarsidae)**	**Barcoding**	**This study**
	Cerobasis guestfalica (Trogiidae)	Targeted PCR and FISH	[95]
Siphonaptera	*Nosopsyllus fasciatus* (Ceratophyllidae)	Targeted PCR	[62]
Trichoptera	***Lepidostoma hoodi* (Lepidostomatidae)**	**Barcoding**	**This study**
	***Rhyacophila dorsalis* (Rhyacophilidae)**	**Targeted PCR**	**This study**
	***Sericostoma* sp. (Sericostomatidae)**	**SRA search**	**This study**
Trombidiformes	***Calyptostomatidae* sp.**	**Barcoding**	**This study**

Bold entries indicate hosts identified in this study. FISH=fluoresence *in-situ* hybridisation; TEM=transmission electron microscopy; SRA=sequence read archive. Accession numbers for *Rickettsia* sequences from newly detected hosts can be found in Additional files 8 and 10.

Availability of Supporting Data and Materials

The data sets supporting the findings of this study are openly available in:

The Barcode of Life Data System (BOLD) repository [37] and the Figshare repository [36][39].

Alignments and trees are also available from the *GigaScience* GigaDB repository [96].

For DNA sequences, accessions are: Bioproject number PRJEB38316; LR798809-LR800243; LR812141-LR812260; LR812269-LR812283; LR812678; LR813674-LR813676; LR813730.

Declarations

List of Abbreviations

BOLD = Barcode of Life Data System

COI = cytochrome c oxidase I

FISH = fluorescence *in-situ* hybridisation

SRA = Sequence Read Archive

Ethics Approval

Not applicable.

Consent for Publication

Not applicable.

Competing Interests

The authors declare that they have no competing interests.

Funding

This work was supported by: a BBSRC Doctoral Training Partnership studentship (BB/M011186/1) awarded to JP; a Development and Promotion of Science and Technology Talents Project (DPST) of the Institute for the Promotion of Teaching Science and Technology,

Thailand to PT and a Harry Smith Vacation studentship (Microbiology society) and a NERC ACCE DTP studentship (NE/L002450/1) to HRD.

Acknowledgments

We would like to thank Dr. Michael Gerth for kindly providing comments on the manuscript.

Author contributions

JP, GDDH, MB and MAS: conception and design of the study. MAS, EVZ, SR and JRD: assembling BOLD datasets and providing DNA extracts for laboratory experiments. Field and laboratory work: JP, CRM and PT. SRA work: HRD and SS. Analyses and interpretation of the data, drafting of the manuscript: JP, PT, HRD, GDDH, MB and SS. All authors assisted in critical revision of the manuscript.

References

1. McFall-Ngai M, Hadfield MG, Bosch TCG, Carey H V., Domazet-Lošo T, Douglas AE, et al. Animals in a bacterial world, a new imperative for the life sciences. Proc Natl Acad Sci. 2013;110:3229–36.

2. Hurst GDD. Extended genomes: symbiosis and evolution. Interface Focus. 2017;7:20170001.

3. Łukasik P, Guo H, van Asch M, Ferrari J, Godfray HCJ. Protection against a fungal pathogen conferred by the aphid facultative endosymbionts Rickettsia and Spiroplasma is expressed in multiple host genotypes and species and is not influenced by co-infection with another symbiont. J Evol Biol. 2013;26:2654–61.

4. Teixeira L, Ferreira A, Ashburner M. The bacterial symbiont Wolbachia induces resistance to RNA viral infections in Drosophila melanogaster. PLoS Biol. 2008;6:2753–63.

5. Rio RVM, Attardo GM, Weiss BL. Grandeur Alliances: Symbiont metabolic integration and obligate arthropod hematophagy. Trends Parasitol. 2016;32:739–49.

6. Douglas AE. The microbial dimension in insect nutritional ecology. Funct Ecol. 2009;23:38–47.

7. Hurst GDD, Frost CL. Reproductive parasitism: Maternally inherited symbionts in a biparental world. Cold Spring Harb Perspect Biol. 2015;7:a017699.

8. Munson MA, Baumann P, Kinsey MG. *Buchnera* gen. nov. and *Buchnera aphidicola* sp. nov., a taxon consisting of the mycetocyte-associated, primary endosymbionts of aphids. Int J Syst Bacteriol. 1991;41:566–8.

9. Zug R, Hammerstein P. Still a host of hosts for Wolbachia: Analysis of recent data suggests that 40% of terrestrial arthropod species are infected. PLoS One. 2012;7:e38544.

10. Siozios S, Gerth M, Griffin JS, Hurst GDD. Symbiosis: Wolbachia host shifts in the fast lane. Curr Biol. 2018;28:R269–71.

11. Duron O, Bouchon D, Boutin S, Bellamy L, Zhou L, Engelstädter J, et al. The diversity of reproductive parasites among arthropods: Wolbachia do not walk alone. BMC Biol. 2008;6:27.

12. Weinert LA, Araujo-Jnr E V, Ahmed MZ, Welch JJ. The incidence of bacterial endosymbionts in terrestrial arthropods. Proc R Soc B. 2015;282:20150249.

13. Weinert LA, Werren JH, Aebi A, Stone GN, Jiggins FM. Evolution and diversity of *Rickettsia* bacteria. BMC Biol. 2009;7:6.

14. Perlman SJ, Hunter MS, Zchori-Fein E. The emerging diversity of Rickettsia. Proc R Soc B. 2006;273:2097–106.

15. Ricketts HT. A micro-organism which apparently has a specific relationship to Rocky Mountain spotted fever. J Am Med Assoc. 1909;52:379–80.

16. da Rocha-Lima H. Zur Aetiologie des Fleckfiebers. Dtsch Medizinische Wochenschrift. 1916;53:567–9.

17. Werren JH, Hurst GD, Zhang W, Breeuwer JA, Stouthamer R, Majerus ME. Rickettsial relative associated with male killing in the ladybird beetle (*Adalia bipunctata*). J Bacteriol. 1994;176:388–94.

18. Chen D-Q, Campbell BC, Purcell AH. A new *Rickettsia* from a herbivorous insect, the pea aphid *Acyrthosiphon pisum* (Harris). Curr Microbiol. 1996;33:123–8.

19. Hurst GDD, Walker LE, Majerus MEN. Bacterial infections of hemocytes associated with the maternally inherited male-killing trait in British populations of the two spot ladybird, *Adalia bipunctata*. J Invertebr Pathol. 1996;68:286–92.

20. Hendry TA, Hunter MS, Baltrus DA. The facultative symbiont *Rickettsia* protects an invasive whitefly against entomopathogenic *Pseudomonas syringae* strains. Appl Environ Microbiol. 2014;80:7161–8.

21. Himler AG, Adachi-Hagimori T, Bergen JE, Kozuch A, Kelly SE, Tabashnik BE, et al. Rapid spread of a bacterial symbiont in an invasive whitefly is driven by fitness benefits and female bias. Science. 2011;332:254–6.

22. Kikuchi Y, Fukatsu T. *Rickettsia* infection in natural leech populations. Microb Ecol. 2005;49:265–71.

23. Kikuchi Y, Sameshima S, Kitade O, Kojima J, Fukatsu T. Novel clade of *Rickettsia* spp. from leeches. Appl Environ Microbiol. 2002;68:999–1004.

24. Küchler SM, Kehl S, Dettner K. Characterization and localization of *Rickettsia* sp. in water beetles of genus Deronectes (Coleoptera: Dytiscidae). FEMS Microbiol Ecol. 2009;68:201–11.

25. Dyková I, Veverková M, Fiala I, Macháčková B, Pecková H. *Nuclearia pattersoni* sp. n. (Filosea), a new species of amphizoic amoeba isolated from gills of roach (Rutilus rutilus), and its Rickettsial endosymbiont. Folia Parasitol (Praha). 2003;50:161–70.

26. Galindo LJ, Torruella G, Moreira D, Eglit Y, Simpson AGB, Völcker E, et al. Combined cultivation and single-cell approaches to the phylogenomics of nucleariid amoebae, close relatives of fungi. Philos Trans R Soc B Biol Sci. 2019;374:20190094.

27. Pilgrim J, Ander M, Garros C, Baylis M, Hurst GDD, Siozios S. Torix group *Rickettsia* are widespread in Culicoides biting midges (Diptera: Ceratopogonidae), reach high frequency and carry unique genomic features. Environ Microbiol. 2017;19:4238–55.

28. Thongprem P, Davison HR, Thompson DJ, Lorenzo-Carballa MO, Hurst GDD. Incidence and diversity of Torix Rickettsia–Odonata symbioses. Microb Ecol. 2020; DOI:10.1007/s00248-020-01568-9

29. Weinert LA. The diversity and phylogeny of Rickettsia. In: Morand S, Krasnov BR, Littlewood DTJ, editors. Parasite diversity and diversification. Cambridge: Cambridge University Press; 2015. p. 150–81.

30. Lagrue C, Joannes A, Poulin R, Blasco-Costa I. Genetic structure and host-parasite co-divergence: evidence for trait-specific local adaptation. Biol J Linn Soc. 2016;118:344–58.

31. Řezáč M, Gasparo F, Král J, Heneberg P. Integrative taxonomy and evolutionary history of a newly revealed spider *Dysdera ninnii* complex (Araneae: Dysderidae). Zool J Linn Soc. 2014;172:451–74.

32. Ceccarelli FS, Haddad CR, Ramírez MJ. Endosymbiotic Rickettsiales (Alphaproteobacteria) from the spider genus Amaurobioides (Araneae: Anyphaenidae). J Arachnol. 2016;44:251–3.

33. Park E, Poulin R. Widespread Torix *Rickettsia* in New Zealand amphipods and the use of blocking primers to rescue host COI sequences. Sci Rep. 2020;10:16842.

34. Smith MA, Bertrand C, Crosby K, Eveleigh ES, Fernandez-Triana J, Fisher BL, et al. Wolbachia and DNA barcoding insects: Patterns, potential, and problems. PLoS One. 2012;7:e36514.

35. BOLD: Barcode of Life Data System. 2007. https://www.boldsystems.org/ Accessed 2 January 2018.

36. Smith MA, Pilgrim J, Zakharov E V., Dewaard JR, Ratnasingham S. BOLD contaminant pool (3,817 specimens) data. Figshare. 2020; DOI:10.6084/m9.figshare.12801107

37. Smith MA, Pilgrim J, Zakharov E V., Dewaard JR, Ratnasingham S. BOLD non-contaminant pool (55,366 specimens) data. Barcode Of Life Data System. 2020; DOI:10.5883/DS-RICKET

38. Ratnasingham S, Hebert PDN. BOLD: The Barcode of Life Data System. Mol Ecol Notes. 2007;7:355–64.

39. Davison HR, Siozios S. *Rickettsia* PhyloFlash and Kraken data from arthropod whole genome projects in the Sequence Read Archive. Figshare. 2020; DOI:10.6084/m9.figshare.12801140

40. Gruber-Vodicka HR, Seah BK, Pruesse E. phyloFlash: Rapid small-subunit rRNA profiling and targeted assembly from metagenomes. mSystems. 2020; DOI:10.1128/mSystems.00920-20

41. Hernández-Triana LM, Prosser SW, Rodríguez-Perez MA, Chaverri LG, Hebert PDN, Ryan Gregory T. Recovery of DNA barcodes from blackfly museum specimens (Diptera: Simuliidae) using primer sets that target a variety of sequence lengths. Mol Ecol Resour. 2014;14:508–18.

42. Wang Z, Wu M. An integrated phylogenomic approach toward pinpointing the origin of mitochondria. Sci Rep. 2015;5:7949.

43. Goodacre SL, Martin OY, Thomas CFG, Hewitt GM. Wolbachia and other endosymbiont infections in spiders. Mol Ecol. 2006;15:517–27.

44. Martin OY, Puniamoorthy N, Gubler A, Wimmer C, Bernasconi M V. Infections with *Wolbachia, Spiroplasma*, and *Rickettsia* in the Dolichopodidae and other Empidoidea. Infect Genet Evol. 2013;13:317–30.

45. Machtelinckx T, Van Leeuwen T, Van De Wiele T, Boon N, De Vos WH, Sanchez J-A, et al. Microbial community of predatory bugs of the genus Macrolophus (Hemiptera: Miridae). BMC Microbiol. 2012;12:S9.

46. Lanzoni O, Sabaneyeva E, Modeo L, Castelli M, Lebedeva N, Verni F, et al. Diversity and environmental distribution of the cosmopolitan endosymbiont "Candidatus Megaira". Sci Rep. 2019;9:1179.

47. Blaxter M. Symbiont genes in host genomes: Fragments with a future? Cell Host Microbe. 2007;2:211-3.

48. Gehrer L, Vorburger C. Parasitoids as vectors of facultative bacterial endosymbionts in aphids. Biol Lett. 2012;8:613-5.

49. Le Clec'h W, Chevalier FD, Genty L, Bertaux J, Bouchon D, Sicard M. Cannibalism and predation as paths for horizontal passage of Wolbachia between terrestrial isopods.

50. Ramage T, Martins-Simoes P, Mialdea G, Allemand R, Duplouy A, Rousse P, et al. A DNA barcode-based survey of terrestrial arthropods in the Society Islands of French Polynesia: host diversity within the SymbioCode Project. Eur J Taxon. 2017;272.

51. Wang H, Lei T, Wang X, Maruthi MN, Zhu D, Cameron SL, et al. A newly recorded *Rickettsia* of the Torix group is a recent intruder and an endosymbiont in the whitefly Bemisia tabaci. Environ Microbiol. 2020;22:1207–21.

52. Caspi-Fluger A, Inbar M, Mozes-Daube N, Katzir N, Portnoy V, Belausov E, et al. Horizontal transmission of the insect symbiont *Rickettsia* is plant-mediated. Proc R Soc B Biol Sci. 2012;279:1791–6.

53. Gonella E, Pajoro M, Marzorati M, Crotti E, Mandrioli M, Pontini M, et al. Plant-mediated interspecific horizontal transmission of an intracellular symbiont in insects. Sci Rep. 2015;5:15811.

54. Li Y-H, Ahmed MZ, Li S-J, Lv N, Shi P-Q, Chen X-S, et al. Plant-mediated horizontal transmission of *Rickettsia* endosymbiont between different whitefly species. FEMS Microbiol Ecol. 2017;93.

55. Jaenike J, Polak M, Fiskin A, Helou M, Minhas M. Interspecific transmission of endosymbiotic Spiroplasma by mites. Biol Lett. 2007;3:23–5.

56. Morrow JL, Frommer M, Shearman DCA, Riegler M. Tropical tephritid fruit fly community with high incidence of shared Wolbachia strains as platform for horizontal transmission of endosymbionts. Environ Microbiol. 2014;16:3622–37.

57. Goodacre SL, Martin OY, Bonte D, Hutchings L, Woolley C, Ibrahim K, et al. Microbial modification of host long-distance dispersal capacity. BMC Biol. 2009;7:32.

58. Giorgini M, Bernardo U, Monti MM, Nappo AG, Gebiola M. *Rickettsia* symbionts cause parthenogenetic reproduction in the parasitoid wasp Pnigalio soemius (Hymenoptera: Eulophidae). Appl Environ Microbiol. 2010;76:2589–99.

59. Hagimori T, Abe Y, Date S, Miura K. The first finding of a *Rickettsia* bacterium associated with parthenogenesis induction among insects. Curr Microbiol. 2006;52:97–101.

60. Li K, Chen H, Jiang J, Li X, Xu J, Ma Y. Diversity of bacteriome associated with *Phlebotomus chinensis* (Diptera: Psychodidae) sand flies in two wild populations from China. Sci Rep. 2016;6:36406.

61. Reeves WK, Kato CY, Gilchriest T. Pathogen screening and bionomics of *Lutzomyia apache* (Diptera: Psychodidae) in Wyoming, USA. J Am Mosq Control Assoc. 2008;24:444–7.

62. Song S, Chen C, Yang M, Zhao S, Wang B, Hornok S, et al. Diversity of *Rickettsia* species in border regions of northwestern China. Parasit Vectors. 2018;11:634.

63. Floris R, Yurtman AN, Margoni EF, Mignozzi K, Boemo B, Altobelli A, et al. Detection and identification of *Rickettsia* species in the Northeast of Italy. Vector-Borne Zoonotic Dis. 2008;8:777–82.

64. Yan P, Qiu Z, Zhang T, Li Y, Wang W, Li M, et al. Microbial diversity in the tick *Argas japonicus* (Acari: Argasidae) with a focus on *Rickettsia* pathogens. Med Vet Entomol. 2019;33:327–35.

65. Potts R, Molina I, Sheele JM, Pietri JE. Molecular detection of *Rickettsia* infection in field-collected bed bugs. New Microbes New Infect. 2020;34:100646.

66. Parola P, Paddock CD, Raoult D. Tick-borne Rickettsioses around the world: Emerging diseases challenging old concepts. Clin Microbiol Rev. 2005;18:719–56.

67. Hoffmann AA, Ross PA, Rašić G. Wolbachia strains for disease control: ecological and evolutionary considerations. Evol Appl. 2015;8:751–68.

68. Iturbe-Ormaetxe I, Walker T, O' Neill SL. Wolbachia and the biological control of mosquito-borne disease. EMBO Rep. 2011;12:508–18.

69. van den Hurk AF, Hall-Mendelin S, Pyke AT, Frentiu FD, McElroy K, Day A, et al. Impact of Wolbachia on infection with chikungunya and yellow fever viruses in the mosquito vector Aedes aegypti. PLoS Negl Trop Dis. 2012;6.

70. Kliot A, Cilia M, Czosnek H, Ghanim M. Implication of the bacterial endosymbiont *Rickettsia* spp. in interactions of the whitefly *Bemisia tabaci* with Tomato yellow leaf curl virus. J Virol. 2014;88:5652–60.

71. Tedeschi R, Visentin C, Alam A, Bosco D. Epidemiology of apple proliferation (AP) in northwestern Italy: evaluation of the frequency of AP-positive psyllids in naturally infected populations of *Cacopsylla melanoneura* (Homoptera: Psyllidae). Ann Appl Biol. 2003;142:285-90.

72. Menzel P, Ng KL, Krogh A. Fast and sensitive taxonomic classification for metagenomics with Kaiju. Nat Commun. 2016;7:11257.

73. Katoh K, Standley DM. MAFFT Multiple sequence alignment software version 7: Improvements in performance and usability. Mol Biol Evol. 2013;30:772–80.

74. Castresana J. Selection of conserved blocks from multiple alignments for their use in phylogenetic analysis. Mol Biol Evol. 2000;17:540–52.

75. Wiens J. Missing data and the design of phylogenetic analyses. J. Biomed. Inform. 2006;39:34-42.

76. Kalyaanamoorthy S, Minh BQ, Wong TKF, von Haeseler A, Jermiin LS. ModelFinder: fast model selection for accurate phylogenetic estimates. Nat Methods. 2017;14:587–9.

77. Nguyen LT, Schmidt HA, Von Haeseler A, Minh BQ. IQ-TREE: A fast and effective stochastic algorithm for estimating maximum-likelihood phylogenies. Mol Biol Evol. 2015;32:268–74.

78. Hoang DT, Chernomor O, Haeseler A von, Minh BQ, Vinh LS. UFBoot2: Improving the ultrafast bootstrap approximation. Mol Biol Evol. 2017;35:518–22.

79. He Z, Zhang H, Gao S, Lercher MJ, Chen W-H, Hu S. Evolview v2: an online visualization and management tool for customized and annotated phylogenetic trees. Nucleic Acids Res. 2016;44:W236–41.

80. Fredslund J, Schauser L, Madsen LH, Sandal N, Stougaard J. PriFi: using a multiple alignment of related sequences to find primers for amplification of homologs. Nucleic Acids Res. 2005;33:W516–20.

81. Martin DP, Murrell B, Golden M, Khoosal A, Muhire B. RDP4: Detection and analysis of recombination patterns in virus genomes. Virus Evol. 2015;1:1–5.

82. Chernomor O, von Haeseler A, Minh BQ. Terrace aware data structure for phylogenomic inference from supermatrices. Syst Biol. 2016;65:997–1008.

83. Okonechnikov K, Golosova O, Fursov M. Unipro UGENE: a unified bioinformatics toolkit. Bioinformatics. 2012;28:1166–7.

84. Ewing B, Hillier L, Wendl MC, Green P. Base-calling of automated sequencer traces using Phred. I. Accuracy Assessment. Genome Res. 1998;8:175–85.

85. Wood DE, Lu J, Langmead B. Improved metagenomic analysis with Kraken 2. Genome Biol. 2019;20:257.

86. Kölsch G, Synefiaridou D. Shared ancestry of symbionts? Sagrinae and Donaciinae (Coleoptera, Chrysomelidae) harbor similar bacteria. Insects. 2012;3:473–91.

87. Li K, Stanojević M, Stamenković G, Ilić B, Paunović M, Lu M, et al. Insight into diversity of bacteria belonging to the order Rickettsiales in 9 arthropods species collected in Serbia. Sci Rep. 2019;9:18680.

88. Murakami T, Segawa T, Bodington D, Dial R, Takeuchi N, Kohshima S, et al. Census of bacterial microbiota associated with the glacier ice worm *Mesenchytraeus solifugus*. FEMS Microbiol Ecol. 2015;91.

89. Noda H, Watanabe K, Kawai S, Yukuhiro F, Miyoshi T, Tomizawa M, et al. Bacteriome-associated endosymbionts of the green rice leafhopper *Nephotettix cincticeps* (Hemiptera: Cicadellidae). Appl Entomol Zool. 2012;47:217–25.

90. Zheng Z, Wang D, He H, Wei C. Bacterial diversity of bacteriomes and organs of reproductive, digestive and excretory systems in two cicada species (Hemiptera: Cicadidae). PLoS One. 2017;12:e0175903.

91. Kobiałka M, Michalik A, Świerczewski D, Szklarzewicz T. Complex symbiotic systems of two treehopper species: *Centrotus cornutus* (Linnaeus, 1758) and Gargara genistae (Fabricius, 1775) (Hemiptera: Cicadomorpha: Membracoidea: Membracidae). Protoplasma. 2020;257:819–31.

92. Zouache K, Voronin D, Tran-Van V, Mavingui P. Composition of bacterial communities associated with natural and laboratory populations of *Asobara tabida* infected with Wolbachia. Appl Environ Microbiol. 2009;75:3755–64.

93. Gualtieri L, Nugnes F, Nappo AG, Gebiola M, Bernardo U. Life inside a gall: closeness does not favour horizontal transmission of *Rickettsia* between a gall wasp and its parasitoid. FEMS Microbiol Ecol. 2017;93.

94. Gerth M, Wolf R, Bleidorn C, Richter J, Sontowski R, Unrein J, et al. Green lacewings (Neuroptera: Chrysopidae) are commonly associated with a diversity of Rickettsial endosymbionts. Zool Lett. 2017;3:12.

95. Perotti MA, Clarke HK, Turner BD, Braig HR. *Rickettsia* as obligate and mycetomic bacteria. FASEB J. 2006;20:2372–4.

96. Pilgrim J; Thongprem P; Davison HR; Siozios S; Baylis M; Zakharov EV; Ratnasingham S; deWaard JR; Macadam CR; Smith MA; Hurst GDD (2021): Supporting data for "*Torix Rickettsia* are widespread in arthropods and reflect a neglected symbiosis" GigaScience Database. http://dx.doi.org/10.5524/100873

Figure Legends

Figure 1. Workflow of the BOLD project demonstrating the acquisition and fates of contaminant and non-contaminant *COI* barcoding sequences.

Figure 2. Cladogram of the maximum likelihood (ML) tree of 1,126 proteobacteria *COI* contaminants retrieved from a BOLD project incorporating 184,585 arthropod specimens. The tree is based on 561 bp and is rooted with the free-living alphaproteobacteria *Pelagibacter ubique*. Parentheses indicate the number of BOLD contaminants present in each group. Tips are labelled by BOLD processing ID and host arthropod taxonomy. The Rickettsiales genera of *Anaplasma*, *Rickettsia* (collapsed node), *Orientia* and *Wolbachia* supergroups (A, B, E and F), as well as the Legionellales genera *Legionella* and *Rickettsiella*, are included as reference sequences (Accession numbers: Additional file 10).

Figure 3. Cladogram of a maximum likelihood (ML) tree of 753 *COI Rickettsia* contaminants retrieved from a BOLD project incorporating 184,585 arthropod specimens. The tree is based on 561 bp and is rooted by the *Rickettsia* endosymbiont of *Ichthyophthirius multifiliis* (Candidatus Megaira) using the TVM+F+I+G4 model. Parentheses indicate the number of BOLD contaminants present in Torix

and non-Torix *Rickettsia* groups. Tips are labelled by BOLD processing ID and host arthropod taxonomy. The *Rickettsia* groups: Spotted fever, Transitional, Belli, Typhus, Rhyzobius and Torix are included as references (Accession numbers: Additional file 10).

Figure 4. Phylogram of the maximum likelihood (ML) tree of 99 *COI Rickettsia* contaminants (prefix “BIOUG”) used for further phylogenetic analysis and 53 Non-BOLD reference profiles (Accession numbers: Additional file 10). The tree is based on the concatenation of 4 loci; *16S rRNA*, *17KDa*, *gltA* and *COI* under a partition model*,* with profiles containing at least 3 out of 4 sites included in the tree (2,834 bp total) and is rooted by *Rickettsia* endosymbiont of *Ichthyophthirius multifiliis* (*Candidatus* Megaira). Tips are labelled by host arthropod taxonomy.

Figure 5. *16S rRNA* and *gltA* concatenated maximum likelihood (ML) phylogram (1,834 bp total) including *Rickettsia* hosts from SRA (Triangles) and targeted screens (Stars). The TIM3+F+R2 (16S) and K3Pu+F+G4 (gltA) models were chosen as best fitting models. Rooting is with *Orientia tsutsugamushi*. Accession numbers found in Additional file 10.

Figure 6. Phylogram of a maximum likelihood (ML) tree of *COI Rickettsia* contaminants (prefix “BIOUG”) giving a host barcode and 43 Non-BOLD reference profiles. The tree is based on 4 loci; *16S rRNA*, *17KDa*, *gltA* and *COI* under a partition model with profiles containing at least 2 out of 4 sites included in the tree (2,781 bp total) and is rooted by the *Rickettsia* endosymbiont of *Ichthyophthirius multifiliis* (*Candidatus* Megaira). The habitats and lifestyles of the host are given to the right of the phylogeny. Accession numbers found in Additional file 10.

Additional file information

Additional file 1.docx Taxonomic classification of BOLD non-target *COI* sequences via Kaiju.

Additional file 2.7z Rectangular phylogram trees of cladograms from Figures 2 and 3.

Additional file 3.docx Primer pairs involved in the unintended amplification of 753 *Rickettsia COI* from BOLD project.

Additional file 4.docx Homology of *Rickettsia* groups and *Wolbachia* to the most common forward primers (C_LepFolF and C_LepFolR) attributed to bacterial *COI* amplification from arthropod DNA extracts.

Additional file 5.xlsx Re-barcoding status and nearest BLAST hit of mtDNA *COI* arthropod DNA extracts accessed for further analysis, along with the success of multilocus *Rickettsia* profiles with allocated *Rickettsia* group (based on phylogenetic analysis) and co-infection status.

Additional file 6.docx The barcoding success rate of taxa which gave at least one bacteria *COI* inadvertent amplification (N=51,475 accessible specimens) with an adjusted *Rickettsia* frequency based on an estimated total number of arthropods to account for inaccessible specimens (N=125,402).

Additional file 7.docx Fisher's Exact analyses for comparison of Torix *Rickettsia* infection in aquatic versus terrestrial insects.

Additional file 8.docx GenBank matches mistaken for true mtDNA barcodes and their homology to *Rickettsia COI* (Accessed 29th June 2020).

Additional file 9.pdf Phylogram of a maximum likelihood (ML) tree of *COI Rickettsia* found in the GenBank database erroneously identified as mtDNA barcodes based on 577 bp. The HKY+F+G4 model was chosen as the best fitting model using Modelfinder with the Bayesian information criterion (BIC).

Additional file 10.xlsx Accession numbers used for phylogenetic analyses (Figures 2, 3, 4 ,5 and 6). Accession numbers generated in this study are marked in BOLD.

Additional file 11.docx Mitochondrial *COI* and bacterial gene primers used for re-barcoding and multilocus phylogenetic analyses.

Figure 1

Click here to access/download;Figure;Figure 1.tif

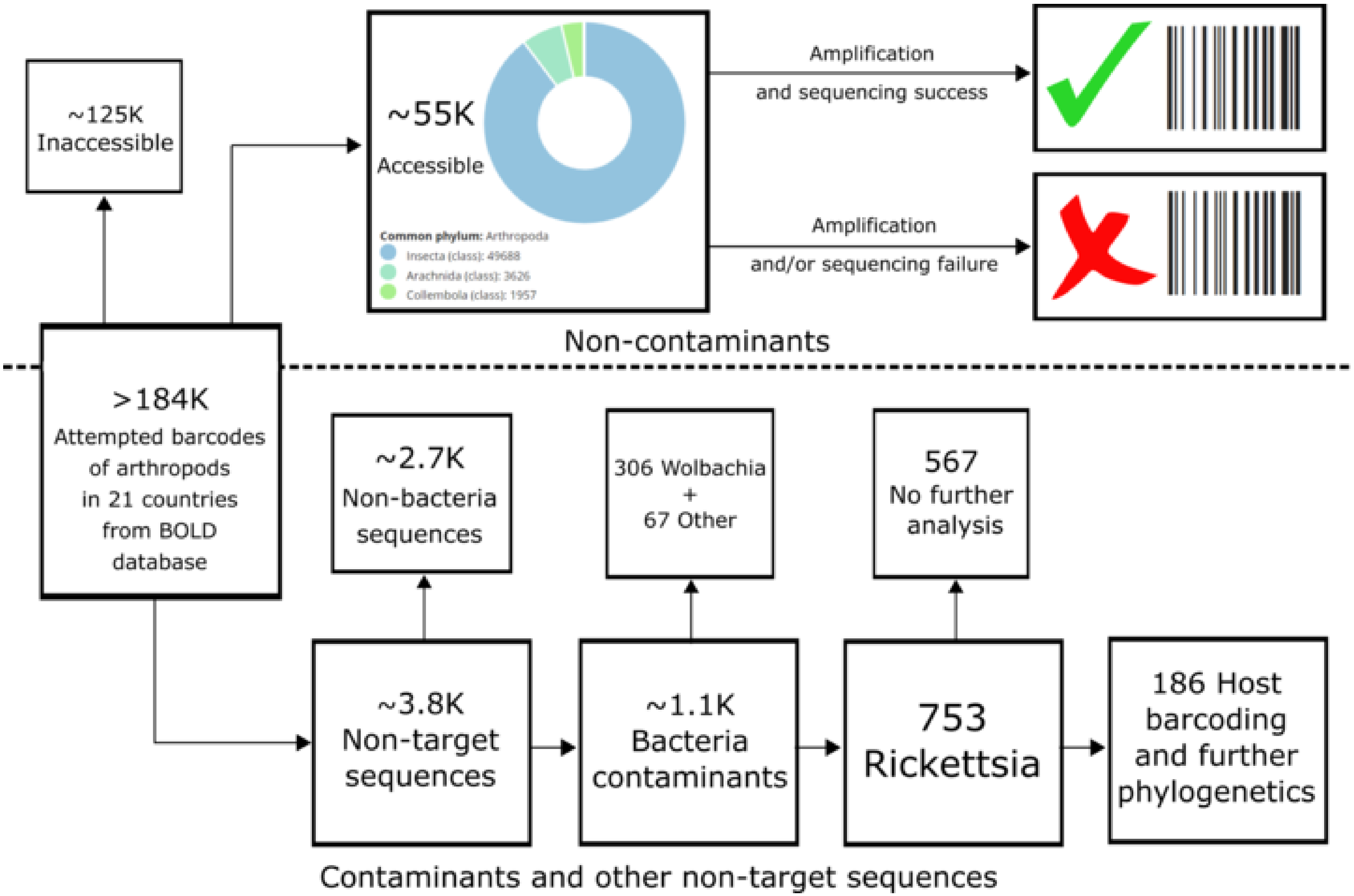

Figure 2

Click here to access/download;Figure;Figure 2.png

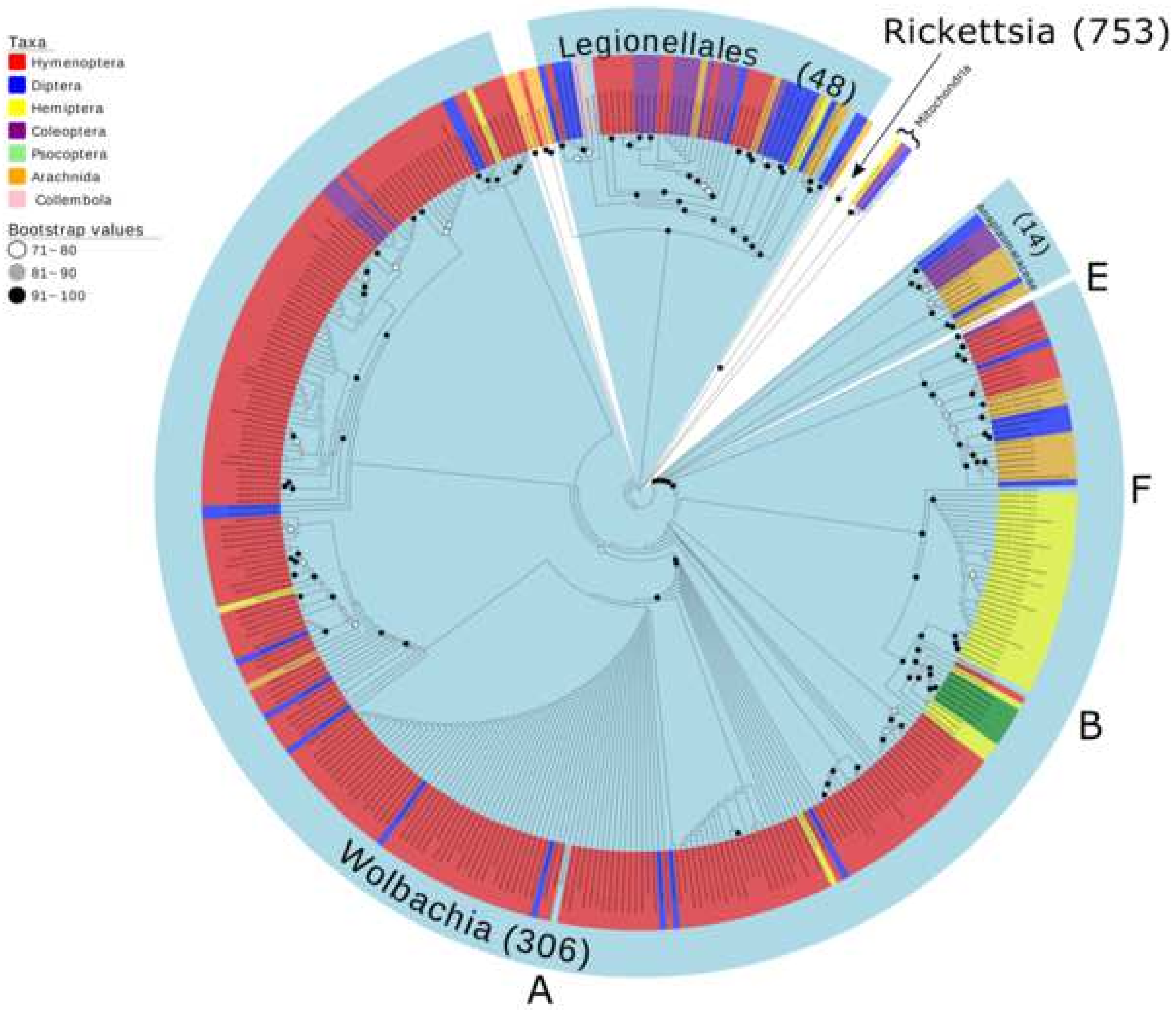

Figure 3

Click here to access/download;Figure;Figure 3.png

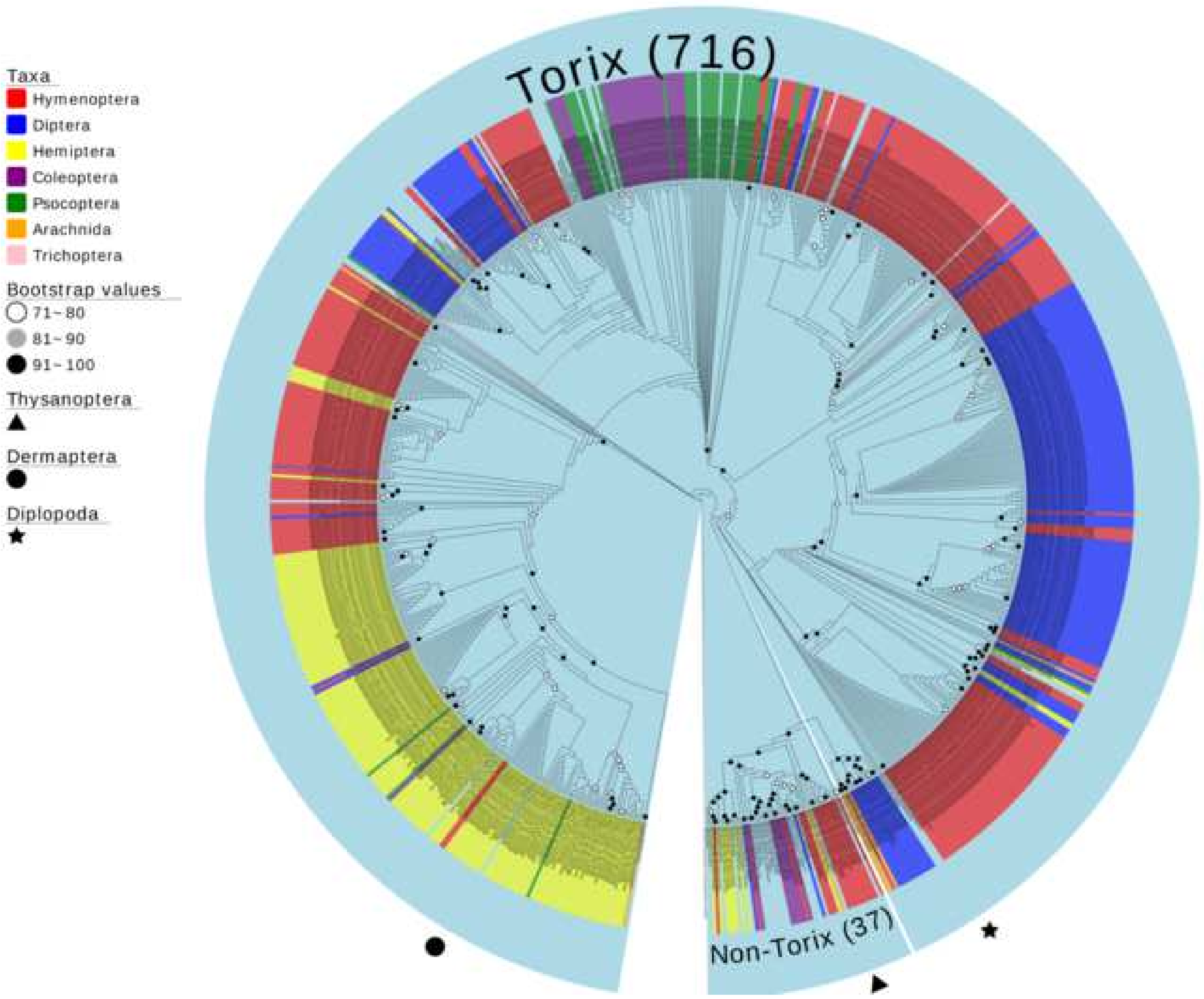

Click here to access/download;Figure;Figure 4.png

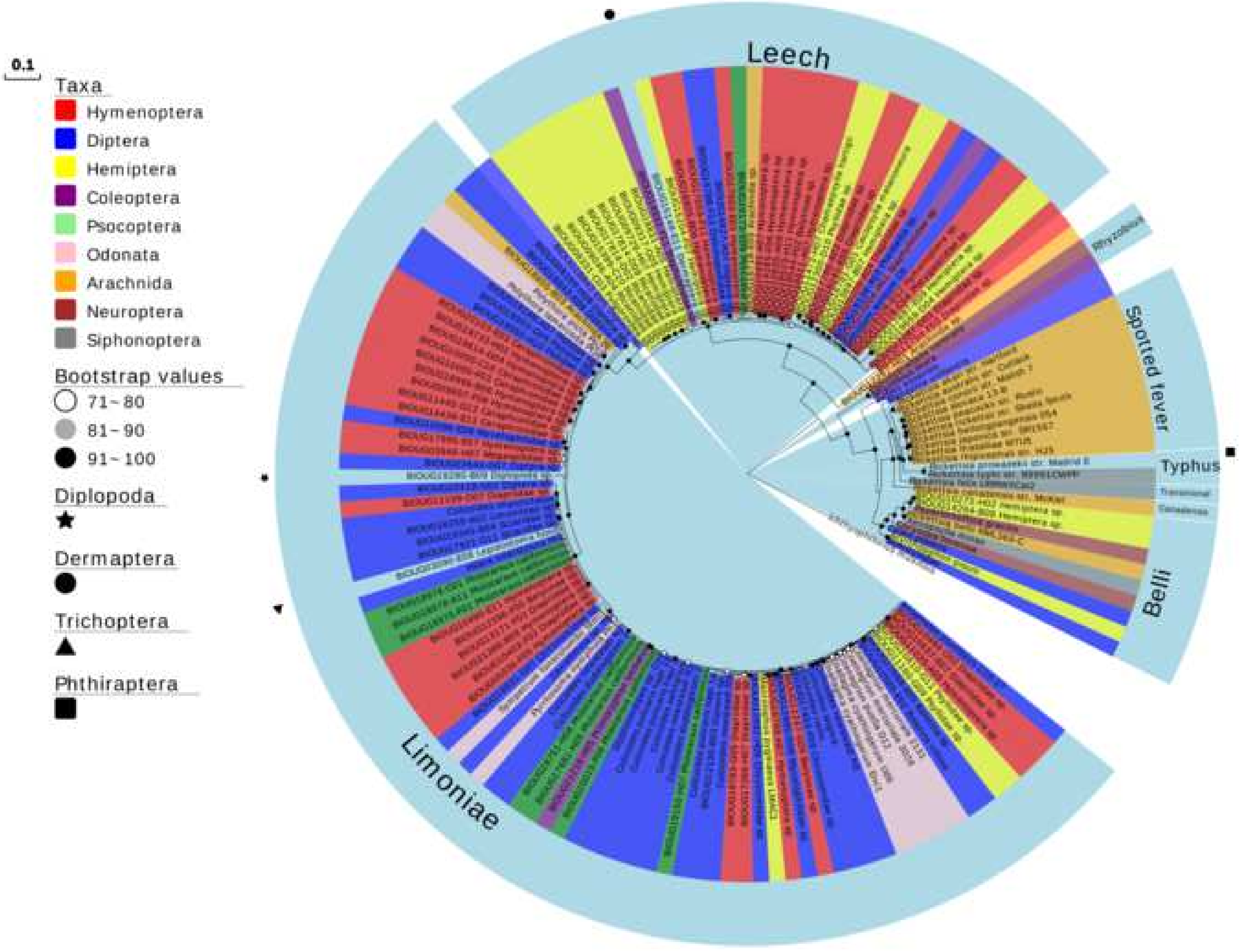

Figure 5

Click here to access/download;Figure;Figure 5 annotated 2.png

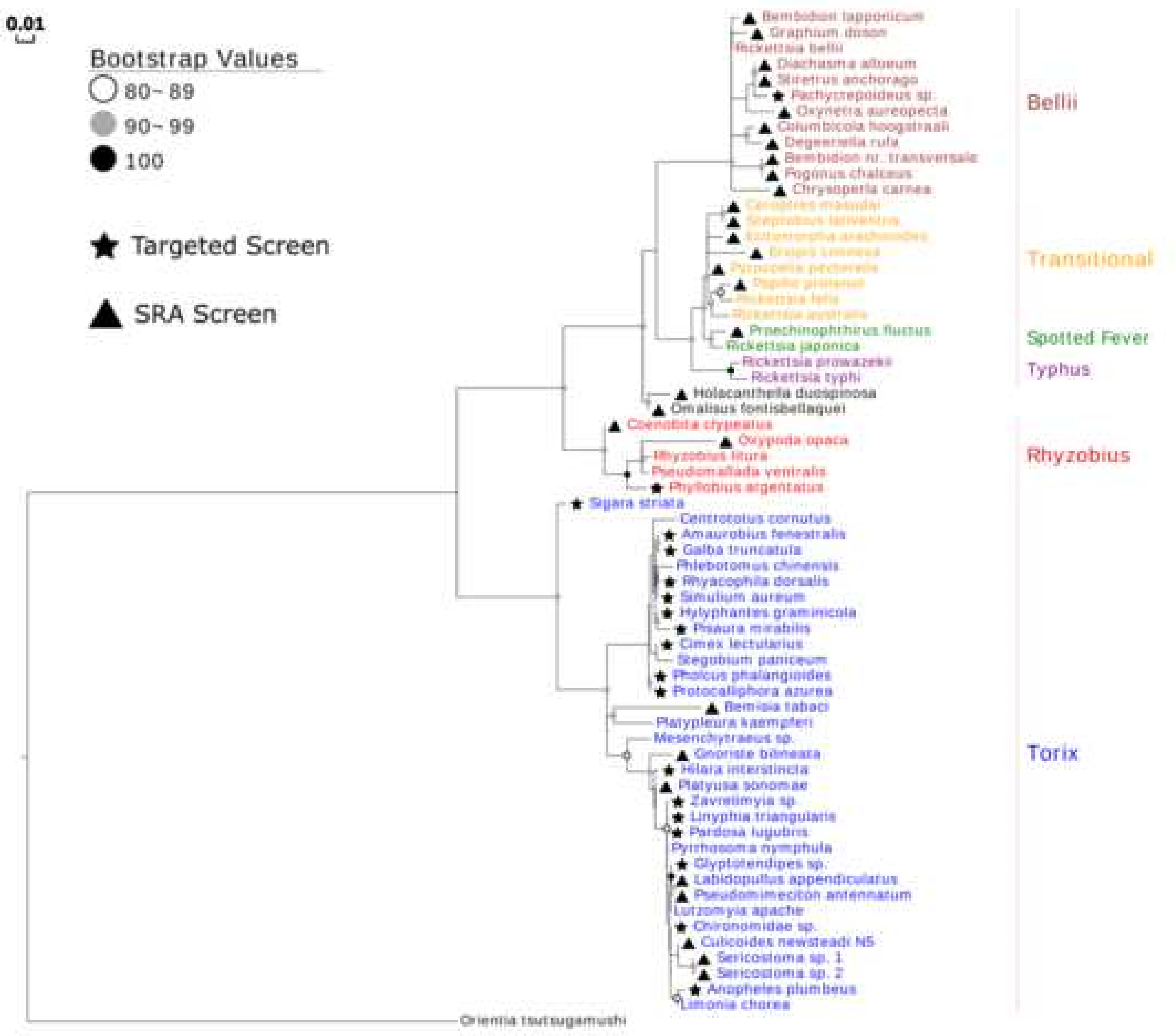

Figure 6

Click here to access/download;Figure;Figure 6.png

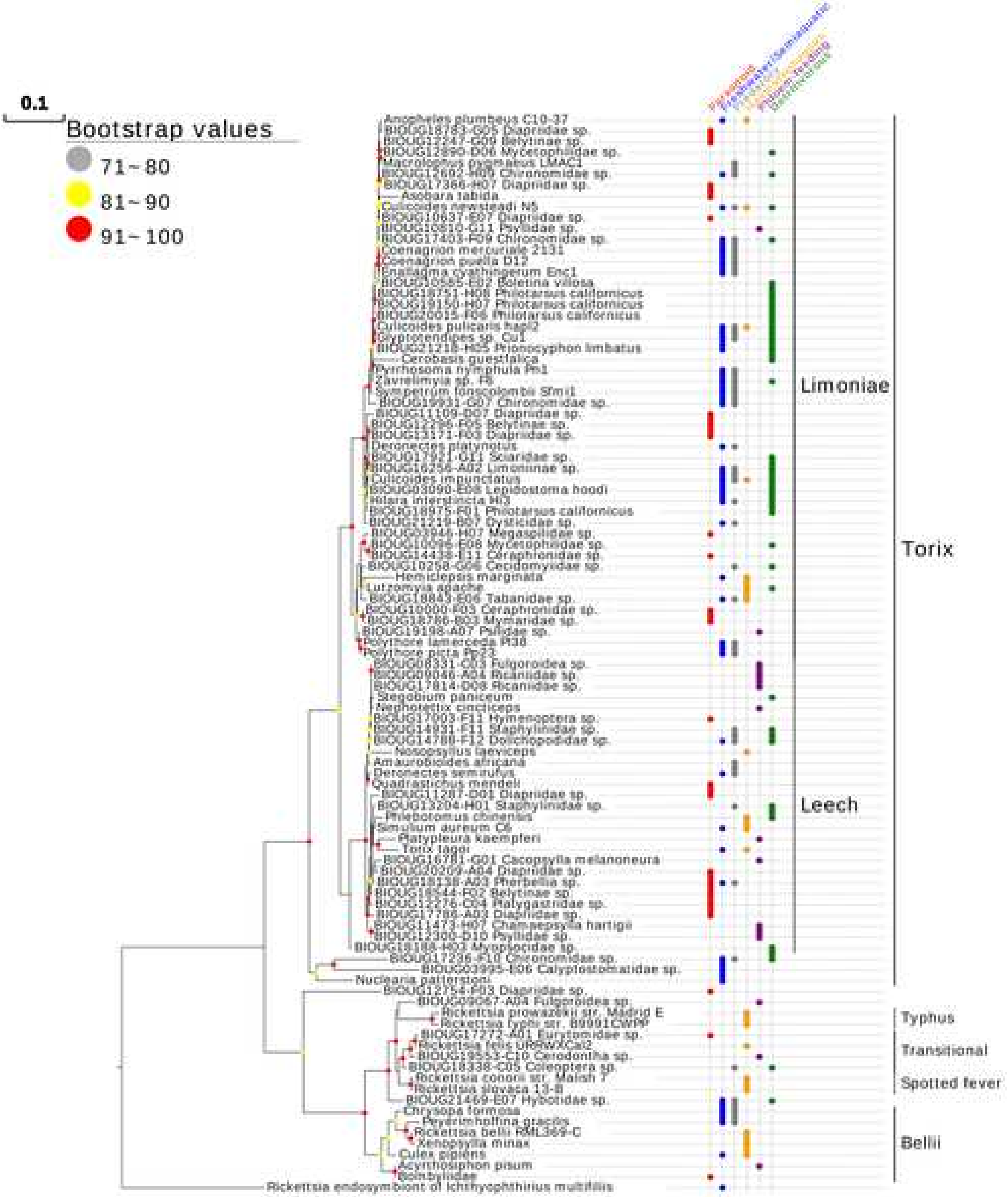

Additional file 1

Click here to access/download
Supplementary Material
Additional file 1.docx

Additional file 2

Click here to access/download
Supplementary Material
Additional file 2.7z

Additional file 3

Click here to access/download
Supplementary Material
Additional file 3.docx

Click here to access/download
Supplementary Material
Additional file 4.docx

Additional file 5

Click here to access/download
Supplementary Material
Additional file 5.xlsx

Click here to access/download
Supplementary Material
Additional file 6.docx

Additional file 7

Click here to access/download
Supplementary Material
Additional file 7.docx

Additional file 8

Click here to access/download
Supplementary Material
Additional file 8.docx

Additional file 9

Click here to access/download
Supplementary Material
Additional file 9.pdf

Additional file 10

Click here to access/download

Supplementary Material

Additional file 10.xlsx

Additional file 11

Click here to access/download
Supplementary Material
Additional file 11.docx
