# Supplementary material for: Torix Rickettsia are widespread in arthropods and reflect a neglected symbiosis
Source: Gigascience. 2021 Mar 25;10(3):giab021. doi: 10.1093/gigascience/giab021 (PMC7992394; doi:10.1093/gigascience/giab021)
Supplement: giab021_Supplemental_Files [file giab021_supplemental_files.zip › Additional file 1.docx]

**Taxonomic classification of BOLD non-target *COI* sequences via Kaiju**


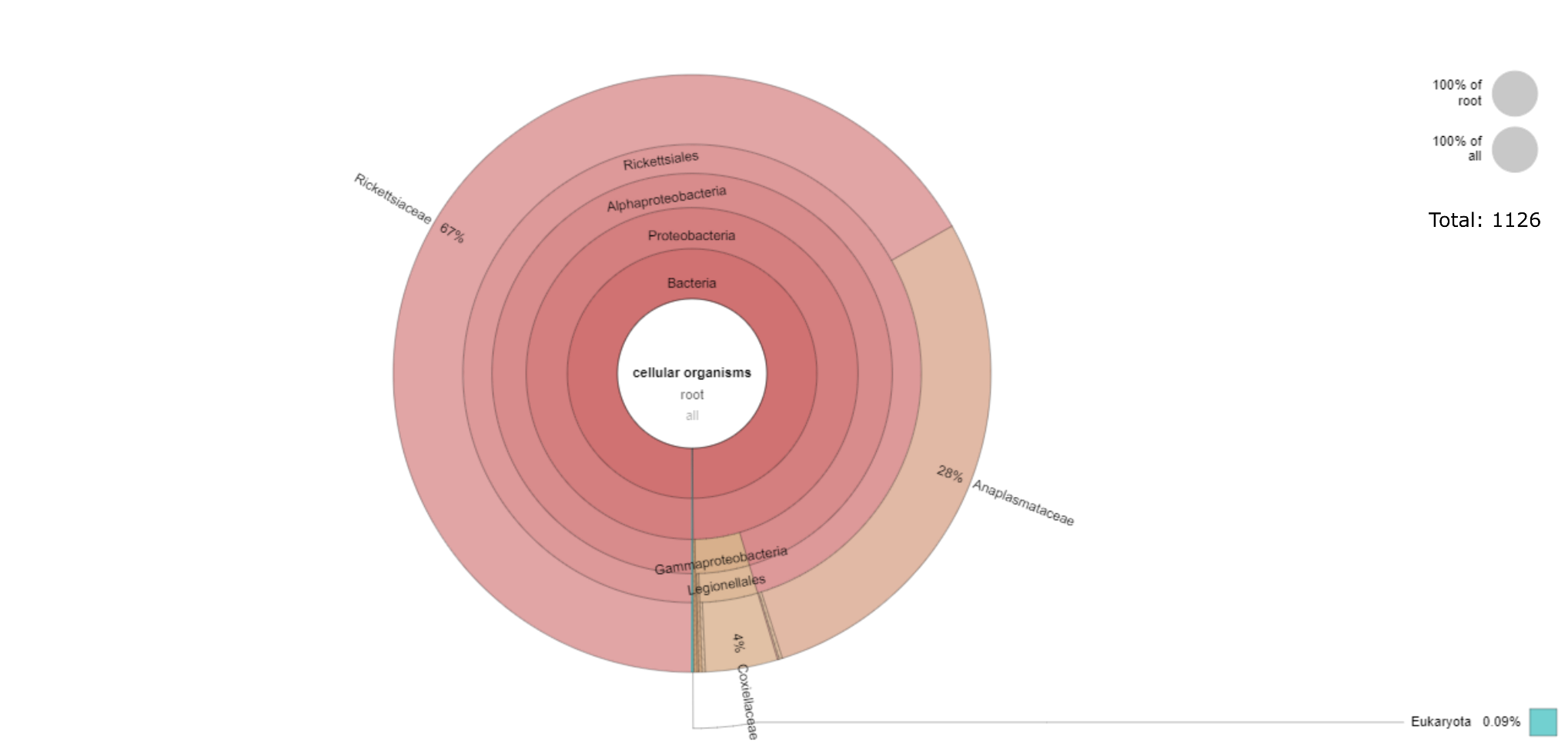


**Additional file 1A**. Kaiju Krona plot of 1,126 sequences filtered from a total of 184,585 BOLD submissions deemed by BOLD’s filtering algorithm to be bacteria. All sequences, except one (BIOUG19659-D04: ‘Eukaryota’), were confirmed by Kaiju as bacteria. However, this sequence was later confirmed as *Rickettsia* through phylogenetic placement (See Figure 3 and Additional file 2).


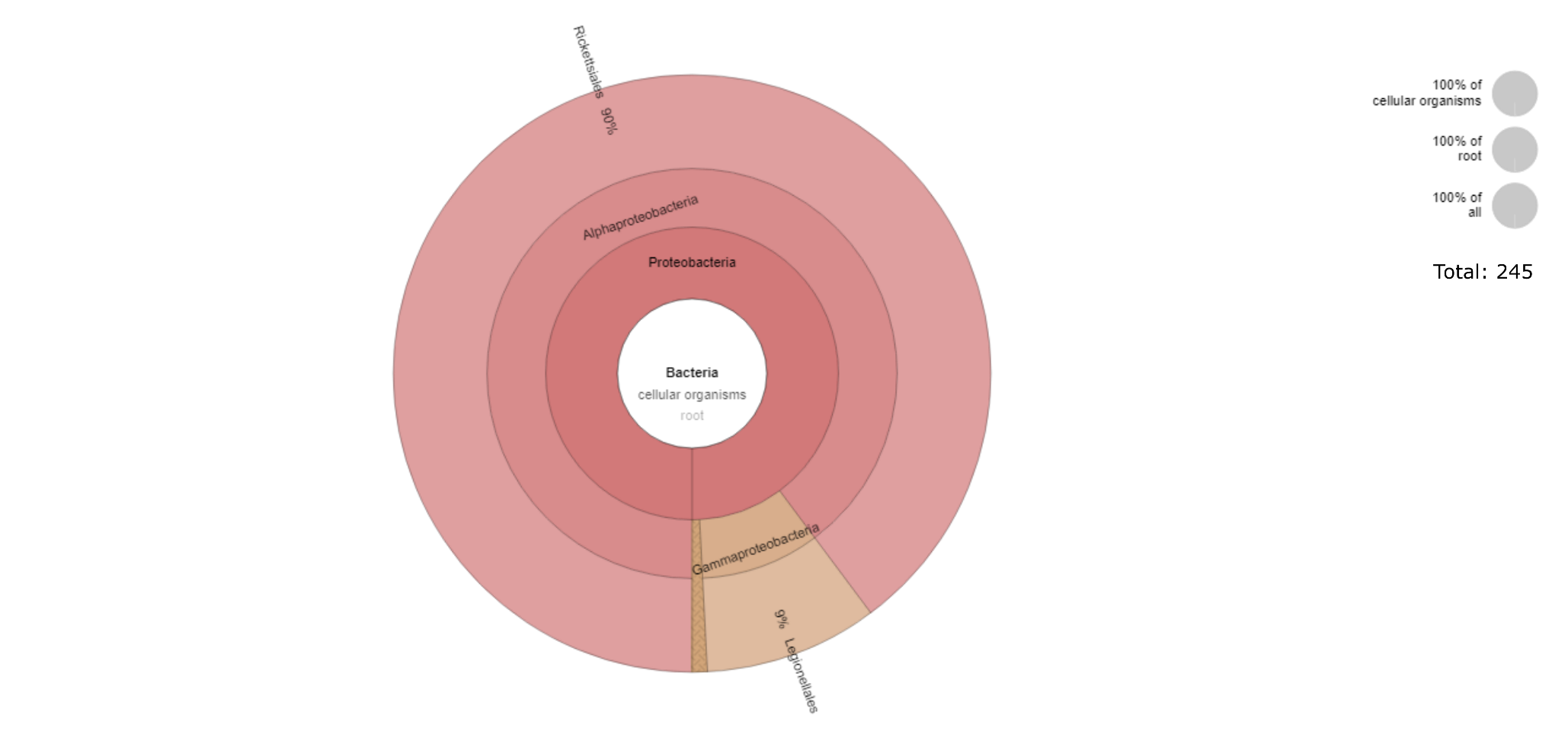


**Additional file 1B**. Kaiju Krona plot of 245 sequences identified as bacteria from a 55,345 subset (initially deemed as not containing non-target amplicons) from 184,585 BOLD submissions.
