# Supplementary material for: Torix Rickettsia are widespread in arthropods and reflect a neglected symbiosis
Source: Gigascience. 2021 Mar 25;10(3):giab021. doi: 10.1093/gigascience/giab021 (PMC7992394; doi:10.1093/gigascience/giab021)
Supplement: giab021_Supplemental_Files [file giab021_supplemental_files.zip › Additional file 11.docx]

| **Gene** | **Product** | **Primer name** | **Sequence (5’-3’)** | **Product length (bp)** | **Reference** |
| --- | --- | --- | --- | --- | --- |
| *COI* | Cytochrome c oxidase, subunit I | C1‐J‐1718 | GGAGGATTTGGAAATTGATTAGT | 455 | Folmer *et al.* 1994; Dallas *et al.* 2003 |
|  |  | HCO1490 | TAAACTTCAGGGTGACCAAAAAATCA |  |  |
|  |  | LCOt_1490 | TGTAAAACGACGGCCAGTGGTCAACAAATCATAAAGATATTGG | 310 | Folmer *et al.* 1994; Hajibabaei *et al.* 2006 |
|  |  | MLepR1 | CCTGTTCCAGCTCCATTTTC |  |  |
|  |  | LepF1 | ATTCAACCAATCATAAAGATAT | 320 | Hebert *et al.* 2004; Fisher and Smith 2008 |
|  |  | C_ANTMR1D_RonIIdeg_R | GGRGGRTARAYAGTTCATCCWGTWCC |  |  |
| *gltA* | Citrate synthase | RiGltA405_F | GATCATCCTATGGCA | 786 | Pilgrim *et al.* 2017 |
|  |  | RiGltA1193_R | TCTTTCCATTGCCCC |  |  |
| *17KDa* | 17KDa antigenic protein precursor | Ri_Meg17kD_F | TGGYATGAATAARCAAGGTGG | 319 | This study |
|  |  | Ri_Meg17kD_R | ATACTCACGACAATAYTGCCC |  |  |
| *16S* | 16S ribosomal RNA | Ri170_F | GGGCTTGCTCTAAATTAGTTAGT | 1170 | Pilgrim *et al.* 2017 |
|  |  | Ri1500_R | ACGTTAGCTCACCACCTTCAGG |  |  |

**Additional file 11.** Mitochondrial *COI* and bacterial gene primers used for re-barcoding and multilocus phylogenetic analyses.

**References:**

Fisher, B. L., & Smith, M. A. (2008). A Revision of Malagasy Species of Anochetus Mayr and Odontomachus Latreille (Hymenoptera: Formicidae). *PLoS ONE*, *3*(5), e1787. doi: 10.1371/journal.pone.0001787

Folmer, O., Black, M., Hoeh, W., Lutz, R., & Vrijenhoek, R. (1994). DNA primers for amplification of mitochondrial cytochrome c oxidase subunit I from diverse metazoan invertebrates. *Molecular Marine Biology and Biotechnology*, *3*(5), 294–299. doi: 10.1371/journal.pone.0013102

Hajibabaei, M., Janzen, D. H., Burns, J. M., Hallwachs, W., & Hebert, P. D. N. (2006). DNA barcodes distinguish species of tropical Lepidoptera. *Proceedings of the National Academy of Sciences*, *103*(4), 968–971. doi: 10.1073/pnas.0510466103

Hebert, P. D. N., Penton, E. H., Burns, J. M., Janzen, D. H., & Hallwachs, W. (2004). Ten species in one: DNA barcoding reveals cryptic species in the neotropical skipper butterfly Astraptes fulgerator. *Proceedings of the National Academy of Sciences*, *101*(41), 14812–14817. doi: 10.1073/pnas.0406166101

Pilgrim, J., Ander, M., Garros, C., Baylis, M., Hurst, G. D. D., & Siozios, S. (2017). Torix group Rickettsia are widespread in Culicoides biting midges (Diptera: Ceratopogonidae), reach high frequency and carry unique genomic features. *Environmental Microbiology*, *19*(10), 4238–4255. doi: 10.1111/1462-2920.13887
