# Supplementary material for: Torix Rickettsia are widespread in arthropods and reflect a neglected symbiosis
Source: Gigascience. 2021 Mar 25;10(3):giab021. doi: 10.1093/gigascience/giab021 (PMC7992394; doi:10.1093/gigascience/giab021)
Supplement: giab021_Supplemental_Files [file giab021_supplemental_files.zip › Additional file 3.docx]

| Primer pair | Sequences (5’-3’) | Rickettsia amplification | Reference |
| --- | --- | --- | --- |
| C_LepFolF/ C_lepFolR | F: RKTCAACMAATCATAAAGATATTGG | 89% | Hernández-Triana *et al.*, 2014 |
|  | R: TAAACTTCWGGRTGWCCAAAAAATCA |  |  |
| LepF2_t1 / LepR1 | F: TGTAAAACGACGGCCAGTAATCATAARGATATYGG | 10% | Hebert *et al.*, 2004; Park *et al.*, 2011 |
|  | R: TAAACTTCTGGATGTCCAAAAA |  |  |
| LepF1/ LepR1 | F: ATTCAACCAATCATAAAGATAT | 0.65% | Hebert *et al.*, 2004 |
|  | R: TAAACTTCTGGATGTCCAAAAA |  |  |
| LepF2_t1 / MHemR | F: TGTAAAACGACGGCCAGTAATCATAARGATATYGG | 0.48% | Park *et al.*, 2011 |
|  | R: GGTGGATAAACTGTTCAWCC |  |  |

**Additional file 3.** Primer pairs involved in the unintended amplification of 753 *Rickettsia* *COI* from BOLD projects.

Hebert, P. D. N. *et al.* (2004) ‘Ten species in one: DNA barcoding reveals cryptic species in the neotropical skipper butterfly Astraptes fulgerator’, *Proceedings of the National Academy of Sciences*, 101(41), pp. 14812–14817. doi:10.1073/pnas.0406166101.

Hernández-Triana, L. M. *et al.* (2014) ‘Recovery of DNA barcodes from blackfly museum specimens (Diptera: Simuliidae) using primer sets that target a variety of sequence lengths’, *Molecular Ecology Resources*, 14(3), pp. 508–518. doi:10.1111/1755-0998.12208.

Park, D. S. *et al.* (2011) ‘Barcoding bugs: Dna-based identification of the true bugs (insecta: Hemiptera: Heteroptera)’, *PLoS ONE*. doi:10.1371/journal.pone.0018749.
