# Supplementary material for: Torix Rickettsia are widespread in arthropods and reflect a neglected symbiosis
Source: Gigascience. 2021 Mar 25;10(3):giab021. doi: 10.1093/gigascience/giab021 (PMC7992394; doi:10.1093/gigascience/giab021)
Supplement: giab021_Supplemental_Files [file giab021_supplemental_files.zip › Additional file 4.docx]

| **Sequence 5-3' (accession number)** | **1** | **2** | **3** | **4** | **5** | **6** | **7** | **8** | **9** | **10** | **11** | **12** | **13** | **14** | **15** | **16** | **17** | **18** | **19** | **20** | **21** | **22** | **23** | **24** | **25** |
| --- | --- | --- | --- | --- | --- | --- | --- | --- | --- | --- | --- | --- | --- | --- | --- | --- | --- | --- | --- | --- | --- | --- | --- | --- | --- |
| C_LepFolF (primer) | R | K | T | C | A | A | C | M | A | A | T | C | A | T | A | A | A | G | A | T | A | T | T | G | G |
| Torix (MWZE00000000) | T | C | . | . | T | . | . | . | . | . | . | . | . | C | . | . | . | . | . | . | . | . | . | . | . |
| Bellii (CP000087) | T | . | . | . | T | . | . | . | . | . | C | . | . | G | . | . | G | . | . | . | . | . | C | . | . |
| Spotted fever (CP000848) | T | . | . | . | T |  | . | . | . | . | . | . | . | C | . | . | . | . | . | . | . | . | C | . | . |
| Typhus (CP004888) | T | . | . | . | T | . | . | T | . | . | . | . | . | C | . | . | . | . | . | . | . | . | C | . | . |
| Transitional (CP000053) | T | . | . | . | T | . | . | . | . | . | . | . | . | . | . | . | . | . | . | . | . | . | C | . | . |
| Megaira (unpublished) | T | . | . | . | T | . | . | T | . | . | . | . | . | . | . | . | G | . | . | . | . | . | A | . | . |
| *Wolbachia* supergroup A (AE017196) | T | . | . | . | C | . | . | . | . | . | . | . | . | . | . | . | . | . | . | . | . | . | A | . | . |

**Additional file 4a.** Homology of *Rickettsia* groups and *Wolbachia* to the most common forward primer (C_LepFolF) attributed to bacterial *COI* amplification from arthropod DNA extracts. SNPs are present at the 3’ priming ends of sequences except for Torix *Rickettsia*.

| **Sequence 5-3' (accession number)** | **1** | **2** | **3** | **4** | **5** | **6** | **7** | **8** | **9** | **10** | **11** | **12** | **13** | **14** | **15** | **16** | **17** | **18** | **19** | **20** | **21** | **22** | **23** | **24** | **25** | **26** |
| --- | --- | --- | --- | --- | --- | --- | --- | --- | --- | --- | --- | --- | --- | --- | --- | --- | --- | --- | --- | --- | --- | --- | --- | --- | --- | --- |
| C_LepFolR | T | A | A | A | C | T | T | C | W | G | G | R | T | G | W | C | C | A | A | A | A | A | A | T | C | A |
| Torix (MWZE00000000) | . | . | T | . | . | . | . | . | . | . | . | . | . | . | . | . | . | . | . | . | . | . | . | C | . | . |
| Bellii (CP000087) | . | . | T | . | . | . | . | . | . | . | . | . | . | . | . | . | . | . | . | . | . | . | . | C | . | . |
| Spotted fever (CP000848) | . | . | T | . | . | . | . | . | . | . | . | . | . | . | . | . | . | G | . | . | . | . | . | C | . | . |
| Typhus (CP004888) | . | . | T | . | . | . | . | . | . | . | . | . | . | . | . | . | . | . | . | . | . | . | . | C | . | . |
| Transitional (CP000053) | . | . | T | . | . | . | . | . | . | . | . | . | . | . | . | . | . | G | . | . | . | . | . | C | . | . |
| Megaira (unpublished) | . | . | . | . | . | C | . | . | . | . | . | . | . | . | C | . | . | . | . | . | C | . | . | C | . | . |
| Wolbachia supergroup A (AE017196) | . | . | . | . | . | . | . | . | . | . | . | . | . | . | . | . | . | . | . | . | . | . | . | C | . | . |

**Additional file 4b.** Homology of *Rickettsia* groups and *Wolbachia* to the most common forward reverse primer (C_LepFolR) attributed to bacterial *COI* amplification from arthropod DNA extracts.
