# Supplementary material for: Torix Rickettsia are widespread in arthropods and reflect a neglected symbiosis
Source: Gigascience. 2021 Mar 25;10(3):giab021. doi: 10.1093/gigascience/giab021 (PMC7992394; doi:10.1093/gigascience/giab021)
Supplement: giab021_Supplemental_Files [file giab021_supplemental_files.zip › Additional file 6.docx]

| **Barcoding success rate of an accessible subsample of total screened taxa containing at least one *Rickettsia* *COI* sequence (N=51,475)** | | | **Estimated total screened taxa containing at least one *Rickettsia COI* and adjusted *Rickettsia* frequency** | | |
| --- | --- | --- | --- | --- | --- |
| Taxa | Specimens (n) | Barcoding success rate | Estimated total specimens (n) | *Rickettsia* (n) | Adjusted *Rickettsia* frequency |
| Hymenoptera | 23,873 | 74.6% | 79,590 | 292 | 0.37% |
| Diptera | 10,062 | 93.3% | 33,545 | 189 | 0.56% |
| Hemiptera | 9,098 | 68.0% | 30,331 | 177 | 0.58% |
| Arachnida | 3,626 | 67.2% | 12,088 | 7 | 0.06% |
| Coleoptera | 3,544 | 83.8% | 11,815 | 40 | 0.34% |
| Psocodea | 736 | 88.6% | 2,453 | 41 | 1.67% |
| Thysanoptera | 471 | 79.4% | 1,570 | 1 | 0.06% |
| Trichoptera | 49 | 91.8% | 163 | 4 | 2.45% |
| Dermaptera | 16 | 56.3% | 53 | 1 | 1.89% |

**Additional file 6.** The barcoding success rate of taxa which gave at least one bacteria *COI* inadvertent amplification (N=51,475 accessible specimens) with an adjusted *Rickettsia* frequency based on an estimated total number of arthropods to account for inaccessible specimens (N=125,402).
