# Supplementary material for: Torix Rickettsia are widespread in arthropods and reflect a neglected symbiosis
Source: Gigascience. 2021 Mar 25;10(3):giab021. doi: 10.1093/gigascience/giab021 (PMC7992394; doi:10.1093/gigascience/giab021)
Supplement: giab021_Supplemental_Files [file giab021_supplemental_files.zip › Additional file 7.docx]

**Additional file 7: Fisher’s Exact analyses for comparison of Torix *Rickettsia* infection in aquatic versus terrestrial insects**

1. Comparison between all insects.


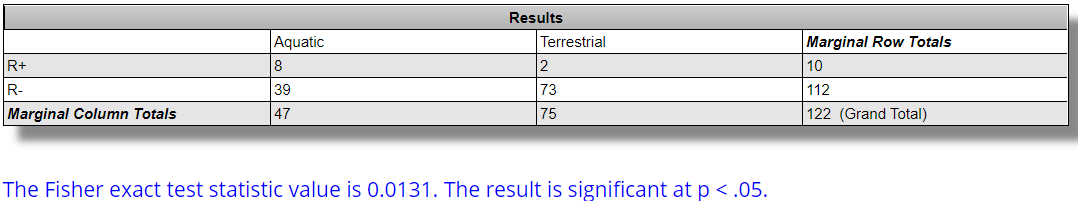


1. Comparison between taxa when controlled for insect orders represented in both aquatic and terrestrial groups (Hemiptera, Diptera, Coleoptera)


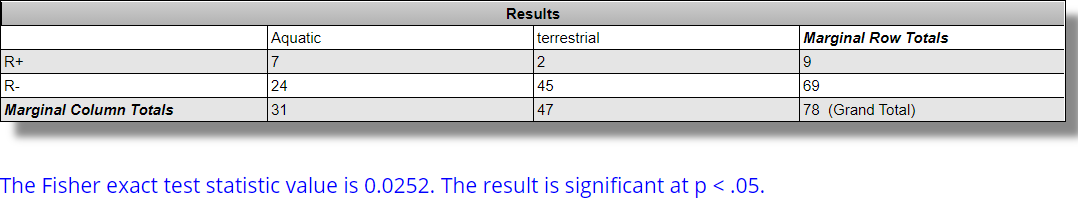


1. Comparison between all invertebrates


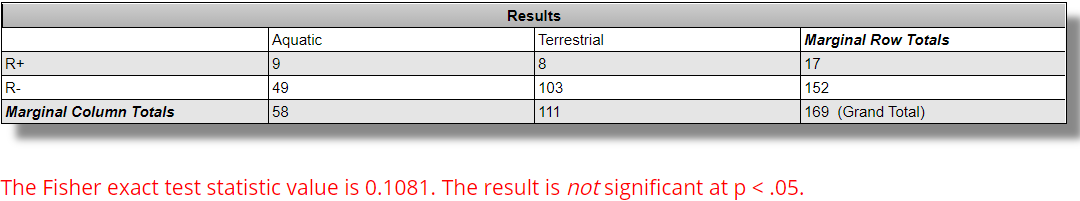

Prevalence of Torix *Rickettsia* in freshwater and terrestrial insects. **A**: the comparisons between freshwater and terrestrial invertebrates from all insect orders. Fisher’s exact test revealed a significant different of the proportion of infected species between the two type of hosts communities (*p*-value = 0.013). **B:** The comparisons between freshwater and terrestrial invertebrates from three insect orders; Hemiptera, Diptera and Coleoptera. The proportion of infected species was significantly different between the two host groups (Fisher’s exact test; *p-value* = 0.025). Error bars represent 95% confidence intervals derived from binomial sampling.
