# Supplementary material for: Torix Rickettsia are widespread in arthropods and reflect a neglected symbiosis
Source: Gigascience. 2021 Mar 25;10(3):giab021. doi: 10.1093/gigascience/giab021 (PMC7992394; doi:10.1093/gigascience/giab021)
Supplement: giab021_Supplemental_Files [file giab021_supplemental_files.zip › Additional file 8.docx]

| **NCBI Host identification** | **Accession number** | ***Rickettsia* BLAST hit** |
| --- | --- | --- |
| *Calopteryx maculata* (Odonata; Calopterygidae) | KM383849 | 100% identity to *Rickettsia* of *Culicoides pulicaris* (KY765405) |
| Eucharitidae sp. (Hymenoptera) | KC182318 | 91% identity to *Rickettsia* of *Amaurobioides africana* (KU600823) |
| Endopterygota sp. | KP422277 | 92% identity to *Rickettsia* of *Amaurobioides Africana* (KU600823) |
| Mandibulata sp. | KP421611 | 91% identity to *Rickettsia* of *Amaurobioides africana* (KU600823) |
| Metcalfa pruinosa (Hemiptera; Flatidae) | MN609260 | 98% identity to *Rickettsia* of *Amaurobioides africana* (KU600823) |
| *Pimelia* sp. (Coleoptera; Tenebrionidae) | MH158030 | 92% identity to *Rickettsia* of *Amaurobioides africana* (KU600823) |
| *Paracalliope fluviatilis* (Amphipoda; Paracalliopiidae) | KR336946 | 92% identity to *Rickettsia* of *Culicoides impunctatus* (KY765403) |
| *Formicidae* sp. (Hymenoptera) | KP421783 | 92% identity to *Rickettsia* of *Culicoides newsteadi* N1 (KY765400) |
| *Flavina* sp. (Hymenoptera; Issidae) | HM452248 | 98% identity to *Rickettsia* of *Amaurobioides africana* (KU600823) |
| *Forficula sp.* (Dermaptera; Forficulidae) | MK644592 | 97% identity to *Rickettsia* of *Amaurobioides africana* (KU600823) |
| *Myce*to*phila lunata* (Diptera; Mycetophilidae) | KM679400 | 98% identity to *Rickettsia* of *Amaurobioides africana* (KU600823) |

**Additional file 8.** Genbank matches mistaken for true mtDNA barcodes and their homology to *Rickettsia* *coxA* (Accessed 29^th^ June 2020).
