# Supplementary figures and images for: Torix Rickettsia are widespread in arthropods and reflect a neglected symbiosis
Source: Gigascience. 2021 Mar 25;10(3):giab021. doi: 10.1093/gigascience/giab021 (PMC7992394; doi:10.1093/gigascience/giab021)

0.1

Bootstrap values

○ 80~89

● 90~100

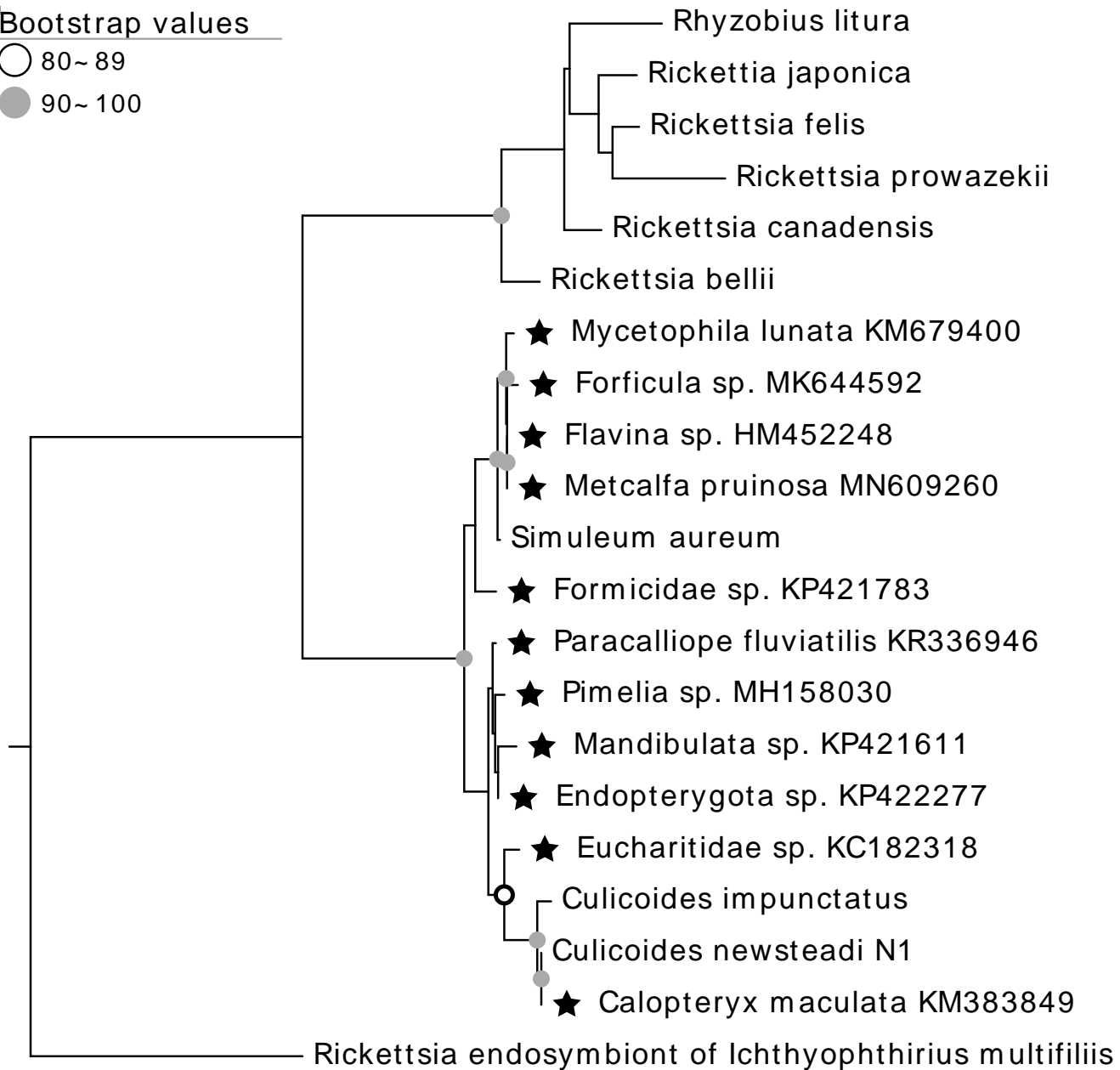

Torix

Supplement: giab021_Supplemental_Files [file giab021_supplemental_files.zip › Additional file 9.pdf]
