# Supplementary material for: Torix Rickettsia are widespread in arthropods and reflect a neglected symbiosis
Source: Gigascience. 2021 Mar 25;10(3):giab021. doi: 10.1093/gigascience/giab021 (PMC7992394; doi:10.1093/gigascience/giab021)
Supplement: giab021_Response_to_Reviewer_Comments_Original_Submission [file giab021_response_to_reviewer_comments_original_submission.pdf]

# Author's Response To Reviewer Comments

Close

Dear Dr. Edmunds,

Thank you for considering a revised version of "Torix Rickettsia are widespread in arthropods and reflect a neglected symbiosis". The authors would like to thank the three reviewers for their time and comments on the manuscript. Please find below a point-by-point response to reviewer comments. Aside from clarificatory points, the major changes to the manuscript include:

• The attempted retrieval of both parasitoid and protist reads from the SRA datasets to ascertain the likelihood of these taxa being responsible for the Rickettsia positives observed in the study.
• The additional analytical step of using the Kaiju bioinformatic tool to confirm COI sequences from the BOLD dataset as being bacterial.
• A more detailed analysis of comparing the presence of Torix Rickettsia in aquatic and terrestrial biomes.
• The inclusion of phylograms for figures 2 and 3 to avoid confusion over long branch attractions.

Reviewer#1
My only concern is that Torix group Rickettsia and their relatives have also been identified in protists, such as nucleariid amoebae. So I wonder how many of these Rickettsia, particularly in aquatic hosts, are symbionts of protists residing in animal guts. Have the authors tried to pull out protist 18S sequences from the SRA datasets (or tried to amplify protist genes via PCR, although that would be much more difficult)?

We thank the reviewer for this insight which we agree with. phlyoFLash analysis retrieved 16S (microbe) and 18S (eukaryote) sequences for each SRA dataset where present, and we have now included this information on the FTP server under the directory name "phyloFlash html files". One instance of an assembled parasitoid 18S rRNA sequence was found in dataset ID SRR6313831 from Bemisia tabaci. However, a B. tabaci-Rickettsia true endosymbiosis has already been confirmed though FISH imaging (Wang et al. 2020; doi:10.1111/1462-2920.14927) suggesting the parasitoid is likely not responsible for the presence of Rickettsia in this case.

Protist sequences were also identified in some of the SRA datasets but these were a significant minority of reads compared to Rickettsia reads (doi:10.6084/m9.figshare.12801140). Intriguingly, one of the highest numbers of protist reads came from our previous study (SRA dataset SRR5298327) which was shown by FISH to be a true endosymbiosis between insect and Rickettsia (Pilgrim et al. 2017; doi:10.1111/1462-2920.13887). Overall, these data suggest that detecting contamination from Rickettsia-infected protists or parasitoids is uncommon. This new information has been added on lines 274-281, 355-364 and 576-578.

Minor comments:

Line 194 - Psyllidae spelling

Line 242 & Table 2 - Chaoboridae spelling

Line 251 - Simulium spelling

Spellings of these taxa have been now rectified.

Lines 340 - I would replace refs 49 and 50 with Gehrer & Vorburger, Biol. Lett., 2012

The references have now been changed per the reviewer's suggestion.

Line 362 - this sentence is confusing because the citations refer to Rickettsia in the belli group

For clarity the sentence has been changed to specify the references refer to the belli group only (line 417).

Table 2 - Siphonaptera spelling

Line 819 - Parentheses spelling

These spellings have now been changed.

Reviewer#2
Abstract 38, 42-43: the introduction of the "aquatic hotspot" hypothesis and that the results were supporting this hypothesis was very appealing (l38), yet this was not addressed in the conclusion, which instead claimed that Rickettsia was associated with a number of habits (l42-43). As these habits were not linked to aquatic, and not introduced previously in the background, the logic flow here is rather difficult to follow.

We thank the reviewer for flagging this. We have now changed the conclusion of the abstract to show that new hotspots of infection were revealed as well as confirming a bias towards aquatic insects (lines 44-47).

69: Rickettsia has been estimated as being present in 20-24% of species. One would be very interested in learning whether this is confirmed/disapproved by the findings of the current study. Which part of the experimental design is set to answer this question? If no, what needs to be done to get a better idea?

The 20-42% prevalence figure for terrestrial arthropod species is derived from model-based estimation techniques which assume populations infected have a minimum of 1/1000 individuals infected. Thus, our figure of ~9% from the targeted PCR screen is likely lower due to small within-species sample sizes. This has been highlighted in lines 366-370.

79-88: It might be a good idea to add something here about the diversity of subgroups of Torix. The results later on revealed two subgroups (Leech and Limoniae), but are these good representatives of the diversity within Torix? How many subgroups are already known?

Previous studies on Torix Rickettsia have highlighted two subgroups: "Leech" and "Limoniae". This was initially based on limited phylogenetic markers but by extension of using multiple markers we confirm in this study that a majority of Torix strains fall into these two subgroups. We have highlighted this on line 85.

90-102: The use of terms Rickettsia CoxA, COI, Rickettsia COI are confusing. If Rickettsia CoxA and Rickettsia COI are actually referring to the same Rickettsia gene, the term needs to be standardized.

We thank the reviewer for making this point. We agree that terms should be standardised as much as possible. Therefore, we have removed any reference to 'CoxA' in the manuscript.

106: does the "template" here refer to DNA extract/aliquot? "Template" in the context of DNA template is primarily used in the description of amplification reaction, which doesn't seem to be the case here. This term is somewhat confusing. As you used "DNA extract" later in the text, I would suggest that these terms be unified.

The term "template" has been swapped for "DNA extract" throughout the manuscript.

109: "function more broadly" here is also vague. Do you mean that the primers used in these PCR assays are more degenerate or specifically designed to target Rickettsia genes? Please clarify.

The primers function more broadly as they were designed from our previous work based on Rickettsia genomes from multiple clades, including the first available Torix genome. This information has been removed from the introduction and is instead clarified in the data description (lines 153-155) and methods (lines 478-480).

123-125: "...deemed as contaminant sequences as a result of not matching initial morphotaxa assignment". I don't think that this is entirely accurate. A significant proportion of barcodes in BOLD are not matching initial morphotaxa assignment, at varied taxonomic levels. These include mis-identification, ambiguous/unstable taxonomic status, lab contaminations, etc. I would assume that BOLD uses an algorithm to confirm the sequence as being contaminants, only when they are matched to the most common non-target contaminants, e.g., bacteria, human etc.

We thank the reviewer for their comment. Yes, this dataset contained both contaminant sequences, as well as misidentified taxa and we have now changed the wording of this sentence to reflect this on line 130-132 and in Figure 1. Information on how contaminants were confirmed as bacterial are also now described in lines 450-465.

125-128: the term "specimens" needs to be clarified. Do these include those that didn't yield a DNA sequence?

Yes-this included some specimens where barcoding had failed to yield a DNA sequence. This has now been clarified on line 126.

142: Explain targeted PCR Rickettsia screen. Does it employ specific primer sets designed for Rickettsia? Although this was described in the method section, a brief explaining of the method would help the readers to understand the context.

Yes, as mentioned above, the primers function more broadly as they were designed from our previous work based on Rickettsia genomes from multiple clades and including the first available Torix genome. This has now been clarified in lines 153-155 and 478-480.

149: Should "Analyses" be "Results"?

The formatting of gigaScience uses "analyses" in place of "results".

160-161: "further unique bacteria contaminants were also detected", where are these results? Please cite.

These results have now been added in Additional file 1 (graphic representation of taxonomic classification as bacteria) and the FTP server file "Kaiju_misc_bacteria_detection" (sequence information). These were sequences flagged as bacterial by the bioinformatics tool Kaiju (lines 173-176).

167-170 : if the BOLD results does not seem to support the aquatic hotspot theory, why?

Both the BOLD and SRA datasets have inherent biases which make them unsuitable to assess whether Torix Rickettsia are more common in aquatic or terrestrial biomes. For example, most SRA submissions are from lab-reared terrestrial insects. Likewise, a majority of the specimens from BOLD containing Rickettsia have limited taxonomic/ecological information, by virtue of not returning an mtDNA COI sequence. Therefore, a PCR-based study targeting both terrestrial and aquatic taxa was implemented in order to specifically test this 'aquatic hot spot hypothesis' (lines 149-158).

170-172: the predominance report of Rickettsia from Canada seems meaningless, given the strongly biased sampling in BOLD (supplementary Fig. 1)

The authors agree. This has now been removed.

180: this is confusing, does it mean that the Torix sequence is identical to that of C_LepFolR at the 3'

end? Or does it have a SNP but different from that of other bacteria?

The Torix sequence has a SNP at the same site as all the other Wolbachia/Rickettsia genomes compared to C_LepFolR at the 3' end. However, all the Wolbachia/Rickettisa genomes assessed apart from the Torix Rickettsia have a SNP at the 3' priming end for C_LepFolF. For clarity, this can be viewed in Additional file 4.

185: How were these 186 Rickettsia-containing samples selected from 753 samples?

These DNA extracts were chosen based on assorted geographic location, host order and diverse phylogenetic placement. This has been clarified on line 196-198.

192: So how many subgroups of Torix are known? How well the findings represent the diversity?

As noted in a previous reply, to date only two subgroups of Torix Rickettsia have been uncovered: "Leech" and "Limoniae". This was initially based on limited phylogenetic markers but by extension of using multiple markers we confirm in this study that a majority of Torix strains fall into these two subgroups. We have highlighted this on line 85.

207: define attempted barcodes

In this context, an "attempted barcode" is an attempt to retrieve a mtDNA COI barcode from the approximately 185,000 arthropods in the study. As mentioned above and indicated in figure 1, not all DNA extracts produced a COI sequence to interpret. Now that the term "specimen" has been clarified on line 126 we have replaced "attempted barcodes" with "specimens" to avoid confusion.

211: Here you used "genomic extracts", is this equivalent to "template"? Try to standardize terms.

We have standardised terms to only "DNA extracts" throughout the manuscript.

217: again, why BOLD taxa with the most presence of Rickettsia NOT associated with aquatic lifestyle?
233-235: why did the comparison between aquatic/terrestrial arthropods only consider the targeted Rickettsia screen results, NOT that of SRA search?

We refer the reviewer back to our earlier response (167-170) to address both of these points.

269-270: This is somewhat misleading. This might imply that these two groups of bacteria cooccur in the same organisms, and the amplification of R is easier than W. I don't think the current experimental design is able to proof or deny this possibility.

The wording has now been changed on lines 310-312 to avoid this confusion.

308-310: we know that there are many other possibilities that might cause barcoding failure. At least provide some alternative causes to avoid biased argument.

We have deleted this argument from the paragraph.

415-416: what are the exact criteria when choosing these DNA templates?

This point has been addressed above (reviewer comment 185)

428: does "linear" mean non-recombined sequence?

In this context, "linear" refers to a parameter of the recombination detection program which refers to the sequences not being circular.

438-439: does this mean that the hosts were NOT identifiable by morphology?

That is correct, the metadata provided for specimens before barcoding is a general morphological classification usually down to the order level. Subsequently, more refined classification can only be achieved from the mtDNA barcode. This has been highlighted on lines 501-504.

459-461: What if the sequence was matched to more than one barcode at >98% identity?

This did not occur.

489-497: Please provide more details on the analysis of phyloFlash, e.g., parameters used. I am a bit concerned about the assembling process employed here. 16S assembling can be difficult/impossible when metagenomics data contain more than 1 bacterial species or multiple variable copies of 16S, both of which might be the case for Rickettsia.

Default parameters were used for phyloFlash (lines 567-578). Phyloflash uses a combination of SPAdes and BBmap to assemble rRNA SSU and references a curated database (SILVA). BBmap cut off for identification is a minimum identity >70% and phyloflash recommends SPAdes as the best method for cases where there may be a lack of close relatives in the reference database. The recent paper (Gruber-Vodika et al. 2020; doi:10.1128/mSystems.00920-20) goes into further details about chimeras, false positives and dataset preparation. While the defaults do what they can to minimise risk of false positives, it cannot be entirely eliminated.

We have attempted to address this by flagging the instances where Wolbachia sequences or other symbionts were also found in the phyloflash notes, though these sequences were not always assembled. This information can be seen in the phyloflash html files on the FTP server.

Table 1: for species without a definite identification to the species level (e.g., Pachycrepoideus sp.), do we know that all specimens analyzed here actually belong to the same species? I assume this can be confirmed using barcodes.

Some arthropods without a definite identification were referred to as “sp.” because barcoding was not successful or did not match any known species in the database (lines 546-547).

Figure legends for Figs. 2 and 3: the term "No colour" is misleading. I thought these would refer to those without any background colors (e.g., Rickettsia lineage in Fig. 2).

We have removed the term “no colour” from the legend.

Fig. 2: So all Rickettsia in this tree were not from non-BOLD reference (says the Fig legend)? If the number in parenthesis represent the number of sequences, why is there only a single tip for Rickettsia? Are they collapsed? If yes, does it mean that the genetic divergence within Rickettsia is much smaller than that within Wolbachia?

Yes, Rickettsia is collapsed and this is now mentioned in the legend (Line 890). Genetic divergence of Rickettsia is deliberately shown in Figure 3 (and Additional file 2) and not in Figure 2 for ease of presentation, due to the number of taxa in the phylogenies.

Fig. 5: Is the lineage distribution associated with methodology used in discovering these sequences (SRA vs. targeted PCR screening)? Provide statistics.

The SRA datasets contain more Belli strains than the targeted screen but this seems irrelevant information as both datasets cannot be reasonably compared. As mentioned above, the SRA dataset contain very few aquatic insects with most depositions deriving from terrestrial insects and/or lab cultivated insects. In contrast, the targeted screen represents mostly wild-caught insects with a mixture of aquatic and terrestrial arthropods. Subsequently, even if it was shown that specific lineages were associated with the two methods for the SRA and targeted screens, it is just a likely that this is due to sampling bias rather than other methodological biases. Thus, our conclusions are measured
1) The BOLD screen demonstrates that Rickettsia (specifically from the Torix group) are overrepresented in barcoding projects and can help identify new hosts.
2) The SRA screen demonstrates that both Torix and Belli clades of Rickettsia are common.
3) The targeted screen provides evidence to suggest Torix Rickettsia are more common in aquatic insects.

Fig. 6: Move the vertical bars representing Typhus, Transitional, Spotted fever, and Bellii, further to the right so that they are in line with that of Torix. My understanding is that these lineages belong to the

same hierarchic level under Rickettsia.

We thank the reviewer for pointing this out and have changed figure 6 accordingly.

Reviewer #3
This study relies heavily on secondary data usage, identifying the presence of Rickettisa symbionts in host samples using discarded data from the BOLD database. This is great, and we should have more studies like this. However, largely, the authors fail to discuss the limitations of their study which comes from secondary data usage. For example, lack of control for cross-contamination of samples, the fact that there may be incomplete taxa sampling, and other biases in the underlying database used. For example, they failed to do a comprehensive analysis looking for batch effects to ensure that samples were not systematically contaminated in data deposited from one organization.

We thank the reviewer for highlighting this. Although this study does use secondary data in the BOLD and SRA screens, our own primary dataset was generated via the targeted screen to prevent an overreliance on secondary data and of course its biases. Regarding the prospect of cross-contamination, this is unlikely for two reasons.
1) A majority of the multilocus profiles assessed from BOLD tend to give unique profiles which is reflected in our phylogenetic trees. Significant cross-contamination would tend to give identical strains.
2) If cross-contamination occurred between DNA extracts then it is likely that an mtDNA COI sequence would be retrieved (either from the original DNA extract or the contaminating one) rather than a Rickettsia COI sequence, as mtDNA is far more likely to amplify than Rickettsia when in competition.

Additionally, due to the aforementioned biases of using secondary data we have tried to be measured in our conclusions as a result of this. Specifically, we are not trying to claim that the Rickettsia sequences discovered in these databases are completely representative of Torix hosts in nature. Merely, that they allow for the discovery of new putative hosts and through combining several methods there is an indication that Torix Rickettsia are more widespread than previously thought and are overrepresented in aquatic insects.

I also have significant concerns over the lack of detail in the methods and not having access to the multiple sequence alignment used.

Sequence alignments, tree files etc. should already be available to the reviewer via the data management team (in the FTP server) at the journal. If this is not the case, we are happy to reupload the relevant data.

Other concerns/criticisms I had, include:

There are no methods for how samples were binned in Figure 1 either in the manuscript or in the figure. For example, how were bacteria contaminants v. non-bacteria contaminants determined? Was it a BLAST search. If so, what were the criteria? I suspect based on results presented Figures 2 and 3 that the criteria were not stringent enough.

BOLD compares COI sequences to common contaminants (e.g. human, bacteria) using BLAST-details can be found in Ratnasingham and Hebert, 2007 (doi:10.1111/j.1471-8286.2007.01678.x). The designation of bacterial contaminants by BOLD, from the dataset containing 3,817 non-target sequences, was confirmed by the taxonomic classification program, Kaiju, using default parameters. We took the sequences provisionally identified as bacterial before placing them phylogenetically with reference bacteria suggested by Kaiju. This has been highlighted in lines 450-465.

Line 154: Phylogenetic placement does not demonstrate these are of microbial origin. If I put a random sequence into the multiple sequence alignment, it would align and it would be in the phylogeny, by nature of the methods. Nothing about the tree or the topology suggests that didn't happen. In fact, some of the long branches may indicate that it did.

We have now included the usage of Kaiju which is a software program designed to designate taxonomic classification of sequences. For all sequences in the alignment used to create Figure 2, these were all identified as bacteria except one erroneously identified as eukaryotic which was later identified as Rickettsia on our phylogeny. Kaiju also allowed us to choose more specific reference sequences to include in our phylogenies. Aside from Rickettsia and Wolbachia, a significant minority of sequences

formed a monophyletic clade with the order Legionellales. In addition, we have now also included mitochondria in the tree on figure 2 to further verify the sequences are bacterial. This is discussed in lines 163-168 and 450-465.

With regards to long branches being problematic, Figures 2 and 3 were constructed as cladograms and not phylograms for neat presentation: branch lengths tell us nothing about clade designation. For transparency we have now included phylograms of figures 2 and 3 in Additional file 2 which demonstrate no long branches.

Since COI is derived from the mitochondrial genome, which is a microbe, language about "microbial origin" needs to be fixed throughout. Many consider organelles to still be microbes. If nothing else, their sequences (including COI) are of microbial origin.

We thank the reviewer for noting this. “Microbial origin” references have now been removed and we now refer to “bacteria” to distinguish from mitochondria throughout the manuscript.

The letters mean in Figure 2 are supposed to be the Wolbachia supergroups. But their placement seems quasi random. The sequences don't appear to be assigned to supergroups. If their placement corresponds to representative sequences, please specify that is the case, and make clear what the representative sequences are, and where they are on the tree.

The supergroup letters are for individual sequences. This has now been noted in the figure 2’s legend with accession details for sequences also clarified as being available in additional file 10.

Regardless, the phylogeny shows issues with very long branches around "A" from around 7 o'clock to 9 o'clock if the phylogeny were a 12-hour clock. This is peculiar. Is this an artifact of the tree rendering? Or the outgroup selection? Or some other problem—like the presence of Wolbachia lateral gene transfers that are no longer under selection? Or were sequences included in the analysis that aren't really from bacteria and is an methodological artifact?

As mentioned above, branch lengths do not say anything about genetic distance on cladograms. We have included phylograms in Additional file 2 for transparency and to show a lack of long branches within clades.

In general, there is no discussion or acknowledgement of the extensive literature on bacterial DNA integrations in host genomes, which for Wolbachia is extensive.

This has now been addressed in lines 352-355.

How much support is there for branches/nodes in the tree? I can see bootstrapping in the methods, but I don't see any indication of bootstrap support.

Bootstrapping is present on all trees in this manuscript and graphically represented as black, white and grey circles in figures 2 , 3, 4 and 5 and coloured circles in 6. This is indicated in the top left corner of all figures.

The multiple sequence alignment and unmodified phylogenetic files need to be made available to the reviewers and the readers either as online supplementary material or in a public repository with a permanent DOI.

As mentioned above, all of these files should already be available to reviewers via the FTP server of the journal.

Line 215-227, using the term prevalence is not correct. You do not know the full extent of prevalence of any of these organisms since you weren't targeting them with more specific primers with rigorous sampling. It is easy for this to be misconstrued and alternate terminology is needed.

“Prevalence” has now been changed to “frequency” throughout the manuscript when referring to the proportion of Rickettsia and Wolbachia deposits within the BOLD dataset.

Line 224: "indicating". There are other explanations as well, so I think using the word "suggesting" is more appropriate.

This has now been changed accordingly.

Line 235: The statement is too definitive for the data used. Yes, the stated p-value may be significant, but the statement and conclusions do not take into account the significant sampling bias in the SRA. But in addition, when I do the Fisher's Exact test I get 0.0550, which is not significant. The methods for the Fisher's Exact test and summary of the matrix is missing. My two by two matrix that yields a p-value of 0.0550 used presence/absence in the taxa in the table:

Aquatic Terrestrial
Has Torix Rickettsia 9 7
Does not have 49 107

Intuitively it isn't surprising it wouldn't be significant he difference is 20% v. 10% with more limited sampling of one than the other and low levels of detection overall.

We appreciate the reviewer's diligence in checking the Fisher's Exact test. However, the matrix presented by the reviewer does not consider Rickettsia subgroup and fails to account for multiple rows containing the same species (be it from a different population).

Subsequently, when taking these factors into account this is the matrix which was used in the submitted manuscript.
Aquatic Terrestrial
Has Torix Rickettsia 9 5
Does not have 49 106

Note that only 5 Torix Rickettsia are present in this matrix for terrestrial species because 2 of the 7 Rickettsia positive strains from the terrestrial species are not from the Torix group.

Since submission of the initial manuscript, table 1 has been updated to reflect previously missing Rickettsia positives detected in 3 spiders. With the addition of these spider positives, there is no significant difference between aquatic taxa and terrestrial taxa (p=0.1038).

However, when considering insects alone, this results in a p value of 0.0131. When controlled for taxonomic group (not all insect orders are represented in terrestrial and aquatic pools) the p value is still significant at 0.025. Subsequently, we have now suggested that the aquatic hotspot for Torix Rickettsia appears to apply for insects but not invertebrates in general. It should also be noted that the within-species sample sizes of terrestrial taxa in this study are often greater than aquatic suggesting that p values are conservative (positives are more likely to be found with greater sample sizes).

Details of Fisher's exact analyses have now been included in Additional file 7 and discussed in lines 245-261 and 554-564.

Line 300-301: what was the minimum criteria to say that a taxa has it? Merely a COI sequence? Or more? It seems given cross contamination of sequencing projects and other issues, that you need more than just the COI sequence in the BOLD database. Making it clear here is important to the discussion and interpretation of results.

The issue of cross-contamination has been addressed in our first response to the reviewer. Of course, ideally to confirm a true endosymbiosis, direct visualisation of the symbiont in the host's tissues is needed due to potential for the bacteria to come from ingested food or parasitism. However, previous studies have predominantly relied solely on PCR to identify putative hosts (as demonstrated in Table 2). To reflect this, we have changed the language accordingly to mention "putative hosts" where appropriate (lines 287, 296, 342, 389, 427). Additionally, we direct the reviewer to our response to reviewer 1, where we have screened SRA datasets to assess how likely contamination from ingested biota and parasitism is. Rickettsia-insertions into the host nuclear genome is also unlikely because all protein-coding genes from this study showed no signs of a frameshift, suggesting a lack of pseudogenization. Further, there are no well supported cases of Rickettsia inserts in the nuclear genome in the literature to date, a marked contrast to Wolbachia.

We agree with the reviewer that these points are important for the interpretation of the results and now

mention them in lines 337-350

Line 310: I'm not sure I agree with your logic. It might be that they fail because of Rickettsia or other bacterial DNA replication.

This argument has been removed from the paragraph.

Line 329: these conclusions seem premature given the data presented, since bootstrap support values or missing in this version reviewed.

We refer the reviewer to our previous response to bootstrapping.

Please check the legends in the additional files. I think Additional File 3 has a legend stating it is "Additional File 2". Likewise Additional File 2 has a legend stating it is "Additional File 1"

We thank the reviewer for flagging this. We have changed the legends accordingly.

Close
