# Supplementary material for: Torix Rickettsia are widespread in arthropods and reflect a neglected symbiosis
Source: Gigascience. 2021 Mar 25;10(3):giab021. doi: 10.1093/gigascience/giab021 (PMC7992394; doi:10.1093/gigascience/giab021)
Supplement: giab021_Response_to_Reviewer_Comments_Revision_1 [file giab021_response_to_reviewer_comments_revision_1.pdf]

Author's Response To Reviewer Comments

Close

We would like to thank the reviewer for their comments. Please find below a point-by-point response from the authors.

“The alignments seem to interchange N/n/-, but I checked the IQTree manual and IQTree treats them all the same. But I don't see how IQ tree handles phylogenetic inference at positions with N/n/-, which it calls "unknowns". For example, in the multigene alignment, I don't believe there is any position without gaps, so presumably it handles them. I'm assuming the alignments I'm looking at are the ones fed into IQTree and not the ones coming out of MAFFT and before Gblocks, but it might be good for you to confirm using this example: looking at the alignment BOLD_just_Rciekttsia_COI_contaminants_alignment.fas, these two sequences have absolutely no overlap, and are significant truncated relative to the complete alignment (and these aren't the only two with this problem). How is the algorithm dealing with this, and does it introduce any artifacts? Do you get the same result if you remove sequences like these and use only sequences where all positions of the alignment have a
character (both by removing short sequences like this and also trimming the ends of the alignment)?”

The reasoning behind including sequences with missing character data in our alignments is based on previous work demonstrating that missing data in most cases should not decrease phylogenetic resolution for taxa with complete data (Wiens 2006, DOI: 10.1016/j.jbi.2005.04.001). To confirm this, we reran a modified alignment of the BOLD_just_Rickettsia_COI_contaminants_alignment.fas file by trimming ends by 50 nucleotides and removing any remaining truncated sequences to get rid of missing data (169 of the original 807 sequences removed), as suggested by the reviewer. In accordance with Wiens’ observations, the generated tree (see FTP file ‘BOLD Rickettsia trimmed.png’) placed taxa into the same designated groups as the phylogeny with missing data in Figure 3 (Designations can be found at DOI:10.6084/m9.figshare.12801107). Additionally, the study cited above ran simulations to show that highly incomplete data can be accurately placed in phylogenetic trees as long as at least 50% of sequences contain complete data. Furthermore, it is suggested that the inclusion of sequences with missing data can also sometimes be better than exclusion as this additional data can subdivide misleading long branches. We have now included the reasoning behind using incomplete data in the methods section (lines 480-483).

“How do double infections confound the results? Do they behave erratically in the cladograms, like a chimera would? Can this be a wider problem in interpreting the results? It seems like it would have to be, but you have chimeras you can use to examine this. Using those, is there a way to address this? With SRA data it seems like you should be able to look for sequence heterogeneity in the reads. Not sure about BOLD data.”

Where double peaks were observed in 10/753 Rickettsia-associated taxa from BOLD, the base call was designated as ‘N’ (See FTP file ‘BOLD_multigene_Rickettsia_alignment.fas’). This prevents erroneous placement of chimeric strains on the phylogeny. For BOLD data we unfortunately cannot reconstruct trees by teasing apart the individual strains of the double-infections because we cannot know what phase the double-peaks are in.

The use of ‘N’ characters at double-peak sites could lead to potential problems in the interpretation of these 10 taxa at terminal branches of the phylogeny but the placement as Torix Rickettsia is not likely to be affected. Furthermore, these double-infections are a minority of the total taxa meaning their effects on interpreting results are likely to be minimal.

“Line 254-261: I think you need to add in a correction for multiple testing. You do at least two tests that are in the main text, but it sounds from the response to reviewer's comments that you did many more than that and are only reporting the ones that are $P<0.05$. However, you should report how many tests you did to find those two and adjust for multiple testing. Otherwise, if you do 20 tests, you would expect

to have 1 that is "significant" (for more information on multiple testing: http://www.biostathandbook.com/multiplecomparisons.html). In addition, I think it is important for people to know what comparisons were done that were not significant as these are also results. Addressing multiple testing seems like an issue throughout. In the methods other statistical tests were clearly undertaken where there was multiple testing."

Two Fisher's exact tests (aquatic vs terrestrial insects-1 controlled for insect order and 1 uncontrolled) were detailed in the main text and additional file 7, as these were the only taxonomically 'matched' pairs. However, one additional test was performed initially to compare terrestrial vs aquatic invertebrates in general which did not give a significant p-value due to a hotspot of Rickettsia in spiders, which are known to be a hotspot for all inherited symbionts tested to date (Wolbachia, Spiroplasma, Rickettsia: Goodacre et al. 2006, doi: 10.1111/j.1365-294X.2005.02802.x.; Cardinium: Duron et al. 2008, doi: 10.1111/j.1365-294X.2008.03689.x.). This detail has now been added in Additional file 7 and lines 266-269. Overall, only 3 tests were done (2 significant and 1 not significant) and this indicates that Torix Rickettsia are over-represented in aquatic insects but this may not be the case for invertebrates in general.

"Line 354: Several, maybe many, of the Wolbachia integrations have no mutations or frameshifts, particularly in insects. Those with frameshifts and mutations are easier to find and identify as integrations such that the number of integrations without frameshifts and mutations is likely an underestimate, particularly given how many groups are still screening Wolbachia sequences out before assembling insect genomes. I have no idea how often that happens with Rickettsia, but it seems like, particularly as more groups use tools like blobplots."

We thank the reviewer for raising this issue. We have now put a caveat at the end of this sentence to indicate that despite no frameshifts or mutations, it is still possible the sequences from this study are host integrations (lines 376-378).
The problem is likely to be less for Rickettsia than Wolbachia, due to differences in the mode of vertical transmission. Wolbachia is present in the germline stem cell niche, such DNA from the symbiont is available for incorporation into the germline. Rickettsia, in contrasts, usually invades the egg after meiosis, through the follicular epithelium. Thus, Rickettsia DNA is much less present in the germline of insects, making integration less likely.

"Line 366-379: This section still has issues with respect to the study design being secondary data analysis. These lines are in the discussion, it is the time to say things like on line 377 that the over-representation here in BOLD data (if that is the data you are referring to, because I can't remember which one was 17/19 and there is not here that clarifies) could be the result of an amplification bias—in not producing the host copy of the gene, amplifying the Rickettsia gene, or both. Those issues are profound in secondary data usage and need to be addressed head-on so that others who read the paper do not misconstrue the results. Likewise, the SRA data is not random, so I am not sure the statement on line 379-381 is correct, and at very least it needs qualifications. If it is correct, you need to better argue in the manuscript why it is correct, like that you used a sampling scheme to reduce bias, or something like that. Personally, I think it is better to acknowledge
the limitations that try to justify, as even if you have a sampling scheme, it can be biased. The PCR screen listed in the table of this manuscripts seems biased from my quick look (e.g. an over-representation of mosquitoes)."

The "17/19 strains" being Torix is a reference to the targeted screen (not the BOLD screen) which was used alongside the BOLD data because of the aforementioned biases relating to amplification bias and this has now been clarified on lines 401-402. Additionally, we have added a sentence to the results section explicitly quoting the 17 strains of Rickettsia found in the targeted screen (lines 248-250). Although 95% of Rickettsia amplifications from BOLD are Torix, we already mention that this is likely due to primer bias (lines 321-324). Subsequently, the targeted screen is used in part to negate the problems of relying entirely on secondary data.
Of course, many studies which aim to investigate the distribution of a symbiont will have sampling and methodological biases. However, having multiple screening strategies, as we have here, is likely to give a more nuanced and holistic view of Torix Rickettsia ecology. We believe that the combined use of several screening methods is a strength and not a weakness of the study. Despite this, we have now added a separate section detailing the limitations of the study (lines 358-388).

Specifically, regarding lines 379-381 (of the 1st revision), this statement is based not just on SRA data but also the targeted screen from this study and Weinert's study as mentioned in the previous lines.

Thus, the SRA is corroborating two separate targeted screens (one which lacked spiders and aquatic insects demonstrating a high number of Belli infections, and another which included spiders and aquatic insects demonstrating a high number of Torix infections.). Subsequently, for clarity we have now changed the statement "Our additional use of a bioinformatics approach based on the SRA appears to confirm that Belli and Torix are two of the most common Rickettsia groups among arthropods." to "Our additional use of a bioinformatics approach based on the SRA appears to corroborate targeted screen data indicating that Belli and Torix are two of the most common Rickettsia groups among arthropods." (lines 403-406).

"Line 387-388: please provide more details. I don't remember reading that. Pointing to an exact result, for instance of how many strains of the same MLST type are in different insect orders is necessary. It should have been in the results if it is in the discussion. In fact, if I look at the figures, the Wolbachia in Figure 2 actually seem to be grouped by insect host taxa at this level. The same is ture for Figure 3 for the Rickettsia. There are a few interleaved colors, but without knowing more I'm not convinced that it can't be explained in another way (like a mite on a host or in the gut from a carnivorous insect or even a double infection); I also can't tell which ones are identical and which ones are just similar. But even if I should infer it from the figures, it should be reported in the results and I didn't find it there. Maybe you are trying to state it in the subsequent sentence if one assumes that all blood feeders are the same taxa and all phloem-feeders are
the same taxa, but that isn't clear. (And at least blood feeding is a trait found in multiple diverse taxa)."

The inferences related to similar strains in distantly-related hosts is best observed in the multigene tree in figure 4 rather than the single gene trees of figures 2 and 3. For example, odonate strains are clearly interleaved between strains from other host orders. More specifically, the two Coenagrion strains have 100% identity to the Culicoides stigma strain in contrast to two other odoante (Polythore) strains where multiple SNPs are observed at all loci (See ftp file 'BOLD_multigene_Rickettsia_alignment.fas'). We thank the reviewer as this was not mentioned in the results but we have now included this on lines 209-211. Furthermore, regardless of exact MLST profiles for strains, taxa from most orders are represented in both Limoniae and Leech Torix subclades indicating a lack of grouping based on insect host taxa. The authors believe this concept is better represented in a phylogeny rather than a list of MLST profiles.

"And once again, I'm left wondering if there is a sampling bias. Are mosquitoes over-represented in the database? It some of the tables they seem over-sampled. Blood feeders and phloem feeders are often well sampled, given their important to human health and agriculture, respectively. But maybe more problematically, these results are being described but they are not clearly described in the results section. If I search for blood, I do not find any results that support this statement. When I search for phloem, there is a mention of them being found in phloem-feeding insects, but not that they are diverse, and I have no way of assessing that as a reviewer or reader. Yet, this result for phloem-feeding is also in the abstract as a taxa that is a hot-spot. I don't see it in the figures in a way that I understand (e.g. phloem-feeding isn't annotated). Additionally, there is no assessment here that convinces me it is a hot spot; there are no statistics to suggest it is overabundant, which would be required to be a hotspot (and any such statistics would need correction for multiple testing or some sort of FDR calculation)."

Mosquitoes are likely to be over-represented in the sequence read archive but as mentioned already in lines 142-144, a single dataset per species was extracted for analysis to negate oversampling of the same species. Although certain genera may still be oversampled, the only instance of mosquito Rickettsia being detected is in the Anopheles plumbeus population of the targeted screen. With regards to phloem-feeding insect strains, psyllids and other phloem-feeders are present in both Limoniae and Leech subclades suggesting again that strains are diverse within similar lifestyles. This is best seen in Figure 6 where both phloem-feeding and blood-feeding are annotated. The common patterns of infection in phloem-feeding bugs and blood-feeders are also already mentioned in lines 296-302 of the results. We agree that the common patterns or 'hot-spots' found in our data should come with caveats and we have now included this in the limitations part of the discussion where we clarify that common patterns of infection refer specifically to our datasets which although extensive, have some biases and may not completely represent Torix Rickettsia infection in nature (lines 358-371).

""as previously described" Is this in a different manuscript, or earlier in the manuscript? I suspect this means earlier in the paper, but it needs to be clear. Was the same alignment method used with MAFFT and Gblocks? Same for ModelFinder? If it is and all ML trees were inferred the same way, I would recommend that you have a methods section that describes this one for all alignments, maybe concluding with what is different (like the model)."
This refers to methods described earlier in the manuscript and yes, the same methods were used for all

ML trees. This suggestion is welcomed and has been included in lines 492-493.

“I've outlined some examples where the statements don't reflect what is presented in the results and the limitations of a secondary data analysis. But they are actually more numerous and pervasive than this. For instance, line 39-41 and 41-43 in the abstract have these issues. Likewise, Line 161-162 should read "Torix Rickettsia is the most common bacterial contaminant sequence currently in BOLD, a major barcoding project". This change reflects that this only holds for what has been barcoded thus far, and the issues with the fact you need both failed host amplification and successful bacterial amplification, and that the biodiversity represented in such projects have their own biases. It is so pervasive, I am not sure I found all the instances. Honestly, I think the paper would really benefit from a large clearly labeled section that more explicitly deals with all the limitations of the study, so others do not misconstrue the results for years into the future. It would make
the paper much stronger and definitely more rigorous.”

With regard to line 39-41 describing how our targeted PCR data supports the aquatic hotspot hypothesis we refer the reviewer to our response to the ‘multiple testing’ above. For lines 41-43, we have changed this sentence to include the caveat that this applies only to arthropod genome projects: “Furthermore, the analysis of 1,341 Sequence Read Archive (SRA) deposits indicates Torix infections represent a significant proportion of all Rickettsia symbioses found in arthropod genome projects.” We have also changed lines 161-162 to reflect a similar caveat for the BOLD data as suggested by the reviewer. As previously mentioned, we have now included a specific section in the discussion detailing the limitations of our datasets (lines 358-388).

Close
