# Supplementary material for: Torix Rickettsia are widespread in arthropods and reflect a neglected symbiosis
Source: Gigascience. 2021 Mar 25;10(3):giab021. doi: 10.1093/gigascience/giab021 (PMC7992394; doi:10.1093/gigascience/giab021)
Supplement: giab021_Reviewer_1_Report_Original_Submission — Steve Perlman -- 8/31/2020 Reviewed [file giab021_reviewer_1_report_original_submission.pdf]

Reviewer Report

Title: Torix Rickettsia are widespread in arthropods and reflect a neglected symbiosis

Version: Original Submission **Date:** 8/31/2020

Reviewer name: Steve Perlman

Reviewer Comments to Author:

Insects are commonly infected with bacterial symbionts, and these play incredibly important roles in the biology of their hosts. The best known insect symbiont is Wolbachia, which is thought to infect at least 25% of insect species. In this paper, the authors make the surprising discovery that a lineage of Rickettsia, called the Torix Rickettsia group (after one of their leech hosts), is at least as, if not more, widespread, particularly in aquatic hosts. While the best known strains of Rickettsia cause serious human diseases, and are vectored by blood-feeding arthropods, this is just a tiny sliver of Rickettsia diversity. This is an exciting discovery for the insect symbiosis field. The host range of this microbe is quite striking: arthropod, gastropod, and protist.

The authors use three different approaches to document Torix group diversity. They first show that most microbial contaminant sequences in the Barcode of Life database belong to Torix Rickettsia. This is because the commonly used animal barcoding primers, used to amplify mitochondrial COI, may also amplify Rickettsia COI (mitochondria evolved from an Alphaproteobacterial symbiont allied with Rickettsia). They use more primers on material stored at the Barcode of Life center to confirm some of these Rickettsia infections. They also pull out Torix Rickettsia 16S rRNA from many insect SRA datasets. Finally, they screen a large sample of arthropods (and a few gastropods) for Rickettsia, using PCR. They report these bacteria associated with a broad range of hosts, including parasitoids, aquatic insects, blood feeders, sap feeders, and molluscs. This paper will shine a light on a previously unrecognized group of symbionts; the next step will be to begin to understand how these microbes affect host biology. It will be useful for the barcoding community to be reminded of possible symbiont contamination. The approach used for pulling out microbes from SRA datasets will also be of general interest.

My only concern is that Torix group Rickettsia and their relatives have also been identified in protists, such as nucleariid amoebae. So I wonder how many of these Rickettsia, particularly in aquatic hosts, are symbionts of protists residing in animal guts. Have the authors tried to pull out protist 18S sequences from the SRA datasets (or tried to amplify protist genes via PCR, although that would be much more difficult)?

Minor comments:

Line 194 - Psyllidae spelling

Line 242 & Table 2 - Chaoboridae spelling

Line 251 - Simulium spelling

Lines 340 - I would replace refs 49 and 50 with Gehrer & Vorburger, Biol. Lett., 2012

Line 362 - this sentence is confusing because the citations refer to Rickettsia in the belli group

Table 2 - Siphonaptera spelling
Line 819 - Parentheses spelling

Methods

Are the methods appropriate to the aims of the study, are they well described, and are necessary controls included? Choose an item.

Conclusions

Are the conclusions adequately supported by the data shown? Choose an item.

Reporting Standards

Does the manuscript adhere to the journal's guidelines on [minimum standards of reporting?] Choose an item.

Choose an item.

Statistics

Are you able to assess all statistics in the manuscript, including the appropriateness of statistical tests used? Choose an item.

Quality of Written English

Please indicate the quality of language in the manuscript: Choose an item.

Declaration of Competing Interests

Please complete a declaration of competing interests, considering the following questions:

- Have you in the past five years received reimbursements, fees, funding, or salary from an organisation that may in any way gain or lose financially from the publication of this manuscript, either now or in the future?
- Do you hold any stocks or shares in an organisation that may in any way gain or lose financially from the publication of this manuscript, either now or in the future?
- Do you hold or are you currently applying for any patents relating to the content of the manuscript?
- Have you received reimbursements, fees, funding, or salary from an organization that holds or has applied for patents relating to the content of the manuscript?
- Do you have any other financial competing interests?
- Do you have any non-financial competing interests in relation to this paper?

If you can answer no to all of the above, write 'I declare that I have no competing interests' below. If your reply is yes to any, please give details below.

I declare that I have no competing interests

I agree to the open peer review policy of the journal. I understand that my name will be included on my report to the authors and, if the manuscript is accepted for publication, my named report including any attachments I upload will be posted on the website along with the authors' responses. I agree for my report to be made available under an Open Access Creative Commons CC-BY license (http://creativecommons.org/licenses/by/4.0/). I understand that any comments which I do not wish to be included in my named report can be included as confidential comments to the editors, which will not be published.

Choose an item.

To further support our reviewers, we have joined with Publons, where you can gain additional credit to further highlight your hard work (see: https://publons.com/journal/530/gigascience). On publication of this paper, your review will be automatically added to Publons, you can then choose whether or not to claim your Publons credit. I understand this statement.

Yes Choose an item.
