# Supplementary material for: Torix Rickettsia are widespread in arthropods and reflect a neglected symbiosis
Source: Gigascience. 2021 Mar 25;10(3):giab021. doi: 10.1093/gigascience/giab021 (PMC7992394; doi:10.1093/gigascience/giab021)
Supplement: giab021_Reviewer_2_Report_Original_Submission — Xin Zhou -- 9/6/2020 Reviewed [file giab021_reviewer_2_report_original_submission.pdf]

Reviewer Report

Title: Torix Rickettsia are widespread in arthropods and reflect a neglected symbiosis

Version: Original Submission **Date:** 9/6/2020

Reviewer name: Xin Zhou

Reviewer Comments to Author:

The manuscript entitled "Torix Rickettsia are widespread in arthropods and reflect a neglected symbiosis" investigated the diversity of the endosymbiont Rickettsia and its distribution across hosts. The authors started by screening the contaminant sequences already filtered from the Barcode of Life Data System, using similarity criteria against the known Rickettsia reference database. This analysis revealed that Rickettsia were the dominant non-target COI-like amplicons in the contaminant sequences, more abundant than Wolbachia, which was previously considered as the most common bacteria source of the barcoding contaminants. In addition, the Torix group was the most common sub-lineage within Rickettsia. As the prevalence of Rickettsia amplicons was affected by the similarity between the barcoding primer sequences and the corresponding region of the bacterial genome, the author further investigated the presence/absence of Rickettsia using 186 Rickettsia-containing samples and multiple DNA loci specifically designed to amplify these bacteria. The results proofed that 135 out of the 186 tested samples were amplified successfully with at least 1 Rickettsia genes, confirming the presence of Rickettsia in most of them. The authors also screened through the public SRA database containing 1341 arthropod species, using 16S sequences assembled from shotgun reads by the program phyloFlash. Finally, a targeted PCR screen for Rickettsia was carried out for 1612 individuals presenting 169 species covering both aquatic and terrestrial taxa. This result indicated that Rickettsia was more prevalent in aquatic/semiaquatic than in terrestrial arthropods.
Overall, this study employed multiple lines of investigations/evidence to reveal the diversity and distribution of Rickettsia. The range of data used in this study is diverse and impressive; the methods are reasonable; and the findings are relatively novel and important. The manuscript might be suitable for publication with GigaScience, but it can benefit from clarification of a number of issues, including technical details and standardization of a number of terms. I would be happy to recommend a final acceptance, if the following issues can be addressed in a revised version.
Abstract 38, 42-43: the introduction of the "aquatic hotspot" hypothesis and that the results were supporting this hypothesis was very appealing (l38), yet this was not addressed in the conclusion, which instead claimed that Rickettsia was associated with a number of habits (l42-43). As these habits were not linked to aquatic, and not introduced previously in the background, the logic flow here is rather difficult to follow.
69: Rickettsia has been estimated as being present in 20-24% of species. One would be very interested in learning whether this is confirmed/disapproved by the findings of the current study. Which part of the experimental design is set to answer this question? If no, what needs to be done to get a better idea?
79-88: It might be a good idea to add something here about the diversity of subgroups of Torix. The results later on revealed two subgroups (Leech and Limoniae), but are these good representatives of the

diversity within Torix? How many subgroups are already known?
90-102: The use of terms Rickettsia CoxA, COI, Rickettsia COI are confusing. If Rickettsia CoxA and Rickettsia COI are actually referring to the same Rickettsia gene, the term needs to be standardized.
106: does the "template" here refer to DNA extract/aliquot? "Template" in the context of DNA template is primarily used in the description of amplification reaction, which doesn't seem to be the case here. This term is somewhat confusing. As you used "DNA extract" later in the text, I would suggest that these terms be unified.
109: "function more broadly" here is also vague. Do you mean that the primers used in these PCR assays are more degenerate or specifically designed to target Rickettsia genes? Please clarify.
123-125: "...deemed as contaminant sequences as a result of not matching initial morphotaxa assignment". I don't think that this is entirely accurate. A significant proportion of barcodes in BOLD are not matching initial morphotaxa assignment, at varied taxonomic levels. These include mis-identification, ambiguous/unstable taxonomic status, lab contaminations, etc. I would assume that BOLD uses an algorithm to confirm the sequence as being contaminants, only when they are matched to the most common non-target contaminants, e.g., bacteria, human etc.
125-128: the term "specimens" needs to be clarified. Do these include those that didn't yield a DNA sequence?
142: Explain targeted PCR Rickettsia screen. Does it employ specific primer sets designed for Rickettsia? Although this was described in the method section, a brief explaining of the method would help the readers to understand the context.
149: Should "Analyses" be "Results"?
160-161: "further unique bacteria contaminants were also detected", where are these results? Please cite.
167-170ï¼š if the BOLD results does not seem to support the aquatic hotspot theory, why?
170-172: the predominance report of Rickettsia from Canada seems meaningless, given the strongly biased sampling in BOLD (supplementary Fig. 1)
180: this is confusing, does it mean that the Torix sequence is identical to that of C_LepFolR at the 3' end? Or does it have a SNP but different from that of other bacteria?
185: How were these 186 Rickettsia-containing samples selected from 753 samples?
192: So how many subgroups of Torix are known? How well the findings represent the diversity?
207: define attempted barcodes
211: Here you used "genomic extracts", is this equivalent to "template"? Try to standardize terms.
217: again, why BOLD taxa with the most presence of Rickettsia NOT associated with aquatic lifestyle?
233-235: why did the comparison between aquatic/terrestrial arthropods only consider the targeted Rickettsia screen results, NOT that of SRA search?
269-270: This is somewhat misleading. This might imply that these two groups of bacteria cooccur in the same organisms, and the amplification of R is easier than W. I don't think the current experimental design is able to proof or deny this possibility.
308-310: we know that there are many other possibilities that might cause barcoding failure. At least provide some alternative causes to avoid biased argument.
415-416: what are the exact criteria when choosing these DNA templates?
428: does "linear" mean non-recombined sequence?

438-439: does this mean that the hosts were NOT identifiable by morphology?
459-461: What if the sequence was matched to more than one barcode at >98% identity?
489-497: Please provide more details on the analysis of phyloFlash, e.g., parameters used. I am a bit concerned about the assembling process employed here. 16S assembling can be difficult/impossible when metagenomics data contain more than 1 bacterial species or multiple variable copies of 16S, both of which might be the case for Rickettsia.
Table 1: for species without a definite identification to the species level (e.g., Pachycrepoideus sp.), do we know that all specimens analyzed here actually belong to the same species? I assume this can be confirmed using barcodes.
Figure legends for Figs. 2 and 3: the term "No colour" is misleading. I thought these would refer to those without any background colors (e.g., Rickettsia lineage in Fig. 2).
Fig. 2: So all Rickettsia in this tree were not from non-BOLD reference (says the Fig legend)? If the number in parenthesis represent the number of sequences, why is there only a single tip for Rickettsia? Are they collapsed? If yes, does it mean that the genetic divergence within Rickettsia is much smaller than that within Wolbachia?
Fig. 5: Is the lineage distribution associated with methodology used in discovering these sequences (SRA vs. targeted PCR screening)? Provide statistics.
Fig. 6: Move the vertical bars representing Typhus, Transitional, Spotted fever, and Bellii, further to the right so that they are in line with that of Torix. My understanding is that these lineages belong to the same hierarchic level under Rickettsia.

Methods

Are the methods appropriate to the aims of the study, are they well described, and are necessary controls included? Choose an item.

Conclusions

Are the conclusions adequately supported by the data shown? Choose an item.

Reporting Standards

Does the manuscript adhere to the journal's guidelines on minimum standards of reporting? Choose an item.

Choose an item.

Statistics

Are you able to assess all statistics in the manuscript, including the appropriateness of statistical tests used? Choose an item.

Quality of Written English

Please indicate the quality of language in the manuscript: Choose an item.

Declaration of Competing Interests

Please complete a declaration of competing interests, considering the following questions:

- Have you in the past five years received reimbursements, fees, funding, or salary from an organisation that may in any way gain or lose financially from the publication of this manuscript, either now or in the future?
- Do you hold any stocks or shares in an organisation that may in any way gain or lose financially from the publication of this manuscript, either now or in the future?
- Do you hold or are you currently applying for any patents relating to the content of the manuscript?
- Have you received reimbursements, fees, funding, or salary from an organization that holds or has applied for patents relating to the content of the manuscript?
- Do you have any other financial competing interests?
- Do you have any non-financial competing interests in relation to this paper?

If you can answer no to all of the above, write 'I declare that I have no competing interests' below. If your reply is yes to any, please give details below.

I declare that I have no competing interests

I agree to the open peer review policy of the journal. I understand that my name will be included on my report to the authors and, if the manuscript is accepted for publication, my named report including any attachments I upload will be posted on the website along with the authors' responses. I agree for my report to be made available under an Open Access Creative Commons CC-BY license (http://creativecommons.org/licenses/by/4.0/). I understand that any comments which I do not wish to be included in my named report can be included as confidential comments to the editors, which will not be published.

Choose an item.

To further support our reviewers, we have joined with Publons, where you can gain additional credit to further highlight your hard work (see: https://publons.com/journal/530/gigascience). On publication of this paper, your review will be automatically added to Publons, you can then choose whether or not to claim your Publons credit. I understand this statement.

Yes Choose an item.
